
JOURNAL INFORMATION
==============================
NLM Title Abbreviation: Chem Senses
Journal ID: Chem Senses
NLM Journal ID: 8217190
Journal ID: chemse

Chemical Senses

ISSN: 0379-864X
EISSN: 1464-3553
Publisher: Oxford University Press

ARTICLE INFORMATION
==============================
PMCID: PMC12746835
DOI: 10.1093/chemse/bjaf055
Article ID: bjaf055
Article version: 1
Subjects: Abstracts, AcademicSubjects/SCI01180

ECRO 2025 CONFERENCE
Annual Meeting of the European Chemoreception Research Organization, ECRO 2025
Bizkaia Aretoa, Bilbao, Basque Country, Spain, 15-18 September 2025

Publication date: 2025
Electronic publication date: 2025 Dec 29
Volume: 50
Electronic Location ID: bjaf055
2025 Dec 29
Copyright: © The Author(s) 2025. Published by Oxford University Press.
Copyright year: 2025
License: This is an Open Access article distributed under the terms of the Creative Commons Attribution License (https://creativecommons.org/licenses/by/4.0/), which permits unrestricted reuse, distribution, and reproduction in any medium, provided the original work is properly cited.
License URL: https://creativecommons.org/licenses/by/4.0/

Page count: 88

==============================

Chemical Senses https://doi.org/10.1093/chemse/bjaf055

### ABSTRACTS

### ECRO 2025 CONFERENCE
Annual Meeting of the European Chemoreception Research Organization, ECRO 2025
Bizkaia Aretoa, Bilbao, Basque Country, Spain, 15-18 September 2025

**Organizing committee:** Laura López-Mascaraque, Pablo Chamero, Enrique Lanuza, Gaskon Ibarretxe, Carolina Gómez-Díaz, Carlos Matute, Almudena Ramos, Laura Gómez-Santos, Ainara Vallejo, Lucía Jiménez-Rojo, José Ramón Pineda

**abstract citation id: bjaf055.001**

### 1. KEYNOTE LECTURES

### Invisible Heritage: smells as cultural records

*Cecilia Bembibre*[1]

[1]*University College London, London, United Kingdom*

Smell holds deep cultural and historical meaning, yet it has long been overlooked in heritage practice. Olfactory heritage is an emerging field that recognises and protects the scents associated with places, objects, and traditions. These odours are often transient, but they carry strong connections to identity, memory, and lived experience. Preserving them supports resilience and sensory continuity in times of environmental, social, and material change.

In recent years, through my work at University College London and with partners across the world, I have developed methods for identifying and documenting historically significant smells. This includes chemically analysing volatile compounds in materials such as historic potpourri, ancient embalming substances, and old manuscripts. We have also worked with museums and heritage professionals to interpret and communicate these smells through public exhibitions and participatory experiences.

But this work isn't only about conservation. Olfactory heritage connects science with human experience. These real-world smells help researchers study how memory works, how emotions are triggered, and how the brain processes complex information. They offer new ways to explore how the senses work together, and how cultural context shapes perception.

Our findings show that olfactory heritage contributes to cultural sustainability by safeguarding sensory knowledge that might otherwise be lost. It helps communities maintain links to past environments, traditions, and everyday practices. Through reconstructed scents and archival approaches, olfactory heritage supports intergenerational knowledge transfer, enabling archives beyond written records and images.

At a time when many valuable smells are at risk, preserving scent offers a new way to protect cultural diversity. It adds value to heritage conservation by considering the sensory dimension of place, and it invites new cross-disciplinary collaborations across chemistry, perfumery, computer science, history, and the arts.

In this presentation, I will share examples of recent research and practical applications in the field, as well as ways in which olfactory heritage can contribute to other disciplines. It will make the case for recognising smell as part of our intangible heritage, and for including olfactory knowledge in how we care for and interpret the cultural record.

**abstract citation id: bjaf055.002**

### Neurogenesis for adult olfactory circuits

*Isabel Fariñas*[1]

[1]*CIBERNED & Instituto de Biotecnología y Biomedicina, Universidad de Valencia*

No abstract available.

**abstract citation id: bjaf055.003**

### Sensing with pheromones in freely moving mice

*Lisa Stowers*[1]

[1]*The Scripps Research Institute, La Jolla, Unites States*

No abstract available.

**abstract citation id: bjaf055.004**

### Brain myelin as an energy source in health and disease

*Carlos Matute*[1]

[1]*CIBERNED, IIE Biobizkaia, Universidad del País Vasco (UPV/EHU), Spain*

No abstract available.

© The Author(s) 2025. Published by Oxford University Press.
This is an Open Access article distributed under the terms of the Creative Commons Attribution License (https://creativecommons.org/licenses/by/4.0/), which permits unrestricted reuse, distribution, and reproduction in any medium, provided the original work is properly cited.

**abstract citation id: bjaf055.005**

### Locust olfactory neuroecology

*Bill Hansson*[1]

[1]*Max Planck Institute for Chemical Ecology.Department of Evolutionary Neuroethology.Germany*

No abstract available.

**abstract citation id: bjaf055.006**

### Mixology

*Diego Cabrera*[1]

[1]*Guru Lab, Madrid, Spain*

No abstract available.

**abstract citation id: bjaf055.007**

### 2. SYMPOSIA

### 150 YEARS OF GLOMERULI

### *Symposium Organizer: Peter Mombaerts*

### Similar and yet different: The glomerulus in amphibians

*Ivan Manzini*[1]

[1]*Justus Liebig University Giessen. Germany*

In vertebrates, it is generally assumed that each olfactory receptor neuron extends a single, unbranched axon to innervate a single glomerulus within the olfactory bulb. This view, however, is primarily derived from studies in rodents. In contrast, in all amphibian species examined so far, axons of olfactory receptor neurons regularly bifurcate and project to multiple glomeruli. Such branching has been observed in both larval and postmetamorphic stages across amphibians with diverse ecological adaptations. Moreover, in amphibians, as well as in teleost fishes and reptiles, the projection neurons within the olfactory bulb often possess multiple dendrites that frequently contact different glomeruli. These anatomical features suggest that both the input and output architecture of olfactory glomeruli is more diverse across vertebrates than previously assumed.

The atypical organization observed in amphibians may underlie an alternative mechanism of olfactory processing. Functional studies in larvae of the African clawed frog (*Xenopus laevis*) support the notion that amphibians process odor information differently from rodents.

Together, these findings challenge the assumption that unbranched receptor neuron axons are a universal trait among vertebrates. They also highlight that the fundamental wiring of glomeruli and the vertebrate olfactory bulb is not conserved across all taxa. To achieve a more comprehensive understanding of the vertebrate olfactory circuitry and its functional implications, future research must include representative species from all major vertebrate classes, namely, fishes, amphibians, reptiles, birds, and mammals.

**abstract citation id: bjaf055.008**

### Convergence and divergence: The glomerulus as a physiological unit

*Izumi Fukunaga*[1]

[1]*OIST Graduate University. Japan*

As the site of convergence for olfactory sensory neurons that express the common olfactory receptor, the glomerulus may be thought of as a functional unit of olfactory processing. Indeed, neurons that belong to a common glomerulus show correlated sensory tuning and synchronised activity. Yet, the glomerulus is known to be more than just a relay for olfactory information, with new data emerging about the heterogeneity of the olfactory bulb outputs. I will discuss our current understanding of, and outlook to get greater insight into, the physiology of this compact but complex circuity.

**abstract citation id: bjaf055.009**

### BITTER TASTE ENIGMA – RECEPTORS, CIRCUITS, AND PERCEPTION

### *Symposium Organizer: Anan Moran*

### Molecular and functional evolution at the origin of vertebrate bitter taste

*Sigrun I Korsching*[1], *Sefatullah Zakary*[1], *Maik Behrens*[2]

[1]*Institute of Genetics, Faculty of Mathematics and Natural Sciences, University of Cologne. Germany*
[2]*Leibniz Institute for Food Systems Biology at the Technical University of Munich. Germany*

Many plant and animal species synthesize toxic substances to deter potential predators. In vertebrates such compounds are detected by bitter taste receptors, T2Rs, which belong to the rhodopsin-associated group of G protein-coupled receptors.

T2Rs appear to have evolved as duplication of an olfactory receptor. The T2R or TAS2R family has expanded to sometimes over one hundred genes in the lobe-finned lineage (coelacanth and tetrapods), whereas it stayed much smaller in the ray- finned lineage (e.g. zebrafish). In addition to their function in avoiding ingestion of toxic food, T2Rs may also serve as monitors of internal metabolites, e.g. endogenous steroids. It is not known, which of these two functions came first during evolution.

In phylogenetic analysis we observe a singular T2R receptor in sharks and rays, which constitutes a sister group to the T2Rs of all bony vertebrates. In bony fish, including the 'living fossil' coelacanth, we detect an unusually conserved basal T2R receptor. We have analysed the ligand repertoires of these conserved T2Rs from both shark and bony fish using HEK 293T- Gα16gust44 cells in a high throughput heterologous expression assay and calcium imaging as read-out. We have computationally modeled these conserved receptors by I-TASSER, GPCR-I-TASSER, and Protenix Server using AlphaFold3.

Docking studies using TomoDock identified two well-separated clusters of ligand positions: one cluster near the extracellular surface likely constitutes a vestibular site, while the second lies deeper within the seven-transmembrane helical bundle, and presumably represents the orthosteric site.

We will discuss these results in the context of the question of original function for T2R receptors.

**abstract citation id: bjaf055.010**

### The interaction of agonists, antagonists and mixtures with the human TAS2R repertoire

*Maik Behrens*[1]

[1]*Leibniz-Institute for Food Systems Biology at the Technical University of Munich. Germany*

Human bitter taste perception is important to identify the presence of potentially pharmacological active compounds in food. The ~25 subtypes of bitter taste receptors, called taste 2 receptors (TAS2Rs), are expressed in sensory cells within the oral cavity. As the number of known bitter compounds collected in curated databases such as the bitterDB exceeds that of the receptors by far (about 1000 to 25), bitter taste in human is considered highly complex.

To functionally characterize the human TAS2R repertoire we employ a well-established heterologous expression assay that allows to monitor the activation of individual receptors expressed in HEK 293T-Gα16gust44 cells on a high-throughput measuring platform. Initially, our experiments contributed highly successfully to deorphanize TAS2Rs and to assess their individual breadth of tuning leading to a categorization into broadly, intermediately, and narrowly tuned receptors.

The subsequent identification of bitter taste receptor antagonists enhanced the complexity of human bitter perception even further. The presentation will highlight some of the recent findings on 1.) bitter compounds and antagonists competing with each other for the same receptors, 2.) the fact that sequential consumption of bitter compounds affects perception, 3.) the local environment of TAS2Rs modulate their signaling. These findings, together with the fact that the human TAS2Rs exhibit pronounced genetic variation and that endogenous agonists might interfere with their evolutionary capability to adapt to food sources, add to the enormous complexity of bitter perception.

**abstract citation id: bjaf055.011**

### Neural and behavioral responses to bitterants: disentangling perception and coding in the taste system

*Oren Mazon*[1], *Dan Ben-Ezra*[1], *Anan Moran*[1]

[1]*Tel Aviv University. Israel*

The bitter taste sensation has evolved as a crucial defense mechanism, alerting organisms to potentially harmful substances and thereby enhancing survival. At the same time, bitterness contributes to the richness of culinary experiences and may indicate the presence of beneficial medicinal compounds. Thus, the bitter taste system in animals, including humans, must be finely tuned to balance the potential risks and benefits associated with bitterants.

Surprisingly, whether different bitterants can be perceptually distinguished remains an open question. Both animal and human studies suggest that, even if such discrimination is possible, it is a difficult task and the taste system may not be optimized for it. This body of evidence appears to contrast with neurophysiological findings from brainstem regions, which show distinct neuronal responses to different bitterants. This discrepancy raises intriguing questions: How can neurons differentiate between bitterants when the subject cannot? And what might this reveal about the brain's design in relation to consciousness and perception?

To address these questions, we combined behavioral experiments with electrophysiological recordings from multiple brain regions involved in taste processing in rats. The behavioral studies aim to determine the extent to which bitterants can be distinguished when intensity is controlled for, using both discrimination tasks and preference testing. In a separate study we recorded neuronal activity in the gustatory cortex, basolateral amygdala, and prefrontal cortex of freely behaving rats in response to varying concentrations of different bitterants.

In this talk, I will present findings from both lines of investigation, highlighting the relationship between perceptual experience and neural coding in the bitter taste system.

**Funding:** Israel Science Foundation

**abstract citation id: bjaf055.012**

### CELL DIVERSITY AND PLASTICITY IN THE MOUSE OLFACTORY SYSTEM

### *Symposium Organizer: Moritz Nesseler*

### Cell Diversity and Plasticity in the Mouse Olfactory System

*Moritz M Nesseler*[1], *Lena Terlau*[1], *Johanna Flesch*[1]

[1]*RWTH Aachen University. Germany*

Mammals rely on their sense of smell to navigate and interact with their environment. Accordingly, chemosensation is fundamental to a variety of behaviors, including foraging, social recognition, mate choice, territory definition, and others. Evolutionarily, these vital functions served as the driving force for the refinement of the olfactory system. As chemosensory specialists, mice are an ideal model organism to analyze the mechanisms underlying olfactory physiology and integration. Here, we present recent research along the mouse olfactory pathway, thereby highlighting the intricate interactions between various cell types that contribute to the complexity and plasticity of the olfactory system. Speakers in this symposium ensure a broad representation of olfactory research across molecular, cellular, systems, and behavioral domains. Shawn D. Burton (Lehigh University) will present the role of main olfactory bulb interneurons in the coordination of mitral cell ensembles. Andres Hernandez-Clavijo (RWTH Aachen University) will showcase the cellular diversity of the accessory olfactory bulb. Next, Federica Genovese (Université Paris Cité) will focus on the role of trigeminal activation in olfactory processing. Kevin Bolding (Monell Chemical Senses Center) will present the multi-locus circuitry and population dynamics during initial odor experience.

**abstract citation id: bjaf055.013**

### Novel cell types mediating pheromone information processing in the mouse accessory olfactory bulb

*Andres Hernandez-Clavijo[1], Uday Rangaswamy[2], Remo Sanges[2], Marc Spehr[1]*

*[1]RWTH Aachen University. Germany*
*[2]SISSA. Italy*

In mice and most mammals, chemical communication is crucial for regulating behavior. Pheromones and other social chemosignals are detected by the vomeronasal organ (VNO). These signal are transmitted to the accessory olfactory bulb (AOB) in the dorsal-posterior region of the olfactory bulb. The AOB processes pheromonal information and connects directly to the amygdala-hypothalamic complex, which governs behavioral responses and internal body states. Despite its central role in pheromone processing, the cellular composition and functional organization of the AOB remain largely unexplored. Here, we employed a multifaceted approach to uncover the cellular diversity of the AOB. We performed single-cell transcriptomic analysis of the AOB in both male and female mice, establishing a robust dataset that captures the cellular heterogeneity of this brain region. Subsequently, we applied in situ spatial profiling techniques to map the distribution of hundreds of genes within the tissue at subcellular resolution. Integrating these single-cell transcriptomic data with spatial distribution maps revealed the organization of distinct AOB cell populations in the tissue. To further characterize individual cell types, we performed patch-clamp recordings combined with high-resolution microscopy to assess electrophysiological properties and morphological features. Post-hoc analysis of marker gene expression in the recorded cells allowed us to integrate transcriptomic, spatial, electrophysiological, and morphological data, providing a multidimensional description of AOB cell types. This study identifies previously uncharacterized neuronal subtypes and represents a comprehensive analysis of cellular diversity in the murine AOB. Our findings build a solid basis for future research aimed at unravelling the complete cellular composition and functional organization of this brain region, enhancing our understanding of its role in pheromone information processing and behavioral regulation.

**abstract citation id: bjaf055.014**

### A low-dimensional glomerular code for olfactory perception

*Walter Bast[1], Cina Aghamohammadi[2], Priyanka Gupta[1], Tatiana Engel[2], Florin Albeanu[1]*

*[1]Cold Spring Harbor Laboratory. United States*
*[2]Princeton Neuroscience Institute, Princeton University. United States*

Despite significant advances in olfaction, the relationship between odorant receptor (OR) activity patterns and odor percepts remains poorly understood. We developed a novel approach to investigate how OR response spectra are related to perceived stimulus similarity. Disentangling this relationship requires controlling olfactory stimuli at the level of OR types; we exploited the anatomical clustering of ORs to individual glomeruli to address this challenge. Using two-photon and widefield imaging in mice, we identified numerous glomeruli and determined their responses to 121 odorants. We then created synthetic olfactory stimuli by patterned optogenetic activation of selected glomerular combinations. To determine the perceptual distances between these glomerular sets, we trained mice to report perceived differences between a reference stimulus and other glomerular patterns. We found that individual glomeruli within the reference set differ in perceptual relevance, as some glomeruli contribute more to the reference stimulus percept than others. This distinct relevance is not dictated solely by individual OR identities but emerges from the response spectra of the entire glomerular reference set. To investigate how odorant responses determine the perceptual similarity of sets of photo-activated glomeruli, we developed an unsupervised, autoencoder-based method to extract latent factors from glomerular response profiles. This approach captured a ~12-dimensional manifold that represents the response spectra of about 40 glomeruli used as inputs in each animal with >90% accuracy. We further trained a model of behavioral performance on these latent factors that successfully predicted individual animals' responses to novel glomerular sets. These findings provide new insight into how OR activation patterns are mapped to olfactory percepts and indicate that olfactory perception is low-dimensional and inherently structured for efficient odor representation.

**Funding:** Swartz Foundation; ISEQB Program (Simons & CSHL); NIH grant 5R01DC014487; NIDCD

**abstract citation id: bjaf055.015**

### From detonation to dendritic spikes: how olfactory bulb interneurons coordinate flexible ensembles of mitral/tufted cells

*Shawn Burton*[1], *Christina Malyshko*[1], *Nathan Urban*[1]

[1]*Lehigh University. United States*

Inhibitory granule cells densely populate the mammalian olfactory bulb, where recurrent and lateral inhibition is critical to reformatting olfactory information as it propagates from peripheral receptors to downstream cortex. Yet functional investigation of granule cells has consistently revealed extremely sparse connectivity and predicted vanishingly weak synaptic output. Whence then cometh bulbar inhibition? To answer this, we have begun directly interrogating unitary synaptic interactions between olfactory bulb projection neurons and a less abundant but highly conserved population of anaxonic external plexiform layer interneurons in acute mouse brain slices. In recent work, we used physiological, morphological, neurochemical, and synaptic analyses to show that this interneuron population subdivides into two major subtypes, with fast-spiking interneurons perisomatically innervating projection neurons to mediate robust recurrent and lateral inhibition driven by unusually prevalent synaptic detonation and high sensitivity to projection neuron synchrony. In subsequent study, we reveal two distinct modes of fast-spiking interneuron activation: global spikes, driving cell-wide synaptic output, and dendritic spikes, manifesting as spikelets in somatic recordings and triggering local synaptic output. These distinct activation modes thus enable fast-spiking interneurons to coordinate flexible ensembles of projection neurons, motivating further investigation into how fast-spiking interneuron activation mode may vary across distinct brain states.

Toward this end, we find prominent raphe innervation of the external plexiform layer and divergent serotonergic modulation of fast-spiking interneurons and granule cells, driving fast-spiking interneurons into global spiking and supporting a state of widespread lateral inhibition. Collectively, these and other findings provide new mechanistic insight into key contributions of diverse interneurons to early olfactory processing.

**Funding:** Funded by NIH grants R01DC021296 to SB and R01DC016560 to NU.

**abstract citation id: bjaf055.016**

### Synaptic inhibition in the mouse olfactory bulb refines multimodal perception of mechanical and chemical stimuli

*Susobhan Das*[1], *Sarang Mahajan*[1], *Debarghya De*[1], *Itishree Kaibarta*[1], *Sanyukta Pandey*[1]

[1]*Indian Institute of Science Education and Research, Pune. India*

Our sense organs gather information from the external world and convert it into neural representations, which are then refined by pre-cortical and cortical regions to facilitate perception and decision-making. In nature, the sensory landscape is dynamic and often covert. The brain's ability to process and integrate relevant sensory stimuli is crucial for the survival and adaptability of any living organism. Unlike humans, rodents primarily use olfactory and vibrissal cues. The rodent olfactory system is capable of processing various sensory stimuli. In vitro studies have demonstrated the mechanical sensing properties of olfactory sensory neurons. Previous study from our lab proved that mice can detect and discriminate mechanical stimuli using their olfactory system. However, the contribution of olfactory bulb (OB) circuitry in integrating and refining multimodal mechanical and chemical stimuli remains unexplored. Our results show that concomitant pairing of subthreshold mechanical and odor stimuli (subthreshold multimodal/SMMD) enhances discriminability. Interestingly, the endoscopic calcium imaging experiments uncovered higher population activity of OB inhibitory interneurons for SMMD stimulations compared to the unimodal cues, implying improved discriminative capacity due to higher inhibition. We further employed optogenetics for bidirectional modulation of these OB interneurons to understand their role in subthreshold multimodal stimuli refinement. Notably, altering inhibition in either direction posed deleterious effects on the discriminability of subthreshold multimodal stimuli in contrast to improved discriminative capacity upon increasing inhibition in unimodal subthreshold conditions. Therefore, our results establish the role of OB inhibitory interneuronal network in the refinement of multimodal perception of mechanical and chemical stimuli.

### References

1 Abraham, N. M., Egger, V., Shimshek, D. R., Renden, R., Fukunaga, I., Sprengel, R., Seeburg, P. H., Klugmann, M., Margrie, T. W., Schaefer, A. T., & Kuner, T. (2010). Synaptic inhibition in the olfactory bulb accelerates odor discrimination in mice. Neuron, 65(3), 399–411. https://doi.org/10.1016/j.neuron.2010.01.009

2 Grosmaitre, X., Santarelli, L. C., Tan, J., Luo, M., & Ma, M. (2007). Dual functions of mammalian olfactory sensory neurons as odor detectors and mechanical sensors. Nature neuroscience, 10(3), 348–354. https://doi.org/10.1038/nn1856

3 Connelly, T., Yu, Y., Grosmaitre, X., Wang, J., Santarelli, L. C., Savigner, A., Qiao, X., Wang, Z., Storm, D. R., & Ma, M. (2015).

G protein-coupled odorant receptors underlie mechanosensitivity in mammalian olfactory sensory neurons. Proceedings of the National Academy of Sciences of the United States of America, 112(2), 590–595. https://doi.org/10.1073/pnas.1418515112
4 Iwata, R., Kiyonari, H., & Imai, T. (2017). Mechanosensory-Based Phase Coding of Odor Identity in the Olfactory Bulb. Neuron, 96(5), 1139–1152.e7. https://doi.org/10.1016/j.neuron.2017.11.008
5 Fukunaga I, Berning M, Kollo M, Schmaltz A, Schaefer AT. Two distinct channels of olfactory bulb output. Neuron. 2012 Jul 26;75(2):320-9. doi: 10.1016/j.neuron.2012.05.017. PMID: 22841316.
6 Mouse olfactory system acts as anemo-detector and -discriminator Sarang Mahajan, Suhel Tamboli, Susobhan Das, Anindya S. Bhattacharjee, Meenakshi Pardasani, Priyadharshini Srikanth, Shruti D. Marathe, Avi Adlakha, Lavanya Ranjan, Sanyukta Pandey, Nixon M. Abraham bioRxiv 2024.08.28.610087; doi: https://doi.org/10.1101/2024.08.28.610087

**abstract citation id: bjaf055.017**

### The role of trigeminal activation in olfactory signal processing

*Federica Genovese[1], Robert Pellegrino[2], Aiden Streleckis[2], Matthew Andres[2], Johannes Reisert[2], Joel Mainland[2]*

*[1]Université Paris Cité. France*
*[2]Monell Chemical Senses Center. United States*

Most volatile compounds entering the nasal cavity activate both olfactory sensory neurons (OSNs) and chemosensory trigeminal fibers, eliciting olfactory and somatosensory (e.g., irritation) sensations. In the olfactory epithelium (OE) OSNs are densely interspersed with trigeminal sensory fibers, which respond together to volatile compounds entering the nasal cavity. By adding CO2, an odorless pure trigeminal agonist, to the odorant stimulus (2-phenylethanol [PEA]), we evaluated how the trigeminal activation affects the neuronal responses in a mouse model and human odor perception. In the mouse model, electro-olfactogram recordings from the OE showed enhanced OSN responses to bimodal stimuli (PEA + $CO_2$) compared to PEA alone. This enhancement was absent in mice lacking the trigeminal receptors TRPA1 and TRPV1, indicating that trigeminal input modulates olfactory signaling at the periphery.

We next examined whether trigeminal modulation influences human odor perception. Fifteen panelists rated the perceived intensity of PEA, $CO_2$, and their mixtures using a generalized Labeled Magnitude Scale (gLMS), with stimuli delivered via gas- sampling bags. Adding 10% $CO_2$ significantly increased perceived intensity compared to either component alone. At 40% $CO_2$, the mixture's intensity matched that of $CO_2$ alone. To model these interactions, we tested several approaches, including linear addition, vector (Euclidean; EUC), U model, and strongest component (SC). We determined that mild trigeminal activation (10%) is best predicted by the EUC model, while strong activation (40%) was better described by the SC model.

Our findings reveal that trigeminal-olfactory interactions shape odor perception in a stimulus-dependent manner starting from the periphery of the olfactory system, underscoring the need to account for multisensory integration in models of olfactory processing.

### References

1 Genovese F*, Xu J, Tizzano M, Reisert J. Quantifying Peripheral Modulation of Olfaction by Trigeminal Agonists. J Neurosci. 2023 Nov 22;43(47):7958-7966. PMID: 37813571
2 Genovese F, Bauersachs HG, Gräßer I, Kupke J, Magin L, Daiber P, Nakajima J, Möhrlen F, Messlinger K, Frings S. Possible role of calcitonin gene-related peptide in trigeminal modulation of glomerular microcircuits of the rodent olfactory bulb. Eur J Neurosci. 2017 Feb;45(4):587-600. PMID: 27891688.

**abstract citation id: bjaf055.018**

### Neural dynamics underlying odor perceptual learning in olfactory bulb and piriform cortex

*Kevin Bolding[1]*

*[1]Monell Chemical Senses Center. United States*

Animals continuously engage in perceptual learning as they explore their environment. In olfaction, this memory shapes future behavior, evidenced by reduced exploration of familiar odors. We presented head-fixed mice with repeated blocks of novel odors and observed continuous, odor-specific decreases in sniffing that persisted up to a week later, suggesting lasting, graded familiarity. PCx neural responses decoded odors experienced two hours earlier more accurately than novel odors, indicating a durable impact of passive exposure on odor processing.

We recorded from olfactory bulb (OB; 444 units) and piriform cortex (PCx; 1708 units) using Neuropixels 2.0 probes, including optotagged PV interneurons, and leveraged a larger database of waveforms and firing properties for cell-type identification. Trial-dependent neural changes included subtle reductions in early PCx response peaks (~80–100 ms post- inhalation) not consistently seen in OB. A robust, trial-dependent second-sniff response emerged in OB during early trials, while PCx showed sustained inter-sniff excitation. Secondary peaks in PCx shifted in amplitude and latency across trials, and individual neurons showed diverse response changes. OB neurons tended to increase firing with trial number, while excitatory PCx cells more often decreased. PV responses were mixed.

Inhibition in PCx might be expected to strengthen with experience, since familiar odors could drive faster and more synchronous ensemble activation that recruits internal inhibitory circuits. While PCx activity was more rapidly suppressed in later trials, PV responses largely mirrored excitatory ones. This suggests that suppression may arise from other interneurons or external sources, or reflect heterogeneity not visible in population averages. Divergent dynamics across OB, PCx, and PV populations support the idea that local circuit plasticity, not sniffing alone, underlies experience-dependent changes in early olfactory processing.

**Funding:** NIH/NIDCD: 5R01DC020927; CZI: 2024-338519

abstract citation id: bjaf055.019

### MARS SYMPOSIUM-ADVANCES IN UNDERSTANDING THE PERCEPTION OF KOKUMI SUBSTANCES

### *Symposium Organizer: Scott McGrane*

### Advances in understanding the perception of kokumi substances

*Scott McGrane*[1]

[1]*Mars Petcare - Waltham Petcare Science Institute. United Kingdom*

Kokumi has been an integral part of Asian cuisine for centuries and has only gained attention in Western foods more recently. Indeed, the word kokumi derives from the Japanese term for richness/ depth (koku) and taste/ flavour (mi). Kokumi substances in isolation are mostly described as tasteless, and have instead been shown to enhance sweet, salty, and umami tastes. Kokumi sensation is mediated by a homodimer of the Calcium Sensing Receptor (CaSR), which is a Class

C G-Protein Coupled Receptor (GPCR), consisting of a large extracellular N-terminus that is tethered by a cysteine-rich region to a seven-transmembrane domain. Kokumi includes a wide range of compounds, particularly amino acids and peptides, with one of the most well-known kokumi substances being glutathione. In recent years, the use of kokumi compounds by food manufacturers to create more nuanced and complex tastes for consumers has increased, and its potential in the food industry is significant. The aim of this symposium is to provide an update on recent advances in our understanding of the mechanisms and drivers of kokumi sensation.

abstract citation id: bjaf055.020

### Kokumi substances as taste modifiers: Sensory properties and Molecular mechanisms

*Seiji Kitajima*[1]

[1]*Ajinomoto Co., Inc. Japan*

The oral sensation perceived when food is eaten is a very complex experience. Much of our understanding of this sensation is derived from studies on the components that elicit diverse sensations, including taste, mouthfeel, and chemesthetic sensations. Difficulties are associated with defining and characterizing each of these sensations because they involve multiple chemosensory systems. Koku perception, which is originated from the Japanese word “koku”, is also a total sensation that is perceived through taste, smell, and texture. In this presentation, I will focus on kokumi substances, which are taste modifiers that have been increasingly reported in recent years and which impart and enhance taste-related koku perception, and will discuss their functions and mechanisms of action. Based on the background of our research conducted until now, I will talk about the complex sensory properties induced by kokumi substances and their molecular mechanisms of action obtained in our research, using γ-glutamyl kokumi peptides, which is the most intensively studied kokumi substance, as an example[1]. In particular, regarding the molecular mechanism of action, I will cover the mechanism of activation of the calcium-sensing receptor CaSR, which is assumed to be a major receptor for kokumi substances, and the mechanism of taste-modifying function of kokumi substances via the activation of CaSR[2,3]. Furthermore, I will discuss the possibility that various components of foods, such as volatile aroma components, may function as kokumi substances via activation of CaSR expressed on the tongue[4]. Our results and a more detailed scientific understanding of kokumi substances will contribute not only to improvements in food palatability, but also to the development of foods with less salt, sugar, and fat, thereby providing health benefits. It also may lead to the design of new foods that contribute to a sustainable society.

#### References

1 Kuroda, M. (2024). Kokumi Substance as an Enhancer of Koku: Its Definition. In Kokumi Substance as an Enhancer of Koku (pp. 15-22).

2 Yamaguchi, H., Kitajima, S., Suzuki, H., Suzuki, S., Nishikawa, K., Kamegawa, A., . . . Sugiki, M. (2025). Cryo-EM structure of the calcium-sensing receptor complexed with the kokumi substance gamma-glutamyl-valyl-glycine. Sci Rep, 15(1), 3894.

3 Kitajima, S., Maruyama, Y., Sasaki, K., Tajima, T., & Kuroda, M. (2022). Increases in the pungency of allyl isothiocyanate and piperine by CaSR agonists, glutathione and gamma-glutamyl-valyl-glycine. Physiol Behav, 256, 113952.

4 Kitajima, S., Maruyama, Y., & Kuroda, M. (2023). Volatile Short-Chain Aliphatic Aldehydes Act as Taste Modulators through the Orally Expressed Calcium-Sensing Receptor CaSR. Molecules, 28(12).

abstract citation id: bjaf055.021

### Involvement of multiple taste receptors in the actions of Kokumi taste stimuli

*Mee-Ra Rhyu*[1], *Yiseul Kim*[2], *Byung-Chang Suh*[3], *Dajeong Jeong*[3], *Albertino Bigiani*[4], *Vijay Lyall*[5]

[1]*Department of Food Science and Biotechnology, Sejong University, Republic of Korea*
[2]*Korea Food Research Institute, Republic of Korea*
[3]*Department of Brain Sciences, DGIST, Republic of Korea*
[4]*Department of Biomedical Sciences, University of Modena and Reggio Emilia. Italy*
[5]*Department of Cellular, Molecular, and Genetic Medicine, Virginia Commonwealth University. United States*

*Kokumi* taste stimuli are ligands that activate the calcium-sensing receptor (CaSR). *Kokumi* stimuli elicit flavor persistence and richness, and also modulate basic tastes, such as enhance salt taste. Most γ-glutamyl peptides produce *Kokumi* taste as natural allosteric modulators of

CaSR. We investigated the effects of γ-Glu-Cys-Gly (GSH) and γ-Glu-Val-Gly (γ-EVG) on salt taste using patch clamp technique and calcium signaling. Salt detection is mediated by at least two pathways. A $Na^+$ selective pathway that utilizes the amiloride (Am)-sensitive epithelial $Na^+$ channel (ENaC), and a cation non-selective pathway that is Am-insensitive. Patch-clamp studies using rat fungiform taste cells expressing ENaC provided direct evidence that GSH and γ-EVG do not alter ENaC activity. We further investigated if Kokumi taste substances can modulate salt response via the Am-insensitive pathway(s). We monitored temporal changes in $[Ca^{2+}]$ in HEK293T cells expressing the human vanilloid receptor 1 (hTRPV1), a non-selective cation channel, which has been suggested as a potential Am-insensitive salt taste mediator. GSH and γ-EVG induced concentration-dependent changes in $[Ca^{2+}]$ that were markedly attenuated in the presence of capsazepine, a specific TRPV1 antagonist. In cells expressing capsaicin-insensitive hTRPV1 mutants, the apparent affinity of hTRPV1 for GSH and γ-EVG was significantly reduced. These results suggest that multiple taste receptors may be potentially involved in the actions of *Kokumi* taste stimuli.

**Funding:** This work was supported in part by the National Research Foundation of Korea (NRF) [grant number NRF2020R1A2C2004661].

**abstract citation id: bjaf055.022**

### In silico framework for investigating the mechanics of kokumi taste: unveiling the chemistry to discover new kokumi compounds

*Florinda Perugino[1,2], Lorenzo Pedroni[1], Francesco Ferri[3], Chiara Dall'Asta[1], Fulvio Mattivi[4], Antonella Di Pizio[3,5], Gianni Galaverna[1], Luca Dellafiora[1]*

[1]*Department of Food and Drug, University of Parma, Parma, Italy*
[2]*Department of Biology, University of Naples Federico II, Naples, Italy*
[3]*Leibniz Institute for Food Systems Biology at the Technical University of Munich, Freising, Germany*
[4]*Research and Innovation Centre, Fondazione Edmund Mach, San Michele all'Adige, Italy*
[5]*Professorship for Chemoinformatics and Protein Modelling, Technical University of Munich, Freising, Germany*

The relevance of kokumi sensation for taste, flavor, mouthfeel, and aftertaste quality of a wide variety of foods has driven the increasing interest in characterization, identification, the study of new kokumi-active molecules [1]. From a mechanistic standpoint, the potential of a compound to elicit kokumi sensations is related to its ability to bind the Venus flytrap (VFT) domain of the Calcium Sensing Receptor (CaSR), resulting in its allosteric activation, triggering the signaling cascade that culminates in the taste perception [2].

The release of 3D structures over the last ten years made possible the application of 3D molecular modelling techniques enabling the fine understanding of CaSR activation and chemical underpinning of kokumi substances from a precise molecular standpoint.

By applying a 3D molecular modelling approach comprising coarse-grained virtual screening subsequently refined by docking and molecular dynamics simulations, our work demonstrated that key ligand-dependent CaSR conformational changes, prodromic to taste elicitation, can predict its activation while elucidating the underlying chemical determinants of ligands.

Besides gaining a deep molecular understanding of CaSR mechanics, the approach enabled the wide screening of compounds either to characterize the kokumi note of foods (by identifying the responsible chemical entities) or to discover novel kokumi compounds.

An additional implementation shall include Machine Learning model trained on docking score and Morgan fingerprints, adapted from a model built for the prediction of ligands for GPR120 (important for fatty taste perception), to empower predicting performances and throughput.

### References

1 Li, Q., Zhang, L., & Lametsch, R. Current progress in kokumi-active peptides, evaluation and preparation methods: a review. Critical Reviews in Food Science and Nutrition, 2020, 62 (5), 1230–1241.
2 Guha, S. & Majumder, K. Comprehensive Review of γ-Glutamyl Peptides (γ-GPs) and Their Effect on Inflammation Concerning Cardiovascular Health. Journal of Agricultural and Food Chemistry 2022, 70 (26), 7851-7870.

**abstract citation id: bjaf055.023**

### Exploring the mechanisms and role of kokumi taste for the domestic cat (*Felis catus*)

*Anni Laffitte[1], Matthew Gibbs[1], Carlos Hernangomez de Alvaro[1], James Addison[1], Zoe Lonsdale[1], Maria Giribaldi[2], Andrea Rossignoli[2], Timo Vennegeerts[2], Marcel Winnig[2], Boris Klebansky[3], Jerry Skiles[3], Darren Logan[1], Scott McGrane[1]*

[1]*WALTHAM Petcare Science Institute, Freeby Lane, Waltham on the Wolds, Melton Mowbray, Leicestershire, LE14 4RT. United Kingdom*
[2]*AXXAM S.p,A, OpenZone, Via Meucci 3, Bresso, MI 20091. Italy*
[3]*BioPredict, Inc., 4 Adele Avenue, Demarest, NJ 07627. United States*

The domestic cat (Felis catus) is an obligate carnivore with a diet abundant in proteins and other meat-derived compounds. Meat is rich in known umami and kokumi-tasting compounds such as amino acids and peptides, and for carnivores it is suggested that these taste modalities drive palatability of food more than other taste-modalities. Kokumi is a well-known taste modality, described as an enhancement of sweet, salty, and umami tastes and the detection of kokumi compounds has been widely attributed to the Calcium Sensing Receptor (CaSR). We hypothesised that the CaSR is also a kokumi receptor for the domestic cat, and that this receptor plays a key role in their taste perception, due to its known role in the detection of different peptides and amino acids. Using *in silico*, *in vitro* and *in vivo* approaches, we compared the cat and human kokumi receptors and taste perception. From our *in silico* modelling using molecular docking, we concluded that the different identified ligand-binding sites for the cat and human CaSR only differ from each other by one amino acid residue, meaning a high probability of similar binding properties. Following this, we then used an *in vitro* cellular model of the cat CaSR to screen 159 compounds for agonist activity and had a hit rate of 40%. We then compared the cat cellular CaSR model to the corresponding human model and found that, all cat CaSR ligands were also ligands of human receptor and the ligand binding affinities between the two were near identical. Using palatability tests with cats and sensory panel tests with humans, we demonstrated that cats show kokumi-like perception with CaCl2 ($p=0.0039$), but no significant effect was identified for Glutathione ($p=0.0630$), whereas for the human panel the reverse is true ($p>0.05$ for CaCl2 and $p<0.05$ for Glutathione).

Our data suggest that while kokumi is an important taste modality for carnivores, key differences persist in the perception of kokumi taste between different species.

**Funding:** All the work presented was funded by Mars Petcare UK.

**abstract citation id: bjaf055.024**

### BIOLOGICALLY INSPIRED AND BIO-BASED SENSORS IN OLFACTION AND TASTE

### *Symposium Organizer: Jérémie Topin*

### Discriminating Ligands via the Capacitive Response of hOR1A1

*Anna Lagunas[1], Christine Belloir[2], Maxence Lalis[3], Loïc Briand[2], Jérémie Topin[3], Pau Gorostiza[1], Josep Samitier[1]*

*[1]Institute for Bioengineering of Catalonia (IBEC). Spain*
*[2]Centre des Sciences Du Goût et de L'Alimentation, CNRS, INRAE, Institut Agro, Université de Bourgogne,. France*
*[3]Institut de Chimie de Nice, Université Côte D'Azur, Campus Sciences. France*

Olfactory receptors (ORs) are class A (rhodopsin-like) G protein-coupled receptors (GPCRs)[1] found primarily in olfactory sensory neurons (OSNs) in the nasal epithelium. Each OSN expresses a single type of OR, which once bound to an odorant ligand undergoes a conformational change that activates cell signaling and neuronal firing. An OR has variable affinity for a subset of odorants and a single odorant can activate multiple receptors with different affinities. The combinatorial effect of this variable regulation produces the sense of olfaction[2]. The human olfactory system can discriminate between thousands of volatile compounds, including those of similar molecular structures[3].

The sensitivity and selectivity of odorant recognition by ORs has led to the development of biohybrid sensors for the detection of volatile organic compounds, reporting concentration levels of up to femtomolar. However, although physiological discrimination of odorants relies on the selectivity of ORs, given by their tertiary structure, current OR-based biohybrid sensors typically fail to distinguish between the agonists of the same receptor[4].

In this work, we present a method for odorant discrimination that exploits the modulation of the capacitive response of the human OR hOR1A1. Using electrochemical impedance spectroscopy and voltammetry, we analyzed the electrical response of hOR1A1 immobilized on a gold electrode in the presence of three known high-affinity agonists. Ligand binding induced a measurable, ligand-dependent decrease in capacitive signal, reaching up to 40% for the cognate ligand dihydrojasmone. This response is attributed to binding-induced alterations in the receptor's electric dipole moment, which modulates its interaction with the external electric field. Our findings demonstrate a strategy for distinguishing between structurally related high-affinity odorants and provide a foundation for the design of highly selective OR-based biosensors[5].

**Funding:** This work was supported by the Biomedical Research Networking Center (CIBER), Spain. CIBER is an initiative funded by the VI National R&D&i Plan 2008–2011, Iniciativa Ingenio 2010, Consolider Program, CIBER Actions, and the Instituto de Salud Carlos III, with the support of the European Regional Development Fund (ERDF). This work was fun ded by the CERCA Program and by the Commission for Universities and Research of the Department of Innovation, Universities, and Enterprise of the Generalitat de Catalunya (2021 SGR 01545, 2021 SGR 01410). This work was also partly supported by grants from the Conseil R´egional Bourgogne, Franche- Comte (PARI grant), the FEDER (European Funding for Regional Economic Development), and by the Agence Nationale de la Recherche (ANR). M.L. was supported by the Fondation Roudnitska under the aegis of Fondation de France and by GIRACT (Geneva, Switzerland).

### References

1 Buck, L., Axel, R., 1991. Cell 65, 175–187. https://doi.org/10.1016/0092-8674(91) 90418-X
2 Saito, H., Chi, Q., Zhuang, H., Matsunami, H., Mainland, J.D., 2009. Sci. Signal. 2, ra9. https://doi.org/10.1126/SCISIGNAL.2000016.
3 Feng, G., Zhou, W., 2019. Elife 8, e41296. https://doi.org/10.7554/ELIFE.41296
4 Wasilewski, T., Brito, A.F., Szulczy Nski C, B., Wojciechowski, M., Buda, N., Claudia, A., Melo, A., Kamysz, W., Gebicki, J., 2022. TrAC, Trends Anal. Chem. 150, 116599. https://doi.org/10.1016/j.trac.2022.116599.
5 Lagunas, A., Belloir, C., Lalis, M., Briand, L., Topin, J., Gorostiza, P., Samitier, J., 2025. Biosens. Bioelectron. 271, 117000. https://doi.org/10.1016/j.bios.2024.117000

abstract citation id: bjaf055.025

### Olfactory receptors for charge-sensitive sensors

*Christophe Moreau*[1]

[1]*Institute of Structural Biology, Grenoble. France*

The development of highly sensitive, specific and versatile electronic noses would offer promising perspectives for the detection of volatile organic compounds in fields such as healthcare (e.g. non-invasive in vitro diagnostics), environmental monitoring (e.g. detection of contaminants), ligand screening (e.g. repellents or olfactory traps) and many others. One of the major technological challenges lies in integrating relevant recognition elements onto transducers to specifically detect molecules of interest without being affected by confounding factors. Olfactory receptors are recognition elements that have naturally evolved to efficiently detect volatile molecules in complex environments. Mimicking this system in bio-electronic noses is an approach being explored by several international laboratories, by interfacing biological probes with suitable transducers. Field-effect transistors (FETs) are particularly well-suited to the detection of charge movement, and can therefore be interfaced with ion channels through which ions transit in response to a stimulus.

Our team has expertise in structure-function studies of G protein-coupled receptors (GPCRs) and ion channels using protein engineering and automated electrophysiological techniques. We have developed fusion proteins composed of GPCRs and ion channels able to generate an electrical signal in response to GPCR ligands. The fusion of olfactory GPCRs with ion channels has been explored with the laboratories of Prof. Tai Hyun Park and Prof. Seunghun Hong (Seoul National University), and more recently we have been focusing on insect olfactory receptors (iORs), which have the particularity of forming ion channels. These iORs are composed of two subunits: a highly conserved olfactory receptor co-receptor (Orco) subunit that spontaneously associates with a huge diversity of olfactory receptors (ORs) that specifically recognize ligands. We are exploring the potential of these probes for future integration into FETs.

**Funding:** European Research Council (ERC) under the European Union's Horizon 2020 programme (grant agreement no. 682286; Agence Nationale de la Recherche (ANR) (ANR-23-CE09-0010-03)

abstract citation id: bjaf055.026

### From molecular binding to conformational change, an extensive study on Olfactory Binding Protein (OBPs)

*Maxence Lalis*[1]

[1]*Université Côte d'Azur. France*

OBPs (Olfactory Binding Proteins) are a promising lead for developing electronic noses, making it essential to understand their mechanisms.

The chemical spaces for OBP binders are vast. Random identification of the chemical binding space is a delicate task. Molecular docking techniques have been employed to reduce the chemical space, complementing isothermal titration calorimetry (ITC) experiments. This targeted approach improves research efficiency and provides a focused framework for further in vitro assays.

In addition, point mutations affect the binding and recognition behavior of OBP. We have identified the key amino acids responsible for binding in rat OBP1. Molecular dynamics (MD) simulations revealed a conserved binding pathway for a variety of ligands, making it possible to modify OBP behavior through protein engineering strategies. For example, OBP activity was selectively abolished, paving the way for robust control in binding assays.

We identified other key amino acids responsible for the selectivity of rat OBP3. MM-GBSA analysis was used to assess the impact of point mutations on OBP binding specificity, providing valuable information for future protein engineering attempts. Finally, we explored and proposed a rationale for the conformational change induced upon binding of molecules to rOBP3.

Local conformational changes were observed in MD simulations and validated by circular dichroism.

The integrated computational-experimental approach not only improves the efficiency of in vitro assays, but also offers a comprehensive understanding of protein behavior and binding dynamics.

abstract citation id: bjaf055.027

### Bioinspired electronic nose for artificial olfaction development

*Yanxia Hou*[1]

[1]*CNRS. France*

In the current era, the advancement of artificial olfaction poses significant scientific and technological challenges. In such a context, bioinspired sensor technology, including bioelectronic noses, shows great promise. These systems utilise biological materials as sensing elements, including olfactory receptors (ORs) and odorant binding proteins (OBPs). They boast significant advantages in terms of high sensitivity and selectivity. However, they are limited by stability and robustness. We have developed innovative bioelectronic noses using more stable biomolecules, such as peptides. Both cross-reactive and selective peptides have been designed and incorporated in a chip with microarray format. Our bioelectronic nose is equipped with an advanced optical detection system known as surface plasmon resonance imaging (SPRi), enabling it to detect odours in a label-free and real-time manner, providing valuable kinetic information. The device is highly versatile for the analysis of large chemical families of odorants, offering good sensitivity, selectivity and stability. Our technology has been used to develop portable devices by a local company, which show promise in terms of on-field applications.

### References

1 M. El Kazzy, M. Lalis, C. Hurot, J. S. Weerakkody, R. Mathey, A. Buhot, T. Livache, J. Topin, L. Moitrier, C. Belloir, L. Briand, Y. Hou,* "Study and Optimization of the Selectivity of an Odorant Binding Protein-based Bioelectronic Nose", Biosensors and Bioelectronics, 2025, 268, 116879. (DOI: /10.1016/j.bios.2024.116879)
2 J. S. Weerakkody, M. El Kazzy, E. Jacquier, P. H. Elchinger, R. Mathey, W. L. Ling, C. Herrier, T. Livache, A. Buhot, Yanxia Hou, * "A Surfactant-like Peptide Self-assembled into a Biohybrid Multi-sensor Array for an Electronic Nose", ACS Nano, 2022, 16, 3, 4444–4457 (DOI: 10.1021/acsnano.1c10734).
3 S. Brenet, J. S. Weerakoddy, A. Buhot, F. X. Gallat, R. Mathey, L. Leroy, T. Livache, C. Herrier, Y. Hou*, "Improvement of Sensitivity of Surface Plasmon Resonance Imaging for the Gas-Phase Detection of Volatile Organic Compounds", Talanta, 2020, 212, 120777. (DOI: 10.1016/j.talanta.2020.120777)
4 S. Gaggiotti, C. Hurot, J. S. Weerakkody, R. Mathey, A. Buhot, M. Mascini, Y. Hou*, D. Compagnone*, "Development of an optoelectronic nose based on surface plasmon resonance imaging with peptide and hairpin DNA for sensing volatile organic compounds", Sensors and Actuators: B. Chemical, 2020, 303, 127188. (DOI: 10.1016/j.snb.2019.127188)
5 S. Brenet, A. John-Herpin, F. X. Gallat, B. Musnier, A. Buhot, C. Herrier, T. Rousselle, T. Livache, Y. Hou*, "Highly-Selective Optoelectronic Nose Based on Surface Plasmon Resonance Imaging for Sensing Volatile Organic Compounds", Analytical Chemistry, 2018, 90 (16), 9879 -9887. (DOI: 10.1021/acs.analchem.8b02036)

**abstract citation id: bjaf055.028**

### HUMAN OLFACTORY-DERIVED IN VITRO MODELS FOR TRANSLATIONAL RESEARCH

### *Symposium Organizer: Maria Figueres-Oñate*

### Translational potential of olfactory-derived models and their applications across various biomedical fields

*Maria Figueres-Oñate*[1]

[1]*Instituto de Salud Carlos III. Spain*

Recent advances in human olfactory-derived in vitro models have opened new avenues for studying neurological disorders, tumor biology, and environmental impacts on brain health. This symposium will explore how patient-derived cellular models, including organoids and primary cultures from the olfactory system, are transforming biomedical research by providing physiologically relevant platforms for disease modeling and therapeutic discovery.

Dr. Leyre Urigüen (University of the Basque Country, Spain) will discuss the role of the olfactory system in neuropsychiatric disorders, highlighting how in vitro models contribute to understanding molecular mechanisms underlying conditions such as schizophrenia and depression.

Dr. Katja Kanninen (University of Eastern Finland, Finland) will present her work on the impact of environmental factors and air pollution on brain health, using patient-derived olfactory cells as a novel model for studying neurotoxicity and neurodegenerative disease susceptibility.

Dr. Bradley Goldstein (Duke University, USA) will showcase his research on patient-derived tumor organoid models from olfactory-related structures, providing insights into the molecular mechanisms of rare tumors affecting the olfactory system and their potential implications for targeted therapies.

The symposium, moderated by Dr. María Figueres-Oñate (Instituto de Salud Carlos III, Madrid, Spain), will promote discussions on the translational potential of olfactory-derived models and their applications across various biomedical fields. By bringing together experts working at the intersection of olfactory research and disease modeling, this symposium aims to highlight the broad applicability of human-derived olfactory models in translational biomedical research.

**abstract citation id: bjaf055.029**

### Impact of air pollution exposure in olfactory mucosal cells in Alzheimer's disease

*Katja Kanninen*[1]

[1]*University of Eastern Finland. Finland*

Air pollution ultrafine particles (UFPs) are an emerging concern for brain health that are implicated in neurodegeneration, including Alzheimer's disease (AD). Mounting evidence suggests that pollutant particles can affect the brain through the olfactory tract, however, the exact cellular mechanisms of how the cells of the olfactory mucosa (OM) respond to air pollutants remains poorly known. This presentation describes the observed alterations in biopsy-derived OM cells of individuals with AD compared to cells derived from cognitively healthy control subjects. It also describes how exposure to air pollution particles, especially the UFPs, affects human OM cells on transcriptional and functional levels.

**abstract citation id: bjaf055.030**

### Patient-Derived Olfactory Tumor Models: Insights into Disease Mechanisms and Therapeutic Approaches

*Bradley Goldstein*[1]

[1]*Duke University. United States*

The peripheral olfactory system is capable of self-renewal throughout life in mammals, producing new neurons and barrier epithelial cells from resident stem cells. While in vivo models have been used widely for dissecting neurogenesis, culture systems have been surprisingly challenging to develop. We review here efforts to cultivate basal stem and progenitor cells, as well as supporting populations, including olfactory ensheathing glia, from rodent and human. Using 3D culture approaches, we discuss recent efforts to cultivate human olfactory ensheathing glia tumor cells. Culture models can be leveraged to help investigate growth properties, such as defining ensheathing glia secretome. Efforts to development additional novel culture models are also discussed, as well as future directions.

**abstract citation id: bjaf055.031**

### Isolation and Differentiation of Neurons and Glial Cells from Olfactory Epithelium in Living Subjects: Advancing Neuropsychiatric and Neurodegenerative Disease Research

*Leyre Urigüen Echeverria*[1]

[1]*University of The Basque Country UPV-EHU. Spain*

The olfactory epithelium (OE) offers a uniquely accessible source of neural cells from living individuals, providing an invaluable model to investigate human brain pathophysiology through a minimally invasive approach. This work presents advanced methodologies for the isolation and differentiation of neurons and glial cells from OE, emphasizing their application in neuropsychiatric and neurodegenerative disease research. Unlike traditional models relying on post-mortem brain tissue or induced pluripotent stem cells (iPSCs), OE-derived cells preserve native phenotypic characteristics and patient-specific molecular signatures, enabling more accurate modeling of disease processes.

The approach integrates transcriptomic assays, including bulk and single-cell RNA sequencing, to dissect the cellular heterogeneity and dynamic gene expression profiles within neuronal and glial populations. Furthermore, extracellular vesicles (EVs) secreted by OE-derived cells into the culture medium are characterized as emerging biomarkers and mediators of intercellular communication, offering insights into cellular health and pathological changes.

Combining molecular profiling with functional studies and advanced culture techniques, these OE-derived models recapitulate key features of neurodevelopment and neurodegeneration. This platform supports longitudinal investigation of disease progression and treatment responses in patient-derived cells, facilitating personalized therapeutic strategies.

Overall, isolating and differentiating neurons and glial cells from the olfactory epithelium, coupled with transcriptomic and extracellular vesicle analyses, provides a powerful framework to advance understanding of cellular mechanisms underlying neuropsychiatric and neurodegenerative disorders, opening new avenues for biomarker discovery, drug screening, and targeted therapies.

### References

1 Unzueta-Larrinaga P, Barrena-Barbadillo R, Ibarra-Lecue I, Horrillo I, Villate A, Recio M, Meana JJ, Diez-Alarcia R, Mentxaka O, Segarra R, Etxebarria N, Callado LF, Urigüen L. Isolation and Differentiation of Neurons and Glial Cells from Olfactory Epithelium in Living Subjects. Mol Neurobiol. 2023 Aug;60(8):4472-4487. doi: 10.1007/s12035-023-03363-2. Epub 2023 Apr 28. PMID: 37118325;

2 Paula Unzueta-Larrinaga, Esteban Cuesta-Vega, Rocío Barrena-Barbadillo et al. Extracellular Matrix Dysfunction and Synaptic Dysfunction in Schizophrenia, 2025, Molecular Psychiatry. In press

**abstract citation id: bjaf055.032**

### OPEN-ACCESS TOOLS AND INNOVATION FOR OLFACTORY EXPERIMENTATION

### *Symposium Organizers: Chloé Guillaume; Kevin Bolding*

### Olfactometry, not rocket science: simple, inexpensive designs for flexible, high-fidelity odor delivery supporting neuroscientific and perceptual studies

*Matt Wachowiak*[1]

[1]*University of Utah. United States*

A novel design principle for odorant generation and delivery will be discussed. The design addresses a number of hurdles to flexible and well-controlled odorant delivery in an experimental setting: it minimizes contamination between odorant channels, allows for flexible (i.e., on-the-fly) generation of odorant mixtures, and eliminates or minimizes the change in airflow to the subject when odorant is presented. A key aspect of the design is a novel, venturi-based approach to achieve free-space mixing of odorant vapor in a carrier stream and a high degree of dilution, with no dead volume. We have developed two variants of this design specialized for different applications; the ‘Odor Gun’ involves the ejection of odorant vapor from disposable reservoirs into a central carrier stream, and is ideal for screening many odorants flexibly and rapidly. The ‘Turbulator’ is designed to accommodate 8 dedicated channels arising from larger odorant reservoirs and to achieve high dilutions of odorant. It is ideal for repeated presentations over many trials, for flexibly generating odorant mixtures and for delivering odorant with no additional cue (e.g., valve click or airflow/pressure change) - useful for behavior. Designs and parts lists are freely-shared and have been adopted for use by a number of research labs.

**abstract citation id: bjaf055.033**

### Visualizing and sonifying neurodata (ViSoND): closing the loop between observation and computation

*Matt Smear*[1]

[1]*University of Oregon. United States*

Recent advances now let us record from thousands of neurons in freely moving animals while tracking their behavior with sub-millimeter-scale precision. These massive, high-dimensional datasets promise new biological insights but pose equally large interpretive challenges: statistical analyses often expose patterns that are hard to map back onto real behavior. To bridge this gap, we created ViSoND — Visualization and Sonification of NeuroData — an open framework that synchronizes behavior video with a musical rendering of neural activity. Continuing a tradition that dates to Lord Adrian, we assign each neuron a distinct MIDI pitch, letting listeners "hear" spikes while they "see" the animal's actions. Pitch does for spike trains what false color does for anatomy: it gives every unit a unique, intuitive identity.

We illustrate ViSoND's utility in two settings. Respiration & olfactory bulb: automated analyses uncovered an unexpected slow rhythm in mouse sniffing; ViSoND immediately revealed that this rhythm marks discrete behavioral states. Eye movements & visual cortex: statistics showed a gaze-locked firing sequence across neurons; ViSoND exposed the same sequence during eye blinks, an insight missed by computation alone.

Beyond discovery, ViSoND makes neural data tangible for students, collaborators, and lay audiences, promoting more inclusive communication of neuroscience.

**abstract citation id: bjaf055.034**

### Reinforcement Learning and Fluid Dynamics Approaches to Olfactory Navigation and Swarming of Copepods in Turbulence

*Martin James[1], Francesco Viola[2], Agnese Seminara[1]*

*[1]Machine Learning Genoa Center (MaLGa), University of Genoa. Italy*
*[2]Gran Sasso Science Institute. Italy*

The survival of aquatic organisms is intricately linked to their ability to mask odor signals and form collectives. However, despite this critical ecological significance, how the swimming of organisms such as copepods moving at small Reynolds numbers affects odor dispersion and how they come together into swarms remains poorly understood. In the first part, we use direct numerical simulations to explore the coupling of the hydrodynamic fluctuations introduced by the organisms with their odor dispersion. We consider a school of small aquatic swimmers (Reynolds number $\leq 50$) immersed in a turbulent open channel flow and vary their Reynolds numbers, evaluating the consequential changes in their odor field. We show that the velocity fluctuations due to the swimmers play a significant role in changing the range and distribution of odor signals by screening the intensity and fluctuations of the odor field. Furthermore, there are substantial differences in screening depending on the swimming modes of the organisms - pushers vs pullers - with pullers being more effective at screening their odor from predators in some environments. In the second part, we use reinforcement learning to study how small swimmers use sparse odor signals to come together to form swarms despite turbulent dispersion. Our findings provide valuable insights into odor masking by aquatic organisms and their swarming in turbulence, with novel considerations regarding the evolutionary preferences of specific swimming modes.

### Reference

1 Martin James, Francesco Viola and Agnese Seminara, 'Swimming mode determines how well mesoscale swimmers shield their odor in turbulence' arXiv:2501.00789 (2025)

**abstract citation id: bjaf055.035**

### Accessible and automated behavioural testing in freely moving mice: open-source tools for multisensory conditioning and odour- driven behaviour

*Chloé Guillaume[1], Harin Wijayathunga[1], Connor Doyle[1], Jieni Wang[1], Yash Agarwal[1], Poort Jasper[1], Elisa Galliano[1]*

*[1]University of Cambridge. United Kingdom*

Advancing behavioural testing methods is essential for progress in systems neuroscience. While several custom-built setups have improved standardization and automation for freely moving mice, replicating these systems remains a technical and/or financial barrier for many laboratories.

This presentation introduces two open-science tools for rodent behavioural testing that prioritize affordability, modularity, and ease of replication. The first is a fully automated system inspired by the AutonoMouse platform, developed by the Schaefer Laboratory. Our re-engineered version emphasizes the use of low-cost, widely available components and open- source software. Moreover, it supports operant conditioning across multiple sensory modalities (olfaction, vision, audition). The system includes a durable cage for up to 20 mice, a corridor equipped with an RFID reader and scale for individual identification and weight monitoring; a test chamber featuring a lickometer for liquid reward delivery, and for stimulus presentation a speaker, a touchscreen, and a custom-built 16-channel olfactometer (expandable), offering precise odour delivery.

The second tool, connected to an identical olfactometer, consists of four infrared transmitting plexiglass arenas, each with an odour port and exhaust. It features top and side cameras in night mode to monitor behaviour during odour-based tasks like discrimination, habituation, and attraction/avoidance.

Both systems allow full customization of key parameters, including stimulus timing, and trial structure, thanks to a graphical interface, Python and Arduino code. Integrated video tracking is compatible with DeepLabCut for automated pose estimation and movement analysis.

These tools reflect open science principles by being freely shared, modifiable, and scalable, lowering barriers to behavioural experimentation and promoting broader participation, reproducibility, and innovation in neuroscience.

abstract citation id: bjaf055.036

### SCENT AND SEXUALITY: EXPLORING THE INTERSECTION OF SMELL, RELATIONSHIPS, AND HEALTH

### *Symposium Organizers: Marlise Hofer; Jessica Gaby*

### The impact of COVID-19 related smell dysfunction on sexual and mental wellbeing: data from a longitudinal sample

*Marlise Hofer*[1]

[1]*University of Victoria, Canada*

Olfactory disorders are associated with poor sexual and mental wellbeing, but most existing research relies on retrospective or cross-sectional designs. This study assessed these associations prospectively in a sample of 73 patients with COVID-19–related olfactory dysfunction, recruited between 2021 and 2023 at a Smell Clinic in Dresden, Germany. Patients were assessed twice, approximately six months apart. At each time point, olfactory functioning was measured using both psychophysical tests and self-rated assessments, alongside measures of sexual and mental wellbeing, including sexual frequency, sexual desire, general wellbeing, and symptoms of anxiety and depression. Within-person improvements in psychophysical olfactory function were linked to increased sexual frequency. Among a subset of 51 participants in stable romantic partnerships, improvements in olfactory function also predicted increases in both sexual frequency and sexual desire. However, no significant associations were found between changes in olfactory function and changes in mental health or general wellbeing. These findings suggest that declines in sexual functioning may be an early psychosocial consequence of olfactory dysfunction, highlighting the potential benefit of incorporating sexual health support into care for individuals with recent-onset smell disorders.

abstract citation id: bjaf055.037

### There’s chemistry between us: romantic partner’s smell and relationship quality

*Egenaz Kiraz*[1], *Johan C.T.M Karremans*[2], *Ruddy Faure*[2], *Jasper H.B. de Groot*[2]

[1]*Radboud University. The Netherlands*
[2]*Radboud University (Behavioural Science Institute). The Netherlands*

Body odors have a nonverbal communication function and convey important cues about the emotions of others, including those of our romantic partners. Given that nonverbal communication and emotional understanding are crucial for romantic relationships, smell may foster connection and attachment in these contexts. To elaborate on this topic, I will present a survey study where we explored how a romantic partner’s odor influenced explicit and implicit indicators of relationship quality. Specifically, we investigated if individuals’ evaluation of their partner’s body odor was associated with relationship satisfaction, emotional intimacy, and sexual intimacy; as well as with implicit partner evaluations. Our results highlight the importance of perceiving a partner’s odor as pleasant for sexual intimacy, controlling for other partner traits. Additionally, our exploratory findings offer preliminary evidence that finding a partner’s smell pleasant predicts increased safety and sexual arousal during exposure to the odor, which in turn are associated with more emotional and sexual intimacy, respectively. In line with these findings, I will discuss how body odors contribute to romantic chemistry and provide directions for future research.

abstract citation id: bjaf055.038

### A survey-based look at the effects of hormone therapy on body odor production and perception

*Jessica Gaby*[1]

[1]*Middle Tennessee State University. United States*

Though anecdotal reports of body odor changes following the initiation of hormone therapy are common, there is a dearth of medical or sensory literature characterizing what body odor changes one might expect when beginning hormone treatment. As such, individuals who are receiving hormone therapy (HT) are often eliminated from body odor research. In this talk I will discuss results from an ongoing self-report survey investigating changes in body odor production and perception among individuals who are receiving HT. 33 individuals have completed the survey thus far. The majority of participants (20/33) reported noticing body odor changes after beginning HT. Specifically, those receiving testosterone (T) reported their body odor becoming stronger and more noticeable to themselves and others. Several reported being complimented on their body odor more often following the initiation of hormone therapy. Those receiving estrogen, estradiol, progesterone, and/or spironolactone (EEPS) reported their body odor becoming sweeter and less intense. On average, those receiving T generally noticed changes within one month, while those receiving EEPS reported noticing these changes within the first 6 weeks. One notable finding is that the majority of participants reported liking their body odor more after HT, even when they experienced increases in sweating and/or body odor intensity, suggesting that these changes are viewed as affirming rather than upsetting. This effect was driven largely by those receiving T, who noticed body odor changes more frequently. These results provide insight into previously unreported social olfactory changes in those receiving hormone therapy, and suggest methodological steps for incorporating those on HT into mainstream body odor research.

**abstract citation id: bjaf055.039**

### Smelling the romantic partner's body odor increases psychological and autonomic but not cortisol stress responses

*Franny Spengler[1], Johannes T Doerflinger[1], Josephine A Noel[1], Beate Ditzen[2], Jessica Freiherr[3], Markus Heinrichs[1]*

*[1]Laboratory for Biological Psychology, Clinical Psychology, and Psychotherapy, Department of Psychology, University of Freiburg, Freiburg, Germany*
*[2]Heidelberg University Hospital, Institute of Medical Psychology, Bergheimer Str. 20, 69115 Heidelberg, Germany*
*[3]Sensory Analytics & Technologies, Fraunhofer Institute for Process Engineering and Packaging IVV, Giggenhauser Straße 35, 85354 Freising, Germany*

While social support from romantic partners is known to attenuate stress responses, it remains unclear whether perceiving a partner's body odor can elicit similar stress-buffering effects. In this study, 179 participants living in heterosexual romantic relationships underwent either the Trier Social Stress Test (TSST) or a non-stressful control condition while being exposed to their partner's body odor (collected under standardized conditions over five consecutive nights) or a neutral, non-social control odor presented via an olfactometer. The partner's odor had no effect on cortisol release. However, contrary to previous findings, subconsciously smelling one's own partner increased subjective stress and heart rates. Potential underlying mechanisms include the causal misattribution of attraction-related, arousal-induced heart rate increases on the stressful experimental situation, or an evolutionary adaptive mechanism that amplifies stress responses when a loved one is potentially involved in the threatening situation.
**Funding:** This research was funded by the University of Freiburg.

**abstract citation id: bjaf055.040**

### THE MEXICAN NETWORK OF OLFACTION: A COLLABORATIVE HUB FOR CUTTING-EDGE RESEARCH AND INNOVATION IN OLFACTORY SCIENCES

### *Symposium Organizer: Rebeca Corona*

### Presentation of the Mexican network of olfaction

*Rebeca Corona[1]*

*[1]Instituto de Neurobiología, Universidad Nacional Autónoma de México. Mexico*

Olfaction is the most conserved chemical sense and is essential for survival and welfare. This system is one of the most sensitive, showing early signs of deterioration under certain conditions, making it a predictor of the onset of various diseases. For many years, olfaction has been a topic of great research interest in Mexico. In this talk, we will introduce the Mexican Network of Olfaction, a collaborative initiative that brings together groups in Mexico that have developed sophisticated and innovative lines of research that contribute to the global understanding of olfaction, from basic research to technological, agronomic, and clinical applications, as well as the impact of olfaction on daily life. The network members study olfaction using various models, techniques, and research questions. Research on chemical communication in insects, through pheromones, will teach us that this communication can be of great benefit for medical and agronomic applications with high economic impact. We will discuss studies of olfactory function from a physiological perspective in innate behaviors that determine survival, as well as the plasticity and functional connectivity phenomena that accompany reproductive and olfactory-motivated behaviors. Animal models where olfactory function reflects pathological and inflammation conditions will be addressed and translational studies where the sense of smell is compromised in certain hormonal conditions and by the quality of the air we breathe and can be used as a biomarker of neurodegenerative and metabolic diseases. This network seeks to promote connections between researchers and institutions interested in the study of smell and to create a specialized discussion forum that fosters and facilitates collaboration and the advancement of knowledge, highlighting the importance of smell.
**Funding:** UNAM-DGAPA-PAPIIT IN214822 and IN220025.

**abstract citation id: bjaf055.041**

### Experimental development of olfactory system in Mexico

*Verónica Viñuela Berni[1]*

*[1]1Instituto de Neurobiología, 2Departamento de Fisiología, Facultad de Medicina, Universidad Nacional Autónoma de México. Mexico*

In this presentation, we will discuss the contributions of three of the early Mexican researchers who initiated the study of the nervous system's role in olfaction. Studies of the olfactory system in Mexico began in the fifties in the Faculty of Medicine at UNAM with professor Dr. Hernández Peón. He focused on the afferent-transmission function to explain the fundamental mechanisms of learning and habituation. In a series of experiments, he reported that if an individual is continuously exposed to a particular odor, they cease to perceive it, indicating a phenomenon of habituation and/ or learning. Dr. Alcocer Cuarón, a friend of Dr. Hernández-Peón, was invited by him to collaborate on a project focusing on learning processes. Dr. Alcocer's group began studying electrical discharges recorded in the frog's epithelium (spindles). By using an olfactory electroencephalogram, Dr. Alcocer's question whether the rhythmic activity recorded in the epithelium in response to an olfactory stimulus correlates with the respiratory rhythm. They demonstrated that an olfactory signal is required, that is, the air must contain odorant particles that activate the olfactory epithelium and are not dependent on the mechanical excitation of the inhaled air. Couple of decades later, Dr. Álvarez-Buylla, made significant contributions studying the plasticity of the olfactory system during adulthood in mammals. He reported that in in vitro experiments, precursor cells of neurons located in the walls of the ventricles in adult mouse brains, when genetically marked and transplanted, could migrate to the olfactory bulb and differentiate into neurons. He proposed that these could be GABAergic inhibitory neurons located in different layers of the olfactory bulb structure, playing a crucial role in olfactory transmission by inhibiting mitral cells in the olfactory bulb. The contributions made by these researchers marked the set point beginning the school of studies in olfaction in Mexico.

**abstract citation id: bjaf055.042**

### Olfactory system: the remarkable contributions of a Mexican neuroanatomist

*Teresa Morales Guzmán*[1], *Víctor Vargas Barroso*[1]

[1]*Instituto de Neurobiología at the Universidad Nacional Autónoma de México. Mexico*

Olfactory systems are among the most complex sensory modalities in neuroscience. In Mammals, the main and accessory olfactory systems detect chemicals that signal important cues from the environment. The detection of these cues is crucial for expressing adaptive behaviors that ultimately enable individuals to enhance their biological fitness. Over a hundred years ago, Santiago Ramón y Cajal proposed the seminal notion that nerve impulses follow a "polarity" rule based on the layered organization of the main olfactory bulb (MOB). Many years later, Dr. Jorge Larriva Sahd, an academic offspring of Cajal (R. Lorente de Nó's protegee), provided the most detailed description to date of the cellular organization, neuronal types, and putative local connectivity of the accessory olfactory bulb (AOB). This detailed description, along with the proposed nomenclature, has since been widely adopted by the scientific community. Aided by the expert analysis of Golgi- stained specimens, Dr. Larriva's excellent neuroanatomical descriptions were frequently coupled to functional predictions, which, in most cases, were eventually substantiated. His novel description of the interstitial neurons of the bulbi (INBs), situated in the Olfactory Limbus, a transition area between the MOB and AOB, leads to the proposal that these neurons could potentially participate in the detection of both pheromones and conventional odorants, and/or as a substrate for communication between the two systems. Indeed, using whole-cell patch clamp recordings in vitro, Larriva and colleagues later demonstrated that these neurons projected axonal collaterals (antidromic responses) to the MOB and received afferents (EPSPs) from the AOB. Dr. Larriva's scientific contributions to the field of olfactory neuroscience have provided a morphological and structural framework that will continue to foster important research and potentially exciting discoveries.

### References

1 The accessory olfactory bulb in the adult rat: a cytological study of its cell types, neuropil, neuronal modules, and interactions with the main olfactory system. Larriva-Sahd J. J Comp Neurol. 2008 Sep 20;510(3):309-50. doi: 10.1002/cne.21790.
2 Olfaction and Pheromones: Uncanonical Sensory Influences and Bulbar Interactions. Vargas-Barroso V, Peña-Ortega F, Larriva-Sahd JA. Front Neuroanat. 2017 Nov 15;11:108. doi: 10.3389/fnana.2017.00108.
3 Electrophysiological Evidence for a Direct Link between the Main and Accessory Olfactory Bulbs in the Adult Rat. Vargas-Barroso V, Ordaz-Sánchez B, Peña-Ortega F, Larriva-Sahd JA. Front Neurosci. 2016 Jan 26;9:518. doi: 10.3389/fnins.2015.00518.

**abstract citation id: bjaf055.043**

### Diseases that affect olfaction, studies in Mexico

*Benito Ordaz Sánchez*[1]

[1]*Instituto de Neurobiología, Universidad Nacional Autónoma de México. Mexico*

Mexico has a long tradition in Neurosciences and the study of alterations in olfactory functions under a variety of pathological conditions is not an exception. Hyposmia is a common early sign in several neurodegenerative diseases and Mexican neuroscientists have provided major contributions to reveal the clinical manifestations of this phenomenon, mainly in Alzheimer's and Parkinson's disease patients. Moreover, Mexican neuroscientists have studied the cellular mechanisms of these alterations in preclinical models in rodents and insects. Olfactory dysfunction associated with mood disorders and schizophrenia have also been characterized in humans and animal models by a different Mexican Research Institutions, using a variety of clinical and experimental approaches. Several Mexican laboratories have identified cellular and molecular alterations in olfactory sensory neurons directly obtained from patients suffering from the already mentioned diseases.

Other neurological alterations exhibiting olfactory alterations characterized by Mexican researchers, in clinical and preclinical settings, are epilepsy, spinocerebellar ataxia and obstructive sleep apneas, among others. Research about alterations of smell during disease performed by scientific groups in Mexico also includes diseases beyond the central nervous system such as diabetes and kidney diseases, among others. Finally, is worth mentioning that a wealth of Mexican research groups has studied olfactory alterations related with environmental pollutants as well as central and peripheral inflammation. This is just a glimpse of the research efforts made by Mexican researchers and scientific Institutions that exemplify the strong tradition of our country in the global effort to understand the olfactory dysfunctions associated to different pathological conditions, aiming for their basic clinical and biological understanding but also potentially reveal avenues leading to their treatment.

**abstract citation id: bjaf055.044**

### CHEMOSENSORY LOSS IN LONG COVID

### *Symposium Organizers: Thomas Hummel; Thomas Finger*

### Long-term effects of COVID-19 on taste function: psychophysics, histology and molecular markers

*Thomas Finger[1], Hanna Morad[2], Tytti Vanhala[3], Marta A. Kisiel[2], Agnes Andreason[2], Mei Li[1], Göran Andersson[3], Göran Laurell[2], Göran Hellekant[2,4]*

*[1]University of Colorado Sch. of Medicine. United States*
*[2]Uppsala University, Sweden*
*[3]Swedish University of Agricultural Sciences, Sweden*
*[4]University of Wisconsin, United States*

Persistent taste dysfunction may occur both as acute and long-term symptoms of SARS-CoV-2 infection (Long COVID), yet the underlying mechanisms are unknown at the histological, cellular, and molecular levels. This study investigates some of its underlying pathology in 28 subjects who reported persistent taste disturbances for over 12 months after testing positive for SARS-CoV-2. To objectively establish the nature of taste deficit in the subjects we used the WETT taste test which quantitatively assesses the subject's ability to taste each of the five basic taste qualities: sweet, umami, bitter, sour and salty. We then biopsied five to seven fungiform taste papillae (FP) in 20 of the subjects. After biopsy, the FPs were analyzed histologically and by quantitative PCR (qPCR) for mRNA expression of markers for different taste receptor cells. Although, all subjects had reported subjective taste dysfunction, only five showed taste scores below the 20th percentile on overall taste sensitivity. However, 12 of 28 subjects exhibited total loss of sensitivity for one or more specific tastes. Loss of PLCβ2- dependent taste qualities (sweet, umami, bitter) was significantly more common and correlated with reduced expression of PLCβ2 mRNA. Histological analysis revealed generally preserved taste bud structure and innervation, but occasional disorganized taste buds and abnormal, scattered PLCβ2+ cells. Our findings suggest long-term taste dysfunction after COVID-19 disproportionately impacts PLCβ2-dependent taste qualities and is not due to widespread structural damage to the taste periphery.

**Funding:** This research was funded by Åke Wibergs Stiftelse (M23-0133) and by grant 3R01DC014728-05S1 from NIDCD of the National Institutes of Health (USA).

**abstract citation id: bjaf055.045**

### Psychophysical assessment of gustatory dysfunction in COVID-19

*Constantin Hintschich[1]*

*[1]University Hospital of Munich (LMU), Germany*

Chemosensory dysfunction is a highly important symptom of COVID-19. Large meta-analyses have reported that up to 48% of patients experience taste loss. However, most diagnoses were based on patients' self-reports, whereas various studies showed that when gustatory dysfunction was assessed by psychophysical tests, the prevalence was much lower. This discrepancy may be due to the misinterpretation of impaired retronasal olfaction as gustatory dysfunction. On the other hand, hypogeusia can be confirmed using validated tests in around 20% of cases. Over the course of the disease, these impairments significantly decrease, indicating the regeneration of the sense of taste. Overall, psychophysical testing is highly relevant for both the diagnosis of gustatory dysfunction in COVID-19 and its assessment over time.

**abstract citation id: bjaf055.046**

### Protocol for endoscopic sampling and histomolecular analysis of human olfactory cleft mucosa in an outpatient setting

*Laura van Gerven[1], Julie van Waterschoot[2]*

*[1]University Hospitals Leuven. Belgium*
*[2]UZ/ KU Leuven. Belgium*

**Background:** Olfactory dysfunction is one of the key symptoms of a Coronavirus Disease (COVID-19) infection, and although the pandemic is already some time behind us, the exact mechanism underlying this COVID-19-associated olfactory dysfunction (C19OD) remains unclear. To acquire fundamental knowledge about the pathophysiology of C19OD, a method for accurately sampling and analyzing olfactory cleft mucosa on a histomolecular level is required.
**Objective:** We present a protocol for harvesting olfactory cleft mucosa in an outpatient setting, as employed in the DysOSMIC study – a multicenter, prospective study including 240 patients, equally divided between those with and without persistent C19OD, across 6 international centers. To objectively assess olfactory function, we perform a validated psychophysical olfactory test, the Sniffin' Sticks, prior to and after tissue sampling. We aim to investigate C19OD by analyzing structural and cellular changes within the olfactory mucosa using advanced techniques including immunofluorescence detection of antigens and RNA, and single-cell RNA sequencing. We developed a minimally invasive approach designed to optimize sample quality while preserving structural integrity, which is essential for accurate analysis.

The novelty of this project lies in the sampling procedure performed in an outpatient setting, combined with parallel information about olfactory function and histomolecular analysis of pathophysiology on samples of living subjects.

**Conclusion:** The pathophysiology of C19OD remains poorly understood. To address this, we developed a novel step-by-step study protocol to obtain high-quality olfactory cleft mucosa samples from patients with and without persistent C19OD. Our goal is to elucidate the underlying mechanism of C19OD through detailed histomolecular analysis.

**abstract citation id: bjaf055.047**

### Parosmia following COVID-19: clinical aspects, pathophysiology, therapeutic perspectives

*Susanne Weise*[1]

[1]*Dept. Otolaryngology, University Hospital Dresden, Germany*

Parosmia is a qualitative olfactory dysfunction in which the perception of an odor is distorted and mainly unpleasant. Parosmia has gained increasing attention due to its high prevalence following COVID-19. However, parosmia has also previously been described following other upper respiratory tract infections or trauma.

Parosmia typically manifests a few months after acute SARS-CoV-2 infection, often following an initial anosmia or hyposmia. Patients frequently report severe impacts on nutrition and quality of life. The pathophysiology of parosmia involves both peripheral and central mechanisms, with damage to the olfactory epithelium and alterations in the central processing.

This presentation reviews recent clinical findings on parosmia following COVID-19 regarding clinical aspects, underlying pathophysiology, and diagnostic options. Further, established therapeutic approaches like olfactory training and recent clinical trials including injections of platelet-rich-plasma are critically evaluated.

**abstract citation id: bjaf055.048**

### Olfactory substitution by the trigeminal system in humans: a promising therapeutic approach

*Susanne Weise*[1], *Halina Stanley*[2], *Clementine Lipp*[3], *Coralie Mignot*[1], *Kostas Garefis*[4], *Maxime Fieux*[2], *Evangelia Tsakiropoulou*[5], *Sissy Genetzaki*[5], *Romain Dubreuil*[6], *Camille Ferdenzi-Lemaitre*[2], *Marina Carulli*[7], *Arnaud Bertsch*[3], *Juergen Brugger*[3], *Iordanis Konstantinidis*[4], *Moustafa Bensafi*[2], *Thomas Hummel*[1]

[1]*Smell & Taste Clinic, Department of Otolaryngology, Technische Universität Dresden, Dresden. Germany*
[2]*Université Claude Bernard Lyon 1, CNRS, INSERM, Centre de Recherche en Neurosciences de Lyon CRNL U1028 UMR5292, NEUROPOP, F-69500, Bron. France*
[3]*Microsystems Laboratory LMIS1, École Polytechnique Fédérale de Lausanne (EPFL), Lausanne 1015. Switzerland*
[4]*2nd Academic ORL Department, Papageorgiou Hospital, Aristotle University, Thessaloniki. Greece*
[5]*2nd Academic ORL Department, Papageorgiou Hospital, Aristotle University, Thessaloniki, Greece*
[6]*Aryballe, 7 rue des arts et métiers, 38000 Grenoble. France*
[7]*Dipartimento di Meccanica, Politecnico di Milano, Via La Masa 1, Milan. Italy*

**Introduction:** Olfactory dysfunction (OD) affects around one-fifth of the population, which can lead to a diminished quality of life. Limited treatment options and recent advances in technologies such as artificial noses are moving olfactory implants into closer reach. Trigeminal stimulation offers a promising, less invasive strategy targeting neural pathways closely interlinked with the olfactory system. This multicentric study aimed to assess the sensitivity of patients with OD to electrical stimuli and their ability to discriminate between different patterns of stimulation.

**Method:** The first study compared the electrical intranasal sensitivity in patients with OD (n=54) of different etiologies and healthy individuals (n=28). A second study involving patients with OD (n=52) and healthy individuals (n=13) assessed their ability to detect and discriminate two patterns of electrical stimuli, which were triggered by a coupled electronic nose.

**Results:** The first study revealed a preserved trigeminal function for the majority of patients with OD. The second study showed that >70% of the patients were able to discriminate between two stimulation patterns. However, 100% of the participants were able to detect the stimuli.

**Discussion:** Trigeminal stimulation could be a promising option to substitute the olfactory system in patients with OD.

**Funding:** This Project has received funding from the European Union's Horizon 2020 research and innovation programme under grant agreement No 964529 (Pathfinder Rose project).

**abstract citation id: bjaf055.049**

### Grey matter alterations in persistent COVID-19-related quantitative and qualitative olfactory dysfunction

*Birgit van Dijk*[1], *Paul Smeets*[1], *Sanne Boesveldt*[1]

[1]*Wageningen University & Research. The Netherlands*

Five years after the onset of the pandemic, the pathogenesis of persistent COVID-19-related olfactory dysfunction (OD) is still unknown. Previous reports have found evidence for both peripheral and central mechanisms, which might differ between quantitative and qualitative symptoms. In the current study we utilized voxel-based morphometry analysis of brain MRI's to study grey matter (GM) alterations in patients with persistent OD after COVID-19 infection. We included patients with persistent (>1 month) anosmia or hyposmia (Sniffin' Sticks score ≤30.5, n=23) and parosmia (self-reported, n=25) aged 18 - 60 years. A

subset of patients (n=28) underwent a second MRI after six months to assess potential recovery.

Additionally, a control group of 48 normosmic control subjects aged 18 - 65 years was included. ROI analysis in olfactory- related regions showed no significant differences in GM density between groups at a corrected threshold, both in the cross- sectional and longitudinal analysis. However, using the expected voxels per cluster as an exploratory threshold, lower GM density was observed in parts of the right anterior cingulate, right middle and superior orbitofrontal cortex, left gyrus rectus, right olfactory cortex, and caudate in quantitative OD versus controls. Parosmic patients showed lower GM density in the superior orbitofrontal cortex compared to controls, and patients with quantitative OD had lower GM density compared to parosmics in the left hippocampus, right insula and left caudate. The Sniffin' Sticks score was associated with GM density in the left putamen in the longitudinal cohort. Whole-brain analysis did reveal significantly lower GM density in patients with quantitative OD compared to controls in part of the left inferior parietal gyrus and various regions related to attention, working memory and executive function. These results contribute to the current understanding of the mechanism behind persistent COVID-19-related olfactory dysfunction.

**Funding:** ZonMW (10430102110001) & NWO/Aspasia (015.013.052)

**abstract citation id: bjaf055.050**

### CHRONIC RHINOSINUSITIS AND OLFACTORY DYSFUNCTION

### *Symposium Organizer: Steven Munger*

### What nasal mucus can teach us about CRS-related olfactory loss

*Jennifer Mulligan*[1]

[1]*University of Florida. United States*

Traditionally, research in olfactory biology has focused on neuronal signaling and central processing, with less emphasis on how the sinonasal microenvironment could influence this process. Recent advances have shifted this paradigm, positioning nasal mucus as a rich, underexplored biospecimen which contains molecular biomarkers relevant to both olfactory function and dysfunction. Emerging studies reveal that nasal mucus contains a diverse array of proteins, cytokines, and odorant- binding molecules, that are reflective of sinonasal tissue and can give us valuable insights into mechanisms of olfactory loss. These biomarkers are proving valuable in diagnosing olfactory disorders, characterizing inflammatory endotypes, such as in CRS, and monitoring recovery in response to targeted therapies. In this presentation, we will also discuss some of the practical considerations for the collection of nasal mucus and compatible assays. As the field advances, nasal mucus biomarkers hold the potential to transform our understanding of olfaction, providing a window into the intersection of mucosal immunology and sensory neuroscience, and unlocking new diagnostic and therapeutic avenues in translational olfactory research.

**abstract citation id: bjaf055.051**

### Restoring Smell in Chronic Rhinosinusitis with Nasal Polyps: Mechanistic Insights from Biologic Therapy and Real-World Outcomes

*Jose Mattos*[1]

[1]*University of Virginia. United States*

Loss of smell is one of the most disruptive symptoms experienced by patients with chronic rhinosinusitis with nasal polyps (CRSwNP), affecting daily safety, nutritional enjoyment, and overall quality of life. While biologic agents such as dupilumab have reshaped the treatment landscape, the processes that lead to smell recovery remain only partly understood.

In this session, we will share prospective, real-world data from 62 patients with CRSwNP who began dupilumab therapy at two tertiary rhinology centers. Within 12 weeks, participants demonstrated striking, clinically relevant gains: mean Threshold-Discrimination-Identification (TDI) scores rose by over seven points, SNOT-22 scores fell by more than 21 points, and Quality of Olfactory Disturbance (QOD) scores improved by 11 points (all $p < 0.001$).

Objective measures mirrored these patient-reported changes. Average olfactory cleft opacification on CT dropped from 77% to 58% ($p < 0.001$), and endoscopic olfactory cleft scores improved from 6.46 to 4.06 ($p < 0.01$). Both imaging and endoscopic changes showed significant correlations with olfactory recovery ($r = -0.36$ and $r = -0.38$), whereas peak nasal inspiratory flow did not —supporting the concept that targeted reduction of olfactory cleft inflammation, rather than increased airflow, drives improvement.

Attendees will come away with a deeper understanding of dupilumab's effect on the olfactory cleft, strategies for using imaging and endoscopy to track progress, and practical tips for identifying patients likely to benefit early in the treatment course. These findings refine our understanding of smell restoration and offer a framework for personalized management in CRSwNP.

**abstract citation id: bjaf055.052**

### The MACRO Trial: Assessing the effectiveness of medical and surgical treatments for the treatment of chronic rhinosinusitis

*Carl Philpott*[1]

[1]*University of East Anglia. United Kingdom*

A lack of evidence regarding use of antibiotics and endoscopic sinus surgery (ESS) in managing chronic rhinosinusitis (CRS) has contributed to 5-fold variation in ESS rates and antibiotic use. The aim of the trial was to compare clinical and cost-effectiveness of ESS or 3 months of clarithromycin alongside intranasal corticosteroids and saline irrigations (intranasal medication (IM) in adults with CRS with (CRSwNP) or without nasal polyps (CRSsNP). A 3-arm randomised controlled trial was conducted at 21 UK secondary/tertiary care sites. CRS patients remaining symptomatic despite IM were randomised to receive either ESS plus IM, clarithromycin (250mg bd for 2 weeks then od for 10 weeks) plus IM or placebo plus IM. Participants were allocated 1:1:1 using an automated, web-based secure randomisation system. Participants/site teams were blinded to medical interventions only. Primary outcome measure was Sino-nasal Outcome Test (SNOT-22) quality-of-life questionnaire at six months. Planned sample size was 510. An intention-to-treat analysis was undertaken. ISRCTN number: 36962030 (17th October 2018). Between 11/2018 and 11/2023, 514 participants (181 (35.2%) female and 333 (64.8%) male), with CRSwNP (n=410) or CRSsNP (n=104), were recruited and randomised to IM plus ESS (n=171), clarithromycin (n=172), or placebo (n=171). SNOT-22 scores were significantly lower following ESS than clarithromycin (-18.13, 98.33% CI -24.26, -11.99) and placebo (-20.44, 98.33% CI -26.42, -14.46). There was no significant difference in SNOT-22 scores between participants randomised to clarithromycin or placebo (-3.11, 98.33% CI (-8.56, 2.33)). Serious adverse events occurred in 11 participants (clarithromycin (2), placebo (4) and ESS (5)). The MACRO trial demonstrates that ESS is clinically effective with CRS patients having significantly improved disease-specific quality of life at 6 months and does not support routine use of low-dose clarithromycin. ESS should be recommended if IM alone fails to achieve symptom control.

**abstract citation id: bjaf055.053**

### HEALTHY SMELLING: HOW SMELLING WELL IMPACTS OUR WELL-BEING

### *Symposium Organizer: Mehmet Mahmut*

### Cognitive and emotional well-being in people with an olfactory dysfunction

*Michal Pieniak*[1]

[1]*University of Wroclaw. Poland*

The neuroanatomical structures involved in the perception of smells are also engaged in a range of cognitive and emotional processes. A similar connection is evident at the behavioral level, where individuals with olfactory dysfunction may exhibit symptoms of cognitive decline and compromised emotional well-being. This talk will review current research on the prevalence of cognitive and emotional disturbances in individuals who lost their sense of smell. The discussion will also briefly consider the potential role of smell tests as screening tools for early detection of neurodegenerative diseases. Finally, the challenges and limitations of assessing the interplay between olfactory, cognitive, and emotional dysfunction will be discussed.

**abstract citation id: bjaf055.054**

### Olfaction, nature contact, and human well-being

*Gregory Bratman*[1]

[1]*University of Washington. United States*

Mounting evidence links nature contact to multiple dimensions of human well-being, yet much of the research in this emerging field has focused on the visual pathway. This talk will present a framework that integrates research from psychoneuroimmunology, environmental and cognitive psychology, and Shinrin-yoku (“forest bathing”) to explore the underexamined role of olfaction in nature and health. We will also discuss recent empirical work that investigates exposures to biogenic volatile organic compounds in a forest setting, as well as potential changes in nasal microbiome characteristics related to well-being that may occur from immersion in natural environments.

**abstract citation id: bjaf055.055**

### How smelling good helps build healthy social and romantic relationships

*Mehmet Mahmut*[1]

[1]*Macquarie University. Australia*

People report falling in love at first sight, but can we fall in love at first smell? If so, what are the underlying mechanisms that may explain the connection between olfaction and relationships? This presentation will unpack the research findings that indicate our sense of smell plays a role in maintaining healthy social and romantic relationships. Specifically, research findings from people with and without a sense of smell and from those who are single or in romantic relationships, will be drawn upon to highlight how olfaction is connected to our social and romantic experiences and in turn impacts our well- being.

**abstract citation id: bjaf055.056**

### Smell and Emotion – Myths, Mechanisms, and Clinical Implications

*Ilona Croy*[1]

[1]*Friedrich Schiller University Jena. Germany*

Odors and emotions share a close evolutionary connection. Early neuroscientist Paul MacLean even referred to what is now known as the limbic system—the brain's emotional center—as the "olfactory system," due to its strong anatomical ties to structures involved in olfactory processing. In line with this neural overlap, olfactory dysfunction is frequently associated with altered emotional perception, including symptoms such as anhedonia and depression.

But how substantial is the evidence behind these associations? This talk aims to disentangle myths from empirical facts and to explore the fundamental mechanisms linking smell and emotion. Additionally, we will discuss emerging approaches, including minimally invasive olfactory-based interventions for the treatment of depression.

**abstract citation id: bjaf055.057**

### Healthy Smelling: Concluding Remarks

*Anna Oleszkiewicz*[1]

[1]*University of Wroclaw. Poland*

For a long time, the sense of smell was considered the neglected stepbrother of human sensory abilities, and the loss of smell has received little attention. This perception changed dramatically with the COVID-19 pandemic, which led to millions of people temporarily losing their sense of smell. COVID-19 not only increased general awareness of olfactory disorders but also accelerated research into the role of smell in nonverbal communication and mental health. This talk summarizes the impact of olfactory disorders on physical, psychological, and social well-being, as well as the benefits of exposure to odors. It offers the conclusion that the consequences of olfactory loss are not always profoundly severe, but they permeate many spheres of daily life.

**abstract citation id: bjaf055.058**

### VOMERONASAL ORGAN: DEVELOPMENT, FUNCTION, AND FUTURE PERSPECTIVES

### *Symposium Organizers: Jean-François Cloutier; Paolo E. Forni*

### One System, Many Strategies: Morphological and Functional Diversity of the Mammalian VNS

*Pablo Sanchez Quinteiro*[1], *Irene Ortiz Leal*[1]

[1]*University of Santiago de Compostela. Spain*

The vomeronasal system (VNS) plays a fundamental role in modulating social and reproductive behaviors in mammals. However, it is far from being a uniform sensory structure. Comparative anatomical, functional and genomic studies have revealed an extraordinary diversity in the VNS across mammalian taxa, reflecting the evolutionary trajectories, ecological adaptations, and social organization of different lineages. This morphofunctional diversity highlights the limitations of generalizing data obtained from traditional laboratory models, especially rodents, and underlines the need to explore non- model species to achieve a more comprehensive and nuanced understanding of the VNS.

Two main axes define this variability: first, the differential expression of vomeronasal receptors, which tends to follow either a segregated (dual receptor zones projecting to distinct regions of the accessory olfactory bulb, AOB) or a uniform model (single-type receptor organization with non-segregated projections). Second, the architecture and neurochemical organization of the AOB itself — the central processing hub of vomeronasal input — shows significant differences across species, some of which remain poorly understood or completely unexplored.

In this presentation, we discuss the morphofunctional diversity of the VNS in selected mammalian species, emphasizing its evolutionary and behavioral correlates. We argue that a broader, integrative, and comparative approach is essential not only to understand the adaptive roles of this system across taxa, but also to approach one of the major scientific enigmas that persist today: the function — if any — of the vomeronasal system in humans. Revisiting this question from a multi-species perspective opens up new possibilities for understanding the sensory bases of social communication in mammals, including our own.

**abstract citation id: bjaf055.059**

### The molecular diversity of the endoplasmic reticulum in vomeronasal sensory neurons is essential for maintaining their homeostasis and identity

*Paolo Forni*[1]

[1]*University at Albany, SUNY. United States*

The vomeronasal organ (VNO) is a chemosensory epithelium that exhibits significant neuronal heterogeneity. These differences across vomeronasal sensory neurons (VSNs) largely depend on the expression of distinct transcription factors, the vomeronasal receptor expressed, guidance cues, and adhesion molecules expressed. The vomeronasal neuroepithelium in rodents comprises two main types of vomeronasal sensory neurons, both of which arise from a common progenitor pool. VSNs of mice express receptors from either of two evolutionarily distinct families of vomeronasal receptor (VR) genes: V1Rs or V2Rs. Neurons expressing V1Rs connect with mitral cells in the anterior accessory olfactory bulb (AOB), while V2R- expressing VSNs connect to mitral cells in the posterior AOB. In the AOB, axons of neurons expressing similar VR or functionally related VR genes converge to form glomeruli along with the dendrites of specific mitral cells. Single-cell transcriptomics have enabled us to identify previously unrecognized genetic differences between VSNs expressing V1R and V2R. Notably, we found significant variations in the endoplasmic reticulum (ER) protein repertoires between V1R and V2R

VSNs. Our data from mutant animals suggest that different cell-type-specific ER protein profiles across the two main types of VSNs dictate their unique capability to process cell-type-specific protein repertoire, manage the neuronal type-specific endoplasmic reticulum stress, and ultimately influence functionality, connectivity and homeostasis.

**Funding:** This work was supported by t(NICHD) under Grants 2R01HD097331 (P.E.F.) and 1R01HD114827 (P.E.F.) and (NIDCD) under Grant R01DC017149 (P.E.F.).

**abstract citation id: bjaf055.060**

### Glomerular map formation in the control of social behaviours

*Jean-François Cloutier*[1], *Sydney Fearnley*[1]

[1]*The Neuro - Montreal Neurological Institute and Hospital - McGill University. Canada*

The flow of information and its processing in the nervous system relies on the formation of selective connections between neurons. More specifically, the establishment of accurate neural maps in sensory systems permits the detection and interpretation of cues from the environment. The formation of this circuitry depends on multiple processes, including the guidance of axons to their target fields and the formation of synapses with their synaptic partners. In the accessory olfactory system, which regulates social and sexual interactions in mice, vomeronasal sensory neurons (VSNs) project their axons to the accessory olfactory bulb (AOB), where they form synapses with second-order mitral cell dendrites inside glomeruli.

Axons of VSNs expressing the same type of vomeronasal receptor coalesce together to form homogenously innervated glomeruli that are spatially conserved in the AOB. The establishment of this glomerular map is proposed to contribute to the representation of the phenotypic qualities of chemosignals detected by VSNs and to the expression of specific social behaviours. Members of the Kirrel family of receptors play a critical role in the accurate coalescence of VSN axons into glomeruli and in the formation of the AOB glomerular map. Using cell-type-specific Kirrel3 loss-of-function approaches to disrupt glomerular map formation, we establish a link between the accurate formation of the AOB glomerular map and vomeronasal acuity in the modulation of VNO-dependent behaviours.

**abstract citation id: bjaf055.061**

### How conspecific chemical communication controls complex behaviors

*Marc Spehr*[1]

[1]*RWTH Aachen University. Germany*

In most mammals, conspecific chemical communication controls complex behaviors. Information about individuality, health, social and reproductive status is conveyed by an elusive class of chemical cues – pheromones. The highly reproducible character of pheromone responses offers a unique opportunity to uncover the neuronal basis of genetically programmed behavior. Despite its fundamental significance, however, the basic chemosensory mechanisms of social communication remain largely unknown. To address these issues, my group has developed a multi-faceted approach to uncover the mechanisms underlying mammalian pheromone sensing. Combining molecular, biochemical, (electro)physiological, and imaging methods, as well as behavioral techniques in wildtype and mutant mouse models, our research analyzes the principle coding logic of pheromone detection, and, thus, sheds light on the neurophysiological basis of social behavior.

**abstract citation id: bjaf055.062**

### The main and accessory olfactory pathways converge in distinct amygdaloid nuclei

*Moritz M Nesseler*[1], *Leonie Büsching*[1], *Marc Spehr*[1]

[1]*Department for Chemosensation, Institute of Biology II, RWTH Aachen University, Aachen, Germany*

Most mammals employ at least two specialized olfactory systems to sense their chemical environment. The relevant peripheral sensory structures, i.e., the main olfactory epithelium and the vomeronasal organ, relay information to the main and accessory olfactory bulbs, respectively. In these circuits, main and accessory olfactory information is separately processed and projected towards largely segregated downstream targets. Here, we ask whether the main and accessory olfactory pathways converge within downstream nuclei. We combine Cre-dependent expression of rAAV genomes in transgenic Tbx21-Cre driver mice to label and trace main and accessory olfactory bulb principal neurons. Notably, we identify both divergent and convergent projections. Two amygdaloid nuclei, the bed nucleus of the accessory olfactory tract (BAOT) and the anteroventral medial amygdala (avMeA), show convergent axonal projections from both olfactory pathways. Next, we use single-cell patch-clamp recordings in acute brain slices to characterize the electrophysiological and morphological properties of neurons within both nuclei. We identify both unique and shared electrophysiological properties between BAOT and avMeA neurons. In addition, we provide a detailed morphological library of reconstructed neurons from either convergence area. In summary, we demonstrate that main and accessory olfactory pathways converge in two distinct amygdaloid nuclei, each harboring neurons with unique electrophysiological and morphological signatures.

abstract citation id: bjaf055.063

### NEUROMODULATION AND PLASTICITY IN OLFACTORY CIRCUITS ACROSS SPECIES

### *Symposium Organizer: Elisa Galliano*

### Neuromodulators drive sex-specific behavioral plasticity in C. elegans

*Sonu Peedikayil Kurien*[1]

[1]*University of Cambridge. United Kingdom*

Divergence in sex-specific evolutionary drives influences behavioral decisions. How each sex learns from a shared experience is still an open question. Using an ethologically relevant paradigm, we reveal sexual dimorphism in pathogen avoidance: C. elegans male do not avoid the pathogenic bacteria PA14 as efficiently and rapidly as hermaphrodites, even though the pathogenicity is perceived. The neuronal representations following training display this dimorphism and suggest a sensory gating mechanism in males. Transcriptomic and subsequent behavioral analysis reveal the role of the neuromodulatory network in male behavior, with npr-5, an ortholog of the mammalian NPY receptor, regulating male behaviour by modulating typical neuronal activity. Furthermore, male decision-making behavior is contingent on sexual status and is modulated by npr-5. Taken together, behavior towards a shared experience is modulated by genetic sex in a context dependent manner.

abstract citation id: bjaf055.064

### Synaptic depression outperforms potentiation in learned stimulus discrimination under relative integration of opposing outputs

*Andrew Lin*[1]

[1]*University of Sheffield. United Kingdom*

Why are brains the way they are? Are their circuit architectures and synaptic plasticity rules in some sense ‘optimal’? If so, in what sense, or in what contexts? We address these questions using olfactory associative memory in the fruit fly Drosophila. Flies can learn to associate a particular odour with a reward (e.g., food) or punishment (e.g., shock) and thereafter approach or avoid the trained odour. These associative memories are stored in Kenyon cells in the mushroom body, by weakening synapses from odour-responsive Kenyon cells onto mushroom body output neurons (MBONs) that lead to incorrect actions (e.g., odour+punishment weakens KC->Approach synapses). Why weaken incorrect actions rather than strengthening correct actions? Notably, synaptic depression is also used for learning in the vertebrate cerebellum, which has a remarkably similar architecture to the insect mushroom body, suggesting that using depression may be functionally advantageous.

We show both analytically and using simulations that depression outperforms potentiation for discriminating odours with overlapping KC representations, under a particular condition: if behaviour depends on the relative, not the absolute, difference between Avoid vs. Approach MBON activities. To test whether behaviour depends on the relative difference, we measured aversive learning for a range of odour concentrations and punishment intensities. We automatically tracked the flies’ decisions to enter or leave the side with the punished odour, and from the statistical distributions of these stochastic decisions, we inferred the mean and variance of the flies’ underlying preference for/against the odour. Bayesian modelling indicated that the data best fit a model where behaviour depends on the relative, not the absolute, difference between Avoid and Approach. These results suggest that flies learn by synaptic depression because, in the mushroom body, it is computationally superior to synaptic potentiation.

abstract citation id: bjaf055.065

### Neural bases of childhood olfactory memory in mice

*Jules Dejou*[1]*, Anna Athanassi*[1]*, Theo Brunel*[1]*, Marc Thevenet*[1]*, Anne Didier*[1]*, Nathalie Mandairon*[1]

[1]*Centre de Recherche en Neurosciences de Lyon. France*

Marcel Proust, in Swann’s Way (1919), recounts the profound joy triggered by the scent of a madeleine dipped in linden tea, transporting him back to afternoons spent with his grandmother. This iconic example illustrates the strong link between olfaction and autobiographical memory. Research has shown that odor-evoked memories tend to be older and more emotionally positive than other memories, yet their neural mechanisms remain poorly understood. To investigate this, we first conducted a human survey to better characterize our earliest olfactory memory and showed that this memory was typically highlighted by the highly repetitive (more than five times) association of a pleasant odorant with a positive original event. Based on this information, we then developed a mouse model of positive childhood olfactory memory and investigate its neural signature in adulthood. By repeatedly pairing a pleasant odorant with a positive enriched environment during postnatal days (P) P23 to P33, we induced a lasting attraction and memory to this odorant in adulthood (2 months). Focusing on the olfactory bulb – a site of olfactory memory and postnatal neurogenesis – we showed that P1-born granule cells code the olfactory memory as they were preferentially reactivated by the childhood odorant and their photo-inhibition disrupted memory recall. We then analyzed the output of the olfactory bulb and identified a functional connectivity network involving reward and memory systems in response to the childhood odorant. Maintaining the olfactory memory over the long term (6 months) required regular re-exposure to the odorant, as observed in humans. At this stage, the memory no longer preferentially involved P1-born granule cells, but was accompanied with a brain functional reorganization centered around the olfactory-limbic system. Altogether, this project offers novel insights into how the brain supports the persistence of early and emotionally powerful olfactory memories.

**abstract citation id: bjaf055.066**

### Olfactory dopaminergic cells: key players in sexual odor perception

*Serena Bovetti*[1]

[1]*Dept. of Life Sciences and Systems Biology and Neuroscience Institute Cavalieri Ottolenghi. University of Turin, Italy*

Olfaction is one of the most conserved senses in animal evolution, and many species rely heavily on it for behaviour, including mate choice and reproduction. In mice, the main olfactory bulb (MOB) is the forebrain region that harbours the most numerous dopaminergic (DA) population, identified by the expression of the tyrosine hydroxylase (TH) enzyme. TH+ cells are inhibitory interneurons classified into two major categories based on their morphology, generation time and biophysical properties. However, whether MOB dopaminergic cells are involved in the processing of sexual odours remains largely unknown. Here, we investigated the recruitment of OB dopaminergic cells in female mice exposed to sexual odours contained in the urine of the opposite sex. We also investigated whether the recruitment of OB dopaminergic cells differs between exposure to familiar and unfamiliar male odours. Using whole-brain labelling of the immediate early gene c-fos and light-sheet microscopy, we show that in females, brief and acute exposure to opposite-sex odorants increases neuronal activation mainly in the most dorsal-posterior region of the OB. In this region, we detect a selective recruitment of olfactory dopaminergic cells, with large DA cells showing higher basal activation compared to small ones. Interestingly, unfamiliar male odours recruit a greater proportion of OB DA cells compared to familiar odours. To further analyse the in vivo physiological response of dopaminergic interneurons to opposite-sex odours, we performed functional in vivo 2-photon calcium imaging to assess the contribution of OB dopaminergic subpopulations in the processing of opposite-sex odours. With this study, we aim at dissecting the early coding process of salient olfactory information at brain level to finally unravel the role of dopamine in the mouse MOB regarding the reproductive behavior.

**abstract citation id: bjaf055.067**

### Glial modulation of peripheral olfactory responses via Eaat2 in Drosophila

*Alejandro Muñoz-Jimenez*[1], *Fernando Martin*[1], *Esther Alcorta*[1], *Carolina Gomez-Diaz*[1]

[1]*Departamento de Biología Funcional - Área de Genética. Facultad de Medicina - Universidad de Oviedo, Spain*

Glial cells are closely associated with neurons throughout the nervous system. Traditionally, their primary role has been considered supportive, maintaining neuronal function and homeostasis. However, increasing evidence has revealed that glia can actively modulate neuronal activity. While several molecular mechanisms underlying neuron-glia interactions have been identified in the central nervous system (CNS), our understanding of these processes in the peripheral nervous system (PNS) remains limited.

The olfactory system of *Drosophila melanogaster* provides a relatively simple and genetically tractable model to study neuron-glia interactions in the PNS. Previous studies have shown that sensory input from olfactory receptor neurons (ORNs) can influence the activity of antennal perineurial glial cells labelled by the Mz317 driver line. Furthermore, altered expression levels in these glial cells of the *Eaat2* gene, a transmembrane protein involved in taurine/aspartate transport, have been shown to impact olfactory-driven behaviour.

In this study, we investigate the modulatory role of *Eaat2* in perineurial glial cells (labelled by the Mz317 driver line) on olfactory receptor neuron (ORN) activity. We show that strong odor stimulation leads to changes in *Eaat2* expression levels both in the main olfactory organ of Drosophila and specifically in Mz317 glia. Using a combination of calcium imaging and RNA interference (RNAi), we further demonstrate that silencing *Eaat2* in these glial cells alters the ORNs' response to odor stimuli, without affecting the structure of Mz317 glia. Our results indicate that *Eaat2* in Mz317 glia plays a critical role in shaping ORN responses to odor stimuli, highlighting a modulatory function of glia in peripheral olfactory processing.

**Funding:** Ministerio Español de Ciencia, Inovación y Universidades [MCINN-24-PID2023-149482MB-100 to C.G.D.], Fundación Científica Manuel González [APR-22-034 to E.A.], and A.M.J was funded through Programa Investigo [SEPEPA-24-AYUD/2023/36906-3882 to E.A and C.G.D.]

**abstract citation id: bjaf055.068**

### Brain-Wide Coding of Stimulus Valence and Behavior in the Larval Zebrafish Chemosensory System

*Bethan Jenkins*[1], *Thomas Offner*[1], *Thomas Frank*[1]

[1]*University of Göttingen. Germany*

How animals translate chemosensory stimuli into context-appropriate behavior remains a poorly understood question in neuroscience. Using larval zebrafish, we combined behavioral assays and whole-brain two-photon calcium imaging to examine how chemosensory valence is represented in the vertebrate brain and linked to motor output.

Freely swimming larvae reliably exhibited approach or avoidance in response to distinct stimuli, allowing us to classify them as appetitive or aversive. In partially immobilized fish, aversive stimuli triggered immediate increases in locomotor activity, while appetitive stimuli typically suppressed movement, suggesting context- and valence-specific motor programs.

Brain-wide imaging revealed distributed neuronal responses to chemosensory stimulation. Stimulus identity could be decoded from neuronal population activity across many regions. A small subset of neurons encoded stimulus valence rather than identity. Notably, these valence-encoding neurons showed distinct, consistent relationships to behavior, being either correlated or anti-correlated to locomotion depending on the valence sign.

Neurons whose activity tracked behavioral output were more broadly distributed and functionally distinct from valence- encoding cells, though interactions between the two populations were evident. Together, our results support a model in which chemosensory valence and behavior are represented by distributed, interacting networks that operate across multiple stages of sensorimotor processing.

**abstract citation id: bjaf055.069**

### ODORS AND THE BRAIN: A JOURNEY WITH THE SPANISH OLFACTORY NETWORK

### *Symposium Organizers: Raúl Fernández Arias; Angel Gonzalez Wong*

### Computational Studies of the Molecular Determinants of Human Olfaction: From Odorant Chemistry to Receptor Variation

*Angel González Wong[1], Natalia González[2], Clàudia Llinàs del Torrent[3], Ramon Cierco Jimenez[4]*

*[1]Universitat Autònoma de Barcelona. Spain*
*[2]Bellvitge Biomedical Research Institute (IDIBELL), Barcelona, Spain*
*[3]University of California, San Francisco, USA*
*[4]International Agency for Research on Cancer (IARC), Lyon, France*

The human sense of smell emerges from intricate interactions between volatile chemical compounds and olfactory receptors (ORs). This "olfactory code"—comprising the molecular physicochemical requirements for odor detection and the genetic diversity of ORs—constitutes the first step in a complex process of perceptual decoding that culminates in the conscious experience of smell.

**Objectives of the Study:** To investigate how odorant chemistry and genetic variation contribute to perceptual variability in humans.

**Computational Methods:** Two complementary strategies were implemented: 1. Odorant Chemistry: A curated library of 3,827 volatile molecules (18,129 odor labels) was annotated with over 100 2D and 3D physicochemical descriptors per molecule. Data-driven classification models were trained to associate molecular features with perceptual categories. 2. OR Genetics: A total of 119,069 natural variants across 378 human OR genes from global population datasets (gnomAD v2; 141,456 individuals) were mapped onto 3D OR structural models to assess their distribution within key domains involved in ligand binding and signal transduction.

**Results:** Odorant physicochemical descriptors revealed a link between structure and perceptual categories. Classification models indicate that a limited set of molecular properties can reliably predict odor quality, supporting scalable odor characterization. A considerable number of natural variants were localized in critical OR functional regions, suggesting potential impacts on receptor function and contributing to individual and population-level variability in olfactory sensitivity.

**Conclusions:** Odorant physicochemical properties enable accurate prediction of perceptual qualities, while OR genetic variation introduces functional diversity. Integrating this knowledge provides deeper insight into the olfactory code and paves the way for applications in precision fragrance design and personalized chemosensory health.

**Funding:** Ministerio de Ciencia e Innovación (MICINN), PID2022-140912OB-I00.

**abstract citation id: bjaf055.070**

### The Vomeronasal Organ as a Natural Chromatographic Column: Differential Processing of Chemical Signals in Ungulates

*Irene Ortiz Leal[1], Antía Martínez Antonio[1], Gadea García Hernando[1], José Luis Rois[2], Pablo Sánchez Quinteiro[1]*

*[1]Santiago de Compostela University. Spain*
*[2]Marcelle Nature Park. Spain*

The vomeronasal organ (VNO) is a chemosensory structure essential for detecting pheromones and other semiochemicals involved in social, reproductive, and territorial behaviors in mammals. While the functional organization of the VNO has been extensively studied in rodents, much less is known about its structural and molecular complexity in ungulates. In this study, we characterized the VNO in two phylogenetically distant ungulate species—the wapiti (*Cervus canadensis*) and the dama gazelle (*Nanger dama*)—using a broad panel of immunohistochemical and lectin histochemical markers.

We investigated the expression of G-protein subunits Gαi2 and Gαo, associated with V1R- and V2R-expressing vomeronasal sensory neurons, along with additional neuronal and structural markers including OMP, GAP-43, calbindin, calretinin, β-actin, tubulin, Gγ8, and PGP 9.5. A lectin panel was used to assess glycoconjugate heterogeneity. High-resolution imaging was performed using confocal microscopy.

Our results reveal a non-uniform distribution of both protein markers and glycoconjugates along the anterior-posterior axis of the VNO, suggesting that different segments of the VNO may be functionally specialized to detect chemically distinct cues. This spatial variation supports the hypothesis that the VNO functions as a natural chromatographic column, selectively filtering and processing chemical signals throughout its length.

These findings not only enhance our understanding of vomeronasal organization in ungulates but also provide crucial insight for the interpretation of protein and glycoconjugate expression studies in other species. Our data highlight the need for caution when evaluating molecular profiles based on single-point sampling within the VNO, as significant longitudinal variation may be overlooked. Moreover, this work opens the possibility that a similar morpho-functional organization may exist in other mammalian groups, inviting broader comparative studies across taxa.

**abstract citation id: bjaf055.071**

### Neural mechanisms for state-dependent female socio-sexual behavior

*Nicolas Gutierrez Castellanos*[1]

[1]*University of Valencia. Spain*

Cyclic fluctuations in the levels of sex hormones coordinate sexual receptivity with reproductive capacity across the female's reproductive cycle. This is clearly illustrated in mice, as only females in the fertile phase engage in sexual behavior. Outside the fertile period, copulation never occurs because females reject male copulation attempts by running away, adopting defensive postures or even punching and kicking the male. Strikingly, while vast efforts have been dedicated to study female receptivity, rejection behavior has been largely ignored and often regarded as a mere lack of receptivity.

Using calcium imaging to monitor neural activity in vivo together with quantitative analysis of behavior and ex vivo electrophysiological recordings, we show that a subpopulation of neurons expressing progesterone receptor in the anterior portion of the ventromedial hypothalamus (aVMH.PR+) is active during rejection behavior in the non-fertile phase and silent during the fertile phase. These activity changes coincide with a dramatic decrease in the excitation/inhibition synaptic balance of aVMHvl.PR+ neurons in the fertile phase. Furthermore, optogenetic activation of this subpopulation in the fertile phase increases rejection behavior, significantly affecting the female's willingness to engage in sexual behavior.

Altogether, our results describe a new role for aVMH.PR+ neurons in the cyclical control of female rejection behavior.

**abstract citation id: bjaf055.072**

### Uncovering the synaptic involvement of NG2-glia in the olfactory connectome

*Raúl Fernández-Arias*[1], *Sonsoles Barriola Arias*[1], *María Figueres-Oñate*[2], *Laura López-Mascaraque*[1]

[1]*Instituto Cajal - CSIC. Spain*
[2]*Instituto de Salud Carlos III. Spain*

NG2-glia, once primarily classified as oligodendrocyte precursor cells (OPCs), are now recognized as a highly plastic and multifaceted glial population within the CNS. While they have well-established roles in development, migration, and neuroprotection, growing evidence points to their active involvement in synaptic processes. Yet, the exact nature of their participation in synaptic networks is still under active exploration.

A key question is whether NG2-glia function strictly as postsynaptic elements, interact with both GABAergic and glutamatergic synapses, or contribute to synaptic organization through broader physiological functions. Furthermore, the degree to which NG2-glia colocalize with synaptic structures remains unclear, raising critical questions about their spatial integration and functional roles within neural circuits. Their interactions with synaptic suggest a level of complexity that challenges conventional models of glial contributions to circuit organization.

In this study, we investigate the role of NG2-glia within the synaptic landscape of the mouse olfactory bulb (OB), a model system uniquely suited for exploring circuit plasticity due to its well-defined layers and persistent adult neurogenesis. Using transgenic reporter lines specific for NG2/Pdgfra lineage, we employed cutting-edge imaging techniques, including TauSTED- Xtend microscopy, to map the spatial relationships between NG2-glia and five distinct synaptic markers. Automated, distance-based analysis enabled us to assess colocalization patterns across OB layers.

Ongoing work incorporates retrograde and anterograde monosynaptic viral tracing to further map NG2-glia–neuron connectivity. Together, these approaches deepen our understanding of NG2-glia as potentially active organizers and modulators of synaptic structure and function within the OB connectome, and possibly throughout the CNS.

**Funding:** Supported by PID2022 - 136882NB - I00 funded by M ICIU/AEI/10.13039/501100011033. R.F.A . was funded by an FPI grant from the Ministerio de Ciencia, Innovación y Universidades

**abstract citation id: bjaf055.073**

### Signature-based therapeutic repurposing of HDAC1 and PARP-1 inhibitors in Parkinson's disease

*Paz Cartas Cejudo*[1], *Marina De Miguel Sánchez*[1], *Leire Extramiana Esquisabel*[1], *Mercedes Lachén Montes*[1], *Silvia Romero Murillo*[1], *Elena Anaya Cubero*[1], *Sven Berres*[2], *Julian Uszkoreit*[2], *Joaquín Fernández Irigoyen*[1], *Enrique Santamaría Martínez*[1]

[1]*Navarrabiomed. Spain*
[2]*Ruhr University Bochum. Germany*

**Objectives:** This study aimed to identify potential therapeutic candidates for Parkinson's disease (PD) by leveraging molecular signatures derived from affected brain regions with a focus on re-purposing existing drugs.

**Experimental methods:** Proteomic analysis was conducted on olfactory tract (OT) tissue from control individuals with no known neurological history (n=17) and PD subjects (n=21), assessing disease-stage-specific protein alterations and α- synuclein (α-syn) interactome changes. A computational drug repositioning strategy was applied using PD-related brainstem omics signatures (BOSs), incorporating differential data from the OT and nine transcriptomic datasets from PD-relevant brain regions. Selected drug candidates were validated in vitro using olfactory, microglial, and dopaminergic neuronal cell lines treated with α-syn preformed fibrils (PFFs).

**Results:** Proteomic analysis revealed Lewy body disease (LBD) stage-dependent proteostasis impairment and progressive α- syn interactome modulation. Computational screening identified HDAC1 and PARP-1 inhibitors as top candidates capable of reversing multiple BOSs. In vitro experiments demonstrated that pre-treatment with HDAC1 or PARP-1 inhibitors reduced α- syn PFF-induced neurotoxicity and conferred neuroprotection against oxidative stress in all tested cell lines.

**Conclusions:** The study supports the use of omics-based strategies for drug repurposing in PD and highlights HDAC1 and PARP-1 inhibitors as promising neuroprotective candidates for further therapeutic development.

**Funding:** Interferencia Entre La Acetilación Y La Fosforilación En La Enfermedad De Alzheimer: Papel De Las Sirtuinas 3 Y 7 En Neurodegeneracion Olfatoria (PID2023-152593OB-I00)

**abstract citation id: bjaf055.074**

### Cystic fibrosis alters the structure of the olfactory epithelium and the expression of olfactory receptors affecting odor perception

*Ignacio Caballero[1], Yvon Mbouamboua[2], Susanne Weise[3], Raquel López-Gálvez[4], Marie Couralet[2], Marta Barrera-Conde[5], Matthieu Keller[4], Nikolai Klymiuk[6], Patricia Robledo[5], Thomas Hummel[3], Pascal Barbry[2], Pablo Chamero[4]*

*[1]Infectiologie et Santé Publique, UMR1282, INRAE, University of Tours, 37380 Nouzilly. France*
*[2]Université Côte d'Azur, CNRS, INSERM, Institut de Pharmacologie Moléculaire et Cellulaire, 06560 Sophia Antipolis. France*
*[3]Department of Otorhinolaryngology, Smell & Taste Clinic, Carl Gustav Carus University Hospital, Technische Universität Dresden, 01307 Dresden. Germany*
*[4]Laboratoire de Physiologie de la Reproduction et des Comportements, CNRS, INRAE, University of Tours, 37380 Nouzilly. France*
*[5]Integrative Pharmacology and Systems Neuroscience, Neuroscience Research Program, IMIM-Hospital del Mar Research Institute, 08003 Barcelona. Department of Medicine and Life Sciences, Universitat Pompeu Fabra, 08003 Barcelona. Spain*
*[6]First Department of Medicine, Cardiology, Klinikum Rechts der Isar, Technical University of Munich, School of Medicine and Health, Munich. Center for Innovative Medical Models, Ludwig Maximilian University of Munich, 81377 Munich. Germany*

A reduced sense of smell is a common condition in people with cystic fibrosis (CF) that negatively affects their quality of life. While often attributed to nasal mucosa inflammation, the underlying causes of the olfactory loss remain unknown. Here, we characterized gene expression in olfactory epithelium cells from patients with CF using single-nuclei RNA sequencing and found altered expression of olfactory receptors (ORs) and genes related to progenitor cell proliferation. We confirmed these findings in newborn, inflammation-free samples of a CF animal model and further identified ultrastructural alterations in the olfactory epithelium and bulbs of these animals. We established that CFTR, the anion channel whose dysfunction causes CF, is dispensable for odor-evoked signaling in sensory neurons, yet CF animals displayed defective odor-guided behaviors consistent with the morphological and molecular alterations. Our study highlights CF's major role in modulating epithelial structure and OR expression, shedding light on the mechanisms contributing to olfactory loss in CF.

**Funding:** This work was supported by the Agence National de la Recherche grant nos. ANR-20-CE92-0003 (to P.C.) and ANR-18-CE20-0024-01 (to I.C.), the Vaincre la Mucoviscidose Association grant nos. RF20200502689 (to I.C. and P.C.) and RF20180502280 (to P.B. and I.C.), the Region Centre Val de Loire grant no. 201900134883 (to P.C.), the Conseil Départemental des Alpes Maritimes grant no. 2016-294DGADSH-CV (to P.B.), the National Infrastructure France Génomique grant ANR-10-INBS-09-03 (to P.B.), and the France 2030 programme ANR-23-IAHU-0007 and ANR-19-P3IA-0002 (to P.B.).

**abstract citation id: bjaf055.075**

### AI IN OLFACTORY SCIENCE - BRIDGING CHEMISTRY, BIOLOGY, SEMANTICS, AND SENSOR TECHNOLOGY

### *Symposium Organizer: Antonella Di Pizio*

### AI and the structural landscape of odorant receptors

*Antonella Di Pizio[1]*

*[1]Leibniz Institutue for Food Systems Biology at TUM. Germany*

Odorant receptors (ORs), with ~400 members, constitute the largest subfamily of membrane proteins in our body. Experimental structures currently cover only a small fraction of the vast olfactory receptor family. AI-based methods, like AlphaFold 2 (Nobel Prize in Chemistry 2024), can predict protein structures with near-experimental accuracy [1]. By providing accurate structural models for virtually all members of the OR family, these approaches address long-standing gaps in our structural understanding, enabling new hypothesis-driven discoveries across olfactory research [2].

This talk will present AI-driven protein modeling protocols to characterize the structural landscape of ORs, with a focus on:

(i) mapping selective regions and motifs across the OR repertoire,

(ii) refining ligand-binding pockets to improve predictive accuracy [2], and
(iii) exploring conformational dynamics to uncover activation mechanisms.

These advances promise to accelerate the discovery of OR modulators and deepen insights into the chemistry and biology of olfaction.

### References

1 Jumper J, et al. (2021) Nature, 596 (7873), 583-589. Highly accurate protein structure prediction with AlphaFold.
2 Kogut-Günthel M, et al. (2025) British Journal of Pharmacology, 182 (14), 3225-3248. The path to the G protein-coupled receptor structural landscape: Major milestones and future directions.
3 Nicoli A, et al. (2023) J Chem Info Model, 63 (7), 2014-2029. Modeling the Orthosteric Binding Site of the G Protein-Coupled Odorant Receptor OR5K1.

**abstract citation id: bjaf055.076**

### Code2smell: a bio-inspired model of combinatorial code for predicting olfactory perception

*Jérémie Topin*[1]

[1]*Université Côte d'Azur. France*

This morning, you may have enjoyed a glass of orange juice and felt that its aroma had stimulated your sense of smell. Impressively, your nose is selectively sensitive to one enantiomer of the two possible form of the Limonene. Enantiospecific recognition is achieved by the olfactory receptors (ORs) expressed in your olfactory epithelium. Each odorant molecule activates a set of ORs, creating a representation that your brain eventually interprets as a perception, which we call smell. But among your 400 ORs, which ones is responsible for chiral discrimination?

To answer this question, and more generally to determine the molecular recognition spectrum of ORs, we design the Molecule to Olfactory Receptor M2OR database (https://m2or.chemsensim.fr/), which brings together 75,050 bioassay experiments for 51,683 distinct OR-molecule pairs.[1] We further combine protein language[2] with graph neural networks to predict OR activation, and propose a tailored architecture incorporating inductive biases from the protein-molecule interaction.[3] this model outperforms state-of-the-art drug-target interaction prediction models as well as standard GNN baselines. Notably, our predictions are in agreement with combinatorial coding theory in olfaction. Our results reveal consistent coding for a large number of odor families and the model suggests new insights such as previously unknown pairs of enantiomers with distinct combinatorial codes.

### References

1 Lalis, M., Hladiš, M., Khalil, S. A., Briand, L., Fiorucci, S., & Topin, J, 2024. M2OR: a database of olfactory receptor–odorant pairs for understanding the molecular mechanisms of olfaction. Nucleic Acids Research, 52(D1), D1370-D1379.
2 Elnaggar, A., Heinzinger, M., Dallago, C., Rehawi, G., Wang, Y., Jones, L., … & Rost, B. (2021). Prottrans: Toward understanding the language of life through self-supervised learning. IEEE transactions on pattern analysis and machine intelligence 2022, 44(10), 7112-7127.
3 Hladiš, M., Lalis, M., Fiorucci, S., & Topin, J. Matching receptor to odorant with protein language and graph neural networks 2023. In The Eleventh International Conference on Learning Representations.

**abstract citation id: bjaf055.077**

### Evaluating language models potential for capturing odor- perceptual and odor-semantic information

*Thomas Hörberg*[1], *Murathan Kurfalı*[2], *Jonas Kristoffer Olofsson*[1]

[1]*SCI-lab, Gösta Ekman Laboratories, Department of Psychology, Stockholm University. Sweden*
[2]*RISE Research Institutes of Sweden. Sweden*

In western languages, the vocabularies for odors are underdeveloped in comparison to those of other sensory domains. Terms that exclusively describe odor experiences (such as, e.g., color terms) are lacking. Instead, odors are described by reference to the source (*buttery*), intensity (*overpowering*), familiarity (*unmistakable*) or the hedonic evaluation (*pleasant*) of the odor, or with cross-modal metaphors (heavy). Odor vocabularies of western languages are thus vague and unspecific, making it challenging to construct lexicons for odor qualities. In recent years, many studies have used natural language processing (NLP) and artificial intelligence (AI) to map odor lexicons. In particular, language models (LMs) have been used to map the odor-semantic space of odor words. However, it is still unclear how effective LMs are in capturing odor-semantic relationships, what aspects of odor-semantic information they capture, and how their performance differs and depends on the training data.

We present results of a comprehensive performance evaluation of static word embedding models (e.g. Word2Vec), encoder- based models (BERT), and decoder-based LLMs (e.g. GPT-4o), under nearly 200 training configurations with different training data or prompting techniques. The models' performances were benchmarked against three odor datasets: odor similarity ratings, odor label similarity ratings, and odor-to-label ratings. Simpler static models perform best in capturing odor- perceptual similarities, while state-of-the-art encoder models (e.g. GPT-4o) excel in simulating odor-semantic relationships. The performance of static models is highly dependent on the quality of the training data: model performance is best when models are trained on odor-related texts. We conclude that natural language encodes latent information about human odor knowledge that is retrievable with text based LMs to varying degrees, and that there is potential of using LMs to map odor- perceptual and odor-semantic spaces.

**Funding:** This work was funded by the Swedish Research Council (2024-01506; 2021-03440; 2021-00178; 2020-00266), the Knut and Alice Wallenberg Foundation (2016:0229), and The Swedish e-Science Research Center.

**abstract citation id: bjaf055.078**

### Machine Learning for Molecular Sensing

*Gianaurelio Cuniberti*[1,2]

[1]*Institute for Materials Science and Max Bergmann Center for Biomaterials, TU Dresden, 01062 Dresden, Germany*
[2]*Dresden Center for Computational Materials Science (DCMS), TU Dresden, 01062 Dresden, Germany*

Olfaction, an ancient sensory system, provides intricate information about the environment. In emulation of this biological process, neuromorphic devices in conjunction with machine learning algorithms, endeavor to replicate and digitize the olfactory capabilities. This presentation focuses on the gas discrimination and identification capabilities of neuromorphic nanosensors. These nanosensors, constructed with functionalized nano materials, were integrated into multi-channel gas sensor devices, and their sensing signals were recorded upon exposure to diverse gases. To unravel the temporal characteristics embedded in the sensing signals, we employ machine learning algorithms to extract meaningful patterns and discern specific gases. The integration of machine learning significantly enhances the electronic olfaction system's gas identification performance across a wide spectrum of gases. This innovative platform not only downsizes electronic noses but also digitizes olfactory information, enabling the precise detection and identification of various gases and volatile organic compounds (VOCs). By leveraging machine learning, our electronic olfaction system demonstrates exceptional capabilities applicable to diverse fields such as pathogen detection, environmental monitoring, and disease diagnosis. The fusion of neuromorphic nanosensors and machine learning algorithms creates a powerful synergy, paving the way for advanced molecular sensing technologies with broad-ranging applications.

**abstract citation id: bjaf055.079**

### Through the Fermi hole of fragrance: machine learning the quantum signatures of smell

*Pinaki Saha*[1], *Sarabeshwar Balaji*[2], *Mrityunjay Sharma*[3], *Aryan Amit Barsinyan*[4], *Volker Steuber*[1], *Michael Schmuker*[1]

[1]*University of Hertfordshire. United Kingdom*
[2]*IISER Bhopal. India*
[3]*CSIR CSIO. India*
[4]*NIT Suratkal. India*

Olfaction is a profoundly complex sensory process involving multifaceted interactions between volatile organic compounds and olfactory receptors (ORs). Despite its centrality to both human perception and industrial applications, the scientific understanding of how molecular structures translate into odour remains incomplete. The difficulty stems from both the biological complexity of olfaction and the limitations in modelling the relationship between molecular features and perceptual outcomes[1]. Recent advances in machine learning have led to highly promising ligand-based methods. Notably, Lee et al. introduced a deep graph neural network (GNN) model that constructs a Principal Odour Map (POM) to encode odorant perceptual similarity[2]. Their model achieved state-of-the-art performance using an ensemble of 50 message passing GNNs (MPGNNs), reaching an AUROC of 0.894. We present here our ligand-based ML framework used for understanding olfaction: Dense-Sense. Dense Sense is a novel machine learning framework that integrates quantum chemistry data—specifically, electron density localization and delocalization matrices—into a GNN architecture for odour prediction. This model is trained to predict the odour labels for each molecule. The best-performing model, DMPN, achieved a validation AUROC of 0.871, exceeding the performance of traditional individual GNN model (0.864 AUROC). To further improve performance, we implemented ensemble methods. An ensemble of 10 DMPNN models yielded a validation AUROC of 0.88, effectively matching model reported by Lee et al. Dense-Sense thus demonstrates that QM data can significantly enrich machine learning models for olfaction.

### References

1 Sell, C.S., 2006. On the unpredictability of odor. Angewandte Chemie, 45(38), pp.6254-6261.
2 Lee, B.K. et al. 2023. A principal odor map unifies diverse tasks in olfactory perception. Science, 381(6661), pp.999-1006

**abstract citation id: bjaf055.080**

### Watching degree of deterioration of dry aged meat, by an electronic nose equipped with artificial intelligence

*Jose Iñaki Alava Marquinez*[1], *Lucia Lazaro De la Escalera*[1]

[1]*Basque Culinary Center -Mondragon University. Spain*

**Introduction:** The release of volatile compounds due to microbial activity is a valuable source of information about the condition of food. Since it can be linked to spoilage and affect quality, having simple, fast, and accurate methods makes them highly effective tools. This study focused on using an electronic nose ("Smell Inspector") with the aim of monitoring the degree of dry-aged meat development by measuring the release of putrescine.

**Material and Methods:** Putrescine (Apollo Scientific) in deionized water dilutions. For this purpose, a device composed of 64 carbon nanotube channels was used, through which a physicochemical change is recorded and translated into resistance data that are stored and processed on an artificial intelligence chip. Putrescine dilutions were prepared as a starting point to generate a data matrix. In parallel, dry-aged meat samples were prepared using the Dry method and stored in sealed Erlenmeyer flasks at 4°C for different durations. The measurements were repeated so that the device could learn to recognize the compound.

**Results:** The graph shows the detection of putrescine at different concentrations and at day 30 of meat aging; the goal is still to reach 60 days of maturation.

**Conclusions:** Therefore, it is concluded that the use of electronic noses as an inspection tool, complementary to sensory analysis, offers promising results. This work has been fully funded by the Basque Culinary Center.

### References

1 On the chirality-dependent adsorption behavior of volatile organic compounds on carbon nanotubes. Bin Li and Changsen Mi, Physical Chemistry Chemical Physic, ,Issue 38,2001,23, 21941-21950

2 Adsorption of volatile organic compounds onto carbon nanotubes,carbon nanofibers, and high-surface-area graphites. Eva Díaz, Salvador Ordóñez , Aurelio Vega, Journal of Colloid and Interface Science 305 (2007) 7–16

3 Characteristic Metabolic Changes of the Crust from Dry-Aged Beef Using 2D NMR Spectroscopy . Hyun Cheol Kim, Ki Ho Baek, Yoon-Joo Ko, Hyun Jung Lee,Dong-Gyun Yim and Cheorun Jo. Molecules 2020, 25(13), 3087; https://doi.org/10.3390/molecules25133087

4 Organic Thin-Film Transistor Based Gas Sensors for Putrescine Detection, Jiaxin Zhu, MSD Thesis, University of Waterlo, Ontario, Canada, 2018o

5 Evolution of Volatile Compounds and Spoilage Bacteria in Smoked Bacon during Refrigeration Using an E-Nose and GC-MS Combined with Partial Least Squares Regression Xinfu Li, Jiancai Zhu , Cong Li, Hua Ye , Zhouping Wang , Xiang Wu and Baocai Xu. Molecules 2018, 23, 3286; doi:10.3390/molecules23123286

**abstract citation id: bjaf055.081**

### Towards a Linear Framework for Predicting Olfactory Mixture Perception

*Robert Pellegrino[1], Jennifer Margolis[1], Carissa Evans[1], Matthew Andres[1], Emily J. Mayhew[1,2], Alexander B. Wiltschko[3], Richard C. Gerkin[3], Joel D. Mainland[1,4]*

*[1]Monell Chemical Senses Center, Philadelphia, PA 10104, United States*
*[2]Michigan State University, East Lansing, MI. United States*
*[3]Osmo; New York, NY, USA. United States*
*[4]Department of Neuroscience, University of Pennsylvania, Philadelphia, PA 19104, United States*

Recent models have shown that physical properties of individual molecules can be used to accurately predict perception, but predicting the perception of natural odors, which often consist of complex mixtures of chemicals, remains challenging. Anecdotal reports from perfumers suggest that odor quality may shift dramatically depending on the specific combination of odorants, even when individual components remain constant; however, linear models have performed well at predicting odor mixture similarity in human behavioral studies. This raises the question if odor mixtures exhibit linear behavior, with no emergent or nonlinear effects (also known as configural odors). To test this, we collected descriptive ratings for a large, diverse set of mixtures (N = 695) and their components (N = 171). Remarkably, all mixtures had qualities that could be explained by a linear combination of the components after accounting for session effects. Additionally, a linear model of eight previously reported configural mixtures (e.g., caramel + strawberry = pineapple) predicted odor perception with high accuracy (r = 0.82). We conclude that most odor mixtures fall within a region defined by linear mixing of their components, challenging prior assumptions in the field. This has broad implications for industries such as flavor and fragrance, as well as environmental monitoring and health.

**abstract citation id: bjaf055.082**

### STRUCTURAL ADVANCES IN CHEMOSENSORY RECEPTORS

### *Symposium Organizer: Masha Niv*

### Structural advances in chemosensory receptors

*Masha Niv[1]*

*[1]The Hebrew University. Israel*

Chemosensory receptors—central to perception of taste and smell—are at the forefront of structural biology. Recent breakthroughs in structural methods have opened an unprecedented window into how these receptors recognize ligands, initiate signaling, and evolve across species.Cryogenic electron microscopy (cryo-EM) has revolutionized the structural biology of membrane proteins, enabling the determination of an exceptional number of chemosensory receptor structures over the past year. These advances are further amplified by transformative AI-based protein structure prediction methods, recognized by the 2024 Nobel Prize in Chemistry, which continue to evolve at astonishing speed.This symposium explores the intersection of experimental and computational approaches that have led to new structural insights into olfactory and taste receptors. The talks will highlight the roles of cryo-EM, deep learning, and molecular dynamics (MD) simulations in bridging gaps between sequence, structure, and function—and in paving the way for rational design in sensory biology.

**abstract citation id: bjaf055.083**

### Bitter Taste Receptors: What Have We Learned by Combining Structural Biology and AI

*Masha Niv[1]*

*[1]The Hebrew University. Israel*

Vertebrate bitter taste receptors are a family of G protein–coupled receptors (GPCRs) called TAS2Rs. They are primarily expressed in taste receptor cells on the tongue, but are found also in various extraoral tissues (e.g., gut, airways, heart), where they may play roles in physiological processes such as metabolism, immunity, and smooth muscle contraction. TAS2Rs exhibit notable diversity in sequence, and their number varies across species. While some receptors can recognize chemically diverse ligands, others display narrow specificity[1]. To investigate the molecular recognition mechanisms of the broadly tuned human TAS2R14, we determined its cryo-EM structure in complex with flufenamic acid. Remarkably, two copies of the ligand were observed, occupying two distinct binding sites[2]. The functional relevance of each site was evaluated through site-directed mutagenesis and functional assays, revealing differential contributions of these binding pockets depending on the ligand. To examine whether a second binding site is a common feature among TAS2Rs, and to computationally map multiple bitter compounds[3] onto TAS2Rs from different species, we evaluated and refined several AI-based prediction approaches. Our findings underscore the value of integrating structural and functional experiments with AI-driven modeling, and reveal subtype-specific features of TAS2Rs that are crucial for rational modulation of bitter taste signaling.

### References

1. Di Pizio A, Niv MY. Promiscuity and selectivity of bitter molecules and their receptors. 10.1016/j.bmc.2015.04.025.
2. Peri L, Matzov D, Huxley DR, et al. A bitter anti-inflammatory drug binds at two distinct sites of a human bitter taste GPCR. 10.1038/s41467-024-54157-6.
3. Ziaikin, E, et al BitterDB: 2024 update on bitter ligands and taste receptors, 10.1093/nar/gkae1044

**abstract citation id: bjaf055.084**

### Molecular simulations assess the structure-based design of GPCR chemical modulators - methods and success stories

*Hugo Gutiérrez de Terán Castañón[1]*

*[1]CSIC (Spanish National Research Council) - CINN (Research Center in Nanomaterials and Nanotechnology). Spain*

In my group we have been routinely assisting for the last decade GPCR ligand design with MD simulations and Free energy perturbation (FEP) protocols, with reported success in the optimization of GPCR orthosteric ligands [1] Importantly for this forum, our main drug discovery projects have been entirely performed on the basis of homology models of yet unresolved structures of the target GPCR [2]. For one of those, on A2B adenosine receptor antagonists, the recent release of two contradictory cryo-EM structures in complex with came with controversial experimental proposals on the binding mode of a partial agonist, which we solved using our original combination of ligand- and residue-FEP analysis of SAR and mutagenesis data, developed along a pharmaceutical collaborative project [3]. With a similar approach, we also contributed to validate the binding mode of surrogate agonists of an orphan receptor [4].

We will also discuss our original map of the GPCR pharmacological equilibrium through thermodynamic cycles, which allowed to modulate the pharmacological profile of GPCR ligands and to elucidate of the mechanism of CIM and CAM mutations [5].

Given the maturity of the structural characterization, with recent cryo-EM structures and high-quality models available via AI-based modeling, we believe that our computational pipelines are especially attractive for the chemosensory receptor community.

**Funding:** Grant PID2023- 150793OB-I00 from the Spanish Ministry of Science and Innovation – State Research Agency – FEDER-UE

### References

1 (a) van der Broek et al. and Gutiérrez-de-Terán, Bioinformatics, 2024, btae66 (b) Jespers et al. and Gutiérrez-de-Terán, J. Cheminf, 2019, 11:26

2 (a) Majellaro et al., J. Med. Chem., 2021, 64, 658 (b) Prieto et al., J Immunother. Cancer, 2022, 10:e004592 (c) Xu et al., Mol Pharmacol, 2018, 93, 323.

3 (a) Tandarić and Gutiérrez-de-Terán. J. Phys. Chem. B, 2025, 119:886 (b) Jespers et al. and Gutiérrez-de-Terán, Ang. Chem Int Ed, 2020, 59:16536

4 Nøhr et al. Sci Rep. (2017) 7:1128

5 Jespers et al. and Gutiérrez-de-Terán. PLoS Comp. Biol., 2021, 17: e1009152.

**abstract citation id: bjaf055.085**

### Structural Insights into How Mammals Discriminate Odorants

*Hiroaki Matsunami[1]*

*[1]Duke University. United States*

The olfactory system discriminates an enormous variety of odors through distinct activation patterns of odorant receptors (ORs), with each pattern encoding specific odor identities. Although mammalian G-protein coupled ORs were discovered over 30 years ago, the precise mechanisms underlying their recognition abilities have only begun to be understood in recent years. The OR family includes Class I ORs, which are generally sensitive to carboxylic acids, and Class II ORs, which respond to a broader range of odorants and constitute the majority of mammalian ORs.

We and others have analyzed the active-state structures of Class I ORs bound to carboxylic acids, providing insights into their ligand interactions. To investigate Class II ORs, we used a consensus protein design strategy to engineer receptors representing specific subfamilies by incorporating the most frequent amino acid at each position. This approach enabled efficient expression and purification in heterologous systems and allowed us to determine cryo-EM structures for three Class II ORs. These structures reveal their unique ligand recognition properties and highlight both shared and distinct features of their odorant-binding pockets compared to Class I ORs.

Together with functional analyses of point mutants and computational simulations, our studies provide a framework for understanding the molecular basis of odorant recognition across diverse ORs, offering new insights into how the olfactory system decodes chemical diversity.

abstract citation id: bjaf055.086

### Conformational landscape of the entire odorant receptor repertoire

*Alessandro Nicoli[1], Florian Bößl[1], Alejandro Peralta-García[2], Toni Giorgino[3], Jana Selent[2], Antonella Di Pizio[1]*

*[1]Leibniz Institute for Food Systems Biology at the Technical University of Munich. Germany*
*[2]Hospital del Mar Research Institute & Universitat Pompeu Fabra. Spain*
*[3]Biophysics Institute (CNR-IBF), National Research Council of Italy. Italy*

With approximately 400 coding genes, odorant receptors (ORs) represent about 2% of all protein-coding genes and the most numerous membrane proteins in our body[1]. However, ORs are poorly characterised in terms of structure and cognate ligands, with more than 88% of ORs still orphan, which hinders our understanding of their physiological role[2].

Recent breakthroughs in AI for protein structure prediction have led to the generation and sharing of all OR model structures in the AlphaFold database[3]. Recently, the first cryo-EM structures of ORs were published, revealing the impressive ability of AlphaFold 2 (AF2) to accurately predict the structural architecture of this class of receptors[4]. In our previous work, we found that the residue conformations need to be optimised to be used for predicting ligand-receptor interactions[5]. Therefore, we hypothesize that, when conformational sampling is applied to AF modeling, we can improve OR structure performance for ligand identification.

In my presentation, I will introduce the first and largest structural dataset of refined OR models. To achieve this, we used state-of-the-art high-throughput molecular dynamics simulations based on receptor models in the different activation states. The receptor models remained stable throughout the simulations and proved new insight into the flexibility of the OR structural domain, especially for the transmembrane 6 and its continuous region, the extracellular loop 3. The preliminary results pinpoint intriguing plasticity of odorant receptors and suggest a peculiar activation mechanism compared to other G protein-coupled receptors.

With a total simulations time of 2.3 ms, we sampled the conformational space of the entire OR repertoire. The availability of OR structural information will enable unprecedented class-wide structural analysis and deorphanisation campaigns, thereby enhancing our understanding of the molecular coding of olfaction.

### References

1 L. Buck, R. Axel. A novel multigene family may encode odorant receptors: a molecular basis for odor recognition. Cell 65, 175–187 (1991).
2 M. M. Scharf, L. J. Humphrys, S. Berndt et al. The dark sides of the GPCR tree – research progress on understudied GPCRs. British Journal of Pharmacology (2024).
3 J. Jumper, R. Evans, A. Pritzel et al. Highly accurate protein structure prediction with AlphaFold. Nature 596, 583–589 (2021).
4 C. De March, N. Ma, C. Billesbølle et al. Engineered odorant receptors illuminate the basis of odour discrimination. Nature 635, 499–508 (2024).
5 A. Nicoli, F. Haag, P. Marcinek et al. Modeling the orthosteric binding site of the G protein-coupled odorant receptor OR5K1. Journal of Chemical Information and Modeling 63, 2014–2029 (2023).

abstract citation id: bjaf055.087

### Optimizing odorant receptor stability and expression

*Mathis Puech[1], Bernard Offmann[2], Ewen Lescop[3], Claire de March[3]*

*[1]CNRS. France*
*[2]Unité en Sciences Biologiques et Biotechnologiques, Nantes Université, Nantes, France*
*[3]Institut de Chimie des Substances Naturelles, CNRS 2301, Université Paris Saclay, Gif-sur-Yvette, France*

Human olfaction relies on 400 odorant receptors (ORs). Despite belonging to the well-studied class of GPCRs, knowledge about ORs remains limited due to the complexity of their in vitro study. Among the 400 members of the human OR family, only a few are efficiently expressed on the surface of common cell line models, leading to a poor understanding of their mechanisms and pharmacology. In this project, we hypothesize that ORs lack cell surface expression due to the poor stability of their structure in the membrane of cell models. We aim to enhance OR cell surface expression by modifying their amino acid sequences, allowing us to better understand the role of each residue in OR structure stabilization. These modifications are guided by computational prediction and validated in vitro through site-directed mutagenesis. Flow cytometry provides feedback to our computational methods, allowing us to evaluate and improve the accuracy of our predictions. This project could enable the expression of ORs that are currently poorly or not expressed at all in our laboratory cell models by applying a limited number of mutations, broadening the repertoire of ORs that can be functionally studied.

This work could also lead to the elucidation of OR structures, their purification, and further functional studies. This research serves as a gateway to unravelling the molecular mechanisms involved in olfaction.

### Reference

1 de March, et al. Nature (2024)
2 Ikegami, de March, et al. PNAS (2020)
3 Ghosh, de March, et al. Biophysical Society (2022)
4 Zhuang, et al. Nature Protocols (2008)

abstract citation id: bjaf055.088

### An updated phylogenetic classification of the human olfactory receptor repertoire

*Sylvester Holt[1], Selma Valling Lauritsen[1], Vandana Kaushal[1]*

[1]*University of Copenhagen - Department of Food Science Section of Food Design and Consumer Behavior. Denmark.*

We present a comprehensive phylogenetic and functional analysis of 404 human olfactory receptors (ORs), clustered into 16 clades using maximum likelihood and average distance (AD $\geq$ 0.6, corresponding to $\geq$40% average sequence identity) thresholds. These clades reveal distinct patterns in chromosomal localization, conserved structural motifs, ligand specificity, and ectopic expression. Ligand-receptor data across over 1000 pairs show clade-specific preferences for chemical superclasses, particularly benzenoids and lipid-like molecules. Principal component analysis confirmed functional clustering, with several receptors displaying broad tuning, including OR1D2 and OR2W1. Conserved motifs such as MAYDRYVAIC, PMYFFL, and dibasic c-terminal sequences varied across clades, and specific substitutions were linked to altered ligand affinity and receptor function. Clade 1, for instance, features leucine substitution at position F6.34 and a reduced presence of the dibasic motif, correlating with higher EC50 values. Ectopically expressed ORs appeared in nearly all clades and showed no consistent sequence similarity. Class I receptors (Clades 14–16) retained hallmark motifs and primarily bound carboxylic acids. Our classification aligns with known families but offers refined insights into evolutionary relationships and structure–function coupling. This study provides a detailed framework for future investigations into OR function, ligand binding specificity, and potential extra-nasal roles.

**abstract citation id: bjaf055.089**

### SCENTED EMOTIONS: THE ROLE OF EMOTIONAL HUMAN CHEMOSIGNALS IN SOCIAL AND EMOTIONAL EXCHANGE

### *Symposium Organizer: Cinzia Cecchetto*

### Scented emotions: the role of emotional human chemosignals in social and emotional exchange

*Cinzia Cecchetto*[1]

[1]*University of Padova. Italy*

Body odors (BOs) serve as carriers of chemical signals that transmit social information from those around us. Research has shown that the chemical composition of BOs emitted during specific emotional states can trigger an 'emotional contagion' in others, leading to a partial replication of the sender's affective state. This phenomenon has been observed for both positive and negative emotions, such as happiness, fear, anxiety, aggression, and disgust. Growing evidence suggests that fear- related BOs, in particular, can have a profound impact on those who perceive them. The proposed symposium aims to present the latest findings from the POTION Consortium, funded by the EU Horizon 2020 program, on the influence of both positive and negative human chemosignals on social interactions. Beyond these results, we will explore new perspectives and future directions.

**abstract citation id: bjaf055.090**

### Neural insights into emotional body odors processing in affective disorders

*Elisa Dal Bò*[1], *Cinzia Cecchetto*[2], *Alejandro Luis Callara*[3], *Saideh Ferdowski*[4], *Alberto Greco*[3], *Luca Citi*[5], *Enzo Pasquale Scilingo*[3], *Claudio Gentili*[2]

[1]*University of Padova. Italy*
[2]*Department of General Psychology, University of Padova. Italy*
[3]*Research Centre "E. Piaggio", Department of Information Engineering, School of Engineering, University of Pisa. Italy*
[4]*School of Mathematics, Statistics and Actuarial Science, University of Essex. United Kingdom*
[5]*School of Computer Science and Electronic Engineering, University of Essex. United Kingdom*

Affective disorders, such as depression and social anxiety, are common conditions often marked by impaired social functioning. While research on social motivation has mainly focused on visual and auditory cues, recent attention has turned to olfaction. Humans can transmit socially relevant and emotional information, such as health, mood, or personality traits, through body odor (BO). Given the unique advantages of chemosensory communication, studying emotional BOs may help clarify the role of social signals in the development and maintenance of affective disorders. However, this area remains underexplored. To investigate the diagnostic potential of chemosensory signals, we examined how BOs collected in happiness- and fear-eliciting conditions modulate the subjective ratings, psychophysiological responses, and neural processing of neutral faces in individuals with depressive symptoms, social anxiety symptoms, and healthy controls ($n = 22$ per group). In this talk, I will present the temporal dynamics of the neural processing of the stimuli, showing that both happiness and fear BOs acted in a similar vein in enhancing the vagal tone of the participants and in modulating the neural processing of ambiguous social visual stimuli. Lastly, I will present a dynamic causal modeling (DCM) analysis aimed at characterising the effective connectivity between brain regions involved in emotional and social information processing. This approach can provide further insights into how emotional chemosignals shape brain network dynamics in affective disorders. With the results of this study, I aim to provide a psychophysiological framework for the role of both happiness and fear BOs in the development and course of affective disorders.

**abstract citation id: bjaf055.091**

### Spatio-temporal independent component analysis of sweat volatile organic compounds for the identification of potential human fear chemosignals: the POTION framework

*Alberto Greco*[1], *Alejandro Luis Callara*[1], *Tobias Bruderer*[2], *Matyas Ripszam*[2], *Andrea Gargano*[1], *Fabio Di Francesco*[2], *Enzo Pasquale Scilingo*[1]

[1]*Research Center "E. Piaggio". University of Pisa, 56122 Pisa, Italy*
[2]*Department of Chemistry and Industrial Chemistry, University of Pisa, 56124 Pisa, Italy*

Within the H2020 POTION project (Promoting Social Interaction through Emotional Body Odours), we developed an experimental framework to identify fear-related chemosignals in human sweat. Participants underwent a fear-inducing virtual reality scenario while sweat volatiles were continuously sampled with PTR-TOF mass spectrometry. Simultaneously, autonomic reactivity was monitored through electrodermal activity (EDA) and heart rate variability (HRV).

We applied two complementary Independent Component Analyses (ICAs): (i) a group-level ICA in the chemical domain to derive candidate "fear fingerprints" based on combinations of volatiles, and (ii) subject-specific temporal ICAs clustered by similarity of chemical loadings, providing candidate "temporal signatures" of fear.

To test their relevance, we first derived a continuous fear index from physiological signals (Electrodermal activity, EDA, and Heart Rate Variability, HRV) following validated approaches. We then employed a Bayesian hierarchical elastic net regression with autoregressive errors, linking ICAs to the fear index while accounting for within-subject temporal structure and multicollinearity across components. This sparse modelling strategy identified subsets of chemical and temporal ICs credibly associated with fear dynamics.

Results showed that both chemical fingerprints and temporal signatures contained ICA-derived components significantly predicting the physiological fear index. These components were characterized by chemically heterogeneous combinations of volatiles, suggesting that potential human fear chemosignals may not be attributable to single molecules but to complex mixtures. Further studies will assess whether these mixtures can convey information about the emitter's psychophysiological state.

**abstract citation id: bjaf055.092**

### Decoding social interaction: the role of emotional body odours in group dynamics

*Maria Eleonora Minissi[1], Lily Cheng[2], Sergio Cervera Torres[1,3], Mariano Alcañiz[1], Luca Citi[2]*

[1]*Institute for Research on Human-Centered Technology (HUMAN-Tech), Polytechnic University of Valencia. Spain*
[2]*School of Computer Science and Electronic Engineering, University of Essex, Colchester, UK. United Kingdom*
[3]*Dpto. de Psicología, Universidad de Castilla-La Mancha, Albacete, Spain*

Social interactions are influenced by multiple factors, yet the impact of chemosignals conveyed by emotional human body odours (HBOs) on group dynamics remains poorly understood. This study investigates the effect of emotional HBOs on vicarious ostracism, which is a psychological phenomenon in which observers perceive and react to the social exclusion of others within a group.

A hyper-realistic virtual reality (VR) adaptation of the cyberball task, that is a validated paradigm for inducing vicarious ostracism, was used. In the task, four players are present: three automated characters and the participant. During each game round, one character is excluded by the other two at three different levels of salience, ranging from subtle to overt. Participants are unaware of the exclusion occurring among the characters.

Seventy Spanish adults, screened for the absence of depressive and social anxiety symptoms, as well as for common olfactory ability, took part in a 3×3 within-subject experimental design. They experienced the three salience levels of the VR game and, within each, were exposed to three odour conditions: synthesized HBOs of fear and neutral emotional states, and clean air.

Behavioural findings suggest that exposure to fear HBO significantly alters both the perception of, and reaction to, social exclusion compared to clean air and neutral HBO. Specifically, fear HBO appeared to increase participants' ability to detect social exclusion, even in an unconscious manner, and intensified behavioural responses toward the excluded player: individuals who compensated for the exclusion did so more strongly under fear HBO, while those who conformed exhibited greater conformity. These results offer novel insights into the role of fear-related chemosignals in shaping group behaviour during social exclusion and pave the way for further research into how emotional olfactory cues may influence social dynamics.

**Funding:** The study was funded by the EU Horizon 2020 POTION Project (ID 824153).

**abstract citation id: bjaf055.093**

### Testing human and artificial chemosignals in mindfulness-based interventions for social anxiety

*Elisa Vigna[1], Emma Eliasson[1], Vladimir Carli[1]*

[1]*National Centre for Suicide Research and Prevention of Mental Ill-Health (NASP), Karolinska Institutet. Sweden*

Drawing from the theory of emotional contagion, the clinical application of the POTION[1] system explored the potential of enhancing accessible, self-administered interventions using human chemosignals as treatment catalysts. This presentation highlights two consecutive randomized control studies investigating whether exposure to emotional chemosignals—human body odours (BOs) in the first study and artificial chemosignals in the second—could enhance mindfulness-based interventions for social anxiety.

In the first study, 98 women aged 18–35, meeting criteria for Social Anxiety Disorder (SAD), were randomly assigned to one of four conditions: fear BO, happiness BO, neutral BO, or clean air (control). BOs were previously collected from healthy male and female volunteers. Participants engaged in two mindfulness sessions over two consecutive days while exposed to their assigned odour. Changes in state anxiety (primary outcome) were measured before and after each session and analysed via mixed models. Although no significant odour × time interaction was found, effect size estimates suggested a greater reduction in state anxiety for the fear BO group (Day 1: Cohen's d = 0.26; Day 2: d = 0.54) compared to the control group. Moreover, participants exposed to BOs rated the mindfulness sessions as significantly more helpful than those in the clean air group (p = .002).

The second study assessed whether artificial chemosignals—synthetic analogues of fear BO, which had shown the strongest effects—could replicate these results. Using a similar design and a new sample of 120 women meeting the same inclusion criteria, the study found that both artificial chemosignal and clean air groups experienced symptom reductions, suggesting that mindfulness itself was the primary driver of improvement.

These findings highlight the potential of human chemosignals to enhance psychological interventions, while also underscoring the challenges of replicating such effects with artificial compounds.

**Funding:** This research was funded by the European Union's Horizon 2020 research and innovation programme under grant agreement No. 824153, project title: "POTION – Promoting Social Interaction through Emotional Body Odours" (2019–2024). https://doi.org/10.3030/824153

**abstract citation id: bjaf055.094**

### State-dependent variation of human body odors: from molecular changes to perception

*Antonie Bierling[1], Natan Horacek[2], Alexander Croy[1], Pavlina Kyjakova[2], Helene Loos[3], Annkatrin Wunder[3], Robert Hanus[2], Ilona Croy[1]*

*[1]Friedrich Schiller University Jena. Germany*
*[2]Academy of Sciences of the Czech Republic*
*[3]Friedrich-Alexander-Universität Erlangen-Nürnberg. Germany*

Body odors play an important role in social communication. For example, they influence sexual attraction, create a sense of belonging in the family, or allow us to infer emotions such as fear or happiness in other people. Still, most research on nonverbal communication is based on face perception, gesture, or voice. With this study we aim at characterizing the state- dependent variation of healthy human body odors and their perception. To this end, axillary sweat was sampled from 40 young male donors in four different conditions (exercise, stress, sexual arousal, control) and the pooled samples were rated on six visual analogue scales and a newly developed multiple choice description matrix of qualitative descriptors by 139 normosmic perceivers in two perception studies. Furthermore, the samples were analyzed for their chemical components using gas chromatography analysis in pools as well as individually. Our findings show a consistent pattern of state- dependent shifts in perception and chemical composition of body odors. Body odors emitted during stress and exercise were perceived as more intense, less pleasant, and were described using a greater number of negatively connoted qualitative labels, such as "rotten," "pungent," and "biting," compared to those from the control condition. Furthermore, donors exhibiting larger perceptual shifts from control to other states were also associated with greater changes in chemical composition. In conclusion, our work demonstrates that natural variations in physiological states are reflected in the chemical profile of body emissions and that these subtle changes can be detected by others, influencing nonverbal communication among humans.

**Funding:** This research has been funded by the European Union via "HORIZON-EIC-2021-PATHFINDEROPEN-01" (grant no. 101046369). The funders have/had no role in study design, data collection and analysis, decision to publish or preparation of the manuscript.

**abstract citation id: bjaf055.095**

### OLFACTORY FUNCTION IN MENTAL AND NEURODEVELOPMENTAL DISORDERS

### *Symposium Organizer: Maria Geraldine Veldhuize*

### In non-clinical depression in undergraduates, odor-evoked autobiographical memories are relatively less vivid than those evoked with words or photographs

*Berçem Yar[1], Yağmur Üngüder[1], Maria Veldhuizen[1]*

*[1]Mersin University. Turkey*

Autobiographical memory (AM) involves remembering our personal past together with the recollection of the self. The Proust phenomenon assumes that odors are particularly evocative reminders of past experiences. Previous work focusing on the experiential qualities of AM demonstrated that memories evoked by odors differ from memories evoked by words and photographs Understanding how different sensory modalities affect the recall of AM may provide insight into the relationship between depression and memory processes. We aimed to investigate differences in memory retrieval in depression using cues from different sensory modalities. Here we measured depressive symptom severity in undergraduate students (n = 99, 58 women, 19-41 years, M = 22.97, SD = 4.42) and used word, photograph, or odor cues to examine memory retrieval. In each of the three conditions, participants were presented with cues corresponding to 10 objects in a random order (odors: the actual scented objects, photographs: color images, words: the names of the objects displayed on a computer screen).

After the presentation of the cue, the participant was asked if any specific memory was evoked by the cue. If a memory was successfully retrieved, the subject was asked to write their memory as detailed as possible, and rated the evoked memory based on its experiential characteristics. Overall, we observed fewer memories recalled in those with depression relative to those without depression, mostly driven by the word and photograph cues. We observed no effect of cue type or depression group on AM specificity. Lower memory vividness was also associated with depression, but mostly in memories evoked by odors. These results suggest that odor cues can be used to examine effects of depression on autobiographical memories other than overgenerality of memory.

**Funding:** This project is funded by the TÜBİTAK 2224-A program.

**abstract citation id: bjaf055.096**

### Differential effects of odors on reward-seeking behavior in individuals with anorexia nervosa

*Androula Savva[1], Marc Guitart-Masip[2], Ata Ghaderi[1], Cynthia M. Bulik[3], Janina Seubert[1]*

*[1]Department of Clinical Neuroscience, Psychology Division, Karolinska Institutet, Stockholm. Sweden*
*[2]Aging Research Center, Department of Neurobiology, Care Sciences and Society, Karolinska Institutet, Stockholm. Sweden*
*[3]Department of Psychiatry, University of North Carolina at Chapel Hill, Chapel Hill, NC. United States*

Anorexia nervosa (AN) is a severe psychiatric disorder with a 5% mortality rate, characterised by persistent restriction of food intake and dangerously low body weight. Disturbances in reward processing, particularly in anticipatory responses to food cues, are thought to contribute to this behavior, but findings regarding the nature and direction of these changes remain inconsistent. Odors in particular are powerful modulators of food-related motivation, yet their impact on anticipatory reward responses in AN is unclear. Building on our prior work demonstrating that food odors can modulate reward-seeking behavior under hunger in healthy individuals, we examined whether similar effects occur in individuals with AN. Eighteen individuals with AN underwent fMRI after a 10-hour overnight fast while completing a food incentive delay paradigm. On each trial, a food or non-food odor cue was presented, followed by a visual symbol. Participants were instructed to press a button as quickly as possible in response to the symbol presentation. Trials were either rewarded or non-rewarded, and participants could earn monetary rewards based on their performance. Preliminary univariate analyses revealed bilateral insula and piriform cortex activation for food odors, while non-food odors elicited activation only in the piriform cortex, indicating that insular responses were specifically driven by food odors. These findings provide initial evidence for distinct neural processing of food versus non-food odors in AN and have potential implications for understanding how sensory cues modulate reward anticipation in this disorder.

**Funding:** VR2022-02239, ERC StG 947886 (JS), VR538-2013-8864 (CB), VR2018-02606 (MG)

**abstract citation id: bjaf055.097**

### Olfactory function in autism spectrum disorder: a meta-analytic perspective with new insights from chemosensory profiling and neural data

*Isabelle Cullen[1], Valentina Parma[1]*

*[1]Monell Chemical Senses Center. United States*

Sensory sensitivities are a core feature of autism spectrum disorder (ASD), but olfactory dysfunction remains less studied than other modalities in ASD[1.] We aimed to quantify the prevalence of olfactory dysfunction in ASD and characterize its neural correlates. A meta-analysis of published studies in children and adults compared prevalence estimates across identification, intensity, and hedonic domains, while considering demographic moderators (age, sex) and assessment method (self-report vs. psychophysical testing). Results showed significantly higher prevalence of olfactory dysfunction in ASD versus neurotypical controls, moderated by age and method. To address gaps in adult data, we collected new evidence from adults with ASD and controls, using the Glasgow Sensory Questionnaire[2] and structural and functional MRI. Adults with ASD reported elevated olfactory hypersensitivity and hyposensitivity ($U=1149$, $p<1e\text{-}9$). Structural MRI showed decreased cortical thickness in inferior frontal gyrus and altered cerebellar volume, correlating with odor identification accuracy. Diffusion tensor imaging revealed lower fractional anisotropy in superior and inferior longitudinal fasciculi ($r=0.41$–$0.52$), predicting sensory sensitivity scores. Functional MRI indicated broader cortical activation during odor processing despite intact discrimination of familiar body odors. These findings, both self-reported and imaging data, support predictive coding models proposing atypical weighting of sensory precision in ASD. Together, our results highlight olfaction as a relevant domain for understanding sensory processing differences and their neural substrates in autism.

### References

1 American Psychiatric Association. (2022). Diagnostic and Statistical Manual of Mental Disorders. Diagnostic and Statistical Manual of Mental Disorders.
2 Robertson, A. E., & Simmons, D. R. (2013). The relationship between sensory sensitivity and autistic traits in the general population. https://doi.org/10.1007/s10803

**abstract citation id: bjaf055.098**

### SENSORY ALTERATIONS IN PATIENTS WITH CANCER: FROM FUNDAMENTAL INSIGHTS TO CLINICAL SOLUTIONS

### *Symposium Organizer: Sanne Boesveldt*

### Sensory alterations in patients with cancer: from fundamental insights to clinical solutions

*Sanne Boesveldt[1]*

*[1]Wageningen University. The Netherlands*

Cancer treatments, while crucial in the fight against malignancies, often come with significant side effects that can severely impact patients' quality of life. Among the most distressing are sensory alterations, particularly in taste and smell, which are commonly reported by patients undergoing chemotherapy, radiotherapy, and immunotherapy. Additionally, side effects on somatosensation, which comprises perception towards texture, temperature, and chemesthetic sensations, may occur prior to, during, and following cancer treatments. These changes can lead to food aversions, making eating a challenging and unpleasant experience, culminating in nutritional problems. This symposium aims to provide a platform to discuss the latest research on sensory alterations in patients with cancer. Through a multi-disciplinary approach, we seek to bridge the gap between basic research and clinical application, offering new insights and potential solutions for improving the management of these challenging symptoms in cancer care.

**abstract citation id: bjaf055.099**

### Oral somatosensory perception of cancer patients: variability and influence on eating experience

*Reisya Riantiningtyas[1], Florence Carrouel[2], Amandine Bruyas[3], Wender L.P. Bredie[4], Camille Kwiecien[5], Agnès Giboreau[1], Anestis Dougkas[1]*

[1]*Institut Lyfe Research Centre. 69130 Ecully, France*
[2]*Health Systemic Process (P2S), Research Unit UR4129, University Claude Bernard Lyon 1, University of Lyon, 69008 Lyon. France*
[3]*Institute of Cancerology, Hospices Civils de Lyon, Hôpital Croix Rousse, 69004 Lyon, France*
[4]*Section for Food Design and Consumer Behavior, Department of Food Science, Faculty of Science, University of Copenhagen, 1958 Frederiksberg C, Denmark*
[5]*Danone Nutricia Research, 3584 CT Utrecht, The Netherlands*

Malnutrition is prevalent among cancer patients, often linked to altered food perception, including taste and smell (Drareni et al. 2019). However, food perception also includes somatosensation (texture, temperature and chemesthetic sensations), which has been less studied (Riantiningtyas et al. 2023). This work aimed to explore somatosensory perception in cancer patients and its link with eating behaviour through two complementary studies.

Study 1: A cross-sectional study assessed somatosensory perception and salivary function in head and neck cancer (HNC) patients (n=30) and healthy controls (n=30). Tests included lingual tactile sensitivity, food texture perception, chemesthetic perception, and thermal sensitivity. HNC patients had significantly reduced chemesthetic ($p<0.05$) and thermal sensitivity ($p=0.038$), as well as impaired salivary function ($p=0.001$). They also showed reduced ability to discriminate food texture, including roughness ($p=0.003$) and firmness ($p=0.025$).

Study 2: A survey of cancer patients (n=100) assessed self-reported sensory perception, oral symptoms, food preferences, and eating difficulties. Hierarchical clustering identified three sensory profile groups: no alteration (48%), increased perception (44%), and decreased perception (8%). Sensory alterations (somatosensory: $r=0.54$, $p<0.001$; chemosensory: $r=0.48$, $p<0.001$) and oral symptoms (food processing issues: $r=0.55$, $p<0.001$; discomfort: $r=0.44$, $p<0.001$) were correlated with eating difficulties. More severe sensory alterations and oral symptoms were linked to greater eating challenges.

These findings highlight that somatosensory and oral changes were linked to changes in food preferences and eating behaviour in cancer patients. They also emphasize the heterogeneity of sensory alterations within this population. This underscores the importance of considering somatosensation and oral comfort, in addition to taste and smell, when developing dietary solutions for cancer patients.

### References

1 Drareni K., Bensafi M., Giboreau A., Dougkas A., (2020), Chemotherapy-induced taste and smell changes influence food perception in cancer patients, Supportive Care in Cancer, 2125-2132, 1-8. https://doi.org/10.1007/s00520-020-05717-1

2 Riantiningtyas, R.R.; Carrouel, F.; Bruyas, A.; Bredie, W.L.P; Kwiecien, C.; Giboreau, A.; Dougkas, A. (2023) Oral Somatosensory Alterations in Head and Neck Cancer Patients—An Overview of the Evidence and Causes. Cancers, 15, 718. https://doi.org/10.3390/cancers15030718

**abstract citation id: bjaf055.100**

### Drivers of nutritional disorders in patients with cancer

*Meropi Kontogianni[1]*

[1]*Harokopio University, Dep. of Nutrition & Dietetics. Greece*

Nutritional disorders, namely malnutrition, sarcopenia and sarcopenic obesity in cancer patients arise from a complex interplay of tumor-related, treatment-related, and host-related factors. Tumors can induce systemic inflammation and metabolic alterations, including increased energy expenditure and protein catabolism, which contribute to cancer cachexia. Additionally, gastrointestinal tumors often impair food intake directly through obstruction or indirectly via symptoms such as nausea, early satiety, or dysphagia. Cancer therapies—particularly chemotherapy and radiotherapy—can exacerbate undernutrition by causing mucositis, anorexia, taste changes, and gastrointestinal side effects, whereas surgeries often performed for cancer treatment may cause net changes in the physiology of food intake or assimilation. Studies consistently show that nutritional disorders in patients with cancer undergoing treatment are associated with poorer survival, reduced treatment tolerance, and diminished quality of life. Despite the high prevalence of nutritional disorders in patients with cancer, nutritional risk screening, assessment and support remain underutilized in clinical practice, emphasizing the need for early screening and integrated nutritional care.

### References

1 Andreyev HJ, Norman AR, Oates J, Cunningham D. Why do patients with weight loss have a worse outcome when undergoing chemotherapy for gastrointestinal malignancies? Eur J Cancer 1998; 34(4): 503–509.

2 Di Fiore A, Lecleire S, Gangloff A et al. Impact of nutritional parameter variations during definitive chemoradiotherapy in locally advanced oesophageal cancer. Dig Liver Dis 2014; 46(3): 270–275.

3 Gyan E, Raynard B, Durand JP, Lacau Saint Guily J, Gouy S, Movschin ML, Khemissa F, Flori N, Oziel-Taieb S, Bannier Braticevic C, Zeanandin G, Hebert C, Savinelli F, Goldwasser F, Hébuterne X; NutriCancer2012 Investigator Group. Malnutrition in Patients With Cancer: Comparison of Perceptions by Patients, Relatives, and Physicians-Results of the NutriCancer2012 Study. JPEN J Parenter Enteral Nutr. 2018 Jan;42(1):255-260

4 He´buterne X, Lemarie´ E, Michallet M et al. Prevalence of malnutrition and current use of nutrition support in patients with cancer. JPEN J Parenter Enteral Nutr 2014; 38(2): 196–204.

5 Laviano A, Meguid MM. Nutritional issues in cancer management. Nutrition 1996; 12(5): 358–371.

6 Persson C, Glimelius B. The relevance of weight loss for survival and quality of life in patients with advanced gastrointestinal cancer treated with palliative chemotherapy. Anticancer Res 2002; 22(6B): 3661–3668.

7 Prado CM, Baracos VE, McCargar LJ, Reiman T, Mourtzakis M, Tonkin K, Mackey JR, Koski S, Pituskin E, Sawyer MB. Sarcopenia as a determinant of chemotherapy toxicity and time to tumor progression in metastatic breast cancer patients receiving capecitabine treatment. Clin Cancer Res. 2009 Apr 15;15(8):2920-6.

8 Sealy MJ, Dechaphunkul T, van der Schans CP, Krijnen WP, Roodenburg JLN, Walker J, Jager-Wittenaar H, Baracos VE. Low muscle mass is associated with early termination of chemotherapy related to toxicity in patients with head and neck cancer. Clin Nutr. 2020 Feb;39(2):501-509.

9 Van Cutsem E, Arends J. The causes and consequences of cancer associated malnutrition. Eur J Oncol Nurs 2005; 9: S51–S63.

10 Vidra N, Kontogianni MD, Schina E, Gioulbasanis I. Detailed Dietary Assessment in Patients with Inoperable Tumors: Potential Deficits for Nutrition Care Plans. Nutr Cancer. 2016 Oct;68(7):1131-9.

11 Arends J, Strasser F, Gonella S, Solheim TS, Madeddu C, Ravasco P, Buonaccorso L, de van der Schueren MAE, Baldwin C, Chasen M, Ripamonti CI; ESMO Guidelines Committee. Electronic address: clinicalguidelines@esmo.org. Cancer cachexia in adult patients: ESMO Clinical Practice Guidelines★. ESMO Open. 2021 Jun;6(3):100092.

**abstract citation id: bjaf055.101**

### The TASTY project: taste steering and taste and smell training in oncology patients

*Madieke Douma*[1]

[1]*University Medical Center Groningen (UMCG). The Netherlands*

**Background:** Anticancer treatments including chemotherapy and targeted therapy often cause taste and smell alterations, which significantly affect the quality of life for patients and their caregivers. These changes can lead to appetite loss, reduced food enjoyment, and poor dietary intake, resulting in nutritional deficiencies. For years, such side effects were seen as unavoidable and received little attention.

**Interventions:** Intervention 1 is taste steering, a coping strategy for patients and caregivers to adapt meals to changes in taste and smell for 6 weeks. An online tool provides personalized advice on food products and becomes more tailored over time through a self-learning algorithm using user feedback. A panel of gastronomic chefs and dietitians offers additional advice. Intervention 2 is taste and smell training for 12 weeks, aiming to restore taste and smell function through daily exposure to standardized stimuli of four tastes (sweet, sour, salty, bitter) and four aromas (rose, cloves, eucalyptol, lime).

**Methods:** Both interventions are studied in multicenter, non-blinded randomized trials with a parallel cluster design in 12 hospitals in the Netherlands. 201 patients with mamma carcinoma, testis carcinoma, or diffuse B-cell lymphoma will participate in the taste steering trial, and 90 patients receiving tyrosine kinase inhibitors will enter the taste and smell training trial. Assessments include smell and taste tests, saliva collection, and questionnaires.

**Conclusion:** The TASTY project investigates the effectiveness of both interventions addressing taste and smell alterations. The project is unique because it is solution-oriented, fosters extensive collaboration across multiple disciplines, and is driven by a strong societal purpose.

**abstract citation id: bjaf055.102**

### Clinical screening with color-associated olfactory testing: a crossmodal tool for early detection of neurodegenerative disorders

*Sally Arnhardt*[1], *Satnam Singh*[2], *Kristin Steinebach*[1], *Juan Manuel Maler*[1], *Timo Oberstein*[1], *Johannes Kornhuber*[1], *Jessica Freiherr*[1,2]

[1]*Department of Psychiatry and Psychotherapy, FAU Erlangen-Nürnberg, Schwabachanlage 6, 91054 Erlangen, Germany*

[2]*Department Sensory Analytics and Technologies, Fraunhofer Institute for Process Engineering and Packaging IVV, Giggenhauser Str. 35, 85354 Freising, Germany*

Olfactory deficits are among the earliest signs of neurodegenerative disorders. As odors are often processed alongside visual cues, within a previous study we developed a color-modulated olfactory test as a potential early screening tool. Two versions of the test were used to assess odor identification under crossmodal conditions: The congruent version used target- associated colors to facilitate odor identification; the incongruent version used distractor colors to increase test difficulty. A clinical sample of 17 patients – 7 diagnosed with Mild Cognitive Impairment (MCI; diagnostic code F06.7) and 10 within the Alzheimer's Disease spectrum (AD; diagnostic codes G30.0, G30.1V, G30.1, G30.9, F00.1, F00.2) – was recruited from the memory clinic at the University Hospital Erlangen. Diagnoses were based on cerebrospinal fluid biomarkers, neuroimaging, and CERAD-Plus. The color-modulated odor test was administered as part of routine follow-up. A two-way ANOVA revealed a significant main effect of diagnosis, indicating that odor identification performance differed significantly between MCI and AD. Post hoc comparisons indicated that MCI participants outperformed AD participants in both congruent and incongruent conditions. A main effect of congruence indicated that odor identification performance varied with odor-color congruency. Post hoc comparisons revealed that MCI participants performed significantly better in the congruent than in the incongruent condition. Similarly, AD participants revealed better performance in the congruent condition. The interaction between diagnosis and congruence was not significant, indicating that the effect of congruence on odor identification performance was similar across diagnostic groups. These findings suggest that the newly developed color-modulated olfactory test may serve as a sensitive screening tool for detection of neurodegenerative disorders, with a potential to differentiate between stages of cognitive decline.

**abstract citation id: bjaf055.103**

### SELECTED SHORT TALKS

### Channel synapse mediates neurotransmission of airway protective chemoreflexes

*Akiyuki Taruno*[1]

[1]*Department of Molecular Cell Physiology, Kyoto Prefectural University of Medicine. Japan*

Neural reflexes to chemicals in the throat protect the airway from aspiration and infection, which can be life-threatening by causing pulmonary disorders. Mechanistic understanding of these reflexes remains premature, exemplified by chronic cough—a sensitized cough reflex—being a prevalent unmet clinical need. Here, in mice, a whole-body search for channel synapses—featuring calcium homeostasis modulator (CALHM) 1/3 channel-mediated neurotransmitter (ATP) release[1–4]—and single-cell transcriptomics uncovered subclasses of the *Pou2f3*$^+$ chemosensory cell family in the throat communicating with vagal neurons via this synapse: type II taste cells in hypopharyngeal taste buds and a subset of tuft cells in the laryngeal epithelium. They express voltage-gated $Na^+$ channels and G protein-coupled receptors (GPCRs) for noxious chemicals, Tas2Rs. Tas2R stimulation exoked action potentials in these cells, as demonstrated by two-photon membrane voltage imaging, and swallow and cough-like expulsive reflexes in the hypopharynx and larynx, respectively. These reflexes were abolished by *Calhm3* or *Pou2f3* knockout and could be triggered by targeted optogenetic stimulation. Furthermore, mold aeroallergen (*Alternaria alternata* extract) exposure augmented CALHM3-dependent expulsive reflex. This study[5] identifies *Pou2f3*$^+$ epithelial cells with channel synapses as chemosensory end organs of airway protective reflexes and sites of their hyperresponsiveness, advancing mechanistic understanding of airway defense programs with distinct therapeutic potential.

**Funding:** This work was supported by JST CREST, JST PRESTO, JSPS KAKENHI, AMED BINDS, Naito Foundation, Takeda Science Foundation, Urakami Foundation, and Salt Science Research Foundation, and MEXT.

### References

1 Nature 495: 223-226, 2013
2 Neuron 98: 547-561, 2018
3 Neuron 106: 815-829, 2020
4 Sci Adv 6: eaba8105, 2020
5 Cell 188: 2687-2704, 2025

**abstract citation id: bjaf055.104**

### Taste regulation of immunity

*Pierre-Yves Musso*[1], *Alix Najera Mazariegos*[2], *Darius Camp*[2], *Martine Berthelot-Grosjean*[3], *Gérard Manière*[3], *Romane Milleville*[4], *George Alves*[3], *Julien Royet*[4], *Yaël Grosjean*[3], *Guy Tanentzapf*[2], *Léo Sillon*[1], *Enisa Aruçi*[1]

[1]*CSGA, TPI team. France*
[2]*UBC. Canada*
[3]*CSGA, PERSING team. France*
[4]*IBDM. France*

Animals use their sensory system to detect cues in their external environment and then communicate, process, and integrate, via the nervous system, these cues in order to induce a specific response. Taste is an important cue used by animals to explore their external environment and can modulate various aspects of behaviour and physiology in animals. A key ongoing challenge for animals is detecting and responding to the presence of a multitude of pathogens in their environment. However, to date, the links between the sensory system and the response to pathogenic threats remain poorly understood. Here we show that Drosophila larvae use their taste system to detect bacterial wall components in their environment and respond by modulating the activity of their cellular immune system. These results show that sensory inputs such as taste have an important role in protecting animals from bacterial infection. Overall, our findings add to the growing list of examples of crosstalk between the nervous system and the immune system and provide novel and important mechanisms for linking them.

### References

1 Detection of bacteria through taste receptors primes the cellular immune response doi: https://doi.org/10.1101/2024.09.26.615243

**abstract citation id: bjaf055.105**

### Role of the lingual GLP-1 system in sweet taste detection

*Savanah Buiret*[1], *Corinne Leloup*[1], *Sandrine Chometton*[1]

[1]CSGA - Centre des Sciences du Goût et de l'Alimentation, CNRS, INRAE, Institut Agro Dijon, Université Bourgogne Europe. France

Sweet taste perception is mediated in the gustatory system by the TAS1R2/TAS1R3 receptor, that responds to a wide range of sweet compounds. Recently, glucose sensors known in other parts of the body to be involved in glucose detection and glucose metabolism have been located in the taste system, but their role is still not clear. The aim of this study was thus to investigate the role of one of them, glucagon-like peptide 1 (GLP-1), in modulating sweet taste sensitivity in mice.

Virogenetic silencing approaches were employed to specifically reduce the expression of the GLP-1 receptor (GLP-1R) in the tongue of adult C57BL/6J mice. Behavioral responses were then assessed using brief-access taste tests in a gustometer. A series of different sweet solutions was presented to the mouse in 10-second trials in a randomized order within a block across a 20-minute session. Three tests were performed: glucose vs fructose (GvF) solutions at three concentrations (0.316 M, 0.56 M, 1.1 M); six concentrations of glucose alone; six concentrations of fructose alone. In the GvF test, a significant difference in licking responses between glucose and fructose was observed at the intermediate 0.56M concentration in GLP-1R knockdown (KD) mice. In the fructose-only test, GLP-1R KD mice exhibited increased licking responses to 0.56 M fructose compared to control mice, while a tendency to a decrease in licking behavior was observed in the glucose-only test at 0.56 M. Virogenetic silencing of GLP-1R in the taste system appears to modulate the ability of mice to perceive sugars at an intermediate concentration, suggesting a role for the GLP-1/GLP-1R system in modulating sweet taste sensitivity. Further studies will assess the impact of GLP-1/GLP-1R signaling in TAS1R2/TAS1R3 knockout models to better understand its role in taste detection without the canonical sweet information. In addition, the potential involvement of lingual GLP-1 in systemic glucose metabolism will also be investigated.

**abstract citation id: bjaf055.106**

### Biomolecular and biophysical AFM probing reveals distinct binding of bitter peptide VAPFPEVF to TAS2R16 without inducing an intracellular calcium response

*Phil Richter[1], Sanjai Karanth[1], Rita Dos Santos Natividade[1], Alessandro Nicoli[1], Małgorzata M. Kogut-Guenthel[1], Julia Benthin[1], Antonella Di Pizio[1], Melanie Koehler[1], Veronika Somoza[1]*

*[1]Leibniz Institute for Food Systems Biology at the Technical University of Munich. Germany*

The conventional method to investigate the activation of G-protein coupled receptors by potential agonists are heterologous cellular expression systems, such as HEK-293T[1], which respond with changes in intracellular calcium levels. Since no specific sequences of bitter-tasting peptides have been identified as bitter taste receptor TAS2R16 agonists so far, we combined molecular biological and biophysical approaches in cellular and cell-free systems as well as molecular dynamics simulations to comprehensively investigate the interactions between the bitter-tasting food-derived peptide VAPFPEVF and TAS2R16. Immortalized parietal cells (HGT-1) were used as cell system as VAPFPEVF was shown to induce proton secretion via functional involvement of TAS2R16.[2,3]

The presented approach revealed that neither VAPFPEVF nor the known TAS2R16 agonist salicin induced a calcium mobilization in TAS2R16-overexpressing HGT-1 cells. However, both compounds stimulated proton secretion, which was amplified by TAS2R16-overexpression by up to 56.5 ± 17.9% ($p \leq 0.05$) and decreased by co-application of the cAMP modulator NKY80 ($p \leq 0.05$). Binding between TAS2R16 and the peptide was demonstrated in cell-based atomic force microscopy (AFM) assays, in which TAS2R16-GFP-overexpressing HGT-1 cells showed higher binding frequencies (13.4 ± 1.9%) than untransfected cells (3.5 ± 0.6%, $p \leq 0.0001$). In a cell-free AFM approach, the binding of the peptide to TAS2R16 embedded in proteoliposomes was validated. Here, the number of binding events was reduced by salicin and TAS2R16 antagonist probenecid ($p \leq 0.05$). Ultimately, AlphaFold multimers and molecular dynamics simulations demonstrated binding of VAPFPEVF to the orthosteric site of TAS2R16.

In summary, we showed that the bitter-tasting peptide VAPFPEVF binds to TAS2R16 without inducing an intracellular calcium mobilization, thereby rendering TAS2R-activation patterns revealed from conventional heterologous expression systems as potentially incomplete.

### References

1 Meyerhof, W., Batram, C., Kuhn, C., Brockhoff, A., Chudoba, E., Bufe, B., Appendino, G., & Behrens, M. (2009). The Molecular Receptive Ranges of Human TAS2R Bitter Taste Receptors. In Chemical Senses (Vol. 35, Issue 2, pp. 157–170). Oxford University Press (OUP). https://doi.org/10.1093/chemse/bjp092

2 Richter, P., Sebald, K., Fischer, K., Behrens, M., Schnieke, A., & Somoza, V. (2022). Bitter Peptides YFYPEL, VAPFPEVF, and YQEPVLGPVRGPFPIIV, Released during Gastric Digestion of Casein, Stimulate Mechanisms of Gastric Acid Secretion via Bitter Taste Receptors TAS2R16 and TAS2R38. In Journal of Agricultural and Food Chemistry (Vol. 70, Issue 37, pp. 11591–11602). American Chemical Society (ACS). https://doi.org/10.1021/acs.jafc.2c05228

3 Richter, P., Sebald, K., Fischer, K., Schnieke, A., Jlilati, M., Mittermeier-Klessinger, V., & Somoza, V. (2024). Gastric digestion of the sweet-tasting plant protein thaumatin releases bitter peptides that reduce H. pylori induced pro-inflammatory IL-17A release via the TAS2R16 bitter taste receptor. In Food Chemistry (Vol. 448, p. 139157). Elsevier BV. https://doi.org/10.1016/j.foodchem.2024.139157

**abstract citation id: bjaf055.107**

### Predicting appetitive response to food odours from expected gustatory properties and taste-liker status

*Putu Agus Khorisantono*[1], *Apostolia Filippopoliti*[1], *Janina Seubert*[1]

[1]*Department of Clinical Neuroscience, Psychology Division, Karolinska Institutet, Stockholm. Sweden*

During food consumption, odours combine with tastes to create a multisensory flavour experience. This repeated concurrent and contingent stimulation leads to some food odours acquiring a taste property, where the taste sensation of different foods can be predicted from their odour. However, how these predicted consummatory properties, such as taste, modulate appetitive behaviour towards different foods remains largely unknown. In this pre-registered study, we combine taste-liker status with subjective maps of food odours in sweet-savoury space to predict their appetitiveness. Healthy participants attend a session where they rate the pleasantness of both sweet and savoury taste stimuli of varying concentrations. They subsequently rate nine orthonasally delivered food odours based on their perceived sweetness and savouriness, along with their wanting to consume the food associated with the odour. Taste-liker status was derived from the turning points of the pleasantness ratings of the sweet tastes and savoury tastes. Mixed-effect modelling indicates that odour-elicited food wanting can be predicted by an interaction between taste-liker status and expected taste properties of the food odour.

Furthermore, an orthogonal model characterising food odours by a vector of sweetness and savouriness outperforms a spectral model, where food odours are characterised by a scalar value derived from the difference of sweetness and savouriness. A leave-one-subject-out cross-validation strategy showed that odour wanting prediction models were generalisable across subjects. Pattern-based analyses on task fMRI data allows us to localise cortical representations of odour maps. These results highlight strong taste-odour connections formed through lifelong associative learning, with important implications for encouraging healthier and more sustainable eating habits.

**Funding:** This project has received funding from the European Research Council (ERC) under the European Union's Horizon 2020 research and innovation programme (grant agreement n° 947886) to JS and by the Swedish Research Council (VR 2018-0318 and VR 2022-02239) to JS.

**abstract citation id: bjaf055.108**

### GLP-1 receptor agonists significantly impair taste function

*Richard Doty*[1], *Rafa Khan*[1]

[1]*University of Pennsylvania. United States*

Over 10% of the US population are prescribed glucagon-like peptide-1 receptor agonists (GLP-1 RAs) to combat obesity. Although they decrease cravings for foods, their influence on chemosensory function is unknown. We employed state-of-the- art quantitative taste and smell tests to address this issue. The 53-item Waterless Empirical Taste Test (WETT®) and the 40- item University of Pennsylvania Smell Identification Test (UPSIT®) were completed by 46 persons taking GLP-1 RAs and 46 controls matched on age, sex, smoking behavior, and COVID-19 infection histories. Data were analysed using analyses of variance. The WETT® scores were significantly diminished in the GLP-1 RA group relative to controls [total means (95% CIs)=28.61 (25.66,31.56) and 40.63 (38.35,42.91), $p<0.001$, $\eta2=0.37$]. Eighty five percent of the GLP-1 subjects scored worse than their individually matched controls. All 5 WETT® subtest scores were similarly affected (ps<0.001). Smell function, although slightly decreased on average, was not significantly impacted ($p=0.076$). Women outperformed men on all tests. Remarkably, UPSIT® and WETT® scores were higher, i.e., better, in those reporting nausea, diarrhoea, and other GLP-1-related side effects. This study demonstrates, for the first time, that GLP-1 RAs alter the function of a major sensory system, significantly depressing the perception of all five basic taste qualities. The physiologic basis of this effect is unknown but may involve GLP-1 receptors in the brainstem and afferent taste pathways, as well as vagus nerve-related processes. Subject payments were made from Sensonics International's Research Fund. No other outside funding was used for this study.

**abstract citation id: bjaf055.109**

### Chemosensory inputs to the drosophila navigation center maps odor to action

*Kavin Nunez Nunez*[1], *Karin Van Hassel*[2], *Yijie Yin*[3], *Jacob Freed*[2], *Mubarak Syed*[4], *Albert Cardona*[3], *Katherine Nagel*[2]

[1]*New York University Langone Health. United States*
[2]*NYU School of Medicine. United States*
[3]*University of Cambridge. United Kingdom*
[4]*University of New Mexico. United States*

Like most animals, Drosophila melanogaster uses chemosensory information to guide navigation and foraging. Fan-shaped body tangential neurons are anatomically poised to provide chemosensory input to the fly navigation center to drive behavior. However, how chemosensory information is encoded across the population of tangential neurons is unknown. Here we characterize chemosensory representations across tangential neurons and link these representations to behavior. First, we surveyed the responses of several developmental classes of tangential neurons to both odorants and tastants using wide-field imaging. We observed chemosensory responses across most classes of tangential neurons. Interestingly, most neurons derived from one developmental lineage exhibited strong inhibition in response to chemosensory input. Next, we validated these measurements using whole-cell electrophysiology, showing that both temporal responses, response polarity, and chemosensory tuning of identified neurons were similar when measured with electrophysiology or imaging.

Electrophysiology from single cells revealed that some tangential neuron types show highly stereotyped tuning across flies while others show variable tuning. Intriguingly, neuron types that show variability across flies also show variable clustering in the two published connectomes, suggesting possible developmental heterogeneity. Finally, based on our imaging data, we either activated or inactivated single tangential neuron types to gauge the effects of their odor responses on behavior. We observed that inactivation of a subset of tangential neurons was able to reliably drive strong upwind navigation. In ongoing work, we are comparing the measured chemosensory tuning of tangential neurons to connectome-derived predictions. The expected output of this work is a first map of how tangential inputs to the fly navigation center transform chemosensory information into behavior.

### References

1 NIH Blueprint(D-SPAN) Award (F99/K00- 5K00NS118741-04)
2 NSF neuronex: Odor2Action

**abstract citation id: bjaf055.110**

### Tuft (taste) cell-like subtypes in human biliary inflammation: chemosensory epithelial diversity revealed by single-cell profiling

*Dmytro Vlasenko[1], Tamara Papadakis[2], Andrea Maccagno[3], Sanna Adriano[4], Shanjid Shiplu[5], Ulrich Gaertner[4], Bruno Maerkl[3], Marco Koch[5], Maryam Keshavarz[5]*

[1]*Department of General, Visceral and Transplantation Surgery, University Hospital Augsburg, Augsburg, Germany*
[2]*Institute for Anatomy and Cell Biology, German Center for Lung Research, Justus Liebig University, Giessen, Germany*
[3]*Department of Neuropathology, Pathology, Medical Faculty, University of Augsburg, Augsburg, Germany*
[4]*Institute for Anatomy and Cell Biology, German Center for Lung Research, Justus Liebig University, Giessen, Germany*
[5]*Anatomy and Cell Biology, Institute of Theoretical Medicine, Faculty of Medicine, University of Augsburg; Augsburg, Germany*

**Objectives of the Study:** This study aimed to investigate the presence and potential immunoregulatory roles of tuft (chemosensory) cells in the human biliary tract. While such cells have been identified in mice, where they sense microbial signals and influence immune responses, their presence and function in human biliary tissues, particularly during inflammation, were previously unknown.

**Experimental Methods Used:** Gallbladder tissues from patients with acute cholecystitis and chronic cholelithiasis were analyzed using immunohistochemistry, transmission electron microscopy, immuno-electron microscopy, and single-cell RNA sequencing. These methods allowed for both structural and molecular characterization of epithelial cell populations.

**Essential Results:** Rare epithelial cells expressing lymphoid-restricted membrane protein (LRMP) were identified, displaying features typical of tuft cells, including apical microvilli and vesicle-rich cytoplasm. Single-cell transcriptomic profiling revealed distinct epithelial subclusters, including rare chemosensory-like populations with gene signatures suggesting sensory and immunoregulatory roles. Acute inflammation was associated with epithelial disruption and immune infiltration, while chronic inflammation showed preserved structure and vesicular remodeling.

**Conclusions:** This study provides the first evidence for tuft cell-like subtypes in the human biliary epithelium. These cells may play important roles in epithelial sensing and modulation of inflammation, offering new insights into the sensory-immune interface in human biliary disease.

**Funding:** This work was supported by the Deutsche Forschungsgemeinschaft (DFG, German Research Foundation), grant to Dr. Keshavarz, project number 561973651.

**abstract citation id: bjaf055.111**

### 3. POSTERS

### Neural oscillation signatures of the human olfactory bulb: patterns in active and passive odor sampling

*Shubin Li[1], Coralie Mignot[2], Susanne Weise[2], Thomas Hummel[2]*

[1]*Universitätsklinikum Carl Gustav Carus Dresden. Germany*
[2]*Smell & Taste, Dept. of ORL, TU Dresden. Germany*

As the first and only relay station between the peripheral and central olfactory systems, the olfactory bulb (OB) plays a crucial role in human olfactory processing. This study investigated OB activity during active and passive sniffing of odors with different valences. We recruited 32 participants, dividing them into two groups: an active sampling group and a passive sampling group. Sixteen participants experienced odor delivery synchronized with their breathing (synchronous design), while the other 16 followed an asynchronous design. Electrobulbogram (EBG), a non-invasive scalp-based method, was used to record OB activity, with 64 channels on scalp and 4 external electrodes on forehead. Our results indicated that early gamma and beta oscillations may reflect the OB's role in initial odor encoding. Both active and passive sniffing exhibited similar patterns of repetitive high-gamma oscillations, potentially signifying rhythmic information refreshing. Additionally, mid-phase beta activation was observed during passive odor sampling, suggesting a greater reliance on intrinsic brain regulation. Furthermore, individuals highly sensitive to olfactory ERP tasks exhibited delayed EBG components, which may reflect individual differences in olfactory processing strategies influenced by higher cortical regions. In conclusion, gamma and beta oscillations are critical for OB function and may underlie different olfactory processing mechanisms in active versus passive sniffing. The results emphasize the role of OB dynamics in olfactory perception and suggest that passive and active odor sampling engage distinct but overlapping neural mechanisms.

**Funding:** From University Clinic of the TU Dresden (BO-EK-78022020)

**abstract citation id: bjaf055.112**

### The attentional blink is not, is not, is not modulated by lavender vs. peppermint smell

*Christina Bermeitinger*[1],
*Ryan P. M. Hackländer*[1], *Pamela Baess*[1]

[1]*University of Hildesheim. Germany*

Lavender and peppermint are commonly utilized odors, believed to induce specific states in individuals—relaxation in the case of lavender, and alertness with peppermint. The possible (physiological or psychological) mechanisms through which these effects occur remain under discussion. Another area of debate is the cognitive processes that are influenced by odors. Our current research focuses on the topic of temporal attention, best examined through the attentional blink paradigm. In this paradigm, a rapid sequence of visual stimuli is displayed on the screen. The task was to attend to two target stimuli.

Typically, the ability to accurately detect the second target stimulus (T2) decreases within 200 to 600 ms after the first target stimulus (T1); this is called the attentional blink (AB) effect. Colzato et al. (2014) reported some evidence suggesting that the AB can be modulated by different ambient odors: with peppermint increasing the AB compared to lavender. They proposed that lavender has a calming effect that might lead to reduced attention allocation to the task, attenuating the AB effect. In contrast, peppermint may heighten arousal, thus accentuating the over-investment in the task and enhancing the AB. Through a series of 3 experiments, we sought to replicate and expand upon this study by employing both trial-by-trial and block-wise odor presentations using an olfactometer. Across all our experiments (no external funding; with 112 human subjects who provided written informed consent and were treated according to ethical guidelines), we did not observe any evidence supporting modulation of the AB by peppermint ("Pfefferminze" from Primavera Life, Germany) compared to lavender ("Lavendel fein" from Primavera Life, Germany) odors. We compare our findings with those of Colzato et al., consider them alongside other empirical research in this area, and discuss them in relation to broader principles of how odors might affect cognition.

### Reference

1 Colzato, L. S., Sellaro, R., Rossi Paccani, C., & Hommel, B. (2014). Attentional control in the attentional blink is modulated by odor. Attention, Perception, & Psychophysics, 76(6), 1510–1515.

**abstract citation id: bjaf055.113**

### The ablation of Tas2r108 induced alterations of leptin and metabolism

*Kyung-Nyun Kim*[1], *Ki-Myung Chung*[1], *Young-Kyung Cho*[1]

[1]*Department of Physiology and Neuroscience, and Research Institute of Oral Sciences, Gangneung-Wonju National University. Republic of Korea*

The sense of taste is crucial for survival and maintaining quality of life. The taste receptors are mainly divided into the T1R family, which comprises receptors for sweet and umami tastes, and the T2R family, which includes bitter taste receptors. Specifically, the genes for the bitter taste receptors in humans (*TAS2R*) and in mice (*Tas2r*) have been identified, with 25 known genes in humans and 35 in mice. Our previous studies have shown that bitter taste receptors are expressed in the tongue with variations in expression levels depending on the gene. Notably, *Tas2r108* was the most highly expressed gene in taste papillae. This research aims to explore the probable physiological functions of the *Tas2r108* bitter taste receptor.

*Tas2r108* knockout mice were created using *CRISPR/Cas*9 technology, and male *Tas2r108*$^{-/-}$ mice were selected for studies. To confirm the long-term effects of *Tas2r108* knockout(KO), we monitored the normal processes of fertilization and pregnancy, as well as the lifespan and physiological indicators including body weight, blood pressure, blood glucose, and plasma leptin levels (approved in GWNU-2020-24-1). The ligand for t2r108 was identified through $Ca^{2+}$ activity assays in CHO cells transfected with *Tas2r108*. *Tas2r108* responded various bitter substances including cycloheximide and denatonium. In *Tas2r108* knockout mice, the bitter taste sensitivity was changed. No significant changes in growth were observed during pregnancy or lactation periods. The lifespan of *Tas2r108*$^{-/-}$ mice was extended without the chronic metabolic diseases except for an increase in plasma leptin and mild overweight. The results suggest that KO of *Tas2r108* would elicit change in metabolism or feeding behavior and/or would induce life span extension.

**Funding:** The work is supported by Basic Science Research Program through the National Research Foundation in Korea funded by the Ministry of Science, ICT and Future Planning (2020R1F1A1049633)

**abstract citation id: bjaf055.114**

### Impact of smelling food odorants on salivary and emotional responses

*Carla Simões*[1], *María Botia Gonzalez*[2], *Damian Escribano*[2], *José Parraça*[3], *Ana Isabel Costa*[4], *José Cerón*[2], *Asta Tvarijonaviciute*[2], *Elsa Lamy*[1]

[1]*MED&CHANGE - University of Évora. Portugal*
[2]*Interdisciplinary Laboratory of Clinical Analysis - University of Murcia (Interlab-UMU). Spain*
[3]*Department of Sport and Health, School of Health and Human Development, University of Évora. Portugal*
[4]*Food Behaviour Lab, CATÓLICA LISBON School of Business & Economics, Universidade Católica Portuguesa. Portugal*

Food smell plays an essential role in ingestive behaviour, triggering psychophysiological responses, including increase salivation and changes in autonomic nervous system activity. The emotional states that food stimuli evoke may vary among individuals. Implicit methods, such as galvanic skin response or heart rate variability, have been proposed to infer arousal, whereas the analysis of facial expressions can detect valence. Saliva has also been used to access stress, however studies linking different measurements of emotional states evoked by food smells with saliva are missing. The present work aims to investigate how measures of emotional states are correlated with salivary levels of cortisol, amylase and oxytocin. We conducted a within-subject design study involving 41 healthy adults, from both sexes. Participants were exposed to 2 different food (vanilla and orange) and a non-food (pine) odorants randomly presented, and saliva samples were collected before and during 3-minute exposure to each odorant. Changes in the activity of the autonomic nervous system were measured through galvanic skin response and heart rate variability, and facial expressions were analyzed through appropriate software. Participants also rated the odors tested in study regarding liking and desire to consume foods containing those odorants. Preliminary results show that smelling food odorants seems to trigger a higher increase in saliva secretion in comparation to non-food. Despite the large interindividual variability in terms of emotional arousal, a clear difference from food to non-food odorants was observed. The association between arousal and valence measurements and changes in salivary cortisol, amylase and oxytocin will be presented. Studying physiological and emotional responses in anticipation to smell of food may offer insight into mechanisms underlying the regulation of ingestive behavior, highlighting the potential for multisensorial interventions targeting eating-disorders.

**Funding:** This work was funded by the Foundation for Science and Technology (FCT) under the PhD studentship 2021.06485.BD awarded to Carla Simões.

**abstract citation id: bjaf055.115**

### Predicting human olfactory perception by odorant structure and receptor activity profile

*Chiori Ijichi[1], Yusuke Ihara[1], Yasuko Nogi[1], Masayuki Sugiki[2], Yuko Kodama[1], Sayoko Ihara[3], Mika Shirasu[3], Takatsugu Hirokawa[4], Kazushige Touhara[3]*

[1]*Institute of Food Sciences and Technologies, Food Products Division, AJINOMOTO CO., INC. Japan*
[2]*Research Institute for Bioscience Products and Fine Chemicals, AJINOMOTO CO., INC. Japan*
[3]*Department of Applied Biological Chemistry, Graduate School of Agricultural and Life Sciences, The University of Tokyo. Japan*
[4]*Division of Biomedical Science, Faculty of Medicine, University of Tsukuba. Japan*

**Objectives:** The objective of this study was to clarify the relationship between molecular structure, OR activity profiles, and perceptual odor similarity and to build a calculation model to predict odors.
**Methods:** The degree of perceptual odor similarity to eugenol among eugenol, vanillin, and structurally similar compounds was scored, and OR activity profile of these compounds was evaluated. We also developed a new method to represent the molecular structure of odorants as 3D shapes and pharmacophore similarity fingerprints, considering the 3D structural similarities between various odorants with multiple conformations. Finally, we conducted structure-OR activity relationship analyses for predicting odor similarity.
**Results:** Our results indicated that eugenol - vanillin structurally similar compounds primarily activated six ORs, and the activity profiles of these ORs correlated with their perception. This enabled the development of a prediction model for the perceptual similarity score from OR activity profiles (coefficient of determination, R2 = 0.687). These 3D shape and pharmacophore fingerprints could also predict the perceptual odor similarity score (R2 = 0.514). Finally, we identified key molecular structural features that contributed to predicting sensory similarities in the structurally related compounds evaluated in this study.
**Conclusions:** Our models can predict odor similarity from OR activity profiles and the 3D structural similarities among odorants in narrow odor space. The odor prediction model using ORs activity data was more accurate than the model using the structure data. This suggests the usefulness of obtaining and using ORs data.

Funding: This work was supported by Ajinomoto Co., Inc., and the ERATO Touhara Chemosensory Signal Project from JST, Japan.
**Funding:** JST ERATO Touhara Chemosensory Signal Project JPMJER1202

**abstract citation id: bjaf055.116**

### Sequence analysis and structural annotation of odorant-binding proteins (OBPs) and Niemann Pick C2 proteins (NPC2) from Dermanyssus gallinae

*Emma Mathieu[1], Rajesh Durairaj[1], Samia Laib[1], Aurore Lamy[1], Patrick Pageat[2]*

[1]*Department of Bioinformatics and Chemical Communication (D-BICC), Research Institute in Semiochemistry and Applied Ethology (IRSEA), Quartier Salignan, 84400 Apt. France*

[2]*Department of Chemical Ecology, Research Institute in Semiochemistry and Applied Ethology (IRSEA), Quartier Salignan, 84400 Apt. France*

Dermanyssus gallinae (Dgal), commonly known as the poultry red mite, is an ectoparasite that infests domestic birds, leading to significant losses in the poultry industry. Mites, a highly diverse group of arthropods, rely on chemical cues to detect their food, hosts, or prey in their environment. Odorant-binding proteins (OBPs) and chemosensory receptors play a crucial role in processing these signals. Sequence and structure annotation studies have not been performed for this species. This research employs bioinformatics approaches to investigate the members of six OBPs and chemosensory protein family of six NPC2s (Niemann-Pick type C2s) in Dgal. The results revealed significant sequence conservation, along with six pairs of cysteines forming disulfide bridges that ensure structural integrity, which are present in all protein sequences except OBP5. Through evolutionary tree analysis, the results indicate that DgalOBP1-5 and OBP6 have diverged into two distinct clades, while NPC2-4 and 6 also grouped into separate clades, associating with different mite members rather than the tics lineage. Furthermore, the 6 OBP and 6 NPC2 structures were predicted by TrRosetta tool, which is web- based platform for fast and accurate protein structure prediction and validated by molecular dynamic simulation (MDS) analysis. DgalOBPs are alpha helices, like insect OBPs, while DgalNPC2s are mainly beta sheets. The DgalOBP3/4 and NPC2-5/6 proved to be more stable in the value of root mean square deviation (RMSD). Therefore, the study will enable us to conduct receptor-ligand interactions with selective disturbing compounds in the future.

### References

1 Bhowmick, B., Tang, Y., Lin, F., Øines, Ø., Zhao, J., Liao, C., … & Han, Q. (2020). Comparative morphological and transcriptomic analyses reveal chemosensory genes in the poultry red mite, Dermanyssus gallinae. Scientific Reports, 10(1), 17923.

2 Mills, R. I. L. (2023). Investigations into the molecular basis of chemically mediated interactions between the poultry red mite, Dermanyssus gallinae and the domesticated chicken, Gallus gallus domesticus (Doctoral dissertation, University of Greenwich).

**abstract citation id: bjaf055.117**

### Vomeronasal organ condition in 5XFAD Alzheimer mouse model: preliminary results of a histological study

*Valentina Gazzano[1], Violaine Mechin[2], Marine Verbaere[2], Alessandro Cozzi[2], Estelle Descout[2], Angelo Gazzano[1], Simona Capsoni[3], Carlo Cantile[1], Patrick Pageat[2], Pietro Asproni[2]*

[1]*Department of Veterinary Sciences, University of Pisa. Italy*
[2]*IRSEA – Research Institute in Semiochemistry and Applied Ethology. France*
[3]*Section of Human Physiology, Department of Neuroscience and Rehabilitation, University of Ferrara. Italy*

The vomeronasal organ (VNO) plays a crucial role in chemical cues detection and animal behaviour. Recent studies revealed that this organ can be affected by degenerative changes induced by natural and pathological aging in mice, strongly affecting the vomeronasal sensory epithelium (VNSE) and reducing semiochemicals detection. Since the Alzheimer's disease (AD) induces olfaction deficits, the aim of this study was to evaluate if also the VNO can be affected by cellular changes in a commonly used AD mouse model, the 5XFAD. The protocol was approved by the Italian Ministry of Health (authorization n. 406/2021-PR of the 04/06/2021) and involved 48 VNOs from 24 5XFAD mice and 36 VNOs from 18 wild type mice, which were submitted to histological analysis (hematoxylin-eosin and PAS stain) to assess the presence of VNSE vacuolization and degeneration, VNSE thickness and glycogen accumulation. The statistical analysis revealed that no differences were observed between the two groups concerning the presence of vacuolization (MOLR, $p=0.79$) and degenerative changes (MOLR, $p=0.22$) of the VNSE. In WT and in 5XFAD mice, VNSE mean thickness was 103,18 µm (±17,61) and 103,69 µm (±10,19), respectively, with no statistical difference among the two genotypes (GLMM, $p=0.9$). Similarly, glycogen accumulation, assessed on PAS-stained sections, did not differ among groups (GLMM, $p=0,12$). Interestingly, also WT mice presented higher VNSE degeneration levels than our previous studies in mice of different strains of similar age. These preliminary results seem to suggest that the pathological aging induced in the 5XFAD AD mouse model does not affect the condition of the VNSE compared to WT, as we previously observed in natural aging. Finally, VNSE conditions of the WT mice suggest that the choice of the model is a crucial stage in semiochemical research, since it is possible that the strain can influence the condition, and possibly the functionality, of the VNO.

### References

1 - Portalés A, Chamero P, Jurado S. Natural and Pathological Aging Distinctively Impacts the Pheromone Detection System and Social Behavior. Mol Neurobiol. 2023, 60:4641-4658. doi: 10.1007/s12035-023-03362-3.

2 - Mechin V, Pageat P, Teruel E, Asproni P. Histological and Immunohistochemical Characterization of Vomeronasal Organ Aging in Mice. Animals. 2021, 11:1211. doi: 10.3390/ani11051211.

3 - Son G, Yoo SJ, Kang S, Rasheed A, Jung DH, Park H, Cho B, Steinbusch HWM, Chang KA, Suh YH, Moon C. Region-specific amyloid-β accumulation in the olfactory system influences olfactory sensory neuronal dysfunction in 5xFAD mice. Alzheimers Res Ther. 2021, 13:4. doi: 10.1186/s13195-020-00730-2.

abstract citation id: bjaf055.118

### Towards a chemosensory understanding of modified peptides: a biln-based graph neural network for bitterness prediction

*Francesco Ferri*[1], *Alexandra Steuer*[1], *Alexander Otterbach*[1], *Antonella Di Pizio*[1]

[1]*Leibniz Institute for Food Systems Biology at the Technical University of Munich. Germany*

Taste is a key factor in food choices and significantly influences overall health. Food processing can generate peptides, including modified peptides, through enzymatic hydrolysis, fermentation, or thermal treatment. These peptides may develop an unpleasant, bitter taste. Predicting the bitter taste of peptides early on will enable the optimization of processing methods.

However, incorporating chemical modifications poses unique challenges in representing peptides for machine learning applications. Traditional line-notation formats, such as FASTA, offer a simplistic view of sequence identity but fail to capture structural and functional complexities, such as cyclization and peptide modification. Conversely, Boehringer Ingelheim Line Notation (BILN) offers a robust framework for encoding these complex peptides.

We propose a bitter peptide predictor that uses BILN notation. Translating BILN notation into structured graph representations has allowed us to bridge the gap between computational precision and biological relevance. The model enables accurate assessments of peptide-induced sensory responses.

Accurately predicting the taste properties of peptides is essential to advancing alternative protein development as the presence of bitterness can negatively impact consumer acceptance.

abstract citation id: bjaf055.119

### Omega-3 fatty acids and their influence on feeding behavior in rainbow trout: unraveling the sensory mechanisms

*Jerome Roy*[1], *Maud Martinat*[1], *Elodie Baranek*[1], *Marie Lasserre*[1], *Claire Vigor*[2], *Guillaume Reservat*[2], *Camille Oger*[2], *Cécile Heraud*[1], *Anne Surget*[1], *Anthony Lanuque*[1], *Frederic Terrier*[1], *Jean-Marie Galano*[2], *Thierry Durand*[2], *Christine Belloir*[3], *Loic Briand*[3]

[1]*INRAE, Université de Pau et des Pays de l'Adour, E2S UPPA, UMR1419 Nutrition Métabolisme, Aquaculture. France*
[2]*Institut des Biomolécules Max Mousseron (IBMM), UMR 5247, Pôle Chimie Balard Recherche, CNRS, Université de Montpellier, ENSCM, Montpellier. France*
[3]*Centre Des Sciences du Goût et de l'Alimentation, CNRS, INRAE, Institut Agro, Université Bourgogne, Dijon. France*

In the context of global aquaculture expansion, reducing reliance on wild fish stocks, still used to produce fishmeal and fish oil, remains a major challenge. Replacing these ingredients with plant-based alternatives is a key priority. However, their full substitution often reduces survival and growth in rainbow trout, from first feeding onward. Increasing evidence suggests that altered feeding behavior may contribute to these impairments, yet few mechanistic studies have addressed this. Marine ingredient exclusion also removes dietary omega-3 fatty acids (DHA and EPA), whose roles in feeding regulation remain unclear. Taste perception and nutrient detection are essential for assessing feed quality and ensuring adequate intake. Our research shows that dietary omega-3 levels significantly influence trout feeding preferences and behavior. Moreover, diet type (marine vs. plant-based) modulates sensory nutrient detection, particularly via omega-3-sensitive receptors in gustatory and olfactory tissues. Notably, even short-term plant-based feeding (one meal or 30 days) alters serotonergic signaling—key for taste transmission and behavioral regulation. Our results also reveal the involvement of free fatty acid receptors (FFARs) in feeding behavior regulation, even from early life stages. These findings highlight how plant-based diets can disrupt feeding responses and reveal trout's high sensitivity to dietary signals. They emphasize the importance of early nutritional programming on sensory function and central control of feeding. This work addresses critical challenges in sustainable aquaculture by supporting the development of improved feeds and identifying biomarkers of dietary resilience under environmental change.

**Funding:** This research was funded by the French National Research Agency (grant no. ANR-21-CE20-0009-01 "FEEDOMEGA").

abstract citation id: bjaf055.120

### Creating an atmosphere for giving: How pleasant scents but not pronouns foster charitable intentions

*Ilja Croijmans*[1], *Jasper De Groot*[2], *Sebastian Sadowski*[1]

[1]*Centre for Language Studies, Radboud University. The Netherlands*
[2]*Behavioral Science Institute, Radboud University. The Netherlands*

Charitable behavior, for example donating money to charitable organizations, is important for social cohesion and fostering equality. The psychological mechanisms underlying charitable behavior, the influence of specific linguistic constructions on charitable behavior, or the influence of smells on it, are not well understood. By examining the impact of linguistic cues (inclusive pronouns) and sensory cues (social presence-signaling odors that are pleasant but unfamiliar, in combination with familiar scents), this study tests whether the congruence of these cues enhances prosocial behavior more effectively than their individual effects. Participants ($N = 161$) enrolled in a mixed-design study with two factors: pronoun condition (between-subjects: 'you' vs. 'we') and odor condition (counterbalanced within-subjects: own smell, other smell, 'we' smell, odorless control). Specifically, participants were exposed to 4 odors, while reading matched texts about 4 charitable organizations, manipulated for pronoun usage. After reading and smelling, they responded to measures of donation intention and attitudes toward each charity. Although the pre-registered interaction between pronoun and odor was not observed, the findings showed pleasant and familiar smells significantly enhanced immediate and future donation behavior and attitudes toward the charity, consistent with theories emphasizing the importance of positive affect and trust in promoting social behavior. The follow-up correlation analyses for smell perception suggest that these effects were in particular driven by the pleasantness of the smells, and were not due to the familiarity per se. There were no main effects for pronoun condition, suggesting comparable charitable intentions following reading advertisements that use 'you' versus 'we'. These results offer actionable insights for charities, suggesting that creating a pleasant olfactory environment has a greater impact on donations than specific language choices.

**Funding:** This study was supported by Radboud University Centre for Language Studies Small Research Grants, grant numbers RG2023-18 and RG2024-34

**abstract citation id: bjaf055.121**

### The benefits of an early olfactory awakening for the development of conscious olfactory skills

*Maryse Delaunay[1], Ana Reutimann[2], Marie Anquetil[1]*

*[1]Laboratoire de Psychologie de Caen Normandie (UR 7452), Université de Caen Normandie. France*
*[2]Centre Hospitalier Robert Bisson. France*

In western societies, sensory awakening neglects olfaction, despite its essential role in feeding, danger avoidance and overall development. Some studies report olfactory performances in young children from birth. Scarce studies further show that young children can pay attention to olfactory stimuli when invited to do so. However, how an awakening olfaction intervention promotes the early development of early olfactory skills has not been described yet. This is the aim of the present pilot study of the EVOLF project, carried out in a nursery from Normandy (France).

A follow-up of 13 children (7 males) from 16 to 42 months (M= 31 ± 10 months) began after obtaining written ethical agreements, as well as parental permission. The children were videotaped facing natural scented objects presented successively, during 3 phases: before, during and after an olfactory awakening intervention. Using The Observer® XT17 (Noldus), two independent observers annotated, in number and duration, the actions of smelling and looking at the object immediately after odorant detection, that reveals a conscious olfactory detection. They also recorded the duration of behavioural inhibition. The observations were reliable ($k > 0.8$).

Wilcoxon tests, comparing the target behaviours displayed during various phases, were first calculated to estimate the impact of the intervention. But, since we were unable to recruit a control group with similar characteristics than those of the group awaken, and because only 9 children were followed-up until the end, individual developmental trajectories were also compared.

The results revealed a significant change in olfactory exploration during and after olfactory awakening ($p<0.05$). However, individual developmental trajectories varied. Inhibited children did not explore odorants. This pilot study proves that early olfactory awakening allows the emergence of conscious olfactory skills, but also that behavioural inhibition blocks children's cognitive engagement.

**Funding:** Financial support from CapEnfants®

### References

1 Delaunay-El Allam M, Marlier L, Schaal B. Learning at the breast: preference formation for an artificial scent and its attraction against the odor of maternal milk. Infant Behav Dev. 2006 Jul;29(3):308-21. doi: 10.1016/j.infbeh.2005.12.008. Epub 2006 Jan 23. PMID: 17138287.
2 Delaunay-El Allam M, Soussignan R, Patris B, Marlier L, Schaal B. Long-lasting memory for an odor acquired at the mother's breast. Dev Sci. 2010 Nov;13(6):849-63. doi: 10.1111/j.1467-7687.2009.00941.x. PMID: 20977556.
3 Kucirkova NI, Tosun S. Children's Olfactory Picturebooks: Charting New Trends in Early Childhood Education. Early Child Educ J. 2023 Mar 18:1-10. doi: 10.1007/s10643-023-01457-z. Epub ahead of print. PMID: 37360605; PMCID: PMC10024514.
4 Marlier L, Schaal B, Soussignan R. Orientation responses to biological odours in the human newborn. Initial pattern and postnatal plasticity. C R Acad Sci III. 1997 Dec;320(12):999-1005. doi: 10.1016/s0764-4469(97)82473-0. PMID: 9587477.
5 Soussignan R, Schaal B, Marlier L, Jiang T. Facial and autonomic responses to biological and artificial olfactory stimuli in human neonates: re-examining early hedonic discrimination of odors. Physiol Behav. 1997 Oct;62(4):745-58. doi: 10.1016/s0031-9384(97)00187-x. PMID: 9284493.

**abstract citation id: bjaf055.122**

### Episodic olfactory autobiographical memory and the persistence of self

*Benjamin D. Young[1]*

*[1]Philosophy and Neuroscience, University of Nevada Reno. United States*

Remembering smells is often claimed to be special. Odors allegedly mentally transport us back to a time and place with a strong sense of presence and vivid emotional engagement. Additionally, both psychologists and philosophers have converged on the idea that autobiographical memories (AM) play an essential role in generating our persisting sense of self. However, it is still an open question "What, if anything, olfactory AM contribute to our persisting sense of self?" Building upon a survey of the experimental and theoretical literature it is argued that olfactory AM are not special in generating a phenomenologically more emotionally vivid connection to the past. Rather, they play an important role in grounding our persisting sense of self. The smell component of AM provides purchase in situating ourselves within an autobiographical episode by providing a sense of embodiment and embeddedness within these historical contexts beyond that provided from other perceptual modalities. Moreover, a further speculative conclusion is offered that the occurrence of olfactory aspects within multi-modal AM can be employed as a means of validating the corresponding accuracy of the episodes depicted by long-term AM memories.

**abstract citation id: bjaf055.123**

### The hidden burden: the overlooked psychological toll of body odour in everyday life

*Monique A.M. Smeets[1], Francesca di Cicco[1], Mark Hirst[2], Ilja Croijmans[3], Tim Madden[2], Lynda Grainger[2], Zoe Jackson[4], Timo Giesbrecht[2], Jeremy Shen[5], Anna Thomas[2]*

*[1]Utrecht University. The Netherlands*
*[2]Unilever R&D Port Sunlight. United Kingdom*
*[3]Radboud University. The Netherlands*
*[4]Unilever R&D Leeds. United Kingdom*
*[5]Unilever R&D Trumbull. United States*

These days there is a lot of interest in body odour as a carrier of chemical signals enabling communication from a sender to a receiver. What tends to be overlooked is the explicit quality of body odour as mostly unpleasant which can impact the sender's psychological wellbeing. We developed the UnderarmCARE (Condition and Affective Response Evaluations) Questionnaire to quantify the impact of (non-clinical) underarm issues on the quality of life of healthy individuals.

**Methods:** The questionnaire has 3 subscales (Sweating, Odour, and Skin issues) that were developed through a multistep iterative process followed up by exploratory and confirmatory factor analysis. It was administered online along with the Perceived Stress Scale (PSS), the Sleep Health Index (SHI), and self-reported assessments of irritation, sweat, and malodour rate, frequency and concern to 1184 participants in the USA.

**Results:** The data was randomly split into Sample 1 and Sample 2 for initial exploratory self-perceive and confirmatory factor analysis. Scores from all three subscales were negatively correlated with the PSS and positively correlated with the SHI, suggesting that lower underarm wellbeing is associated with higher stress levels and worse sleep. Additionally, self-reported assessments of underarm sensitivity, sweat, and malodour were negatively correlated with underarm wellbeing, indicating that increased frequency, rates and concerns in these areas are linked to decreased overall wellbeing. The 3 subscales were reduced from 75 items to 25 items with good reliability.

The UnderarmCARE Questionnaire is a valid tool for quantifying self-perceived underarm wellbeing and its impact on the quality of life of healthy individuals. It can be used to gain valuable insights into how body odour affects social and emotional wellbeing as well as to assess possible improvements produced by deodorant products to address these issues.

**Funding:** This study was funded by Unilever

**abstract citation id: bjaf055.124**

### Super-resolution shadow imaging reveals anatomical and morphological details of the mouse vomeronasal sensory epithelium

*Christoph Hamacher[1], Friederike Seifert[1], Marc Spehr[1]*

*[1]RWTH Aachen University. Germany*

In most mammals, the accessory olfactory system plays an important role in the detection and processing of both inter- and intraspecific chemical cues. The vomeronasal organ forms the system's peripheral sensory structure. It is a blind-ended tube, divided into three principal compartments: (i) a crescent-shaped mucus-filled lumen, into which stimuli are actively drawn by contractions of (ii) a lateral cavernous tissue, and (iii) a sensory epithelium that harbors vomeronasal sensory neurons (VSNs). Surprisingly, we still lack detailed information on many aspects of vomeronasal anatomy and VSN morphology. Here, we quantify and characterize morphometric features of the vomeronasal sensory epithelium, and VSNs in particular, in live tissue. We use super-resolution shadow imaging, a method that stains the extracellular space with fluorophores prior to super-resolution (STED) imaging. We then reconstruct and analyze the resulting shadow images both manually and with machine learning-based approaches. We investigate acute tissue slices on different levels. First, we assess macro-scale properties of the sensory epithelium, including general cell count and the cellular-to-interstitial volume ratio. Second, meso-scale analysis addresses VSN morphometry and its correlation with epithelial location. Third, we examine micro-scale features of subcellular VSN compartments, including soma, dendrite, and knob morphologies. Finally, we compare general morphometric features to those of cells expressing a specific receptor type. Together, these findings demonstrate distinct heterogeneity in shape and morphometric features among mouse VSNs.

**abstract citation id: bjaf055.125**

### A Cre-lox technique to address olfactory dopaminergic cell functional heterogeneity

*Ilaria Ghia[1], Stefano Zucca[1], Maddalena Pieroni[1], Sara Bonzano[1], Giulia Demenego[2], Simona Lodato[2], Paolo Marcello Peretto[1], Silvia De Marchis[1], Serena Bovetti[1]*

*[1]Dept. of Life Sciences and Systems Biology and Neuroscience Institute Cavalieri Ottolenghi University of Turin, Italy*

[2]*Humanitas University, Dept. of Biomedical Sciences and IRCCS Humanitas Research Hospital, Neurodevelopment Biology Lab. Italy*

The main olfactory bulb (OB) is the first and only relay structure for odors before reaching the olfactory cortex and hosts the most numerous dopaminergic (DA) forebrain population. These inhibitory interneurons are typically identified by tyrosine hydroxylase (TH) expression and two main subpopulations can be recognized based on soma size, electrophysiological properties, and generation time. Although increasing evidence places the role of these cells in the early stages of sensory processing, their exact function remains unclear. Moreover, their high heterogeneity challenges the study of these cells and thus a specific method to target the two subpopulations differentially is required to study their properties and roles separately. To this aim, we validated a virally delivered, time-controlled Cre-lox technique that allows to express either morphological or functional reporters in the OB TH-positive cells selectively. The specificity of this labelling is comparable to that of the TH-GFP transgenic mouse line and can target TH-positive subpopulations enriched in either small or large cells whether it is performed in adult mice or in embryos and newborns, respectively. By expressing GCaMP indicators in TH-positive cells, we were able to perform in vivo two-photon functional calcium imaging experiments in anesthetized, head- fixed adult mice. This allowed the assessment of their spontaneous activity as well as their evoked responses to opposite- sex urine. Since we previously observed a specific TH-positive cell recruitment in the posterior OB of female mice exposed to male urine, our goal is to focus on this area while observing TH-positive cell function using our labelling strategy. Taking advantage of a multidisciplinary approach based on this OB DA cell targeting technique, our main aim consists in dissecting a possible differential role for the two DA subpopulations in processing ethologically relevant odors, specifically those associated to sexual behaviors.

### References

1 Kosaka, T., & Kosaka, K. (2009). Two types of tyrosine hydroxylase positive GABAergic juxtaglomerular neurons in the mouse main olfactory bulb are different in their time of origin. Neuroscience Research, 64(4), 436-441.

2 Kiyokage, E., Pan, Y. Z., Shao, Z., Kobayashi, K., Szabo, G., Yanagawa, Y., . . . Shipley, M. T. (2010). Molecular identity of periglomerular and short axon cells. Journal of Neuroscience, 30(3), 1185-1196.

3 Galliano, E., Franzoni, E., Breton, M., Chand, A. N., Byrne, D. J., Murthy, V. N., & Grubb, M. S. (2018). Embryonic and postnatal neurogenesis produce functionally distinct subclasses of dopaminergic neuron. eLIFE, 7(e32373), 1-36.

4 De Marchis, S., Bovetti, S., Carletti, B., Hsieh, Y., Garzotto, D., Peretto, P. M., . . . Rossi, F. (2007). Generation of distinct types of periglomerular olfactory bulb interneurons during development and in adult mice: Implication for intrinsic properties of the subventricular zone progenitor population. Journal of Neuroscience, 27(3), 657-664.

5 Bonzano, S., Bovetti, S., Fasolo, A., Peretto, P. M., & De Marchis, S. (2014). Odour enrichment increases adult-born dopaminergic neurons in the mouse olfactory bulb. European Journal of Neuroscience, 40(10), 3450-3457.

**abstract citation id: bjaf055.126**

### Perceptual deviance processing in odour mixtures is different for foods and non-foods

*Leonie Seidel[1], Astrid E. Iversen[2], Janina Seubert[1]*

[1]*Karolinska Institutet. Sweden*
[2]*Copenhagen University. Denmark*

The perception of an unfamiliar note in a familiar odour mixture can vary by context. For example, we previously found that participants were more likely to perceive food as contaminated by non-food odours than vice versa. This effect was driven by perceptual amplification of non-food components, a bias that persists despite changes in attentional focus, and might represent a protective mechanism against accidental ingestion of contaminants. However, these processing differences between food and non-food odours, particularly our tolerance for unusual notes, are still not fully understood. In the current study, we explored the effects of attention on the perception of odour mixtures composed of only food or non-food components. Participants ($N = 24$) assessed the perceived dominance of a target odour within an odour mixture. Analysis of response times (RT) showed both a linear ($\beta = 0.14$, $p < 0.001$) and quadratic term ($\beta = -0.01$, $p < 0.001$) with fastest RT for pure odours and slowest RT around the dilution series' midpoint. Decisions were also faster for food odours compared to non-food odours ($\beta = 0.25$, $p < 0.001$). The likelihood of perceived target dominance generally increased with target concentration ($OR = 2.6$, $p < 0.001$), but followed different patterns for the two target categories. While p(dominant) increased linearly with rising target concentrations for non-food targets, it exhibited a sigmoidal shape with a steeper slope (larger just-noticeable difference, JND) for foods than for non-foods. These findings imply that distinguishing two food odours may be easier than distinguishing between two non-food odours, likely due to clearer definitions of object categories. Conversely, individuals might exhibit lower contamination sensitivity in non-foods, allowing for greater tolerance of deviations. This work was funded by the European Research Council (Grant agreement No. 947886 to JS).
**Funding:** European Research Council

**abstract citation id: bjaf055.127**

### Discrimination of intranasal electrical stimulation patterns and associative learning: neurophysiological and behavioral correlates

*Coralie Mignot[1], Susanne Weise[1], Mei Ren[1], Biranche Tandon[2], Halina Stanley[3], Moustafa Bensafi[3], Thomas Hummel[1]*

[1]*TU Dresden, Smell and Taste Clinic. Germany*
[2]*Microsystems Laboratory, École Polytechnique Fédérale de Lausanne (EPFL). Switzerland*

*[3]Université Claude Bernard Lyon 1, CNRS, INSERM, Centre de Recherche en Neurosciences de Lyon CRNL. France*

Using the trigeminal system as a substitution in the context of an olfactory implant has been proposed. This assumes that patients can differentiate between trigeminal stimuli and associate them with corresponding odorants.

The neural correlates of the perceptual discrimination between two patterns of intranasal electrical stimuli delivered at the septum were studied in healthy subjects and patients with olfactory loss using EEG. In the learning protocol, we examined whether the association between such patterns and the mental image of an odor would improve discrimination and recognition of these patterns. The protocol included four sessions: 1) an initial discrimination of the two electrical patterns, 2) & 3) 5-minutes associative learning phases, and 4) a final assessment of discrimination. To test discrimination, we gave subjects a random series of 24 triplets, each comprising two identical and one different electrical pattern, from each of which they had to choose the odd-one-out Preliminary results (4 patients, 7 controls) suggest that patients were less sensitive to electrical stimuli than controls, but they could differentiate electrical patterns. Indeed, the two electrical patterns were generally rated as different in terms of intensity, pleasantness and irritation (two-way Wilcoxon test, $p<0.04$). All participants performed the discrimination task above chance (two-way Wilcoxon test, $p=0.004$), even though 3 participants had a low performance and none had perfect discrimination. In the time-frequency domain, the baseline EEG assessment of intranasal electrical processing revealed significant differences of theta, delta and beta oscillations between patients and controls, and between electrical stimulation patterns (ANOVA, $p<0.05$).

In conclusion, healthy participants and patients are able to discriminate different types of electrical stimuli, which is also reflected at an electrophysiological level.

**Funding:** This project has received funding from the European Union's Horizon 2020 FET Open programme under grant agreement No 964529

**abstract citation id: bjaf055.128**

### State-dependent modulation of odor valence and social behavior via the main olfactory pathway

*Jana Marie Sleeboom[1], Ilona Grunwald Kadow[1], Annika Cichy[1]*

*[1]Institute of Physiology II, Faculty of Medicine, University Clinic Bonn (UKB), University of Bonn. Germany*

The perception of sensory stimuli depends not only on its physical properties but can be significantly modulated by the internal state, experience and context. The olfactory system provides an ideal model to study modulation of perception and behavior, as it is heavily innervated by major neuromodulatory centers and chemosensory communication drives essential behaviors.

Especially, odor valence is a critical feature as it elicits appropriate aversion and attraction—motivated behaviors crucial for survival. Odor valence can also be significantly modulated by factors including internal state (e.g., hormonal status). Despite its importance, the mechanisms underlying encoding and modulation of odor valence remain poorly understood. In particular, how innate odor valence is encoded and modulated by non-conditioned factors like internal state remains unclear. Here, we identified a naturally occurring switch in behavioral valence responses towards a volatile social odor that dependents on the neuroendocrine state. In this model, 1) the response of female mice to the male social cue trimethylamine (TMA) significantly depends on the estrus state; 2) This behavioral switch depends on a defined receptor, TAAR5, which is expressed in the main olfactory system and is specifically activated by TMA; 3) This switch in behavior is specific to TMA. This naturally occurring switch in odor valence provides a unique opportunity to study a non-conditioned, potentially inducible change in perception.

To identify neural circuits and molecular mechanisms underlying estrus-dependent modulation, we combine awake in vivo 2- photon calcium imaging with RNA sequencing of specific neuronal subtypes. Since valence responses to TMA are abolished in TAAR5-deficient females, we hypothesize that this receptor plays a critical role in social and reproductive behaviors. To test this, we developed a behavioral assay comparing wild-type and mutant females at different estrus stages interacting with males.

**abstract citation id: bjaf055.129**

### Insights into Adult Neurogenesis of Vomeronasal Sensory Neurons

*Terlau Lena[1], Paula Schoedder[1], Friederike Seifert[1], Christoph Hamacher[1], Stefanie Kurth[1], Kristin Serè[1], Martin Zenke[1], Marc Spehr[1]*

*[1]RWTH Aachen University. Germany*

Neuronal turnover in olfactory epithelia ensures reliable detection of environmental cues throughout the lifetime of an animal. Thus, adult neurogenesis in mammalian olfactory epithelia persists throughout the lifespan of an individual.

However, the precise physiological processes that govern adult neurogenesis in the vomeronasal organ remain elusive. Here, we begin to describe characteristics of neurogenesis in the mouse vomeronasal sensory epithelium. We label newly generated vomeronasal sensory neurons (VSNs) using a genetic approach: upon tamoxifen injection, VSN progenitor cells in Id2CreERT2 :: Rosa26R-tdTomato mice express tdTomato upon coincident Id2 promoter activity. Descendants of these cells are thus identifiable by red fluorescence. Using the Id2 proliferation and differentiation marker as a VSN lineage tracer, we describe the proportion of new-born neurons within the VSN population. We identify the spatial distribution and morphology of individual new-born neurons along with their age-dependent migration patterns within the sensory epithelium.

Furthermore, our results offer insights into VSN turnover-rates. Finally, by analysing co-expression of different marker proteins, we evaluate the differentiation and maturation state of new-born neurons at different timepoints post injection.

**abstract citation id: bjaf055.130**

### Investigating the regenerative potential of the mouse vomeronasal organ

*Paula Teresa Schoedder[1], Lena Terlau[1], Friederike Seifert[1], Christoph Hamacher[1], Stefanie Kurth[1], Marc Spehr[1]*

*[1]RWTH Aachen University. Germany*

The olfactory epithelium (OE) and vomeronasal organ (VNO) are continuously exposed to environmental insults such as toxins and pathogens. To maintain their vital functions both chemosensory epithelia depend on ongoing cellular turnover and robust regenerative mechanisms. Regenerative processes that maintain OE integrity, particularly the roles of globose basal cells (GBCs) and horizontal basal cells (HBCs), have been thoroughly described. By contrast, comparatively little is known about the regenerative potential of basal stem cell populations in the VNO. Here, we begin to investigate the regenerative dynamics of GBCs and HBCs in the VNO. Using immunohistochemical approaches, we (i) determine the abundance and proliferative capacity of GBCs and HBCs, (ii) describe their spatial distribution within the respective epithelia, and (iii) compare their relative proportions and localisation patterns. We aim to provide insight into whether and, if so, to what extent cellular mechanisms identified in the OE are conserved within the VNO. Together, these data enhance our understanding of epithelial maintenance and sensory neuron turnover in the vomeronasal system.

**abstract citation id: bjaf055.131**

### What makes a scent trigger a memory? A cognitive decomposition of odor-evoked retrieval

*Anne-Lise Saive[1], Juliette Greco-Vuilloud[1], Ruby Perrine[2], Jane Plailly[2]*

*[1]Lyfe Institute Research Center. France*
*[2]Lyon Neuroscience Research Center. France*

A single scent can unlock vivid memories. This study investigates the factors that make some odors more evocative than others. We examined odor-evoked episodic memory in 106 participants who experienced odors embedded in distinct visuospatial contexts, and whose memory was tested 24–72 hours later. The protocol empirically dissociates odor recognition ("I've already smelled this scent") and associative memory ("It evokes a memory") processes. Using machine learning with SHapley Additive exPlanations, we identified distinct predictors for each process. Recognition was driven by emotional strength – especially for unpleasant odors – and the richness of verbal descriptions. Associative memory followed a U-shaped relationship with familiarity and was strongly influenced by semantic distinctiveness – how uniquely each odor was described. Together, these findings reveal that odor memorability depends not only on its emotional salience, but also on how specifically it is conceptualized and how familiar we are with it.

Highlights: Dissociation of odor-evoked memory into recognition and associative processes. Emotional strength, especially unpleasantness, drives odor recognition. Familiarity follows a U-shaped relationship with associative memory. Semantic richness and uniqueness enhance odor-evoked memory retrieval

**Funding:** Juliette Greco-Vuilloud was founded by L'Oréal Research & Innovation.

**abstract citation id: bjaf055.132**

### Modulation of bulbar activity in a mouse model of impaired intrinsic excitability

*Sana Gadiwalla[1,2], Elisa Galliano[2], Petur Henry Petersen[1]*

*[1]Department of Anatomy, Biomedical Center, Faculty of Medicine, University of Iceland, Reykjavik Iceland*
*[2]Department of Physiology, Development and Neuroscience, University of Cambridge, Cambridge, United Kingdom; (2) Department of Physiology, Development and Neuroscience, University of Cambridge, Cambridge. United Kingdom*

Intrinsic excitability regulation is a critical yet understudied aspect of adaptive neuronal plasticity, essential for maintaining stable neural activity in response to perturbations. Uncovering the molecular regulators of intrinsic excitability modulation is challenging due to its dynamic, cell-specific nature and the interplay between synaptic and intrinsic mechanisms. In the olfactory bulb headway has been made by discovering that the microphthalmia-associated transcription factor (Mitf) is specifically expressed in projection neurons (PN, mitral and tufted cells) and that in primary culture of knock-out mutant (MT) mice, the loss of Mitf results in hyperactive PNs. Using immunohistochemistry and cell whole cell patch clamp in acute slices this project assessed the role of Mitf in the transcriptional control of intrinsic excitability in bulbar PNs in a model where circuitry is maintained.

Compared to WT controls, Mitf mutants PNs have shorter axon initial segments (WT 25.42±0.4μm; MT 23.05±0.7μm; t-test $p=0.004$) that are closer to the soma (WT 4.7±0.2μm; MT 3.7±0.2μm; t-test $p<0.001$). These structural changes are alignment with the physiological hyperexcitability of MT PNs.

In current clamp, MTs fired more action potentials (WT 32.1 ±5.4; MT 51.0±4.1; Mann-Whitney p=0.013) with shorter latency to fire (WT 23.6±3.1ms; MT 16.9±0.2ms; Mann-Whitney p=0.029). Moreover, after 30 minutes of home-cage exposure to 8 odours before slice preparation, Mitf MTs showed altered gain control and transcription of excitability-related genes compared to control littermates. Together these results offer a new perspective over the transcriptional control of homeostatic mechanisms that contribute to excitability.

**Funding:** Icelandic Research Fund Project Grant 217945-051 (EG, PHP); Icelandic Research Fund Doctoral Student Grant 2511469-051 (SG); BBSRC Project Grant BB\W014688\1 (EG)

**abstract citation id: bjaf055.133**

### Blood biomarkers predict idiopathic taste dysfunction: a case- control study

*Mariano Mastinu[1], Stephan Habiger[2], Siegfried Heckmann[3], Thomas Hummel[1]*

[1]*Smell & Taste Clinic, TU Dresden. Germany*
[2]*Dental Practice, Hof. Germany*
[3]*Dept. of Dental prosthetics and materials science, University Clinic of the Saarland, Homburg. Germany*

Taste dysfunction affects quality of life, yet its biological development remains poorly understood. This study explored differences in blood biomarkers between participants with and without taste dysfunction. We investigated clinical and biochemical differences in 50 patients with idiopathic dysgeusia and 102 healthy controls (110 women, 42 men; mean age 46.4 ±1.4 years).

Following a detailed medical history, each participant underwent taste testing and blood analysis for iron, copper, zinc levels and iron metabolism (ferritin, transferrin), serum electrolytes, hematocrit, complete blood count, transaminases, creatinine, complement C3/C4 and ANA titers, using atomic absorption spectrometry among others.

Patients with idiopathic dysgeusia, who were older (61 vs. 39 years; p<0.001) and predominantly females (86 vs. 66%; p=0.009), were compared to healthy controls using age-adjusted statistical analysis. Taste performance assessed by “Taste Strips” was lower in patients, particularly for sour, bitter, and salty modalities (p<0.001). They also reported higher scores on depression (Beck Depression Index) and mood questionnaires (Zerssen Mood Scale; p<0.001). Blood analyses revealed that patients had higher levels of complement C4 (p=0.011) and calcium (p=0.019; ANCOVA), and a trend toward lower potassium (p=0.054). A logistic regression model including age, gender, C4, Ca, K, Na, and transferrin predicted taste dysfunction with high accuracy ($R^2$=0.742; 91.4% correctly classified). Increased age, higher C4, Ca, and Na were associated with higher odds of taste dysfunction, while male gender, higher potassium, and transferrin levels were protective factors (p≤0.030).

These findings suggest that idiopathic taste dysfunction is accompanied by systemic immune imbalance while lower level of electrolytes may reflect a metabolic imbalance. These parameters should be included in clinical assessment. Further research is needed to explore causal mechanisms and clinical implications.

**abstract citation id: bjaf055.134**

### Foul and fragrant: differential naming of disgust eliciting odors

*Bea van den Berg[1], Ilja Crojimans[1], Josh Tybur[2], Monique Smeets[3]*

[1]*Radboud University. The Netherlands*
[2]*Vrije Universiteit Amsterdam. The Netherlands*
[3]*University of Utrecht. The Netherlands*

Being able to efficiently communicate about potential harmful stimuli in the environment is beneficial for survival. Therefore, disgusting odors, e.g., signaling danger and/or pathogens in the environment, are expected to be easier to describe compared to neutral or positive control odors. To test if disgust inducing odors are more nameable, the current study analyzed free responses to disgusting and control odors collected by Tybur et al. (2022). In Tybur et al. (2022), 5 disgusting and 5 control odors were described twice by 119 Dutch participants. We hypothesized that disgusting odors had (a) a higher within-participant consistency and (b) longer responses, both indicating more proficient language use. Binary logistic regression showed that odor type significantly predicted within- participant consistency. Specifically, the odds of within-participant consistency occurring was 41% higher for disgust inducing odors compared to control odors. Thus, disgusting odors are described more consistent than control odors. Furthermore, linear regression showed that disgust inducing odors ($M$ = 73.28, $SD$ = 38.60) had a significantly longer response (measured with number of characters) than control odors ($M$ = 64.74, SD = 34.82, $p$ < .001). Thus, participants had significantly longer responses to disgusting odors. Exploratory analyses showed what terminology was used for the different odor types. As expected, source terms were used the most. Additionally, binary logistic regression showed that odor type did not predict the use of source or abstract terms. Odor type did predict the use of evaluative terms, where disgusting odors were more likely than control odors ($b$ = 0.96, $p$ < .001). Odor type also predicted responses that showed uncertainty, where control odors were less probable than disgust inducing odors ($b$ = -0.55, $p$ < .001).

To conclude, disgust inducing odors were described more consistently and with a longer response compared to control odors.

### Reference

1 Tybur, J. M., Croijmans, I. M., van Huijstee, D., Çınar, Ç., Lal, V., & Smeets, M. A. (2022). Disgust sensitivity relates to affective responses to–but not ability to detect–olfactory cues to pathogens. Evolution and Human Behavior, 43(4), 284-295.

abstract citation id: bjaf055.135

### Differential regulation of neurofunctional and synaptic genes in the heads of Apis mellifera infected with Deformed Wing Virus (DWV-A)

*Diego Eduardo Silva Vasquez[1], Marisol Vargas Concha[1], Lucila Traverso[2], Sheila Ons[2], Yazmin Fuentes[1], Felipe Becerra[1], Carolina Gomez Diaz[3]*

*[1]Laboratorios de Virología y Neurociencia en Abejas, Facultad de Agronomía, Universidad de Concepción, Chillán. Chile*
*[2]Laboratorio de Neurobiología de Insectos. Centro de Endocrinología Experimental y Aplicada - Centro Regional de Estudios Genómicos. Facultad de Ciencias Exactas y Facultad de Ciencias Médicas. UNLP-CONICET, La Plata, Buenos Aires. Argentina*
*[3]Instituto de Neurociencias del Principado de Asturias (INEUROPA), Facultad de Medicina y Ciencias de la Salud, Universidad de Oviedo, Asturias. Spain*

The Deformed Wing Virus (DWV-A) is one of the most prevalent pathogens affecting *Apis mellifera* colonies and has been linked to impairments in bee behavior and neural function, altering the insect's cognitive capacity. In this study, we analyzed the gene expression profile in the heads of bees with high viral loads (I-DWV; $1 \times 10^{11}$ viral genome copies per bee) compared to those with low viral loads (N-DWV; $1 \times 10^{3}$ viral genome copies per bee), using RNA sequencing (RNA- Seq). Over 145 million reads were obtained, with an average mapping rate of 78.94%. A total of 9,113 transcripts were examined, identifying 147 differentially expressed genes (DEGs) (s-value < 0.05), of which 109 were upregulated and 38 downregulated in I-DWV bees. Gene Ontology (GO) enrichment analysis revealed 23 significantly associated terms, highlighting cellular processes related to intercellular communication and transmembrane transport. Specifically, we detected upregulation of genes associated with glutamatergic signaling, including Neuropilin and tolloid-like (Neto), Glutamate receptor ionotropic, kainate 2 (LOC102653800), Excitatory amino acid transporter 2 (Eaat2), and Sodium-dependent nutrient amino acid transporter 1 (Naat1), while Anoctamin-4 was downregulated. Additionally, Pfam domain analysis identified 77 enriched domains, highlighting those related to synaptic functions (CUB domain, receptor ligand- binding domain) and transcription factors associated with the nurse-to-forager physiological transition (Helix-loop-helix domain). In contrast, domains associated with stress responses (TPR and Hsp70) showed decreased expression. These results suggest that DWV-A infections profoundly affect the gene regulation of key processes in the nervous system of *A. mellifera*, including neuronal signaling, immune response, and functional plasticity, with potential repercussions on behavior and colony social organization.
**Funding:** This study was supported by the FONDECYT N 1241994 grant from the National Research and Development Agency (ANID), Chile; the National Agency for Research and Development (ANID), Chile, through the National Doctoral Scholarship (No. 21201234); Programa de Doctorado en Ciencias de la Agronomía, Facultad de Agronomía, Universidad de Concepción, Chillán, Chile.

abstract citation id: bjaf055.136

### Location matters: mucus moves slower in the olfactory cleft than at the lateral nasal wall

*Anna Kristina Hernandez[1], Karoline Lantzsch[1], Sero Biguerdi[1], Katharina Schindowski[2], Nadine Gunder[1], Thomas Hummel[1]*

*[1]Smell and Taste Clinic, TU Dresden. Germany*
*[2]Biberach University of Applied Science. Germany*

**Objectives:** To assess and compare intranasal mucus movement based on location (olfactory cleft [OC] versus the lateral nasal wall at the level of the attachment of the middle turbinate [MT]) and material (particulate matter - silver flakes vs. liquid - methylene blue gel).
**Methods:** In this cross-sectional study, 59 healthy, normosmic adults (38 women, median age: 24 years) were evaluated. Mucus movement was endoscopically observed (30° Hopkins endoscope, Karl Storz, Tuttlingen, DE) and video-recorded with participants seated upright. Silver flakes (~1-2mm$^2$) or one drop of methylene blue dye in Xylometazoline hydrochloride nasal gel (Nasengel AL 1mg/g, Aliud Pharma, Lachingen, DE) were applied to the olfactory cleft (silver flakes only) and the base of the MT (both materials) using a microsuction tube, without prior nasal decongestion or anesthesia. Olfactory function was assessed using the "Sniffin' Sticks" Odor Identification Test (Burghart, Holm, DE).
**Results:** Silver flakes placed on the MT exhibited movement within 2 minutes, whereas flakes in the OC showed minimal to no movement even after 7 mins ($\chi^2$ =19.9, p<0.001). Mucus movement from the MT was predominantly in an anterior-inferior direction, in contrast to the OC where movement was absent or minimal ($\chi^2$ =29.9, p<0.001). Among participants assessed with both materials at the MT (n=24), 58% showed consistent anterior-inferior movement (Fisher Freeman Halton Test=17.1, p<0.02).
**Conclusions:** Consistent with previous studies, mucus at the lateral nasal wall moves rapidly (within 2 minutes) in an anterior-inferior direction, whereas mucus over the olfactory cleft remains largely stationary even after 7 minutes. These findings demonstrate distinct regional differences in mucociliary movement within the nasal cavity.
**Funding:** This work was supported by a grant from the Deutsche Forschungsgemeinschaft (DFG HU441/27- 1) for T.H. and A.K.H., (DFG ZI- 1143) for K.S. and the Volkswagenstiftung (project PERCEPTRONICS, Az 9B396) for T.H.

abstract citation id: bjaf055.137

### Decoding the bitterness of food-derived peptides

*Silvia Schaefer[1], Alexandra Steuer[1], Laura Sophie Eckrich[1], Antonella Di Pizio[1], Corinna Dawid[1], Maik Behrens[1]*

[1]*Leibniz Institute for Food Systems Biology at the Technical University of Munich. Germany*

With the increasing world population, the demand for nutritious foods is rising. One partially essential macronutrient is protein and hence are peptides and amino acids; nevertheless, it was found that a variety of peptides and amino acids elicit an aversive bitter taste, diminishing the food's quality and consumers´ acceptance. Generally, bitter taste initiates an innate aversive reaction to avoid the ingestion of potentially pharmacologically active substances. In humans, about 25 G protein- coupled receptors are responsible for sensing bitter taste. Five of these bitter taste receptors were identified as bitter peptide receptors, namely TAS2R1, -R4, -R14, -R39, and -R46 with tritryptophane being a potent activator. As bitter-tasting peptides are abundant in a plethora of foods such as dairy, or plant products, it is crucial to understand the reason behind this off-taste to increase food quality.

In our work, with the usage of functional cell assays, we were able to show, that, starting from the potent bitter peptide tritryptophane, an extension of the peptide chain with further tryptophanes modifies the receptor response. Also the position of the amino acid tryptophane either on the C- or N-terminus is important for human bitter taste activation. Further, we were able to identify a bitter peptide motive in the off-tasting Gouda peptide YPFPGPIHN as well as the corresponding activated human bitter taste receptor. Multidisciplinary cooperation between computational, sensory, and *in vitro* approaches enabled us to generate and verify a prediction model for the bitterness of peptides. All in all, we managed to identify several factors that contribute to the off-taste of food-derived peptides, such as length, amino acid composition, and position. These insights, will help to increase the quality and acceptance of nourishing foods.

**Funding:** Funding was provided by the Deutsche Forschungsgemeinschaft (DFG) (BE 2091/7-1, PI 1672/3-1, DA 2112/3-1).

**abstract citation id: bjaf055.138**

### Stress-associated and neutral human body odors differ in their effects on facial emotion recognition

*Josephine Alexandra Noel[1], Franny B. Spengler[1], Jessica Freiherr[2], Markus Heinrichs[1]*

[1]*Laboratory for Biological Psychology, Clinical Psychology, and Psychotherapy, Department of Psychology, University of Freiburg, Freiburg, Germany*
[2]*Sensory Analytics & Technologies, Fraunhofer Institute for Process Engineering and Packaging IVV, Freising, Germany*

The transmission of social signals via olfactory cues (i.e. chemosensory signaling) is essential for social communication across numerous species. Yet, surprisingly little is known about the effect of chemosensory signaling on human social behavior. While recent evidence suggests that human body odors convey socially relevant information such as emotional states, the precise socio-affective processes mediating the potential effects of human chemosignals on social behavior remain elusive. In this study, we investigated how exposure to human body odors affects emotion recognition from facial expressions - a key component of effective social communication. A sample of 37 healthy male participants performed an emotion recognition task while being exposed to three different chemosensory conditions in a within-subjects design: stress body odor (sampled from an independent male sample during the Trier Social Stress Test for Groups, TSST-G), neutral body odor (sampled during the control condition of the TSST-G) and a non-human control odor (damped cotton pad without human body odor). Face stimuli varied in emotional valence (anger, happiness, neutral) and intensity (low, high), and participants indicated by button press whether they perceived an emotional state in each presented face. Preliminary results suggest that exposure to neutral body odors improved overall emotion recognition. Additionally, participants' anxiety seems to modulate the effect of body odors on emotion recognition. These findings highlight the role of chemosensory signals in human social cognition and behavior and may encourage further research to investigate the effect of chemosensory communication in clinical conditions associated with altered social interaction and emotional processing.

**Funding:** This research was funded by the University of Freiburg.

**abstract citation id: bjaf055.139**

### Can eating creative food make you more creative?

*Alberte Seeberg[1], Nurdin Topham[1], Michael Bom Frøst[1], Qian Janice Wang[1]*

[1]*University of Copenhagen. Denmark*

Surely most of us recognize the phrase 'You are what you eat'. However, is this also true when eating creative food? This study explores whether creatively prepared food - defined by novel combinations of ingredients, visually striking presentations, or distinctive taste profiles - can enhance cognitive creativity compared to conventionally presented dishes.

A total of 102 healthy participants were randomly assigned to consume either a creatively prepared food sample (fruit/vegetable lasagna) or a less creative but nutritionally identical version (fruit/vegetable purée). Participants completed two standard creativity tasks for assessing convergent and divergent thinking, the Remote Associates Test (n correct out of five) and the Alternative Uses Task (fluency, n different uses; flexibility, semantic distance from prompt word), both before and after eating.

Contrary to our initial hypothesis, only participants who consumed the low-creativity sample showed an increase in performance on the flexibility measure of the Alternative Uses Task. We speculate that this counterintuitive result may be linked to the violation of hedonic expectations: the familiar appearance of the purée might have led to strong prior expectations that were then disrupted by the taste profile of the sample, resulting in a prediction error. Such surprise or cognitive dissonance could have transiently boosted divergent thinking.

These findings suggest that the cognitive effects of food presentation may depend less on novelty per se and more on how presentation interacts with consumer expectations. In this view, surprise, rather than creativity, may be the key mechanism by which food can modulate cognition.

**Funding:** This study was funded by the Novo Nordisk Foundation (NNF23OC0082731).

**abstract citation id: bjaf055.140**

### A transformer-based approach to predict cellular organization principles in iPSC derived olfactory organoids

*Ferhat Hamurcu[1], Moritz Klingenstein[1], Stefanie Klingenstein[1], Stefan Liebau[1]*

*[1]Institute of Neuroanatomy and Developmental Biology, Eberhard Karl University of Tübingen. Germany*

The advantages of employing three dimensional in-vitro culture systems come with the price of varying cytoarchitectural as well as protein expression heterogeneity. Thus, organoid to organoid as well as batch to batch intervariability in differentiation outcomes following the same protocols remains a central challenge in developmental biology. Our lab-intern grown induced pluripotent stem cell-derived olfactory placode organoids vary in their volume, cellular organization as well as their protein expression patterns. Immunofluorescence staining remains as a method to truly assess and confirm the cellular identities of cell cultures.

In this study, we established a pure Vision-Transformer based approach to accurately predict the cellular identities of olfactory placode organoids, which were categorized into three classes for the expression of (A) epithelial, (B) neural and (C) olfactory placode protein markers based on bright-field images which were acquired beginning on 12 of differentiation and later validated using immunofluorescence staining of 5 key protein markers on single organoid level. We fine-tuned 10 different pre-trained Transformer-based architectures on a training data set of 300 bright-field images of organoids, on which we performed on-the-fly augmentation. The data was each split into 5 folds to perform 5-fold cross validation.

The ensemble model of the vision transformer predicted the never before seen test data set of organoids with an overall accuracy of 73,3%, far exceeding the independent expert group with 57,21% tasked with labelling the same bright-field images of organoids on day 12 of differentiation.

Our study suggests that a Vision Transformer–based model can reliably infer early cell fate trajectories in olfactory placode organoids by offering a label-free, non-invasive alternative to traditional immunostaining, enabling early prediction of differentiation outcomes as early as day 12.

**Funding:** University of Tübingen; Interdisziplinäres Promotionskolleg Medizin, University of Tübingen

**abstract citation id: bjaf055.141**

### Forest vs. urban environment exposure is associated with improved well-being and compositional nasal microbiome changes

*D. Connor Lashus[1], Andres Gomez[2], Thomas Hummel[3], Lucia F. Jacobs[4], Asifa Majid[5], Ravikiran M. Raju[6], Caroline J. Smith[7], Gregory N. Bratman[8]*

*[1]School of Environmental and Forest Sciences, University of Washington, Seattle WA 98195. United States*
*[2]Department of Animal Science, University of Minnesota, St. Paul, MN 55108. United States*
*[3]Interdisciplinary Center Smell & Taste, Department of Otorhinolaryngology, Faculty of Medicine Carl Gustav Carus, Technische Universität Dresden. Germany*
*[4]Department of Psychology, University of California, Berkeley, Berkeley, CA 94720. United States*
*[5]Department of Experimental Psychology, University of Oxford. United Kingdom*
*[6]Department of Brain and Cognitive Sciences, Picower Institute for Learning and Memory, Massachusetts Institute of Technology, Cambridge, MA; Division of Newborn Medicine, Harvard Medical School, Boston Children's Hospital, Boston, MA. United States*
*[7]Department of Psychology and Neuroscience, Boston College, Chestnut Hill, MA. United States*
*[8]School of Environmental and Forest Sciences, University of Washington, Seattle, WA 98195. United States*

The benefits of nature contact for human well-being are well-recognized, yet much remains to be understood about the underlying causal mechanisms. This pilot study employed a natural experimental design with students at the University of Washington in Seattle, WA (USA) to explore the potential role of the nasal microbiome in mediating psychological well-being improvements associated with an 8-week immersion in a forest vs. urban environment. After the academic year, during which all participants (N=13) were full-time students in Seattle, one group of students relocated to remote forest sites in western Washington for 8 weeks (N=5; forest condition), while another group of students remained in urban Seattle (N=8; urban condition). Self-reported affect and psychological well-being were assessed pre- and post-exposure using validated surveys, and nasal swabs were collected pre- and post-exposure for bacterial 16S rRNA gene sequencing.

The forest group exhibited significantly greater improvements in affect ($p < 0.05$) over the 8-week exposure relative to the urban group. No between-group differences in overall nasal bacterial community composition were detected pre-exposure, but significant compositional divergence was observed post-exposure ($p < 0.01$), with the forest group exhibiting higher bacterial richness than the urban group ($p < 0.05$). The forest – but not urban – group also exhibited significant enrichment ($FDR < 0.05$) over time in bacterial genera with established links to well-being, including Bifidobacterium and Akkermansia. Increases in bacterial richness and the relative abundance of these genera were significantly associated ($p < 0.05$) with affective improvements over time. These findings provide evidence that suggests the nasal microbiome may be part of the mechanism through which nature contact benefits psychological well-being.

**Funding:** This work was supported by funding from University of Washington's Center for the Studies of Demography and Ecology.

**abstract citation id: bjaf055.142**

### Odor naming as a tool for cognitive and perceptual assessment

*Thomas Hörberg[1], Jonas Kristoffer Olofsson[1], Maria Larsson[2], Murathan Kurfalı[3], Erika Jonsson Laukka[4]*

*[1]SCI-lab, Gösta Ekman Laboratories, Department of Psychology, Stockholm University. Sweden*
*[2]Gösta Ekman Laboratories, Department of Psychology, Stockholm University. Sweden*
*[3]RISE Research Institutes of Sweden*
*[4]Aging Research Center, Department of Neurobiology, Care Sciences and Society, Karolinska Institutet and Stockholm University. Sweden*

Tests of cued odor identification (OID) are often used as an assessment tool in cognitive, neuropsychological, or aging research. Due to the difficulty of naming odors, free OID (i.e. odor naming) is less frequently used. When free OID is used, a binary scoring model (i.e. correct or incorrect) is generally applied to each trial. This approach ignores the additional information available in odor misnamings (e.g., naming strawberry odor as peach) and odor naming omissions (i.e., complete failure to provide a name). Using data from a large-scale dataset collected in Sweden (SNAC-K; n=2280), we show that odor misnamings contain important information about olfactory-perceptual and olfactory-semantic abilities, and that free OID draws on a broader range of cognitive abilities than cued OID. A high frequency-of-use of odor misnamings that are specific and concrete (e.g., cinnamon) rather than vague and abstract (e.g., sweet), that are low in frequency, and that are semantically similar to the target odor name is associated with better OID performance. Further, whereas cued OID performance only is associated with an estimate of perceptual ability, perceptual speed, free OID outcomes (i.e., correct namings, misnamings and omissions) are additionally associated with verbal fluency, vocabulary, and episodic memory proficiency. We suggest that free OID / odor naming is an underutilized tool for cognitive and perceptual olfactory assessment, especially if the full breadth of the data available in correct namings, misnamings and omissions can be harnessed.

**Funding:** This work was funded by the Swedish Research Council (2021-03440; 2021-00178; 2020-00266); the Knut and Alice Wallenberg Foundation (2016:0229), and The Swedish e-Science Research Center.

**abstract citation id: bjaf055.143**

### Machine learning of an electronic nose as a tool, to monitor key biomarkers of food spoilage

*Lucia Lazaro De la escalera[1], Mónica Delgado[1], J. Iñaki Alava[1], Emily Ortega[1]*

*[1]Basque Culinary Center. Spain*

**Introduction:** The main objective of this article is to demonstrate the capability of the electronic nose made from carbon nanotubes to detect trimethylamine (TMA). For the tests, the "Smell Inspector" device was used — a prototype electronic nose developed by the company SmartNanotubes. The device is accompanied by software for data processing, which also enables the creation of predictive models using artificial intelligence.

**Material and Methods:** Trimethylamine (Fisher Thermoscientific) was chosen as the final marker of putrefaction. An initial stock dilution (1:1000) was prepared, from which further dilutions ranging from 1:9000 to 1:10,000 were made. These were placed in Erlenmeyer flasks with a mouth diameter of 4 cm and a volume of 1 L (Kriz, P. et al., 2023; Peters, R., et al., 2023; SmartNanotubes, 2022), and exposed to the sensors for 5 minutes, generating the data needed for training and subsequent odor recognition by the device. With training at different concentrations, it is expected that a portable device capable of real-time prediction of compound presence can be developed.

**Results:** Graphs show blank and trimethylamine dilutions data acquired by device at different TMA perception channels.

**Conclusions:** This study demonstrates that, although much work remains to be done, the electronic nose has the potential to become an excellent method for quality control, especially in fish markets. This work has been fully funded by the Basque Culinary Center.

**Funding:** This work has been fully funded by the Basque Culinary Center.

### References

1 Kriz, P., Sikora, P., Riha, K., Burget, R., & 2023 15th International Congress on Ultra Modern Telecommunications and Control Systems and Workshops (ICUMT) (2023). Unveiling the Smell Inspector and Machine Learning Methods for Smell Recognition. In 2023 15th International Congress on Ultra Modern Telecommunications and Control Systems and Workshops (ICUMT) (pp. 182–187). https://doi.org/10.1109/ICUMT61075.2023.10333105

2 Peters, R., Ruud and Beijer, N., Niels and 't Hul, B., Bauke van and Bruijns, B., Brigitte, B., and Munniks, S., and Knotter, J., "Evaluation of a Commercial Electronic Nose Based on Carbon Nanotube Chemiresistors," in Sensors, vol. 23, no. 11, 2023. https://doi.org/10.3390/s23115302

3 Technical manual Smell Inspector, SmartNanotubes Technologies, Dresden, Germany, June 2022. Accessed: Jun. 25, 2023. [Online]. Available: https://smart-nanotubes.com/wp-content/uploads/2023/07/SNT_Manual_Smell-inspector_V1.3.1.pdf

**abstract citation id: bjaf055.144**

### Inputs to dopaminergic neurons in the mouse olfactory bulb

*Ailie McWhinnie[1], Maggy Lau[1], Menghon Cheah[2], Li Huang[1], Emily Winson-Bushby[2], Matthew Grubb[2], Elisa Galliano[1]*

*[1]Department of Physiology, Development and Neuroscience, University of Cambridge, CB2 3DY Cambridge, United Kingdom*
*[2]Centre for Developmental Neurobiology, Institute of Psychiatry, Psychology & Neuroscience, King's College London, London, SE1 1UL, United Kingdom. United Kingdom*

Dopaminergic (DA) neurons in the murine olfactory bulb (OB) modulate the gain of the first synapse in the olfactory chain through the release of GABA and dopamine. They can be cleanly classified into two subtypes with distinct morphological and physiological properties: one axon-bearing and embryonic-born, and one anaxonic and regenerating. These are hypothesised to mediate odour discrimination and sensitivity, respectively, but understanding their distinct contributions to olfactory behaviour relies on knowing the inputs they receive and how they in turn modulate olfactory circuitry.

To understand the landscape of olfactory input to DA neurons, we used rabies tracing to map and reconstruct its presynaptic partners. This revealed cells of variable morphology within each OB layer and in the olfactory cortex, suggesting diverse inputs to DA neurons, including top-down influences.

Patch clamp experiments allowed us to look at these inputs functionally and to separate the two DA subtypes. Axon-bearing DA neurons receive more excitatory inputs, including more from ETCs. While the frequency of inhibitory inputs is the same across both subtypes, the much smaller size of anaxonic neurons means they receive a significantly higher density of inhibitory input and, accordingly, the addition of a GABA blocker appears to be more impactful on their activity than it is for the larger axon-bearing neurons.

**Funding:** PDN-Wolfson PhD Studentship (AM), URKI Biotechnology and Biological Sciences Research Council BB \W014688\1 (EG)

**abstract citation id: bjaf055.145**

### Olfactory substitution by the trigeminal system in humans: a promising therapeutic approach

*Susanne Weise[1], Halina B Stanley[2], Clémentine Sophie Sarah Lipp[3], Thomas Hummel[1]*

*[1]Smell & Taste Clinic, Department of Otolaryngology, Technische Universität Dresden, Dresden, Germany*
*[2]Université Claude Bernard Lyon 1, CNRS, INSERM, Centre de Recherche en Neurosciences de Lyon CRNL U1028 UMR5292, NEUROPOP, F-69500, Bron, France*
*[3]Microsystems Laboratory LMIS1, École Polytechnique Fédérale de Lausanne (EPFL), Lausanne 1015, Switzerland*

**Introduction:** Olfactory dysfunction (OD) affects around one-fifth of the population, which can lead to a diminished quality of life. Limited treatment options and recent advances in technologies such as artificial noses are moving olfactory implants into closer reach. Trigeminal stimulation offers a promising, less invasive strategy targeting neural pathways closely interlinked with the olfactory system. This multicentric study aimed to assess the sensitivity of patients with OD to electrical stimuli and their ability to discriminate between different patterns of stimulation.
**Method:** The first study compared the electrical intranasal sensitivity in patients with OD (n=54) of different etiologies and healthy individuals (n=28). A second study involving patients with OD (n=52) and healthy individuals (n=13) assessed their ability to detect and discriminate two patterns of electrical stimuli, which were triggered by a coupled electronic nose.
**Results:** The first study revealed a preserved trigeminal function for the majority of patients with OD. The second study showed that >70% of the patients were able to discriminate between two stimulation patterns. However, 100% of the participants were able to detect the stimuli.
**Discussion:** Trigeminal stimulation could be a promising option to substitute the olfactory system in patients with OD.
**Funding:** This Project has received funding from the European Union's Horizon 2020 research and innovation programme under grant agreement No 964529 (Pathfinder Rose project).

**abstract citation id: bjaf055.146**

### Odor exposure modulates pain perception: insights from behavioral and neural evidence

*Yiling Mai[1], Coralie Mignot[1], Michal Pieniak[1], Antje Haehner[1]*

*[1]Smell and Taste Clinic, TU Dresden. Germany*

**Objectives:** This study examined whether odors that trigger non-painful cooling trigeminal sensations modulate experimentally induced pain, and compared the effects between odors that induce olfactory versus cooling trigeminal sensations.
**Methods:** Using a within-subject design, we included 30 healthy adults (24.9±4.4 years, 17 women). Participants had a 30-minute odor exposure by wearing a nasal clip filled with one of the three conditions: phenyl ethyl alcohol (PEA), menthol, or an odorless control. After 30 minutes, pain perception was measured using pulsatile electrical stimulation, and electroencephalography (EEG) recordings were taken in response to painful electrical stimuli. The procedure was performed for all three odor conditions in a randomized sequence.
**Results:** There was a significant odor effect on pain tolerance ($F=5.20$, $p<0.01$), with participants showing higher pain tolerance after exposure to menthol and PEA than control ($t=2.42$-$3.16$, p's<0.05). EEG results showed similar patterns (F=3.61-6.17, p's<0.03), with significantly smaller event-related potential (ERP) amplitudes for PEA (P2, N1P2) and menthol (N1, P2, N1P2) than control (t=2.53-3.19, p's<0.05). Time-frequency analysis showed that in the first 500ms in the delta/theta band, both menthol and PEA induced less synchronization than control. Approximately between 400-700ms, only PEA showed significantly more synchronization in the alpha/beta band than control. From around 750-1500ms, all conditions showed desynchronization in the delta band, but PEA had significantly less desynchronization than control.

**Conclusions:** Short-term exposure to "olfactory" and cooling "trigeminal" odors can reduce pain perception at behavioral levels which is also reflected in brain activity. Menthol and PEA show similar effects but may involve different mechanisms. Menthol might primarily modulate pain in the early stage, while PEA appears to modify processing over a prolonged period of time.

**Funding:** This research was supported by a grant from the Deutsche Forschungsgemeinschaft to TH (DFG HU441/27-1)

**abstract citation id: bjaf055.147**

### Impact of genetic polymorphism on TAS1R2/TAS1R3 receptor activation by sweeteners

*Christine Belloir*[1], *Mathilde Jeannin*[1], *Adeline Karolkowski*[1], *Loïc Briand*[1]

[1]*INRAE. France*

The sweet taste receptor, a heterodimer composed of TAS1R2 and TAS1R3 subunits, detects chemically diverse sweet-tasting compounds. Genetic variations in these genes, particularly single-nucleotide polymorphisms (SNPs), have been associated with altered sweet taste perception and metabolic phenotypes. However, their functional consequences on receptor activation remain incompletely characterized. This study evaluates the effect of 28 coding-region SNPs (12 in TAS1R2, 16 in TAS1R3) on receptor activation by 12 structurally diverse sweeteners.

Using calcium mobilization assays with HEK293T cells transiently expressing wild-type or variant of TAS1R2/TAS1R3 receptors, dose-response relationships were quantified ($EC_{50}$ and maximal amplitude). Additionally, 162 DNA samples from overweight/obese individuals were genotyped to identify novel SNPs predicted to alter receptor function.

Our functional assays revealed that I595T and K689Q variants of TAS1R2, as well as L95P, M110T, G367C, S551N, and F749S variants of TAS1R3, significantly reduced receptor activation for several sweeteners. Conversely, R813K and G832R variants of TAS1R3 increased receptor sensitivity to the studied sweeteners. Seven previously unreported SNPs found in the obese cohort were predicted to alter receptor function.

Despite these in vitro functional alterations, no consistent genotype-phenotype association emerged for sweet taste preference or obesity. These results suggest that although specific TAS1R2 and TAS1R3 variants modulate receptor sensitivity, sweet taste perception and sugar intake are multifactorial traits influenced by additional genetic, environmental, and behavioral determinants.

In conclusion, this comprehensive functional analysis elucidates the impact of variants encoding TAS1R2/TAS1R3 on sweet taste receptor activity and highlights the complexity underlying genotype-phenotype relationships in metabolic regulation.

**Funding:** This project received funding from the EU H2020, grant agreement no. 774293.

**abstract citation id: bjaf055.148**

### Odor effects on sleep quality: evaluating odor quality and application methods

*Zetian Li*[1], *Jean-Christophe Gerami-Manesch*[1], *Lisa Hoehnel*[1], *Jonathan Warr*[2], *Antje Haehner*[1], *Thomas Hummel*[1]

[1]*Smell & Taste Clinic, Department of Otorhinolaryngology, Faculty of Medicine Carl Gustav Carus, Technische Universität Dresden. Germany*
[2]*Takasago Europe, Paris, France*

**Purpose:** The role of smell in improving sleep quality is inconclusive. This study aimed to investigate whether the type of odor and the method of application affect sleep quality.

**Methods:** Based on individual preference 113 healthy participants (mean age ± SD = 32 ± 12 years, 44 men) selected an odor (lavender, orange, specifically designed "perfume") and an application method (odorized nasal clips, odors on pillow, odors through diffuser). Their performance was compared to a control group (n = 19; mean age ± SD = 29 ± 6 years, 9 men). Except for the control group, all groups slept with exposure to odor for 2 weeks (phase 1), followed by 2 more weeks of normal sleep without odors (phase 2). The Pittsburgh Sleep Quality Index and the wearable sleep monitoring device "Fitbit Charge 2" were used to measure sleep quality.

**Results:** After adjusting for baseline differences, participants exposed to "perfume" reported better subjective sleep quality during the odor exposure phase compared to the control group. Among the applications evaluated, the use of a diffuser was associated with better sleep quality and reduced daytime fatigue. No significant group differences were observed in sleep parameters measured by Fitbit.

**Conclusion:** Exposure to odors during the night influences sleep quality. Using a diffuser to deliver the odor appears to be associated with better subjective sleep quality and reduced daytime fatigue. No sustained improvement in sleep quality over time was observed with odor exposure.

### References

1 Arzi A, Sela L, Green A, et al (2010) The Influence of Odorants on Respiratory Patterns in Sleep. Chem Senses 35:31–40. https://doi.org/10.1093/chemse/bjp079

2 Stuck BA, Stieber K, Frey S, et al (2007) Arousal Responses to Olfactory or Trigeminal Stimulation During Sleep. Sleep 30:506–510. https://doi.org/10.1093/sleep/30.4.506

3 Ackerley R, Croy I, Olausson H, Badre G (2020) Investigating the Putative Impact of Odors Purported to Have Beneficial Effects on Sleep: Neural and Perceptual Processes. Chemosens Percept 13:93–105. https://doi.org/10.1007/s12078-019-09269-5
4 Perl O, Arzi A, Sela L, et al (2016) Odors enhance slow-wave activity in non-rapid eye movement sleep. J Neurophysiol 115:2294–2302. https://doi.org/10.1152/jn.01001.2015
5 Ko LW, Su CH, Yang MH, et al (2021) A pilot study on essential oil aroma stimulation for enhancing slow-wave EEG in sleeping brain. Sci Rep 11:. https://doi.org/10.1038/s41598-020-80171-x
6 Okabe S, Fukuda K, Mochizuki-Kawai H, Yamada K (2018) Favorite odor induces negative dream emotion during rapid eye movement sleep. Sleep Med 47:72–76. https://doi.org/10.1016/j.sleep.2018.03.026
7 Schredl M, Atanasova D, Hörmann K, et al (2009) Information processing during sleep: The effect of olfactory stimuli on dream content and dream emotions. J Sleep Res 18:285–290. https://doi.org/10.1111/j.1365-2869.2009.00737.x
8 Alqahtani AS, Aldhahi MI, Alqahtani F, et al (2022) Impact of the loss of smell on the sleep quality and fatigue level in COVID 19 survivors. European Archives of Oto- Rhino-Laryngology 279:4443–4449. https://doi.org/10.1007/s00405-022-07381-z
9 Fismer KL, Pilkington K (2012) Lavender and sleep: A systematic review of the evidence. Eur J Integr Med 4:e436–e447. https://doi.org/10.1016/j.eujim.2012.08.001
10 Her J, Cho MK (2021) Effect of aromatherapy on sleep quality of adults and elderly people: A systematic literature review and meta-analysis. Complement Ther Med 60
11 Tang Y, Gong M, Qin X, et al (2021) The Therapeutic Effect of Aromatherapy on Insomnia: a Meta-Analysis. J Affect Disord 288:1–9. https://doi.org/10.1016/j.jad.2021.03.066
12 Sabiniewicz A, Zimmermann P, Ozturk GA, et al (2022) Effects of odors on sleep quality in 139 healthy participants. Sci Rep 12:1–12. https://doi.org/10.1038/s41598-022-21371-5
13 Mahmut MK, Oelschlägel A, Haehner A, Hummel T (2022) The impact of olfactory training using a nasal clip and extended periods of odor exposure. J Sens Stud 37:e12721. https://doi.org/10.1111/joss.12721
14 Hummel T, Sekinger B, Wolf SR, et al (1997) 'Sniffin'sticks'': olfactory performance assessed by the combined testing of odor identification, odor discrimination and olfactory threshold.' Chem Senses 22:39–52. https://doi.org/10.1017/S0021121400013067
15 Buysse DJ, Reynolds CF, Monk TH, et al (1989) The Pittsburgh sleep quality index: A new instrument for psychiatric practice and research. Psychiatry Res 28:193–213. https://doi.org/10.1016/0165-1781(89)90047-4
16 Montgomery-Downs HE, Insana SP, Bond JA (2012) Movement toward a novel activity monitoring device. Sleep and Breathing 16:913–917. https://doi.org/10.1007/s11325-011-0585-y
17 Hirokawa K, Nishimoto T, Taniguchi T (2012) Effects of Lavender Aroma on Sleep Quality in Healthy Japanese Students. Percept Mot Skills 114:111–122. https://doi.org/10.2466/13.15.PMS.114.1.111-122
18 El Alaoui C, Chemin J, Fechtali T, Lory P (2017) Modulation of T-type Ca2+ channels by Lavender and Rosemary extracts. PLoS One 12: e0186864. https://doi.org/10.1371/journal.pone.0186864
19 Goel N, Kim H, Lao RP (2005) An Olfactory Stimulus Modifies Nighttime Sleep in Young Men and Women. Chronobiol Int 22:889–904. https://doi.org/10.1080/07420520500263276
20 Haze S, Sakai K, Gozu Y (2002) Effects of Fragrance Inhalation on Sympathetic Activity in Normal Adults. Jpn J Pharmacol 90:247–253. https://doi.org/10.1254/jjp.90.247
21 Chien L-W, Cheng SL, Liu CF (2012) The Effect of Lavender Aromatherapy on Autonomic Nervous System in Midlife Women with Insomnia. Evidence-Based Complementary and Alternative Medicine 2012:1–8. https://doi.org/10.1155/2012/740813
22 Lewith GT, Godfrey AD, Prescott P (2005) A Single-Blinded, Randomized Pilot Study Evaluating the Aroma of Lavandula augustifolia as a Treatment for Mild Insomnia. The Journal of Alternative and Complementary Medicine 11:631–637. https://doi.org/10.1089/acm.2005.11.631
23 Howard S, Hughes BM (2008) Expectancies, not aroma, explain impact of lavender aromatherapy on psychophysiological indices of relaxation in young healthy women. Br J Health Psychol 13:603–617. https://doi.org/10.1348/135910707X238734
24 Martinec Nováková L, Miletínová E, Kliková M, Bušková J (2021) Effects of all-night exposure to ambient odour on dreams and affective state upon waking. Physiol Behav 230:113265. https://doi.org/10.1016/j.physbeh.2020.113265
25 Stuck BA, Grupp K, Frey S, et al (2008) Chemosensorisch induzierte Arousals im Schlaf – eine erweiterte Studie. Somnologie - Schlafforschung und Schlafmedizin 12:212–218. https://doi.org/10.1007/s11818-008-0349-5

**abstract citation id: bjaf055.149**

### Perceived naturalness in perfumery: a cognitive construct

*Ulrich Ambassa Menyié*[1]

[1]*Nice Chemistry Institute. France*

In the flavor and fragrance industry, naturality is a key driver of consumer preference, brand positioning, and regulatory strategy. This study explores the perception of olfactory naturalness through a mixed-methods approach combining psychophysics and sensory evaluation. Qualitative data collected from 377 participants allowed for the identification of olfactory families commonly associated with the perception of naturalness. In parallel, quantitative data from subjective evaluations involving over 30 participants from two distinct cultural contexts (French and South Korean) enabled an examination of cultural influences on this perception. This cross-cultural perspective contributes to a deeper understanding of how odors are perceived as "natural," independent of their chemical composition or the presence of visual cues such as labeling. The results will open up promising avenues for future research, particularly into the potential cognitive effects of odors perceived as natural.

**abstract citation id: bjaf055.150**

### Modeling heavy metal-induced taste dysfunction using human iPSC-derived taste bud organoids

*Lisa Marie Reinhardt*[1]*, Andrea Rossi*[1]

[1]*IUF- Leibniz Research Institute for Environmental Medicine. Germany*

Environmental heavy metals such as lead and cadmium are known to impair taste perception and damage the olfactory system; however, the underlying mechanisms remain poorly understood, largely due to the absence of physiologically relevant human models. We hypothesize that exposure to these pollutants disrupts the function and viability of taste receptor cells, contributing to age-related taste loss and altered dietary behavior. To address this, we will employ a two- pronged strategy: a 2D culture of immortalized human fungiform taste cells, and the development of a novel 3D

taste bud organoid model derived from human induced pluripotent stem cells (iPSCs). These complementary systems, coupled with sophisticated exposure setups, will enable us to investigate the cellular and molecular impact of defined concentrations of cadmium and lead under conditions that mimic real-world exposure. We will analyze chemosensory function and cellular integrity by functional assays-including calcium imaging, gene expression profiling (qPCR, RNA-seq), and immunostaining for the key taste receptors TAS1R (sweet/umami), TAS2R (bitter), and mechanoreceptors (PIEZO1/2). Fundamental results demonstrate that heavy metal exposure significantly reduces taste receptor expression and impairs calcium signaling in taste bud organoids, indicating compromised taste transduction[1,2]. Additional analyses will address the impact on mechanoreceptor function and taste cell subtype differentiation. By integrating these molecular and cellular data, the project aims to clarify the mechanisms through which pollutants impair taste perception, identify the exposure levels at which this occurs, and to establish 2D cells and 3D human organoid models as a relevant platform for chemosensory toxicology research. This work will provide a foundation for understanding pollutant-induced taste dysfunction and inform strategies to mitigate sensory health risks in exposed populations.

### References

1 Genter, M. B., & Doty, R. L. (2019). Toxic exposures and the senses of taste and smell. Handbook of Clinical Neurology, 164, 389–408. https://doi.org/10.1016/B978-0-444-63855-7.00022-8

2 Zheng, Y., Shen, Y., Zhu, Z., & Hu, H. (2020). Associations between Cadmium Exposure and Taste and Smell Dysfunction: Results from the National Health and Nutrition Examination Survey (NHANES), 2011–2014. International Journal of Environmental Research and Public Health, 17(3), 943. https://doi.org/10.3390/IJERPH17030943

**abstract citation id: bjaf055.151**

### Olfactory function and mental health

*Barbara Żyżelewicz[1], Anna Oleszkiewicz[1]*

*[1]Institute of Psychology, University of Wroclaw, Wroclaw. Poland*

**Aim of study:** The risk of developing depressive symptoms increases with age. The following study aimed to verify if olfactory training, a regular, structured exposure to odors, may relieve symptoms of depression in the older people population.

**Methods:** Older people participants ($N$ =101, $M_{age}$=72.6, $SD$=5.44, 88% women) participated in this study. The experimental group ($N$ = 50, $M_{age}$ = 72.0, $SD$ =5.49) underwent 12-week olfactory training sniffing twice a day four scented felt-tip pens (so-called the Sniffin' Sticks) containing scent of cloves, scent of rose, scent of eucalyptus, and scent of lemon. Placebo group (N=51, $M_{age}$= 73.2, SD=5.37) was performing the same training regimen with odorless Sniffin' Sticks. Depressive symptoms were measured before and after the training with the Patient Health Questionnaire (PHQ-9), compliant with diagnostic criteria according to the DSM-V.

**Results:** The analysis did not show a statistically significant effect of olfactory training on depressive symptoms in the overall sample of older individuals. However, we found promising preliminary evidence for the effectiveness of olfactory training to relieve depressive symptoms in the subsample (N = 20) of older individuals with baseline moderate depression.

**Conclusions:** The present investigation offers preliminary evidence that olfactory training is an enjoyable and easy-to-administer intervention in the geriatric population, which further predestines it for psychological effectiveness testing in the older populations, especially clinical groups. These results should be replicated in an extended, targeted geriatric sample with moderate depression.

**Funding:** This work was supported by the National Science Centre grant (OPUS scheme 2020/37/B/HS6/00288) awarded to Anna Oleszkiewicz.

**abstract citation id: bjaf055.152**

### Flavor-flavor and flavor-nutrient conditioning with odors: competition or amplification?

*Lucile Rey[1], Maria Sifakaki[1], Amy Xu[1], Janina Seubert[1]*

*[1]Department of Clinical Neuroscience, Psychology Division, Karolinska Institutet. Sweden*

Imagine you are hungry, and the sweet scent of vanilla draws you in to buy a cake. Two forms of conditioning can explain this behavior: flavor-flavor (FFc) where sweet taste acts as the unconditioned stimulus, and flavor-nutrient (FNc), where associations with postprandial metabolic effects are formed. Whether these two processes amplify, inhibit, or remain independent of each other remains unclear. To answer this question, fasted participants completed two sessions during which FFc of a target odor with sweet taste was implemented and disguised via triangle tests across four blocks of five trials each. Each trial consisted of participants tasting three solutions and indicating whether a cup contained a different flavor or all were the same. Of the five trials (randomized), two were conditioning trials, with three cups of 1. an unfamiliar odor mixed with sucralose (CS+), and 2. another unfamiliar odor mixed with water (CS-). The remaining trials served as distractors and included one odd stimulus among two others (coconut/almond odor, saline, or water). Each block was either preceded by a maltodextrin-based caloric beverage or water (control), depending on the session (FNc). Associative learning and metabolic responses were assessed via odor ratings (wanting, sweetness, liking) and blood glucose measures collected before and after the full conditioning session.

Preliminary results show that both CS+ and CS– odors were rated sweeter and elicited higher wanting during the maltodextrin compared to the water session. However, in the water session only, CS+ ratings increased more than CS– from pre- to post-conditioning, both in sweetness and wanting. These results suggest that FFc and FNc rely on distinct mechanisms, with FNc attenuating or overriding the effects of FFc. This could reflect the involvement of distinct neural processes and highlight wanting dimension in assessing odor memories.

**Funding:** This study was supported by the ERC under the H2020 program (Grant No. 947886).

**abstract citation id: bjaf055.153**

### Human olfactory sensitivity varies across geographical locations

*Aleksandra Reichert[1], Anna Oleszkiewicz[1,2], Thomas Hummel[2]*

*[1]University of Wrocław. Poland*
*[2]Technische Universität Dresden. Germany*

It has been assumed that olfactory sensitivity is relatively consistent in different populations worldwide. Emerging, yet fragmented, evidence lends credit to the hypothesis that olfactory sensitivity may be ethnically and geographically diverse. To gain deeper insight regarding the interplay between environmental, demographic, and health factors in the context of olfactory sensitivity, we conducted a multicenter study comprising data from 1046 from 19 locations around the world. Our results revealed that location accounted for 17-20% of the variance in chemosensory sensitivity. Demographic and psychological factors related to working memory and depressive symptoms are additionally helpful in explaining sensitivity to odors, accounting for 1.8-2.7% of variance in chemosensory sensitivity. People inhabiting different regions may have different sensitivities to chemical stimuli due to varying exposures to atmospheric conditions and different chemosensory experiences in daily life. We discuss the potential source of ~80% of unexplained variance.

**Funding:** This work was supported by National Science Centre in Poland (#2020/39/B/HS6/01533 awarded to Anna Oleszkiewicz).

**abstract citation id: bjaf055.154**

### Olfactory cues evoke more autobiographical memories than visual and auditory stimuli

*Yağmur Üngüder[1], Berçem Yar[1], Maria Veldhuizen[1]*

*[1]Mersin University. Turkey*

Odor-evoked autobiographical memories are often described as pleasant, vivid and emotionally rich. However, empirical evidence regarding their distinctiveness compared to other sensory cues remains mixed. This study examined the characteristics of autobiographical memories triggered by naturalistic odors, photographs, and sounds, focusing on their emotionality, vividness, and pleasantness. The final sample in this between-participants design consisted of 106 participants from Türkiye (51 women, 25-45 years, M=31.90, SD=7.49). In each of the three conditions, participants were presented with cues corresponding to 10 objects in a random order (odors: the actual scented objects, photographs: color images, words: the names of the objects displayed on a computer screen). Following each cue, participants were instructed to recall a personal memory associated with the presented cue. If a memory was successfully retrieved, the subject was asked to write their memory as detailed as possible, and rated the evoked memory based on its experiential characteristics (emotionality, pleasantness, vividness of the memory). Results showed no effect of cue condition on emotionality, pleasantness, or vividness of the recalled memories ($p>.05$). However, a significant main effect of cue type was found for the total number of memories retrieved ($F(2,98) = 3.383$, $p = .038$, $\eta^2p = .065$), with odors eliciting more memories overall compared to the other modalities, although post hoc t-tests did not reach significance after correction for multiple comparisons. The results suggest that odors may serve as more effective cues for retrieving autobiographical memories in terms of quantity, though not in emotional or sensory quality. Previous studies rarely found an advantage for odor cues, and we speculate that our use of complex naturalistic odors may have contributed, which future studies may test directly.

**abstract citation id: bjaf055.155**

### A low-cost olfactometer for simultaneous odour delivery in multi- animal rodent behavioural studies

*Connor Doyle[1], Elisa Galliano[1], Chloe Guillaume[1]*

*[1]University of Cambridge. United Kingdom*

Olfactometers are essential tools in chemosensory research, enabling precise temporal and spatial control of odour delivery to study odour-driven behaviour in animals and humans. While sophisticated designs are available, replicating custom setups can be challenging for laboratories with modest budgets and without access to in-house workshops. Commercial systems, though reliable and convenient, often involve high costs, proprietary components, and limited flexibility for customisation or integration with behavioural and recording setups. To address these accessibility barriers, we developed a versatile, cost-effective 4-module (expandable) olfactometer using only commercially available parts and open-source software. Each module has two channels (diluted odorant and solvent alone), with outputs combined and delivered in parallel to four behavioural test chambers – each receiving the same stimulus simultaneously. To ensure equal air flow, only one channel per module is active: for single-odour presentations, one odour bottle and three solvent bottles are opened; for mixtures, selected modules deliver odour while the others deliver solvent. An Arduino microcontroller operates solenoid valves for precise, programmable stimulus delivery. Performance was validated using miniPID recordings across all modules and chambers, confirming consistent temporally controlled odour delivery across all modules at various valve opening times and odour concentrations. Behavioural validation of the olfactometer with mice was achieved using innately attractive or neutral odours. Mouse behaviour was recorded during exposure with inexpensive USB cameras and analysed using open- source tools such as Bonsai and DeepLabCut. Analyses revealed mice show clear preference for opposite sex urine over solvent controls and spent more time investigation odour ports during active odour delivery, validating both the technical performance and biological relevance of the system.

**abstract citation id: bjaf055.156**

### Spatial distribution and characterization of olfactory and respiratory epithelium in aging human nasal tissue

*Robin Indro Scherer*[1], *Moritz Klingenstein*[1], *Stefanie Klingenstein*[1], *Stefan Liebau*[1]

[1]*Institute of Neuroanatomy & Developmental Biology (INDB), Eberhard Karls University Tübingen. Germany*

It is well established that the olfactory epithelium (OE) regresses throughout human life and undergoes metaplasia into respiratory epithelium (RE). To further investigate this process, several human nasal cavities were decalcified using EDTA solution, cryosectioned, and analyzed via immunohistochemistry. A multiplexing approach was established to maximize information from each section, enabling multiple rounds of antibody staining on the same cryosection. Using this method, both OE and RE were characterized in detail.

Based on the cryosection data, a 3D model was reconstructed to visualize the nasal cavity and the spacial distribution of the two epithelia. The analysis revealed extensive areas lacking clear markers for either OE or RE such as OMP, CALB2, CD56 or AcTUB. These regions may represent transitional epithelium that is gradually replaced by RE over time, or permanent aneuronal zones of the OE. In addition, the surface area occupied by OE showed a large interindividual variability.

These findings contribute to a better understanding of epithelial remodeling in the aging human olfactory system and may serve as a foundation and inspiration for further investigations into the structure of the olfactory epithelium and the cause of its metaplastic transformation into respiratory epithelium.

**Funding:** All funding was granted by the University of Tübingen.

**abstract citation id: bjaf055.157**

### Analysis of chemosensory function in mice using a novel contact- based stimulus delivery device

*Yifat Elizera*[1], *Itamar Frachtenberg*[1], *Gerard Elberg*[1], *Abdullah Khatib*[1], *Yoram Ben Shaul*[1]

[1]*Hebrew University of Jerusalem. Israel*

Like many other organisms, mice rely on their olfactory system to obtain critical information about their animate and non- animate environment. The vomeronasal system (VNS) plays a key role in processing cues from other organisms. One of its defining features is the mode of stimulus uptake, which requires physical contact with the substrate for efficient stimulus sampling. This feature allows the VNS to detect non-volatile molecules, which do not efficiently activate the main olfactory system. Thus, to systematically investigate VNS dependent behavioral abilities, and ongoing neuronal activity during stimulus processing, an effective way to deliver stimuli to behaving animals is needed. To address this need, we recently developed the liquid stimulus delivery system (LSDS), designed to deliver fluid natural secretions to awake head-fixed mice using direct nasal contact. Here, we used this system to test chemosensory discrimination of urine from two conspecific strains. We characterized performance as a function of stimulus dilution, contact vs. no contact delivery mode, and for distinct urinary fractions containing high and low molecular weight components. At this stage, our analyses indicate that mice can successfully discriminate urine samples using all urine fractions, both with and without nasal contact. These results suggest that the mice can utilize diverse cues to discriminate among strains, and that more challenging behavioral tasks may be required to identify sensory abilities that strictly rely on vomeronasal system activation.

**abstract citation id: bjaf055.158**

### Integrating biological and psychophysical knowledge into Machine Learning models for predicting human scent perception

*Luana Queiroz de Pinho*[1], *Robert Pollice*[2], *Bernardo Aguilera-Mercado*[3], *Idelfonso Nogueira Bessa dos Reis*[1]

[1]*Norwegian University of Science and Technology. Norway*
[2]*Stratingh Institute for Chemistry, University of Groningen. The Netherlands*
[3]*Corporate Fragrance R&D, The Procter & Gamble Company. United States*

This project aims to develop biologically informed artificial intelligence models that integrate molecular, psychophysical, and receptor-level data to predict human scent perception. The main objective is to establish robust correlations between a scent's perceptual attributes, namely its character and intensity, its molecular structure, and olfactory receptors activation patterns. The central hypothesis suggests that both qualitative (character) and quantitative (intensity) are mediated by the same olfactory receptors, suggesting a unified biological mechanism underlying human olfactory experience. The methodology involves four main stages. First, large, structured datasets are compiled and harmonized from both public and commercial sources, containing molecular descriptors, perceptual ratings, and experimentally validated receptor interaction data. Next, data curation includes ontology mapping to align the diverse scent vocabularies used across datasets.

Chemometric analysis is applied to extract latent variable structures and improve data conditioning. Two parallel AI modeling approaches are pursued. In the first, graph neural networks are trained on receptor interaction profiles and physicochemical properties encoded in graph form. In the second, transformer-based sequence models are developed using augmented molecular strings such as SMILES and SELFIES. These models are pre-trained unsupervised on large datasets, then fine-tuned with supervised learning to predict perceptual labels, including scent character and intensity. This study highlights the potential of integrating biological and psychophysical insights into AI to model olfaction. Preliminary results support the hypothesis that incorporating available olfactory receptor data and psychophysical information in model pretraining improves predictive accuracy for both character and intensity. These models contribute to fundamental sensory science and practical applications in fragrance design and innovation.

**abstract citation id: bjaf055.159**

### c-Kit signaling confers damage-resistance to sweet taste cells upon nerve injury

*Suyoung Ki*[1], *Yong Taek Jeong*[1]

[1]*Korea University. Republic of Korea Korea.*

Taste buds relay taste sensory information to the primary taste neurons but depend on those same neurons for essential components to maintain function. While denervation-induced taste bud degeneration and subsequent regeneration were discovered decades ago, the mechanisms underlying these phenomena (e.g., heterogenous cellular responses to nerve injury and the signaling pathways involved) remain poorly understood. Here, using mouse genetics, nerve injury models, pharmacologic manipulation, and taste bud organoid models, we identify a specific subpopulation of taste cells, predominantly c-Kit-expressing sweet cells, that exhibit superior resistance to nerve injury. We found the c-Kit inhibitor imatinib selectively reduced the number of residual c-Kit-expressing sweet cells at post-operation week 2, subsequently attenuating the re-emergence of other type II cells by post-operation week 4. In taste bud organoids, c-Kit-expressing cells were resistant to R-spondin withdrawal but susceptible to imatinib, while other taste cell types showed the opposite behavior. We also observed a distinct population of residual taste cells that acquired stem-like properties, generating clonal descendent cells among suprabasal keratinocytes independent of c-Kit signaling. Together, our findings reveal that c-Kit signaling confers resilience on c-Kit-expressing sweet cells and supports the broader reconstruction of taste buds during the later regenerative stage following nerve injury.

**Funding:** This work was supported by National Research Foundation of Korea (NRF) grants funded by the Korean Government (the Ministry of Science and ICT, RS-2023-00208193, and RS-2022-NR071221).

**abstract citation id: bjaf055.160**

### Establishment of taste bud organoids from anterior lingual mucosa

*Min Gyeong Lee*[1], *Yong Taek Jeong*[1]

[1]*Korea University. Republic of Korea*

Excessive salt intake is a major risk factor for various modern diseases. Salt perception is regulated by taste cells within taste buds, particularly those responsive to salty stimuli located in the fungiform papillae (FuP) of the anterior tongue. However, their limited number has made molecular and cellular analysis difficult. While many in vivo studies on taste have been conducted, suitable in vitro models remain scarce. Although organoid technology has been applied to mimic taste buds, all existing taste bud organoids have been derived from circumvallate papillae (CVP) in the posterior tongue. No model has been reported that recapitulates FuP-specific taste cells. Here, we successfully established culture conditions to generate anterior tongue-derived taste bud organoids (aTBOs) containing all major taste cell types. This represents the first in vitro model that mimics FuP taste cell biology. Our platform provides a valuable tool to investigate the mechanisms of salty taste perception and may contribute to developing strategies for regulating salt intake.

**abstract citation id: bjaf055.161**

### Whole-brain encoding of opposite-sex odors in mice

*Flavia Maria Domizia Di Fiore*[1], *Alessandra Stella*[2], *Stefano Zucca*[1], *Sarah M. Zala*[2], *Silvia de Marchis*[1], *Dustin J. Penn*[2], *Paolo M. Peretto*[1], *Serena Bovetti*[1]

[1]*Lab. of Adult Neurogenesis, Dept. of Life Sciences and System Biology, University of Turin, Turin, Italy and Neuroscience Institute Cavalieri Ottolenghi, University of Torino, Orbassano, Italy*
[2]*Konrad Lorenz Institute of Ethology, University of Veterinary Medicine, Vienna, Austria. Austria*

Sexual imprinting is a form of learned mate preference in which individuals develop attraction (positive) or avoidance (negative) to traits observed during their early social environment[1]. These preferences, shape adult mate choice and promote pairing with partners likely to provide high-quality parental care [2]. In rodents, olfaction is the major sensory modality and plays an important role in regulating social behaviors, particularly in mate selection[3]. Here, we performed whole-brain analyses of females reared with and without their fathers and then exposed to familiar (father-exposed) and unfamiliar (novel) olfactory stimuli. We also measured females' behavior during stimulus exposure, to correlate brain activation with behavioral performance. Additionally, for generalisation and ecological validation, we compared whole-brain activation and behavior in C57BL/6j laboratory females with wild house mice. Neuronal activity was measured using whole- brain immunolabeling for the immediate early gene c-Fos. Brains were cleared using the iDISCO protocol and imaged by light-sheet fluorescence microscopy. We used ClearMap for single-cell detection and brain atlas registration. Our results show clear differences between laboratory and wild mice in both recruited brain areas and behavior. Laboratory mice actively interact with stimuli, whereas wild mice have less interaction with stimuli, with increased contact to familiar compared to novel odor cues. Differences in behavior correlate with differences in brain activation. Although we see an overall overlap in macro-regions between experimental groups, wild mice show less activation compared to laboratory mice, and specific recruitment of different subregions. Brain activation correlates well with differences in behavioral performance between laboratory and wild mice highlighting different specific strategies for odor-driven social decision-making and providing insight into the neural basis of learned mate preferences.

### References

1 Immelmann, K. (1975). Ecological significance of imprinting and early learning. Annual Review of Ecology and Systematics, 6, 15–37.

2 Little, A. C., Jones, B. C., & DeBruine, L. M. (2011). Facial attractiveness: Evolutionary based research. Philosophical Transactions of the Royal Society B: Biological Sciences, 366, 1638–1659. doi:10.1098/rstb.2010.0404

3 Rymer TL. The Role of Olfactory Genes in the Expression of Rodent Paternal Care Behavior. Genes (Basel). 2020 Mar 10;11(3):292. doi:10.3390/genes11030292

**abstract citation id: bjaf055.162**

### Olfactory training using nasal inserts is more effective due to increased adherence

*Anja Winter[1], Sofie Henecke[1], Evelina Thunell[1], Mattias Swartz[1], Joakim Martinsen[1], Pernilla Sahlstrand Johnson[2], Johan Lundstrom[1]*

*[1]Karolinska Institutet. Sweden*
*[2]Lund University. Sweden*

The recommended treatment for hyposmia (a clinically reduced sense of smell) is olfactory training using odor containers that the patients smell twice a day for several weeks. Adherence to the olfactory training regimen is, however, generally low. We aimed to investigate if a new form of odor delivery, using scented nasal inserts, could enhance adherence to olfactory training by allowing participants to be mobile during the training and thereby lower the perceived intrusion on everyday life. Using a randomized controlled parallel-group design, individuals (N = 116) with hyposmia underwent 8 weeks of olfactory training. One group was assigned olfactory training using scented nasal inserts (nasal devices that retain nasal patency) while the other group was assigned the standard care regimen currently recommended by the Swedish healthcare system. We assessed objective and subjective olfactory ability before and after olfactory training as well as adherence to training.

Both groups significantly improved both their objective and subjective olfactory abilities, and training with nasal inserts produced similar improvement as standard care in overall treatment outcome. However, there was a significantly greater increase in discrimination performance and lower dropout rate (6.7%) in the nasal insert compared to the standard care group (23.2%). Critically, after exclusion of the drop-out participants, the nasal insert group still showed significantly higher adherence to the training regimen. Olfactory training with nasal inserts could serve as a more effective form of treatment for hyposmia due to patients' improved adherence to protocol and increased tendency to finish their treatment regimen.

**abstract citation id: bjaf055.163**

### Receptor responses to pure odorants in Drosophila melanogaster olfactory receptors

*Giovanni Galizia[1], Alja Lüdke[1], Ajayrama Kumaraswamy[1]*

*[1]University of Konstanz. Germany*

Olfactory coding relies on primary information from olfactory receptor cells, that respond to volatile airborne substances. Despite of extensive efforts, our understanding of odor-response profiles across receptors is still poor, due to the vast number of possible ligands (odorants), the high sensitivity even to trace compounds (creating false positive responses), and the diversity of olfactory receptors. Here, we linked chemical purification with a gas chromatograph to single-receptor type recording with transgenic flies using calcium imaging to record olfactory responses to a large panel of chemicals in 7 Drosophila ORs: Or10a, Or13a, Or22a, Or42b, Or47a, Or56a, Or92a. We analyze the data using linear-non-linear modeling, and reveal that most receptors have "simple" response types (mostly positive: Or10a, Or13a, Or22a, Or47a, Or56a).

However, two receptors (Or42b, Or92a) have, in addition to "simple" responses, "complex" response types to some ligands, either positive with a negative second phase, or negative with a positive second phase, suggesting the presence of multiple binding sites and/or transduction cascades. We show that some ligands reported in the literature are false positives, due to contaminations in the stimulus. We recorded all stimuli across concentrations, showing that at different concentrations different substances appear as best ligands. Our data show that studying combinatorial olfactory coding must consider temporal response properties and odorant concentration, and in addition is strongly influenced by the presence of trace amounts of ligands (contaminations) in the samples. These observations have important repercussions for our thinking about how animals navigate their olfactory environment.

**abstract citation id: bjaf055.164**

### Safeguarding olfactory and flavor heritage amid climate change: the SCENTINEL Project in Brazil

*Idelfonso Nogueira Bessa dos Reis*[1], *Luana Queiroz de Pinho*[1], *Jana Striova*[2], *Barbara Zitová*[3], *Cecilia Bembibre*[4], *Matija Strlič*[5]

[1]*Norwegian University of Science and Technology. Norway*
[2]*National Research Council - National Institute of Optics. Italy*
[3]*The Czech Academy of Sciences, Institute of Information Theory and Automation. Czech Republic*
[4]*Institute for Sustainable Heritage, University College London. United Kingdom*
[5]*Heritage Science Laboratory Ljubljana, Faculty of Chemistry and Chemical Technology, University of Ljubljana. Slovenia*

The SCENTINEL Project aims to preserve and digitize scents and flavors of living cultural and historical significance that are under threat due to climate change. Recognizing the vital role of chemosensory experiences in shaping identity and heritage, the project seeks to identify, evaluate, protect, and reproduce olfactory and taste elements integral to human cultural practices. To identify intangible sensorial heritage at risk, the project combined extensive literature reviews with in-depth interviews conducted with community leaders and cultural practitioners. These qualitative methods informed the selection of four Brazilian cultural expressions as case studies: Lavagem do Bonfim (Washing of the Bonfim Church), Baianas do Acarajé (Street Food of Bahia), the Festival of Yemanjá, and elements of Yorubá culture. Fieldwork included the capture of volatile organic compounds (VOCs) using headspace sampling techniques, including Tenax tubes and solid-phase microextraction (SPME) fibers.

Preliminary VOC data were successfully collected from each cultural setting. Samples are currently undergoing laboratory analysis to identify key scent compounds. These results will inform the formulation of synthetic reproductions and enable digital encoding of the olfactory and flavor profiles. Machine learning techniques are being developed to assist in both scent and flavors classification and digital preservation. The SCENTINEL Project demonstrates a multidisciplinary and community- based approach to safeguarding sensorial heritage. It highlights how olfactory, and flavors elements can be documented and preserved through scientific and digital methods. Moreover, the project underscores the impact of chemoreception on cultural identity and community cohesion, offering a novel perspective within sensory research.

**abstract citation id: bjaf055.165**

### Building a shared digital infrastructure for olfactory research: the Hub4Smell initiative

*Valentina Parma*[1], *Patricia Lucas Schnarre*[2], *Richard Kedziora*[3], *Danielle Reed*[1], *Pamela Dalton*[1]

[1]*Monell Chemical Senses Center. United States*
[2]*Ahersla Health. United States*
[3]*Estenda Solutions Inc. United States*

Hub4Smell is a novel digital infrastructure developed to advance olfactory science by providing standardized, scalable tools for human smell data collection, integration, and analysis. Although olfaction is increasingly recognized as critical to health and behavior, the widespread implementation of olfactory assessment and data-driven research remains constrained by the absence of validated, accessible methodologies and interoperable data systems.

Hub4Smell addresses these limitations through a customizable suite of tools for data collection, curation, and analysis, built on principles of open science and incorporating recent advances in conversational analytics. A secure online environment fosters methodological exchange, expert consultation, and multi-site study coordination. The platform was developed through collaboration among academic researchers, digital health developers, and medical device experts, and aligns with national public health priorities outlined in the NIDCD 2023–2027 Strategic Plan and Healthy People 2030.

To assess its utility, we analyzed a dataset from N = 400 adults, with and without olfactory dysfunction, who completed four validated olfactory tests (Sniffin' Sticks Identification, NIH Toolbox Odor Identification, BSIT, and SCENTinel® test) and visual analog scales assessing smell-related attributes (e.g., perceived odor intensity). Results demonstrate Hub4Smell's capacity to integrate heterogeneous datasets across test types and participant characteristics.

These findings illustrate the platform's potential to support reproducible olfactory phenotyping and enable comparative analyses across diverse populations and measures, thereby facilitating scalable, translational chemosensory research.

**Funding:** NIDCD (grant #R44DC021198).

**abstract citation id: bjaf055.166**

### Temporal discrimination by sensory and projection neurons of the accessory and main olfactory bulbs

*Judit Levy*[1], *Karen Marom*[1], *Shiran Bercu*[1], *Yoram Ben-Shaul*[1]

[1]*Hebrew University of Jerusalem. Israel*

Olfaction relies on temporal patterns of neural activity to encode information about odor stimuli. In mammals, the main olfactory bulb (MOB) and accessory olfactory bulb (AOB) are key brain regions involved in early olfactory processing. While the ability of the main olfactory system to encode fine temporal features has been previously demonstrated, the vomeronasal system is traditionally viewed as less involved in such discrimination. Our research investigated the capacity of mice to detect subtle changes in the timing of optogenetic stimulation delivered to the MOB or AOB. We focused on four types of temporal manipulations: pulse frequency (different pulse length and rate), pulse duration (different pulse length, same rate), inter-pulse intervals (different gap between fixed-length pulses), and timing relative to the breathing phase.

Experiments were conducted in behaving, head-fixed transgenic mice using a modified lick/no-lick two-alternative forced choice paradigm. We optogenetically activated mitral/tufted cells or olfactory sensory neurons and compared behavioral responses following MOB and AOB stimulation. Both systems exhibited robust discrimination when mitral/tufted cells were activated, with mice distinguishing pulse duration differences as small as 15 ms and inter-pulse interval differences of 60 ms. In contrast, with sensory neuron activation, mice could still discriminate 15 ms pulse duration differences, but failed to detect even 170 ms inter-pulse interval gaps. Notably, the vomeronasal system showed discrimination abilities comparable to those of the main olfactory system across tasks, indicating that both systems can reliably encode temporal features of sensory input.

**Funding:** This research was supported by ISF grant No. 612/19.

**abstract citation id: bjaf055.167**

### Stimulus-dependent modulation in mouse olfactory signal transduction

*Victoria K. Switacz[1], Daniela R. Drose[1], Marc Spehr[1]*

*[1]Department of Chemosensation, Institute for Biology II, RWTH Aachen University. Germany*

Short-term sensory adaptation to prolonged or repetitive stimuli is essential for maintaining dynamic sensitivity in sensory systems. In olfactory sensory neurons (OSNs), odorant receptor activation triggers a G-protein-mediated cAMP cascade, which is counterbalanced by $Ca^{2+}$/calmodulin-dependent negative feedback. Many olfactory receptors and, thus, OSNs are highly sensitive, allowing detection across odor concentrations that span several orders of magnitude. Beyond adaptation, it remains unclear whether additional dose-dependent modulatory mechanisms influence OSN sensitivity.

Here, we use patch-clamp recordings from OSNs in acute slices to assess how varying interstimulus intervals and stimulus intensities modulate action potential generation, thereby influencing olfactory signal transmission to the brain. We aim to dissect (*i*) which components of the signaling cascade are critical key players in modulation, (*ii*) whether dose-dependent modulation is receptor-dependent, and (*iii*) whether response modulation arises with the maturation of OSNs. Together, we seek to gain insight into how OSNs fine-tune their sensitivity and responsiveness to encode a wide range of odorant concentrations.

**abstract citation id: bjaf055.168**

### Multiple routes to olfactory cortex

*Tabitha L. James[1], Louis Renoult[1], Carl M. Philpott[1], Fraser W. Smith[1]*

*[1]The University of East Anglia. United Kingdom*

Neuroimaging research has shown that both primary and secondary olfactory areas can be activated via top-down imagery and via cross-modal stimuli associated with olfaction. However, it is unclear how similar olfactory representations are when olfactory stimuli are directly presented as compared to when the olfactory network is activated by olfactory imagery or via cross-modal connections. We aimed to explore overlap in olfactory neural representation when olfactory information is received via vision or via olfactory imagery in comparison to bottom-up olfactory input. The study had a final sample of 22 female and 3 male participants ($M_{age}$ = 28.48, ± 6.03 years). In the first fMRI session participants viewed pictures of olfactory associated and non-olfactory associated objects (picture task) followed by an olfactory imagery task. Objects evoking pleasant and unpleasant odours were used. Nineteen participants then completed an olfactory fMRI session where phenethyl alcohol (rose) and hydrogen sulphide (rotten egg) were presented using a computer-controlled olfactometer. MVPA will be used to compare activation patterns across tasks in the following regions: piriform cortex (PC), amygdala (AMY), hippocampus (HPC), insula (INS), and the orbitofrontal cortex (OFC). Preliminary cluster-level corrected whole brain analysis shows activation of key nodes in the olfactory network (PC, AMY, INS, and OFC) in response to olfactory associated pictures when compared to non-olfactory associated pictures. These initial findings suggest that early olfactory regions may process olfactory information from visual sources. Overall, this work will highlight how olfactory information is represented in the brain when received through alternative sensory and cognitive domains as opposed to bottom-up sensory stimulation.

**abstract citation id: bjaf055.169**

### Tropisms of adeno-associated viruses in projection neurons of the mouse olfactory bulb

*Johanna Flesch[1], Moritz M. Nesseler[1], Marc Spehr[1]*

*[1]Department of Chemosensation, Institute for Biology II, RWTH Aachen University, Aachen, Germany*

The main olfactory bulb integrates sensory input from neurons in the main olfactory epithelium and relays information to downstream targets via two projection neuron populations, i.e., mitral and tufted cells. Although these neuronal cell types play a crucial role in olfactory processing, we still lack tools to selectively target either neuron type.

Due to their efficient gene delivery, low cytotoxicity, and versatility, recombinant adeno-associated viruses (rAAVs) have emerged as reliable tools for circuit mapping, functional modulation of neuronal activity and various other aspects of neuroscientific research. However, achieving cell type-specific transduction with rAAVs depends on various factors such as viral volume, titer, serotype and promotor. Here, we sought to identify the rAAV variants most suitable for studying mitral and/or tufted cells of the main olfactory bulb. We systematically evaluated the transduction patterns of different anterograde rAAV virus constructs and serotypes after performing stereotactic MOB injections in transgenic Tbx21-Cre mice, which grant genetic access to olfactory bulb projection neurons.

Our results show that apart from the rAAV serotype, other genomic design factors – especially the promotor sequence – play a crucial role in the cell type specific tropism of the virus.

**abstract citation id: bjaf055.170**

### Astringent compounds target Gαq protein-coupled receptors and the calcium release-activated Orai channels in the human tongue cell line HSC-3

*Alina Ulrike Müller[1], Gaby Andersen[1], Veronika Somoza[1]*

*[1]Leibniz Institute for Food Systems Biology at the Technical University of Munich. Germany*

Astringency is a poorly understood dry and rough sensation in the mouth after consuming polyphenol-rich beverages and is often experienced in conjunction with bitterness. Although there are several theories on how astringency might be perceived, there is still no clear evidence if and which G protein-coupled receptors (GPCRs) are involved.[1,2] We hypothesize that a GPCR coupled to $G\alpha_q$ plays a role in astringency in distinction to bitterness, in which the $G\alpha_i$ type is involved. We also propose that calcium release-activated Orai channels play a role in the lingering perception of astringency by amplifying the transient $Ca^{2+}$ signal of GPCRs through an influx of $Ca^{2+}$. To investigate this, we applied the astringent compound tannic acid (TA), the astringent and bitter compound epigallocatechin gallate (EGCG) and the bitter tasting quinine to the human tongue HSC-3 cell line, as a widely established surrogate model for investigating astringency,[3,4] in $Ca^{2+}$ and cAMP signaling assays. We detected $Ca^{2+}$ signals for TA (EC = 11 μM), EGCG (EC = 330 μM) and quinine (EC = 1.49 mM), but only for TA and EGCG a reduction completely to the baseline ($p < 0.0001$) with the $G\alpha_q$ inhibitor FR900359 and not for quinine. Additionally, a reduction in the signal with the Orai inhibitor Syntha66 for EGCG and TA was detected but not for quinine. Knock down experiments to verify their participation are currently underway. Furthermore, we also showed that EGCG ($IC_{50}$ = 360 μM), TA ($IC_{50}$ = 53 μM) and quinine ($IC_{50}$ = 720 μM) target GPCRs with a $G\alpha_i$ activity by reducing the cAMP levels, which is verified with the $G\alpha_i$ inhibitor pertussis toxin. In summary, these results indicate that astringency might be perceived via $G\alpha_q$ protein- coupled receptors and the lingering effect results from the participation of Orais. We also showed that TA, EGCG and quinine stimulate bitter signaling pathways, a result that aligns with sensory studies, as astringent compounds are commonly perceived as bitter as well.

### References

1 Huang, R. and C. Xu, An overview of the perception and mitigation of astringency associated with phenolic compounds. Compr Rev Food Sci Food Saf, 2021. 20(1): p. 1036-1074
2 Schöbel, N., et al., Astringency is a trigeminal sensation that involves the activation of G protein-coupled signaling by phenolic compounds. Chem Senses, 2014. 39(6): p. 471-87.
3 Soares, S., et al., Oral interactions between a green tea flavanol extract and red wine anthocyanin extract using a new cell-based model: insights on the effect of different oral epithelia. Scientific Reports, 2020. 10(1): p. 12638.
4 Soares, S., et al., Development of a New Cell-Based Oral Model To Study the Interaction of Oral Constituents with Food Polyphenols. Journal of Agricultural and Food Chemistry, 2019. 67(46): p. 12833-12843.

**abstract citation id: bjaf055.171**

### Modulating the modulators: chemogenetic dissection of bulbar dopaminergic contributions to olfactory behaviour

*Chloé Guillaume[1], Ailie McWhinnie[1], Sonu Peedikayil Kurien[1], Connor Doyle[1], Edina Horvath-Gulacsi[1], Elisa Galliano[1]*

*[1]Department of Physiology, Development and Neuroscience, University of Cambridge. United Kingdom*

Dopaminergic neurons have been identified as a distinct class of interneurons within the olfactory bulb (OB), with the majority having the capacity for regeneration throughout life. Although dopamine is known to influence smell-related behaviours, the specific functional contributions of OB dopaminergic neurons to processes such as odour detection and discrimination remain poorly understood. Existing hypotheses about their behavioural roles often lack anatomical specificity, with results potentially confounded by dopaminergic activity from the midbrain and further complicated by the diversity of behavioural assays used, leading to inconsistent findings.

In this study conducted on mice, we used immunohistochemistry to confirm that the number of tyrosine hydroxylase (TH)- expressing neurons in the OB remains stable across life stage. We also quantified their proportion relative to all neurons in the glomerular layer, labelled with NeuroTrace which, unlike DAPI, selectively stains neuronal nuclei. To assess the causal role of this stable but non-dominant DA population, we stereotaxically injected pAAV-hSyn-DIO-hM4D(Gi)-mCherry into the OB of adult dopamine transporter (DAT)-Cre mice, thus targeting exclusively bulbar DA neurons. One month later to allow DREADD expression, we administered clozapine-N-oxide (CNO) intraperitoneally to transiently inhibit these infected DA neurons and evaluated performance across olfactory tasks. These included odour habituation, discrimination, and responses to aversive and appetitive stimuli. Using a custom behavioural setup and associated olfactometer, we measured time spent near the odour port and overall locomotion. Preliminary data suggest that modulating the activity of dopaminergic neurons increases behavioural sensitivity to certain odours.

This work lays the groundwork for unravelling the functional significance of the dopaminergic interneuron population wihtin the OB and their specific contributions to odour-guided behaviour.

**Funding:** BBSRC Grant

**abstract citation id: bjaf055.172**

### Olfactory-evoked reliving of autobiographical memories: music as a comparative modality

*Arisa Kobayashi[1], Saho Ayabe-Kanamura[2]*

[1]*The Master's Program in Neuroscience at the Graduate School of Comprehensive Human Sciences, University of Tsukuba. Japan*
[2]*Faculty of Human Science, University of Tsukuba. Japan*

**Objectives of the study:** This study investigates the sense of reliving the past evoked by olfactory cues, which are closely linked to emotion and autobiographical memory. Music was used for comparison. The focus is on immersive recall as a component of reliving and how the two modalities differ in evoking it.

**Experimental methods used:** Fifty-one university students (mean age = 22.14) were randomly assigned to an odor group (n = 26) or a music group (n = 25). After completing questionnaires on sensory traits, participants were exposed to 15 odor or music stimuli and rated how strongly each evoked autobiographical memories. The stimulus that received the highest rating was chosen for a 3-minute oral memory report. They then rated the memory on reliving, vividness, emotional intensity, pleasantness, and the age at the event. Five items from the Game Experience Questionnaire (GEQ) were also used to assess immersive recall.

**Essential results:** Odors evoked a sense of being brought back to the past (M = 3.81, SD = 1.13), comparable to music (M = 3.96, SD = 0.86), likely because the most evocative stimulus was selected. Odor-evoked memories also showed high GEQ scores (M = 12.04, SD = 3.50), though not significantly different from music (M = 13.33, SD = 4.27). For the item "Time seems to kind of stand still or stop", music scored slightly higher ($p$ = .078). Fewer events were recalled in the odor group (M = 1.32, SD = 0.63) than in the music group (M = 1.92, SD = 0.97; $p$ = .015). However, the amount of reported content did not differ, suggesting odors may evoke more detailed descriptions per event.

**Conclusions:** Olfactory cues can evoke immersive memory experiences comparable to those triggered by music. Moreover, odor-evoked memories tended to contain richer details, underscoring the distinctive potential of olfaction as a cue for autobiographical recall.

**Funding:** This study received no external funding.

### Reference

1 Johan Willander, Sverker Sikström, and Kristina Karlsson, "Multimodal Retrieval of Autobiographical Memories: Sensory Information Contributes Differently to the Recollection of Events," Frontiers in Psychology 6 (2015), https://doi.org/10.3389/fpsyg.2015.01681.

**abstract citation id: bjaf055.173**

### A cake surprise to further investigate the recently discovered visual food stream

*Sümeyra Nur Doğan[1], Gizem Özen[2], Burcu A. Urgen[1], Maria Geraldine Veldhuizen[3]*

[1]*Bilkent University. Turkey*
[2]*Middle East Technical University. Turkey*
[3]*Mersin University. Turkey*

Perception forms a crucial source of information for the control of behavior. The human brain has a category-selective organization to recognize and discriminate between different kinds of especially salient types of visual information, like faces, scenes, words and bodies. A specialization area for food, a primary reinforcer and salient category for behavior, has been suspected (van der Laan et al, 2011), but was only recently confirmed by data-driven methods in three studies of neural responses to a large variety of natural scenes in photos (Jain et al., 2023a; Khosla et al., 2022; Pennock et al., 2023). Food object selective responses were observed in the ventral visual stream in the fusiform cortex. Illusions are an important instrument in the behavioral neuroscientist's toolkit (Eagleman, 2001). Illusions that use perceptual switches between categories allow for the dissociation of category selective responses (Tong et al., 1998). Here we used a dynamic food-object illusion to characterize perceptual and neural responses to foods in the ventral visual stream, with hyper realistic cakes. In "hyper realistic cake videos" artists convincingly disguise a cake as a non-edible object, which is revealed after slicing into the object. In a behavioral study we showed that participants (n = 18) find the hyper-realistic cakes surprising ($F(1,14.64)$ = 23.786), $p$ < .001) and more edible than control videos ($F(1,17.01)$ = 39.457, $p$ <.001). Data collection in the fMRI study is ongoing (n = 15). Preliminary analyses comparing the hyperrealistic cake after the knife cut (relative to before and relative to control videos) indicate that the cake videos evoke a response in the fusiform gyrus (left, 219 voxels, z = 4.45, $p_{FWE}$ = .003, right, 347 voxels,

$z = 4.24$, $p_{FWE} < .001$). Our results suggest that the illusion is successful in providing a perceptual switch and response in the visual food stream.
**Funding:** Funded by TÜBİTAK 223K638

abstract citation id: bjaf055.174

### Altered maturation of doublecortin neurons in the olfactory cortex, olfactory tubercle and paralaminar amygdala in Fmr1-KO and Mecp2-KO mice

*Rafael Esteve Pérez[1], Elisa Corti[2], Carlos Duarte[2], Mónica Santos[2], Enrique Lanuza[1], Jose V. Torres Pérez[3], Vicente Herranz Pérez[1], Carmen Agustín Pavón[1]*

*[1]Universitat de València. Spain*
*[2]CNC-UC - Center for Neuroscience and Cell Biology, University of Coimbra. Portugal*
*[3]Departament de Biologia Cel·lular i Biologia Funcional, Universitat de València, Spain*

Rett syndrome (RTT) and Fragile X syndrome (FXS) are rare neurodevelopmental disorders, affecting mainly females and males, respectively. Patients affected by these syndromes exhibit intellectual disability, motor impairment, autistic features and altered sensory processing, including olfactory deficits. These disorders are caused by loss of function mutations in the X-linked genes MECP2 and FMR1, which are involved in neural maturation. Immature neurons located in the olfactory cortex and paralaminar nucleus of the amygdala are generated during embryonic development and undergo slow postnatal maturation, with sharp decrease during the pubescent period. We hypothesised that deficiency in either Mecp2 or Fmr1 would impair the postnatal maturation trajectories of these neurons. To test this, we performed immunofluorescent detection of doublecortin, a marker of immaturity, in coronal brain sections from infantile, adolescent, and young adult Mecp2-KO, Fmr1-KO and WT mice. In addition, we combined this marker with NeuN, a marker of neuronal maturity, and Ki67, an intrinsic proliferation marker. Our data show that the density of doublecortin neurons and their co-expression with NeuN vary depending on genotype, age and sex, with an excess of doublecortin neurons at specific timepoints in both types of mutants. In addition, we analysed doublecortin-expressing neurons in the olfactory tubercle, where we also observed increased density of immature neurons. Altogether, our results support a key role of Fmr1 and Mecp2 in the protracted maturation of doublecortin-expressing neurons in olfactory regions and suggest that their deficit could contribute to olfactory dysfunction in patients.
**Funding:** Funded by the Conselleria de Educación, Universidades y Empleo from the Generalitat Valenciana (CIAICO/2023/027, CIPROM/2023/053) and FinRett 2022.

abstract citation id: bjaf055.175

### Calretinin-positive interneurons are abundant in the chemosensory amygdala and might be altered in Mecp2- KO mice

*Daniela Jiménez Díaz[1], Rafael Esteve Pérez[1], Enrique Lanuza[1], Carmen Agustín Pavón[1]*

*[1]University of Valencia. Spain*

The amygdala is involved in a wide range of behavioural functions essential for survival, including social interactions and olfactory processing, thanks to their direct inputs from the main and accessory olfactory bulbs (OB). In the OB, there is an abundant population of calretinin-positive (CR+) interneurons, and the olfactory pathway is enriched in CR. However, the distribution of CR+ neurons in the chemosensory nuclei of the amygdala, i.e. cortical and medial, remains poorly studied. In this work, we investigated the distribution of CR-immunoreactivity in the amygdala of mice. To do so, we performed an immunohistochemical staining for CR in WT male mice and Mecp2-KO, model of Rett syndrome, a rare neurodevelopmental disorder causing autistic features and olfactory dysfunction. We found that CR is expressed throughout the amygdala, with different density of both fibres and somata among nuclei. In particular, the density of CR+ somata is higher in the medial and cortical amygdala as compared to the lateral and basolateral nuclei, and scarce in the central amygdala. In addition, we observed two groups of CR+ interneurons in caudal levels with high expression of CR near to the amygdalopiriform transition area, posterolateral cortical amygdaloid nucleus and posteromedial cortical amygdaloid nucleus. On the other hand, one study showed a higher density of CR+ neocortical interneurons in Mecp2-KO, so we focused on further investigating the density of these interneurons in the posteromedial cortical amygdala to see if the results are consistent and the possible effects in the Rett syndrome model. Future research should investigate the implication of CR interneurons in the chemosensory amygdala in the atypical social and olfactory behaviours displayed by Mecp2-KO mice.
**Funding:** Funded by the Conselleria de Educación, Universidades y Empleo from the Generalitat Valenciana (CIAICO/2023/027, CIAICO/2023/041) and FinRett 2022.

abstract citation id: bjaf055.176

### Effects of olfactory stimuli on the recognition of facial expressions in individuals with depressive symptoms: an event-related potential EEG study

*Letizia Zurlo[1], Elisa Dal Bò[2], Claudio Gentili[1], Cinzia Cecchetto[1]*

[1]Department of General Psychology, University of Padova. Italy
[2]University of Padova. Italy

In everyday settings, perception is inherently multisensory, with congruent cues from different sensory modalities often working together to enhance object recognition. Among these senses, olfaction holds a unique connection to emotion and has been shown to influence the perception of emotional stimuli in other modalities. For instance, odors can bias the interpretation of emotional facial expressions, typically shifting perceived valence in line with the odor's affective tone.

Altered olfactory abilities have been linked to many mental health conditions, including depression, though findings on how individuals with depressive symptoms perceive pleasant and unpleasant odors are mixed. Notably, it also remains unclear how people with depressive symptoms integrate emotional information across modalities, such as odors and facial expressions. While individuals with major depression struggle in recognizing happy, disgusted, and neutral faces, the role of olfactory input in shaping this process has not yet been explored. In this pre-registered study, during EEG recording, participants are shown facial expressions of fear or happiness, morphed with neutral expressions along a linear continuum, to create subtle emotional cues. Each image is paired with a pleasant (lavender) or unpleasant (isovaleric acid) odor.

Participants then categorize the emotion of the face and rate its intensity. The study is underway, with a planned sample of 108 participants divided into depressive symptoms and healthy control groups. Behavioral (emotion categorization accuracy; reaction times) and event-related potential measures of early (N1/P1; N170) and late (P3; LPP) processing will be analysed. Understanding how olfactory stimuli affect the recognition of emotional facial expressions in people with depressive symptoms, along with the related neural mechanisms of multisensory integration, will deepen our knowledge of the olfactory context's role in shaping emotional processing in depression.

**abstract citation id: bjaf055.177**

### Sensory Evaluation of Effervescent Nutritional Supplements: Identification and Characterisation of Off-Tastes

*Thomas Delompré[1], Christophe Martin[2], Loïc Briand[1], Christian Salles[1]*

[1]Centre des Sciences du Goût et de l'Alimentation, CNRS, INRAE, Institut Agro Dijon, Université Bourgogne Europe, F-21000 Dijon, France
[2]PROBE ChemoSens Platform, Dijon, France

Effervescent nutritional supplements, while offering advantages such as improved bioavailability and ease of ingestion, often suffer from poor sensory qualities due to the presence of vitamins, minerals, and amino acids known for their bitter, astringent, and metallic tastes. This study aimed to systematically evaluate and characterize the sensory profiles of four effervescent formulations using a trained panel and quantitative descriptive analysis (QDA). Sensory attributes were assessed across three perception modalities: orthonasal smell, retronasal aroma during tasting, and aftertaste. Products differed significantly in both aromatic and gustatory dimensions. Bitterness, metallic sensation, and astringency were identified as major negative attributes, particularly in products enriched with specific minerals. Flavouring agents showed differential effects on sensory perception, where fruity and citrus notes often enhanced sweetness and masked unpleasant tastes via cross-modal interactions. Blocking retronasal airflow using nose clips confirmed that these odour-induced effects notably influenced sweetness and reduced bitterness and astringency. However, in some cases, flavouring also unexpectedly intensified metallic sensations. The results highlight the complex interplay between taste and aroma perception in these products and underscore the need for tailored flavour strategies. Understanding these interactions opens promising avenues for improving consumer acceptance of nutritional supplements through reduced reliance on additives or encapsulation, thereby enhancing both palatability and nutritional alignment.

**Funding:** This research was funded by Bayer Healthcare SAS and ANRT (National Agency for Research and Technology, France).

**abstract citation id: bjaf055.178**

### Activity-dependent plasticity in the mouse olfactory bulb: effects of prolonged enrichment

*Harin Wijayathunga[1], Elisa Galliano[1]*

[1]University of Cambridge. United Kingdom

Activity-dependent plasticity has been well-described in the various cell types of the murine olfactory bulb (OB) as it adapts to prolonged changes in odour space. These changes span the bulb, from modulation of the input from the olfactory sensory neuron (OSN), to modulation of activity, synaptic strength, intrinsic excitability, and structure of the principal mitral and tufted cells (M/TCs) and inhibitory dopaminergic (DA) interneuron populations. However, much of this picture comes from the case of prolonged olfactory deprivation, and little is known about plasticity under its inverse, olfactory enrichment.

Here, we describe a robust passive olfactory enrichment protocol in which adult mice of either sex were exposed to a panel of eight monomolecular odorants in their individually-ventilated home cage for durations spanning from a day to a week.

Immunohistochemical analysis and confocal microscopy confirmed that the odorant panel was broad enough to activate the OB glomerular map in a non-spatially clustered manner, allowing for random sampling.

By comparing enriched mice to control littermates, we compared changes in OSN input in OB glomeruli using vesicular glutamate transporter type 2 (VGlut2) staining intensity and proportional labelling. In M/TCs and dopaminergic interneurons, activity level was explored by comparing staining intensity of immediate early gene cFos, with further confirmation in DA cells by measuring tyrosine hydroxylase (TH) fluorescence. Finally, Ankyrin G (axon initial segment (AIS) protein) staining in M/TCs and DA cells allowed for 3D tracing of the AIS, a marker of structural plasticity which is closely linked to intrinsic excitability.

Preliminary analysis indicates a structural plasticity phenotype in the AISes of DA interneurons. This analysis suggests an asymmetrical manifestation of plasticity compared to olfactory deprivation, thus prompting a reconsideration of how bidirectional homeostatic plasticity operates in sensory systems.

**abstract citation id: bjaf055.179**

### The limited capacity of olfactory working memory

*Shirley Lim[1], Artin Arshamian[1], Johan N. Lundström[1], Mikael Lundqvist[1]*

*[1]Karolinska Insitutet. Sweden*

Working memory (WM) is a core cognitive function that enables the temporary maintenance of information, but its capacity is inherently limited. In three experiments, we examined the capacity limits of olfactory WM using three memory paradigms: the delayed match-to-sample task, Sternberg's paradigm and N-back task. The first and second experiments investigated whether a single odor could be retained in WM over different delay periods. On each trial, an odorant (item) was presented, followed by another odorant (probe) after one of three delays (6s, 12s, 24s). Although recognition performance declined with increasing delay, it remained significantly above chance, demonstrating that humans can maintain an odor object representation in short-term memory. Experiment 2 employed Sternberg's paradigm to assess the capacity limits.

Participants studied arrays of 1, 2, or 4 odorants and then judged whether a subsequent probe matched any item in the set. Using Cowan's K to measure capacity quantitatively, we found that the capacity of olfactory WM is limited to approximately 2-3 odor objects. Finally, the third experiment employed 2-back task to probe the ability to manipulate these items held in WM. Despite the added executive demands, participants can update and manipulate information in WM. Taken together, these results demonstrate that, although olfactory WM capacity is smaller than that reported for vision or audition, humans nonetheless can maintain and manipulate multiple odor object representations in WM.

**Funding:** Funded by ERC

**abstract citation id: bjaf055.180**

### Beyond odorants: a fragment-based approach to discover ligands for human odorant receptors

*Rafaella Naressi[1], Frederico Gueiros[1], Bettina Malnic[1]*

*[1]University of São Paulo. Brazil*

The majority of human odorant receptors (ORs) remain orphan, with no known activating ligands. Functional screenings to deorphanize ORs typically rely on odorants—volatile, low molecular weight molecules naturally associated with smell perception. Although odorants are chemically diverse, many ORs remain orphan even after extensive testing. Some of these receptors are also expressed ectopically, which may mean that their preferred ligands could include endogenous molecules that are not like traditional odorants. In this context, we explored an alternative approach using chemical molecular fragments—small synthetic compounds, some of which are structurally inspired by natural products ("natural-like"), but that are not necessarily volatile and are typically used for drug screenings. We screened two curated libraries containing 4,480 such fragments against two variants of a selected human OR, using a heterologous expression system coupled to a cAMP- dependent luciferase reporter assay. Our results show that multiple fragments elicited receptor activation, demonstrating that synthetic chemical fragments can engage human ORs and serve as a complementary strategy for ligand discovery. This approach expands the chemical space beyond traditional odorants and may help uncover novel ligands for ORs that remain unresponsive in standard odorant-based assays.

**abstract citation id: bjaf055.181**

### Sweetness preference in a multi-lab global population: a replication study

*Jonas Yde Junge[1], Ha Nguyen[2], May M. Cheung[3], Keiland W. Cooper[4], Group author See note 1 below, Global Consortium for Chemosensory Research[5], Valentina Parma[2], Maria Geraldine Veldhuizen[6], Surabhi Bhutani[7]*

*[1]Aarhus University. Denmark*
*[2]Monell Chemical Senses Center. United States*
*[3]City University of New York - Brooklyn College. United States*
*[4]University of California, Irvine. United States*
*[5]Global Consortium for Chemosensory Research*
*[6]Mersin University. Turkey*
*[7]San Diego State University. United States*

High free sugar intake contributes to the global epidemic of obesity and related metabolic disorders, which may be mediated by a higher sweetness preference. A solution to mitigate these risks is to reduce the amount of added sugar in foods; however, this may compromise their overall acceptability. More research is needed into the variation and flexibility in sweetness preferences. In 2011 Mennella et al. developed a fast psychophysical test (the Monell Forced-Choice Paired- Comparison, MPC) to assess an individual's sucrose preference. They showed that sweet preference is related to the sugar content of preferred and frequently consumed foods and beverages. However, whether these results generalize beyond an urban United States (US) population is unclear. We conducted a large-scale global data collection to replicate and extend those observations. Methods: i) the MPC to measure sweetness preference, ii) a survey to assess the liking and intake frequency of foods, iii) available global data on consumption of sugar-sweetened beverages, and iv) preferences for commercial sweetened beverages. Data were collected from 989 women and 777 men across 31 laboratories in 20 countries, including laboratories in Africa, Asia, Europe, North America, Oceania, and South America (data collection ongoing). Preliminary analyses show a similar reliability of the MPC as in the study of Mennella, 2011 (intraclass correlation = 0.56). An average (m) sucrose preference of 12.5 %w/v (standard deviation, sd: 8.2) was observed in the MPC. We observed a significant effect of self-reported sex at birth; women (m=11.2, sd=8.1) prefer lower concentrations of sucrose than men (m=14.2, sd=8.9) ($F(1,1764) = 53.09$, $p < .001$). These preliminary results show that our global sample of women prefers a lower concentration of sucrose relative to the US women in the original study by Mennella et al. (m=14.4, sd=10.4), and also relative to a global sample of men.

### Reference

1 GCCR group author: Maame Yaakwaah Blay Adjei, University of Ghana; Zeynep Altınkaya, Karamanoğlu Mehmetbey University; Glenn Andersen, Aarhus University; Lisa Arvieu, Queensland University of Technology; Adeola Eniola Ayano, Bells University of Technology, Ota Ogun State Nigeria; Niko A. Busch, University of Münster; İlkim Büyükgüdük, Mersin University; Eva M. Čad, Wageningen University and Research; Samira Casanueva, Universidad de Chile; Géraldine Coppin, UniDistance Suisse; Lívia de Oliveira, Universidade de Brasília ; Valerie B. Duffy, University of Connecticut; Florian Ph.S Fischmeister, Medical University of Vienna; Wambura C. Fobbs, Swarthmore College; Marcia A. Gularte, Federal University of Pelotas; Tuan Hoang, Hanoi University of Science and Technology; Joanne Hort, Feast, Massey University; Patrice A. Hubert Hubert, Monell Chemical Senses Center; Phuong Quyen Huynh, HUTECH University ; Liang-Dar Hwang, The University of Queensland; Teodor Jernsäther, Stockholm University; Nilmini J. Karunarathna, Wayamba University of Sri Lanka; Ulla Kidmose, Aarhus University; Maria A. Klyuchnikova, Severtsov Institute of Ecology & Evolution; Ryann Kolb, Monell Chemical Senses Center; Svetlana Kopishinskaia, CHU de Quebec-Universite Laval; Elisabeth Koussissi, University of West Attica; Ilya G. Kvasha , Severtsov Institute of Ecology & Evolution; Tatiana K. Laktionova, Severtsov Institute of Ecology & Evolution; Zhibin Liu, Fuzhou University; Monica Mars, Wageningen University and Research; Chloe V. Mayo, Brunel University London and University of Reading; Ellen M. Menezes Ayres, Federal University of the State of Rio de Janeiro; Lisa Methven, University of Reading; Yuko Nakamura, The University of Tokyo; Chathumalika M. Narampanawa , Wayamba University of Sri Lanka; Marta Navarro, The University of Queensland; Dzung Hoang Nguyen, Ho Chi Minh-city University of Technology ; Thanh B. Nguyen , Industrial University of Ho Chi Minh City; Alexia Nunez-Parra, Universidad de Chile; Kathrin Ohla, dsm-firmenich; Jonas K. Olofsson, Stockholm University; Larisa Olteanu, UniDistance Suisse; Mikaela Pal, Stockholm University; Jane K. Parker, University of Reading; Mei Peng, University of Otago; H. Thinh Pham, Ho Chi Minh City University of Technology; Phuong D.L. Pham, Industrial University of Ho Chi Minh City; Kumari M. Rathnayake, Wayamba University of Sri Lanka ; Danielle R. Reed, Monell Chemical Senses Center; Eugeni Roura, The University of Queensland; Michael C-K. Ryoo, University of Queensland; Yee-How Say, Sunway University; Stefan Schulreich, University of Vienna; Charlotte Sinding, Centre des Sciences du Goût et de l'Alimentation; Fernanda Spada, University of São Paulo; Thierry Thomas- Danguin, Centre des Sciences du Goût et de l'Alimentation; Vera V. Voznessenskaya, Severtsov Institute of Ecology & Evolution; Sumeia Werfalli, Libyan International Medical University/ University of Benghazi; Theresa L. White, Le Moyne College; Berçem Yar, Mersin University;

**abstract citation id: bjaf055.182**

### Identification of novel TAS2R4 agonists by screening of a natural compound library

*Sara Montelatici[1], Marcel Winnig[1], Bernd Bufe[2]*

[1]*Axxam SpA. Italy*
[2]*Kaiserslautern University of Applied Sciences. Germany*

The NatPure compound library, one of the largest collections of pure natural compounds, comprises around 15,000 molecules from diverse plant and microbial origins. It's chemical diversity and the fact that about 30% of its compounds are novel and unpublished, makes it a unique resource for drug discovery and taste research. The present study illustrates a screening campaign to identify novel TAS2R4 ligands and identified new compounds from different classes activating TAS2R4. Interestingly, some of the newly identified compounds do not contribute to bitterness perception. This result supports different TAS2R4 signaling properties and may suggest physiological effects in disparate organs in which the receptor is expressed. This opens new research avenues for understanding the broader physiological roles of taste receptors and the therapeutic potential of these non-bitter agonists in both pharmaceutical applications and in the food industry.

**abstract citation id: bjaf055.183**

### Effects of cognitive behavioral therapy on the perception and worry of own body odor in Olfactory Reference Disorder

*Mats J. Olsson[1], Volen Z. Ivanov[1], Susanna Österman[1]*

[1]*Karolinska Institutet. Sweden*

Olfactory reference disorder (ORD; a new disorder in ICD-11) is characterized by a strong belief in emitting an unpleasant body odor from one's body, such as armpits, mouth or genitalia. This condition is stressful and associated with an increased risk of suicide, but no treatment guidelines are available. We first conducted a pilot study evaluating the feasibility and preliminary efficacy of internet-based cognitive behavioral therapy (iCBT), which indicated promising results: symptom reduction in ORD with a large effect size (>2.0), and 80% of participants achieving at least a 30% decrease in symptom severity. Notably, 47% of participants no longer met diagnostic criteria for ORS at post-treatment. Patient satisfaction was high and no serious adverse events were reported, supporting the feasibility of the intervention.

Based on these findings, we have now launched a preregistered randomized controlled trial (RCT, n=60) evaluating 10 weeks of iCBT for ORD. The study will also examine which types of body odors that are most common in ORD reports and also to what extent the self-perception of own body odor or rather worry for smelling bad is the largest problem to this group. As part of the pilot study, we also collected body odor pads from the ORD and control participants which will be perceptually evaluated by a separate group of participants (n=30) to reveal whether there actually are any differences in body odor between ORD and controls.

**Funding:** Swedish Research Council (Swedish Research Council: 2020-02567 to MO)

**abstract citation id: bjaf055.184**

### Adolescents aged 12-18 years score similarly in the Sniffin' Sticks Screening 12 Test and U-Sniff tests

*Katarzyna Resler[1], Katarzyna Fułek[1], Monika Augustyn[1], Ewelina Bobak-Sarnowska[1], Anna Oleszkiewicz[2,3], Tomasz Zatoński[1]*

*[1]Department of Otolaryngology, Head and Neck Surgery, Wroclaw Medical University. Poland*
*[2]University of Wrocław, Faculty of Historical and Pedagogical Sciences, Institute of Psychology, Wroclaw, Poland*
*[3]Interdisciplinary Center Smell & Taste,Department of Otorhinolaryngology, Faculty of Medicine Carl Gustav Carus, Technische Universität Dresden. Germany*

**Objectives:** The epidemiology of olfactory disorders in the pediatric and adolescent population is not well defined, primarily due to underreporting, the lack of routine olfactory testing in children, and the tendency of young children to not recognize or articulate olfactory deficits. Testing children requires standardized, validated olfactory tests specific to this age group. However, dedicated tests are not always available in diagnostics and the age of the patient may allow to adopt tests designed for adults. The study aimed to evaluate the Sniffin' Sticks Screening 12 Test scores applied in an adolescent population (but designed for adults) and compare them with the scores in the U-Sniff test designed for children.
**Methods:** A group of 85 children aged 12-18 years (Mean=15,7, SD=2,1; 41 men) were examined using two olfactory identification tests the Sniffin' Sticks Screening 12 Test and the U-Sniff test. In addition, the subjects completed the Individual Significance of Olfaction Questionnaire (the question about kissing a partner was omitted) and the Sense of Smell Importance Questionnaire in the areas of eating, forming bonds, and avoiding danger.
**Results:** Within the U-Sniff test, the smell of butter was the most difficult, obtaining 41.2% of correct answers. The other smells ranged from 63-98%. For the Sniffin' Sticks Screening 12 Test, only two items scored lower at 60 and 62.4% for leather and pineapple, respectively. There were no gender differences in odor identification performance. No significant correlation emerged between odor identification test results and questionnaire responses.
**Conclusions:** The use of the the Sniffin' Sticks Screening 12 Test and Test U-Sniff in the adolescent population can be used interchangeably, with attention to single items in both tests.
**Funding:** This research was funded by Wroclaw Medical University SUBZ.C250.24.088.

**abstract citation id: bjaf055.185**

### Smell and Taste Distortions in U.S. Adults: Prevalence and Risk Factors during the COVID-19 Pandemic

*Chuan-Ming Li[1], Howard J. Hoffman[1], Le Chen[1], Shristi Rawal[2], Susan E. Coldwell[3], Diana Fisher[4], John Hayes[5], Valerie B. Duffy[6]*

*[1]Epidemiology, Statistics, and Population Sciences Section, National Institute on Deafness and Other Communication Disorders (NIDCD), National Institutes of Health (NIH). United States*
*[2]Department of Clinical and Preventive Nutrition Sciences, Rutgers School of Health. United States*
*[3]School of Dentistry, University of Washington. United States*
*[4]National Eye Institute (NEI), National Institutes of Health (NIH). United States*
*[5]Sensory Evaluation Center, College of Agricultural Sciences, The Pennsylvania State University. United States*
*[6]Department of Allied Health Sciences, University of Connecticut. United States*

**Background:** Phantosmia or parosmia and parageusia or dysgeusia are sensory distortions that can lower quality of life and impair eating, sleeping, and emotional well-being.
**Methods:** The 2021 National Health Interview Survey (NHIS) included questions about smell and taste from 29,482 U.S. adults (≥18 years). We estimated the prevalence of distortions of smell and taste and their associations with COVID-19 and other risk factors. Estimates were adjusted for the complex random sampling design and logistic regression was used after adjusting for age, sex, race/ethnicity, education, income, and geographic area.
**Results:** A history of COVID-19 was reported by 14.2%. Prevalence of altered sensation was 19.7% for smell and 13.3% for taste. Altered sensation occurred more frequently among those with COVID-19 (50.7% and 41.3%, for smell and taste, respectively). Distortions of smell and taste were reported by 4.7% and 2.9% overall, and by 12.4% and 7.6% among those with

COVID-19. Prevalences increased greatly with COVID-19 symptom severity for smell (3.1% to 19.7%) and taste (2.7% to 15.5%), from no symptoms to severe symptoms. Prevalences peaked among those with partial recovery of smell (30.2%) or taste (29.2%). Smell and taste distortions were more frequent with female sex, lower income, cigarette smoking, poorer health, obesity, recent cold/flu, asthma, dry mouth, hypertension, depression/anxiety, medication use, immunosuppression, and cognitive decline. Among adults aged ≥40 years with distortions of smell and taste, 20.6% and 38.6%, respectively, reported discussing their symptoms with a health care provider; proportions were higher among those with COVID-19 (37.3% and 52.9%).

**Conclusions:** Smell and taste distortions were prevalent among adults, particularly those with COVID-19, poorer health, and certain demographic factors. Findings highlight the need for greater clinical attention and research on assessment, management, comorbidities, and prevention.

abstract citation id: bjaf055.186

### The importance of smell and its functions in humans: a comparison with other senses and other activity domains in France

*Typhaine de Simone[1], Eric Angelini[2], Malick Diop[2], Moustafa Bensafi[1]*

*[1]Centre de Recherche en Neurosciences de Lyon. France*
*[2]MANE. France*

Since Antiquity and Aristotle's hierarchy, olfaction has been considered as the least important sense, a belief reinforced by centuries of philosophical and scientific discourse in various fields. In recent decades, this assumption has been challenged by research into the significance of olfaction. The present study aims to clarify the role of olfaction in daily life by comparing its perceived value to other sensory modalities and common life commodities. An online study was conducted in 130 normosmic respondents in France (97 women, 33 men, aged 18-79), measuring willingness to lose each sense and ranking the importance of chemical senses to a range of everyday items (e.g. salary, living space, social media, streaming platforms, smoke detectors, perfume, travel, sports, car, phone, pets, friends, hair and little toe). Results confirm the dominance of distal senses over proximal ones (p<0.001). This result may be explained by the values of Western societies, where vision and hearing are culturally associated with anticipation, and knowledge acquisition. Olfaction ranks in the middle of the sensory hierarchy due to its position as both a proximal and distal sense. Yet, its functions, particularly those linked to food, social communication, and danger avoidance, are valued more than several commodities (p<0.05 in all cases) but are ranked equally to affective terms (friends, pets), body parts (hair, toe) and leisure (travel, sports). Among olfactory functions, food-related seems most important, followed by those supporting social and protectives roles. These findings suggest olfaction hold a more significant role in daily life than traditionally assumed, despite persistent visual dominance. Our next steps will examine how culture and individual olfactory profile (e.g. patients or experts) influence these valuations.

**Funding:** This study is a part of Typhaine de Simone's PhD project, funded by an ANRT CIFRE research program between Université Claude Bernard Lyon 1 and MANE.

abstract citation id: bjaf055.187

### Central olfactory responses to odor repetition on the seconds timescale

*Maria Grazia Fioriello[1], Ian F Chapman[2], Kevin Bolding[2], Michele Dibattista[1]*

*[1]University of Bari Aldo Moro. Italy*
*[2]Monell Chemical Senses Center. United States*

Olfactory cues in natural environments exhibit wide concentration ranges and dynamic temporal patterning, requiring neural processes to adjust according to recent stimulus history to optimize sensitivity. Short timescale stimulus repetition elicits decreased responses in olfactory receptor neurons (i.e., adaptation), and central olfactory responses in OB and PCx are reduced after prolonged stimulus exposure.

Though peripheral and central adaptation have been studied extensively in reduced and anesthetized preparations, observations in the intact, awake brain remain rare. This gap constrains our ability to link the perceptual and behavioral consequences of repeated odor exposure to their underlying neural mechanisms. To characterize responses to odor stimulus repetition, we acquired neural population recordings from OB (n = 563 units) and PCx (n = 1128 units) of awake, head-fixed mice responding to odor pulses with varying interpulse intervals (IPIs: 2, 4, 8, 16, 32 s). We selected odors to overlap with previous work on ORN and OB adaptation in awake mice (Storace & Cohen, 2021). First pulse responses exhibited odor sensitivity consistent with previous reports (~10-20% of cells in both regions responding with activation or suppression).

Second pulse responses in both regions exhibited adaptation at 2 s (OB: 16.2%; PCx: 7.5% of cells) that gradually recovered over the next 30 s, with greater overall adaptation and steeper recovery in OB.

Surprisingly, second pulse responses at short intervals significantly increased for a substantial subset of neurons in both regions (OB: 11%; PCx: 9% of cells at 2 s). Our results reveal that, unlike the predominantly suppressive responses of ORNs, central circuits exhibit both adaptation and facilitation, suggesting an active rebalancing of network output. This reorganization may support computations that track temporal structure in odor signals while preserving dynamic range.

**Funding:** Monell Chemical Senses Center Institutional Funds (MIF); NIH/NIDCD: 5R01DC020927

### Reference

1 Storace DA, Cohen LB. The Mammalian Olfactory Bulb Contributes to the Adaptation of Odor Responses: A Second Perceptual Computation Carried Out by the Bulb. eNeuro. 2021 Sep 24;8(5):ENEURO.0322-21.2021. doi: 10.1523/ENEURO.0322-21.2021. PMID: 34380657; PMCID: PMC8474650.

**abstract citation id: bjaf055.188**

### Niemann-Pick type C1 mouse models show impaired lipid profile and microglia activation

*Maria Grazia Fioriello[1], Donatella Lobraico[1], Raffaella Pia Gatta[1], Johannes Reisert[2], Simona Lobasso[1], Antonio Frigeri[1], Michele Dibattista[1]*

*[1]University of Bari Aldo Moro. Italy*
*[2]Monell Chemical Senses Center. United States*

Niemann-Pick type C1 (Npc1) is a rare neurodegenerative disorder linked to altered cholesterol biosynthesis and irregular lipid regulation. In patients, the most common variant in the Npc1 is a missense mutation (I1061T) that leads to a faulty Npc1 protein and consequently to cholesterol accumulation in lysosomes and its impaired synthesis and metabolism. We tried to better understand what happen to the olfactory system in a mouse model bearing the most common missense mutation found in human patients, namely the Npc1 (I1061T) mouse model. Our preliminary data show that neurodegeneration in the olfactory epithelium (OE) becomes dramatic at two months of age and involves mainly mature OSNs while leaving intact the regenerative ability of stem cell niches in the OE. In addition, we performed positive ion MALDI- TOF MS analysis and found that phosphatidylcholine (PC) 36:4 and 38:4 and PC/LPC ratio increase in mutant mice (MUT) in OE after only one month of age, while negative ion analysis showed an increase in phosphoinositol (PI) and a decrease in sphingomyelin (SM) 16:0 and phosphatidic acid (PA) 34:1 in MUT at two months of age. Since LPC alterations levels and PC/LPC ratio are key markers in neuronal damage and microglia-mediated inflammatory response, we then investigated the neuroinflammation and microglia state. We found that MUT mice present impaired macrophages activation, morphology and localization in the olfactory epithelium (OE) after only one month of age. We noticed an abundant number of phagocytic cups in MUT which are very different from the ramified phagocytes in wild-type mice. It is possible that the intrinsic ability of microglial cells to migrate and actively phagocytes is affected by the lack of Npc1 and the lysosomal accumulation of cholesterol. Our results demonstrate that cholesterol dysmetabolism and neuroinflammation in Npc1 disorder are remarkable pathological hallmarks that exacerbate the progression of neuronal dysfunction.
**Funding:** Regional funding

**abstract citation id: bjaf055.189**

### Taste and smell disorders in U.S. adults: prevalence, risk factors, and associations with health care

*Howard J. Hoffman[1], Diana Fisher[2], Shristi Rawal[3], Susan E. Coldwell[4], Chuan-Ming Li[1], John E. Hayes[5], Valerie B. Duffy[6]*

*[1]National Institutes of Health (NIH), National Institute on Deafness and Other Communication Disorders (NIDCD), Bethesda, MD 20892. United States*
*[2]National Institutes of Health (NIH), National Eye Institute (NEI), Bethesda, MD 20892. United States*
*[3]Rutgers University, School of Health Professions, Newark, New Jersey 07107-1709. United States*
*[4]University of Washington, School of Dentistry, Seattle, WA 98195. United States*
*[5]The Pennsylvania State University, College of Agricultural Sciences, State College, PA 16802. United States*
*[6]University of Connecticut, Department of Allied Health Sciences, Storrs, CT 06269. United States*

**Background:** Taste and smell disorders are common, associated with aging, socio-economic factors, and poorer health conditions that impact negatively on overall quality of life.
**Methods:** In the U.S. National Health and Nutrition Examination Survey (NHANES), 2011-2014, taste and smell questions were administered to adults aged 40+ years (n=7,413; mean age=58 years; females, 53%). In 2013-2014, an 8-item odor identification test and taste assessment using the generalized Labeled Magnitude Scale (gLMS) to rate whole mouth and tongue tip perceived intensities of bitter and salt solutions was added (n=3,219). Hypogeusia and hypergeusia were defined by cut points for 10th and 90th percentiles for younger adults, aged 40-44 years. Dysgeusia was the report of unwanted persistent tastes in the mouth during the past year. Hyposmia was defined by low odor identification scores. Associations between taste or smell disorders with risk factors and health care use were examined using survey weighted logistic regression.
**Results:** Prevalences of any smell problem, any taste problem, and dysgeusia were 22.3%, 14.9%, and 5.3%, respectively. Women were more likely to have dysgeusia and hypergeusia and less likely to have hypogeusia compared to men ($p<0.01$). Dysgeusia was associated with Hispanic ethnicity, less education, obesity, smoking, hypertension, diabetes, arthritis, asthma, cancer, persistent cold/flu, dry mouth, nasal congestion, and frequent sinus or ear infections. Dysgeusia decreased from 11.2% to 2.4% for reported poor vs. excellent oral health, while hypogeusia increased from 13.8% to 17.7% (both $p<0.05$). Dysgeusia was associated with frequent aching in the mouth (18.6%) vs. never (3.1%). Individuals with taste disorders (hypogeusia or dysgeusia) were more likely to seek health care compared to those with smell disorders.
**Conclusions:** Understanding differences in modifiable factors is key to preventing chemosensory disorders and improving their clinical management.

**abstract citation id: bjaf055.190**

### Flexible intramolecular disulfide bonding strategies in mammalian odorant receptors

*Nonoko Muto[1], Tomoyo Koshizawa[1], Miki Takeda[1], Ryosuke Inoue[1], Masafumi Yohda[1], Hiroaki Matsunami[2], Yosuke Fukutani[1]*

*[1]Tokyo University of Agriculture and Technology. Japan*
*[2]Duke University. United States*

Mammals detect a vast array of odorant molecules via odorant receptors (ORs), a large subfamily of G protein–coupled receptors (GPCRs). In many GPCRs, intramolecular disulfide bonds between or within extracellular domains are critical for proper structural formation. However, certain GPCRs, including some ORs, lack one of the conserved cysteine residues in extracellular loop 2 (ECL2), leaving the role of these bonds unclear. Here, we reveal the functional importance of a unique cysteine residue in the N-terminal region of an OR that compensates for the missing ECL2 cysteine and modulates receptor expression and activity. In ORs lacking the conserved ECL2 cysteine, a cysteine in the N-terminal domain may form an alternative disulfide bond, as observed in OR51E2, the only native mammalian OR with an experimentally determined structure[1]. We demonstrate that the N-terminal cysteine in OR51E2 is essential for full ligand-induced responsiveness in heterologous cells. Moreover, per-residue confidence scores from AlphaFold for cysteine mutants of ORs correlate with both surface expression and receptor function, suggesting the role of alternative disulfide bond–mediated structural stabilization. We propose that ORs, and potentially other GPCRs, utilize flexible disulfide bonding strategies involving extracellular cysteines, including those in the N-terminus, to achieve proper folding and function.

**Funding:** This work was supported by JSPS-KAKENHI, JST ACT-X, Program on Open Innovation Platform with Enterprises, Research Institute and Academia, and JST SPRING.

### Reference

1 Billesbolle, C. B. et al. Structural basis of odorant recognition by a human odorant receptor. Nature (2023)

**abstract citation id: bjaf055.191**

### Habituation to saltiness in unimodal and crossmodal perceptions

*Lucie Ben-Sussan[1], Thibault Chabin[1], Thierry Tomas-Danguin[1], Charlotte Sinding[1]*

*[1]Centre des Sciences du Goût et de l'Alimentation, CNRS, INRAE, Institut Agro, Université Bourgogne Europe, F-21000 Dijon, France*

We investigated whether saltiness perception decreases with repeated exposure to a salty stimulus, a phenomenon referred to as sensory habituation. If habituation occurs, it might promote higher salt intake to compensate for taste attenuation. Secondly, we assessed whether a congruent aroma—smoky bacon—could attenuate this habituation.

The experiment was initially designed for EEG; however, only the sensory results are presented here. Stimulations were delivered via a gustometer on the extended tongue and saltiness was rated on a vertical continuous scale from "very low" to "very high." in the course of the blocks. The protocol consisted of three blocks: **1)** participants received short pulses (500 ms) of 2% NaCl every 2.5 s, repeated 40 times, embedded in water pulses to reduce somatosensory artifacts. The sequence was repeated five times, with 1-minute breaks; **2)** they received pulses of 1% NaCl during 20.4 s; and had to held the solution in mouth for another 10 s. This was repeated 20 times (1-minute intervals); **3)** same as Block 2, but with 0.03% smoky bacon aroma added. In **Block 1**, a slight but significant saltiness habituation occurred ($-0.71 \pm 0.20$ units on a 10-point scale; $z < -5.13$, $p < 0.001$), sustained across trials despite recovery periods. In **Block 3** compared to **Block 2**, the addition of the smoky bacon resulted in a strong increase in initial saltiness perception compared to the salt-only condition ($+2.71 \pm 0.39$ units). However, stronger habituation was found in the crossmodal condition compared to unimodal ($-1.32 \pm 0.36$ units; $z < -6.56$, $p < 0.001$). Saltiness perception habituates over short repeated exposures. The addition of a congruent aroma amplifies initial saltiness but also accelerates habituation, suggesting that olfactory habituation may be more pronounced than gustatory. This raises considerations for aroma-based salt-reduction strategies.

**Funding:** *This research was supported by a grant from French Research National Agency (ANR) [Grant N° ANR-19-CE21-0009].*

**abstract citation id: bjaf055.192**

### Smell, self, and sustenance: exploring sensory and emotional correlates of deviant eating behavior

*Ekaterina Rzhevskaia[1], Ilja Croijmans[1], Laura Speed[1], Margot van Mulken[1]*

*[1]Center for Language Studies, Radboud University. The Netherlands*

Previous research has shown lowered olfactory functions and odor discrimination in individuals with eating disorders. However, little is known about people with deviant eating behavior (DEB), a potential subclinical precursor to eating disorders. This study, part of a larger project, investigates the association between individual differences in eating behavior and self-reported food relationships, olfactory perception, sensory imagery, and body image.

This cross-sectional, mixed-methods study uses continuous measures of eating behavior patterns to explore links with scores from other questionnaires. Now, 97 participants completed an online survey assessing their sensitivity to food-related disgust (FDS), body perception (BISS), olfactory awareness (OAS-6), vividness of sensory imagery (Psi-Q), and eating behavior patterns (TFEQ-18).

We hypothesize that individuals' higher emotional scores on scales will indicate more extreme patterns of eating behavior. We expect DEB participants to have higher scores on the Food Disgust Scale while having lower scores on the Positive Eating Scale. It is also expected that they will have lower scores on both the Odor Awareness Scale and the Body Image State Scale, suggesting lowered smell perception and greater body dissatisfaction, respectively, due to reduced engagement with sensory input and elevated body concerns, as commonly reported in people with disordered eating tendencies. We also predict higher scores on the emotional eating, uncontrolled eating, and cognitive restraint subscales of the Three Factor Eating Questionnaire among DEB participants, as these measurements reflect regulatory and cognitive control strategies that are often reinforced in disordered eating cases.

We expect the findings to shed light on sensory factors involved in DEB reflected in an emotional relationship with food, odors processing, imagery, and the associations they evoke.

**Funding:** Starting grant from Radboud University granted to Dr. Ilja Croijmans

**abstract citation id: bjaf055.193**

### Microprism-based two-photon imaging allows in vivo monitoring of mouse AOB mitral cell activity

*Franke Melissa[1], Yoram Ben-Shaul[2], Spehr Marc[1]*

*[1]Department of Chemosensation, Institute for Biology II, RWTH Aachen University, Aachen. Germany*
*[2]Department of Medical Neurobiology, Institute for Medical Research Israel Canada, Faculty of Medicine, The Hebrew University of Jerusalem, Jerusalem, Israel*

In mammals, the accessory olfactory system is essential for pheromone detection and processing, thus controlling innate behaviors. The accessory olfactory bulb (AOB) is the system's first central processing structure, receiving sensory input from vomeronasal neurons and relaying information to higher brain areas, including amygdala and hypothalamus. AOB mitral cells (AMCs) are the sole projection neurons of the AOB. Their activity is shaped by a complex network of local interneurons. Previous studies in acute slices have shown that AMCs exhibit spontaneous infra-slow oscillatory activity and form microcircuits within the AOB network. However, in vivo investigation of AMC population activity has proved challenging.

Here, we present a novel approach to perform network-scale $Ca^{2+}$ imaging of AMCs using two-photon microscopy. We selectively label AMCs with genetically encoded $Ca^{2+}$ indicators of the GCaMP family. Next, inserting a microprism into the frontal cortex of anaesthetized mice enables AMC recordings. Thus, we characterize spontaneous AMC firing patterns in vivo. We describe kinetics of AMC $Ca^{2+}$ transients and compare these data to previous studies in acute slices. Together, we show that microprism-based two-photon imaging allows for robust in vivo imaging of AMC population activity and provides the basis for future in vivo investigation of the mouse AOB circuitry.

**abstract citation id: bjaf055.194**

### The role of age-related white matter lesions in odor identification

*Adei Roel[1], Eloy Garcia-Cabello[1], Xin Li[2], Jonas Persson[2], Nira Cedres[1], Jonas Olofsson[3]*

*[1]Fernando Pessoa Canarias University. Spain*
*[2]Aging Research Center (ARC), Karolinska Institute. Sweden*
*[3]Stockholm University. Sweden*

White matter lesions (WML) as a biomarker of cerebrovascular damage are known to increase with age affecting the integrity of white matter (WM) projections; however, the contribution of age-related WML in the brain to olfactory functioning remains uncertain. We aimed to determine how baseline levels WML mediate the association between age and WM fibers' neurodegeneration at five years follow-up. Also, we studied the interaction between baseline age-related WML volumes and follow-up WM fibers' neurodegeneration in the prediction of odor identification performance. A sample of 370 adults from the general population (mean age 62.4; 49.1% men), from the Swedish Betula cohort were tested at baseline with 178 individuals also tested at five years follow-up. Participants underwent odor identification testing and magnetic resonance imaging including T1-weighted-3D for WML volume, and Diffusion Tensor Imaging for WM fiber integrity. Mediation and interaction models were used to test our hypothesis. Baseline WML were a significant mediator when predicting follow-up WM integrity, showing that older age association with increased WML volumes partially predicted poorer WM integrity at follow-up. Age-related WML and their interaction with WM integrity significantly predicted odor identification performance at follow-up, showing that individuals with greater baseline WML volumes and poorer WM integrity performed significantly worse compared to those with average or lower WML. These preliminary data show complex associations WML and odor identification performance, highlighting the potential of olfactory testing as an early and accessible marker of underlying cerebrovascular damage in the general population. WML volume are an important contributor to aging trajectories, underscoring the demand of preventive strategies over cerebrovascular risk factors in the aging population. Future analyses focused on location-dependent impact of WML on olfactory function are needed.

**abstract citation id: bjaf055.195**

### Automated in vitro platform for the development of repellents targeting insect olfactory receptors

*Guillaume Audic[1], Justine Magnat[1], Franck Pelissier[2], Claude Grison[2], Christophe Moreau[1]*

[1]Institut de Biologie Structurale. France
[2]Laboratoire de Chimie Bio-inspirée et d'innovations écologiques. France

In recent decades, insects carrying emerging diseases have been spreading throughout the world. The tiger mosquito – Aedes albopictus – is a prime example. Thanks to his environmental adaptability, it has spread from Asian forest to tropical regions. Uses of repellent is one of the main public strategies to avoid mosquito's bites. DEET is a synthetic powerful repellent largely use, however its toxicity combined with first signs of resistance from mosquitos are a cause for concern. Natural alternatives like essential oils are emerging on the market but have shown limited effectiveness over time. New healthy, efficient and friendly environmental natural compounds are desired to fight against this threat.

In the screening pipeline of compounds, primary screening methods of compounds prior to test on mosquitoes are being sought. We initiated the development of an in vitro platform based on automated electrophysiological recordings and insect olfactory receptors targeted by repellents. Using this method, we identified one olfactory receptor as a general target of standard and innovative repellents and performed quantitative assessment of compound efficacy.

**abstract citation id: bjaf055.196**

### Odor induced autobiographical memories: AI based recognition of facial expressions

*Kwangsu Kim[1], Antonia von Schroeter[1], Anne Abriat Hemmendinger[2], Antje Hähner Mueller[1], Jaime Ferreira[3], Thomas Hummel[1]*

[1]Smell and Taste Clinic, Department of Otorhinolaryngology, TU Dresden. Germany
[2]The Smell & Taste Lab. Switzerland
[3]The Estee Lauder Companies. United States

This study examined how olfactory stimuli affect the recall of autobiographical memories and the accompanying facial expressions. Perfumes were used as a means to elicit odor-related memories. Seventy young and healthy participants (age: 24.41, 70 women) selected their two most familiar perfumes from a set of sixteen commercially available perfumes and described related personal memories. Their facial expressions during memory recall were recorded and analyzed using a deep learning-based system combining YOLOv8 for face detection and ResNet-18 for emotion classification. Participants also completed questionnaires regarding their memories and rated the familiarity of each perfume. The results showed that perfume familiarity and the frequency with which a perfume was chosen to induce a memory varied significantly across perfumes. Among the eight classified facial expressions, "neutral" was most common, especially in the high familiarity group. "Fearful" expressions were more often related to more negative memories. Findings suggested that familiar odors induced more stable facial responses. On the other hand, less familiar perfumes or more negative memories are related to more varied facial expressions. Overall, this study indicates the potential of integrating the automated analyses of facial expressions into explorations of odor-induced memories.

**abstract citation id: bjaf055.197**

### Unveiling the Harderian gland in rabbits: morphological, histochemical, and immunohistochemical characterization of a specialized orbital structure

*Irene Ortiz Leal[1], Uxía Yáñez Ramil[1], Silvia Alejandra Fraga Abelleira[1], Renato Barrionuevo Ponce[1], Luis Ángel Quintela Arias[1], Pablo Sanchez Quinteiro[1]*

[1]University Santiago de Compostela. Spain

This study presents a comprehensive morphological, histochemical, and immunohistochemical analysis of the Harderian gland in rabbits (Oryctolagus cuniculus), a prominent orbital gland implicated in ocular protection and chemical communication. Located within the orbit, this gland plays a key role in the production of lipid- and porphyrin-rich secretions, which are believed to contribute to grooming, photoprotection, and potentially to chemosensory signaling. Despite its anatomical relevance and suggested physiological importance, the Harderian gland remains poorly characterized in lagomorphs, especially regarding its tissue organization and biochemical features.

To address this gap, we conducted a detailed histological investigation of the Harderian gland in adult rabbits of both sexes. Histochemical labeling with selected lectins was employed to examine the distribution of glycoconjugates, while immunohistochemical analysis targeted markers associated with epithelial specialization and secretory function. Our results reveal a complex glandular architecture with distinct regional variation in epithelial morphology and glycan expression patterns. Immunoreactivity profiles further support the presence of functional compartmentalization, suggesting a high degree of specialization in secretory activity.

These findings contribute to a better understanding of the structural and biochemical complexity of the Harderian gland in rabbits, highlighting its potential role in species-specific physiological and behavioral processes. This foundational work opens the path for future research on the gland's endocrine regulation, its possible involvement in chemical communication, and its response to environmental or hormonal changes.

**Funding:** Spanish Ministry of Science and Innovation (PID2021-127814OB-I00).

**abstract citation id: bjaf055.198**

### The Vomeronasal System of Talpa occidentalis: A Combined Histological, Immunohistochemical, and Lectin-Binding Approach

*Irene Ortiz Leal*[1], *Gadea García Hernando*[1], *Antía Martínez Antonio*[1], *Mostafa G. A. Elsayed*[2], *Álex Vázquez Castiñeira*[1], *Pablo Sanchez Quinteiro*[1]

[1]*University Santiago de Compostela. Spain*
[2]*Sohag University. Egypt*

The vomeronasal system (VNS) is critical for detecting pheromonal cues that modulate sociosexual behaviors. Despite its central role in chemical communication, our understanding of its anatomical and functional variability across mammals remains incomplete. This study provides the first detailed characterization of the VNS in the Iberian mole (Talpa occidentalis), a fossorial species endemic to the Iberian Peninsula. We performed a morphofunctional and neurochemical analysis of the vomeronasal organ (VNO) and the accessory olfactory bulb (AOB) using histology, immunohistochemistry, and lectin histochemistry. The VNO in T. occidentalis exhibited an unusual circular lumen lined by a uniform sensory epithelium, lacking the dual epithelial organization seen in most species. The vomeronasal cartilage was limited in extent and did not form the typical J-shaped structure. Importantly, no evidence of a vomeronasal pump was found, suggesting alternative mechanisms for semiochemical entry, likely facilitated by the organ's anatomical position and continuous receptor distribution. Immunohistochemical analysis revealed strong expression of Gαi2 and Gϒ8 in sensory neurons, with weaker Gα0 expression, suggesting predominance of V1R-type signal transduction. The AOB, though small, exhibited clear lamination and specific marker localization (Gαi2, OMP, CR, MAP2), indicating robust functional organization. Lectin binding revealed specific glycosylation patterns in the glomerular layer, with STL and LEA marking synaptic regions. These findings uncover unprecedented anatomical and molecular features in the VNS of T. occidentalis, positioning this species as a valuable model for studying vomeronasal diversity and evolution among Laurasiatherian mammals.

**abstract citation id: bjaf055.199**

### Odor Awareness Scale in Children (OAS-C) – a novel method to measure odor awareness in preschool children

*Marta Rokosz*[1], *Daniel Marek*[1], *Daniel Jędrzejczyk*[2], *Anna Oleszkiewicz*[1,3]

[1]*Institute of Psychology, University of Wrocław. Poland*
[2]*Institute of Psychology, SWPS University of Social Sciences and Humanities. Poland*
[3]*Smell and Taste Clinic, Departament of Otorhinoloaryngology, Techniche Universitat Dresden. Germany*

People differ in their ability to pick up odors from the environment consciously, their awareness of ambient odors, and the role scents play in their lives, all reflected in a metacognitive ability called odor awareness. These differences can be observed rather early, in infants and children, e.g. girls report higher attention and reactivity to odors in daily settings than boys. Yet, research on odor awareness in children, particularly regarding metacognitive olfactory abilities, is limited. The existing methods display good psychometric characteristic, however they require advanced verbal and cognitive abilities, and abstract thinking. High cognitive demand makes available odor awareness measures difficult to use in children. We aimed to validate Odor Awareness Scale in Children (OAS-C) – an open-ended task where children spontaneously list odors they know from daily life. In the pilot study, 34 children aged 6 years (21 girls) were interviewed with our new tool and Children's Olfactory Behavior in Everyday Life (COBEL) questionnaire, to measure convergent validity. Children's olfactory performance was measured with an odor identification U-Sniff test. We also measured food neophobia to assess divergent validity, and verbal fluency, since they can affect odor identification skills and could potentially influence the ability to answer the odor awareness-oriented questions. In the overall sample, our tool trended a positive correlation with COBEL ($r=0.31$, $p=0.095$), and significantly correlated with verbal fluency ($r=0.42$, $p=0.020$). As expected, neither OAS-C nor COBEL correlated with food neophobia ($p>.220$). The new method might serve as a quick and useful tool for measuring odor awareness in preschool children. Upon more extensive validation, OAS-C can provide useful insights into children's olfactory environment and shed light on how children learn about ambient odors.

**Funding:** This work was supported by University of Wroclaw, with an IDUB program grant awarded to MR.

**abstract citation id: bjaf055.200**

### Study of olfactory behavior in Drosophila adults using optogenetic tools

*Fernando Martín López*[1], *Ruth Coya*[1], *Laura Calvin-Cejudo*[1], *Carolina Gomez-Diaz*[1], *Esther Alcorta*[1]

[1]*Departamento de Biología Funcional (Genética). Facultad de Medicina. Universidad de Oviedo. Calle Julian Clavería, 6. 33006. Oviedo, Spain*

Optogenetics allows the alteration of cell activity using genetically targeted expression of light activated receptors as channelrhodopsin or halorhodopsin. Thus, by expressing these receptors in different cells of the behavioral circuit, it is possible to evaluate their effect on the final behavior.

With this approach the native behavioral stimulus is replaced by the light-induced electrical activation of different points of the circuit and its effects on subsequent steps of the circuit or on the final behavior can be analyzed.

In this work we first explore a dual excitation model where the subject is responding to odors while the 70% of the OSNs are optogenetically activated using an Orco-Gal4 insert that drive the expression of a UAS-channelrhodopsins insert. Thereby, we can assess if and how the olfactory behavior is modified. We measure the effects of light excitation on the response to several odorant concentrations in a T-maze paradigm. The dose-response curve of these flies still depends on odor concentration, but with reduced sensitivity compared to olfactory stimulation alone. These results are consistent with the behavioral tests performed with a background odor and suggest an additive effect of light and odor excitation on OSNs.

We also use this optogenetic tools to alter the activity of a type of antennal perineural glial cells, and we observe changes in the olfactory perception, this suggest that there is interaction of these glial cells with the olfactory receptor neurons.

**Funding:** This work was supported by the Spanish Ministry of Science, Innovation and University [MCINN-24-PID2023-149482NB-I00], Spanish Ministry of Economy, Industry and Competitiveness [SAF2013-48759-P and BFU2017-85882-P], the University of Oviedo [PAPI-17-PEMERG-2 and PAPI-GR-2016-0012], and the Principality of Asturias Government [IDI-2018-000182]. R.C., A.C.S. and L.C.C. were funded by Severo Ochoa PhD fellowships from the Principality of Asturias Government.

**abstract citation id: bjaf055.201**

### The sickness perfume: validation of a percept-first body odor augmentation

*Tim L. Jesgarzewsky[1], Michał Pieniak[2], Ilona Croy[1]*

*[1]Department for Clinical Psychology, Friedrich Schiller University Jena. Germany*
*[2]Institute of Psychology, University of Wroclaw, Poland*

Body odor conveys socially relevant information about health, stress, and kinship. However, its inherent complexity presents significant challenges for experimental investigation. In two studies (N=40 each) we tested if adding a single monomolecular odorant to natural body odor could induce a targeted perceptual shift, specifically the scent of sickness, while maintaining perceived naturalness.

Sickness manipulation was created by augmenting axillary body odor samples with p-Tolyl Acetate, an odorant which is described with labels that are also used for sickness-related body odors.

In Study 1, participants rated the augmented body odor as significantly more sick than the natural odor ($z = 2.95$, $p = .006$), without being rated as more artificial ($z = 1.92$, $p = .55$) or intense ($z = -1.59$, $p = .11$). A triangle test confirmed participants perceived the augmented odor as a variant of natural body odor, distinct from the pure odorant. In Study 2, we compared three different augmentations of natural body odor: 4-Decanolide (supposed to enhance the health percept), Eucalyptol (neutral), and p-Tolyl Acetate (sick). The 'sick' manipulation was rated as significantly more sick and less approachable than the healthy (sick: $z = 4.63$, $p < .001$; approach: $z = -4.48$, $p < .001$) and neutral manipulations (sick: $z = 5.60$, $p < .001$; approach: $z = -2.84$, $p = .014$). Further, the 'sick' manipulation elicited stronger disgust-related muscle activity (m. levator labii superioris) than the healthy manipulation ($z = 3.37$, $p = .003$), and shallower breathing inhalation compared to the natural body odor ($z = -2.82$, $p = .024$).

These findings reveal a simple, effective method for creating targeted body odor percepts. This technique provides a new tool for systematically investigating the nuanced role of body odor in social communication.

**Funding:** The study was funded by the "Volkswagen Foundation" as part of the research project "Olfactorial Perceptronics".

**abstract citation id: bjaf055.202**

### Neural and behavioral discrimination of bitter taste in rats

*Oren Mazon[1], Anan Moran[1]*

*[1]Tel Aviv University. Israel*

Bitterness is often considered a single, uniform taste quality. However, emerging evidence suggests that under the right conditions, animals and humans can distinguish between different bitter compounds, even when their intensity is factored out. In this study, we investigated the behavioral and neural discrimination of bitter tastes in rats (Long-Evans), to test whether bitterants can be discriminated rather than perceived as one general sensation. First, we calibrated iso-intense concentrations for several bitterants so that each compound was matched for equal perceived intensity. Using these balanced stimuli, we trained rats in a taste discrimination task while increasing tasting time. A validation test with one rat, discriminating quinine from potassium chloride (KCl), resulted in over 80% correct choices. This high performance confirmed the reliability of our behavioral paradigm for assessing taste discrimination. Next, we initiated Neuropixels recordings of neural activity in the gustatory cortex (GC) while delivering three bitterants – propylthiouracil (PROP), quinine, and denatonium benzoate – each at two iso-intense concentrations. Analysis of these neural data is currently underway and will focus on identifying patterns of activity in the gustatory cortex in response to different bitterants. These data will be used to examine the neural encoding of bitter stimuli and its relationship to behavioral discrimination performance.

**Funding:** Israel Science Foundation abstract citation id: bjaf055.203

### Sensory responses of olfactory and vomeronasal neurons in does are influenced by buck odors and their reproductive status

*Helene Vacher[1], Maxime Meunier[2], Elliott Trives[2], Emma Gerardin[1], Chantal Porte[1], Adrien Acquistapace[1], Philippe Chemineau[1], Pablo Chamero[1], Matthieu Keller[1]*

*[1]UMR Physiologie de la Reproduction et des Comportements, INRAE, CNRS, Université de Tours, Nouzilly. France*
*[2]Neuroendocrinology lab, GIGA Neurosciences, University of Liège. Belgium*

The "male effect" refers to the phenomenon where the introduction of a sexually active male (buck) stimulates ovarian activity and induces estrus in anovulatory females (does) after seasonal anestrus. This effect is mediated by sensory cues, especially chemicals from buck hair, which act via the female's olfactory system to trigger neuroendocrine changes, leading to increased secretion of gonadotropin-releasing hormone and luteinizing hormone surges. However, the specific chemicals in buck hair and the exact sensory pathways used by females remain elusive. Here, we investigated whether sensory cells from the main olfactory epithelium (MOE) and vomeronasal organ (VNO) of does respond differently to olfactory stimuli from sexually active bucks (SAB) and wethers (CAS, castrated males), and how this response is influenced by female reproductive status (breeding season, anestrous, or ovariectomized (OVX)). To do so, freshly dissociated MOE and VNO cells from does in the three states were exposed to chloroform/methanol and aqueous extracts from SAB and CAS buck hair. Calcium imaging was used to assess neuronal activation. Our results showed that across all extraction methods, more sensory neurons were activated by SAB hair extracts than by CAS extracts. Notably, MOE cells from anestrous does showed higher activation to SAB extracts compared to cells from breeding season or OVX does. The majority of responsive cells were identified as non-mature olfactory neurons. Our findings indicate that female goats can discriminate olfactory cues from sexually active and castrated males via both MOE and VNO pathways. The increased MOE responsiveness during anestrus suggests an enhanced role of the MOE in mediating the "male effect" when females are not in their regular breeding season.
**Funding:** This work was supported by a grant from Région Centre Val de Loire (France) and the INRAE PHASE Department (France).

abstract citation id: bjaf055.204

### Role of prolactin on the reconfiguration of the accessory olfactory bulb mitral cell electrical activity and behavioral responses in females

*Benito Ordaz Sánchez[1], Verónica Viñuela Berni[1], Fernando Peña Ortega[1], Teresa Morales[1], Rebeca Corona[1]*

*[1]Instituto de Neurobiología, Universidad Nacional Autónoma de México. Mexico*

Reproduction in mammals is highly driven by odors processed by the accessory olfactory bulb (AOB). Prolactin (PRL) participates in reproduction and its receptors are expressed in the mitral cell layer (MCL) of the olfactory bulb. Acute injection of PRL alters AOB activation and augments the exploration of male stimuli. In the current project, we assessed the participation of PRL on reproductive behaviors and the AOB physiological responses by PRL. Adult CD1 female mice received PRL for 10 days (5mg/kg) to generate hyperprolactinemia (hPRL) before subjected to olfactory preference and sexual behavioral tests; slices to record the basal electrical activity of the MCL-AOB and in presence of PRL were obtained from behavior-hPRL group, behavior control group, lactating group (physiological hPRL), estrous and diestrus group. Results show that PRL promotes behavioral alterations: increased olfactory preference for sociosexual stimuli and altered sexual interaction, with more rejections towards the male and less stimulation received. PRL administered during recording did not modify the total power of the MCL-AOB of any of the groups. For the total potency of the MCL basal activity, behavior group showed higher activity but behavior-hPRL and estrous groups showed significantly lower activity, compared to the other groups. Our results show that PRL during the recording does not alter the electrical activity of the MCL, the basal activity of the MCL-AOB is lower in the estrus compared to diestrus and lactation, and reproductive behavior increases the electrical activity of MCL, however, a period of hPRL prior to the behavior prevents this increase. These results suggest that PRL might have a long-term effect on the electrical activity of the AOB, since a hPRL period alters the behavioral responses of the females and prevents the increase in MCL-AOB activity generated by behavioral training, suggesting that PRL might reconfigures the AOB circuits.
**Funding:** Financial support by UNAM-DGAPA-PAPIIT IN214822 and IN220025.

abstract citation id: bjaf055.205

### Identification of emotion-influencing aroma components in Sichuan pepper based on psychophysical and psychophysiological methods

*Zhiyang Chen[1], Xiaobo Zou[2], Lei Zhao[3], Patricia Viret[1], Tao Jiang[1]*

*[1]Center for Research in Neuroscience in Lyon. France*
*[2]School of Food and Bioengineering, Jiangsu University. China*
*[3]China National Institute of Standardization, Beijing. China*

Sichuan peppers (*Zanthoxylum spp.*) are a traditional Chinese condiment that has gained prominence in Asian cuisines.

Rich in volatile and bioactive compounds, Sichuan peppers possess a distinct flavor and are hypothesized to affect human emotions via olfactory and trigeminal pathways. A comprehensive investigation into the mechanisms by which Sichuan pepper aroma influences emotions may offer valuable insights for advancing flavor science, nutritional strategies, and mental health research. Numerous studies have identified several chemical components that may be associated with emotional responses to Sichuan pepper, but our knowledge of the perceptual and emotional responses these components is still very limited. To achieve this goal, we constructed 40 odor stimuli containing the 9 key volatile compounds from Sichuan pepper in single and mixed systems and delivered these stimuli using an olfactometer. The perception and emotions evoked by these olfactory stimuli were evaluated with response on the scales displayed on screen and multi-channel physiological monitoring (including electroencephalogram (EEG), electrocardiogram (ECG), electrodermal activity (EDA) and respiratory rate (RR)). Preliminary data analysis allowed us to identify at least 4 key compounds associated respectively with the positive or negative emotional responses to Sichuan pepper. The study also found that the same compound triggered different emotional responses in different carriers. We will further analyze the effects of each key compound in a single or mixed system to explore the laws and mechanisms by which they stimulate emotional responses. It is expected that our findings can provide theoretical guidance and practical applications for optimizing food formulations and improving emotional valence while maintaining the flavor characteristics of Sichuan pepper in foods. This study received support from public financial sources.

**abstract citation id: bjaf055.206**

### Learning tunes olfactory sensitivity

*Shreya Choudhuri*[1], *Mark Conway*[2], *Zongqian Zhang*[1], *Jamie Johnston*[1]

[1]*University of Leeds. United Kingdom*

Olfactory learning enables animals to form essential associations between odours and biologically meaningful outcomes, such as locating food or avoiding predators. We are investigating how learning modifies the olfactory circuitry to support these diverse behavioural responses. To do this, we developed a behavioural paradigm that allows us to measure innate odour sensitivity in naïve mice. Our findings reveal that this innate sensitivity can vary by several orders of magnitude.

We then measured neural sensitivity using in vivo multiphoton $Ca^{2+}$ imaging of odour-evoked activity at the input to the olfactory bulb. We show that appetitive learning induces a large shift in the neural input to the olfactory bulb. Importantly, the tuning curve of the olfactory input matches the concentration gradient that was presented during the learning paradigm. Preliminary data further indicate that this tuning curve adapts to different concentration gradients used in appetitive learning, while fear conditioning results in an increased gain at lower concentrations.

These results suggest that the behavioural demands of a task can evoke distinct context-dependent changes in odour sensitivity at the level of olfactory input to the brain.

**Funding:** This research has been generously supported by the BBSRC White Rose DTP.

**abstract citation id: bjaf055.207**

### Olfactory symptoms in patients of the Post Covid Center Erlangen

*Moritz Engelhardt*[1], *Eva Morawa*[2], *Yesim Erim*[3], *Freiherr Jessica*[4]

[1]*Department of Psychiatry and Psychotherapy, Friedrich-Alexander-Universität Erlangen-Nürnberg, 91054 Erlangen, Germany*
[2]*Department of Psychosomatic Medicine and Psychotherapy, University Hospital of Erlangen, Friedrich-Alexander University Erlangen-Nürnberg (FAU), 91054 Erlangen, Germany*
[3]*Department of Psychosomatic Medicine and Psychotherapy, University Hospital of Erlangen, Friedrich-Alexander University Erlangen-Nürnberg. Post-COVID Center, University Hospital Erlangen, 91054 Erlangen, Germany*
[4]*Department of Psychiatry and Psychotherapy, Friedrich-Alexander-Universität Erlangen-Nürnberg, 91054 Erlangen, Germany. Sensory Analytics and Technology, Fraunhofer Institute for Process Engineering and Packaging. Germany*

Years after the Sars-CoV2 pandemic, millions of people still suffer from long term symptoms of their infection, summarized as post-acute CoViD-19 syndrome (PACS). PACS comes in many forms and with a diverse symptom spectrum, including fatigue, cognitive impairments, and olfactory dysfunction. In this interdisciplinary project, we conducted olfactory testing on patients of the Post Covid Center Erlangen between March 2024 and May 2025. 142 patients with confirmed PACS diagnosis as established by specialists were included in our study. We assessed olfactory function in each patient using the Sniffin‘ Sticks TDI test and self-assessment questionnaires. These findings were put in context with a wide range of psychophysiological tests and scales as well as sociodemographic data. The standardized TDI test resulted in 43.66% of patients showing no signs of olfactory deficits, while 53.52% of patients reported subjectively lower olfactory function compared to before their infection. For patients that rated their olfactory function as 5 out of 10 or lower, the self- assessment correlated significantly with the TDI score. On average, olfactory scores were in the 31st percentile compared to age/

gender-matched normative data. 30.99% of patients reported qualitative olfactory dysfunction. The hedonic values of 3 odors of the identification test were perceived significantly different between hyposmic and normosmic patients. A lower percentile rank for olfactory function was associated with more severe signs of insomnia and the fatigue subitem of the patient health questionnaire, overall more severe symptoms in the burden of somatic symptoms, and worse scores in the cognitive subtests for verbal fluency and working memory. Overall, we offer a comprehensive and detailed characterization of olfactory symptoms and their relationship to other manifestations within the PACS spectrum in a patient cohort.

**abstract citation id: bjaf055.208**

### Olfactory dysfunction in a mouse model of kidney disease

*Verónica Viñuela Berni[1], Joana Hernández Lara[1], María Antonieta Carbajo Mata[1], Teresa Morales[1], Rebeca Corona[1]*

*[1]Instituto de Neurobiología, Universidad Nacional Autónoma de México. Mexico*

Mexico has a high prevalence of kidney disease (KD). KD is characterized by functional and structural abnormalities of the kidneys. KD patients report olfactory alterations that affect their quality of life and can lead to unbalanced feeding behavior, altering metabolism. The onset and evolution of the pathophysiology of the olfactory alterations have been little addressed. In the present study, a KD model was generated in adult C57BL/6J female mice by oral administration of adenine (tx; 50 mg/kg, n=10). To assess olfactory alterations in this model, we employed four behavioral tests: the olfactory threshold test (OTT), the habituation/dishabituation test (HDT), the olfactory food preference test (OFPT), and the buried food test (BFT). These tests evaluated the mice's ability to detect (sensitivity-OTT and locate food-BFT using olfactory cues), differentiate odors (memory and discrimination processes-HDT), and their preference (OFPT). The tests were performed before the tx and 4, 6 and 8 weeks after adenine-tx. Before adenine-tx, all female mice showed intact olfactory capacity. Four weeks after adenine-tx, mice displayed decreased odor sensitivity-OTT, showing reduced sniffing time and decreased HDT performance over time, becoming more pronounced at 8 weeks after adenine-tx. Regarding BFT, in week 8 after adenine-tx, mice displayed a decrease in the latency for uncovering the buried food. Interestingly, at 4 and 6 weeks after adenine treatment, KD mice showed an increase in time spent eating the buried food. In OFPT, neither group showed any olfactory food preference at 4, 6, and 8 weeks after adenine-tx. However, we observed that the percentage of the total exploration time to each food stimulus decreased in KD mice. Our data indicate that adenine-tx disrupts olfactory function in mice modulating the detection processes and altering memory and discrimination processes. Further experiments are needed to unravel the olfactory dysfunction in the KD mouse model.

**Funding:** Supported by UNAM-DGAPA-PAPIIT IN214822, IN220025 and IN205423.

**abstract citation id: bjaf055.209**

### Ongoing Neuronal Activity in the Gustatory Cortex Actively Contributes to the Consolidation of Taste Memory

*Tasabeeh Ghazawi[1], Anan Moran[1]*

*[1]Tel Aviv University. Israel*

Long-term memory consolidation has traditionally been attributed to autonomous molecular cascades. However, whether ongoing neuronal activity plays an active, time-dependent role in driving this process remains unresolved. Using conditioned taste aversion (CTA) in rats, we are investigating the potential causal contribution of post-learning activity in the gustatory cortex (GC) a key site for taste learning. To this end, we employ long-term optogenetic silencing (using AAV containing eOPN3 channel) of GC pyramidal neurons during discrete two-hour post-training windows (0–2 h, 2–4 h, 4–6 h, and 6–8 h), using chronically implanted fiber optics for light delivery. CTA retention is quantified via sucrose consumption and aversion index. We hypothesize that silencing GC activity during a critical 4–6h post-training window (previously described as a CTA consolidation phase) will impair memory retention, whereas silencing during earlier or later periods will have minimal effects. If confirmed, this would suggest that GC activity during a specific consolidation window is necessary for memory stabilization. Such findings would challenge purely molecular models of memory and support a framework in which consolidation depends on temporally structured neuronal activity. This work could refine our understanding of systems-level plasticity and inform future strategies for targeting memory-related disorders.

**abstract citation id: bjaf055.210**

### Dose-response relationship of sugar-sweetened beverages with and without non-nutritive sweetener sucralose and diet-induced thermogenesis

*Dilan Deniz Yilmaz[1], Marga Geraldine Veldhuizen[1], Zeynep Altinkaya[2], Uğur Dal[1]*

*[1]Department of Physiology. Faculty of Medicine, Mersin University, Turkey*
*[2]Department of Medical Services and Techniques, Karamanoğlu Mehmetbey University. Turkey*

There is a strong association between sugar-sweetened beverage intake and weight gain, obesity and metabolic syndrome. Sucrose and sucralose are often used in combination in sweetened products and can be encountered in many "diet" products, sports products and regular products. However, very little is known about the interactions of nutritive and non- nutritive sweeteners on metabolism. When sweetness and calories are mismatched, particularly when the drink is "far too sweet" for its calories (from maltodextrin), a decreased diet-induced thermogenesis (DIT) response is observed relative to matched beverages. One difficulty in interpreting these observations is that there is no benchmark for a dose-response DIT relationship for sugar-sweetened beverages. Here we investigate the effect of calories in and sweetness of beverages on metabolic response to sucrose across a range of nutrient doses. In a mixed design, we used 6 doses of sucrose (from 0 to 150 kcal in 30 kcal steps, adjusted for individual BMR) as a within-subjects independent variable, and as a between-subjects variable we added no extra sucralose ($n = 4$) or a constant dose of sucralose equi-sweet to 75 kcal sucrose ($n=4$).

Unexpectedly we observed that drinks that are "too sweet" lead to a higher DIT ($F(5,30) = 7.636$, $p<.001$) relative to sucrose on its own, particularly in the range of 90-120 kcal. We conclude that mismatches between sweetness and calories lead to aberrant metabolic responses. Future studies may further investigate the influence of combining non-nutritive sweeteners with different types of nutritive sweeteners, such as sucrose vs maltodextrin.

**Funding:** The study was supported by the TUBITAK 2224a project

**abstract citation id: bjaf055.211**

### Tracking neuronal activity and connectivity across CTA learning and extinction

*Mai Shafiki Shafiki*[1], *Anan Moran*[1]

[1]*Tel Aviv University. Israel*

Conditioned taste aversion (CTA) is a powerful form of associative learning in which a single pairing of a palatable taste with visceral malaise leads to long-lasting avoidance. While its behavioral expression is well characterized, much less is known about how neuronal activity and connectivity change throughout the learning process. Most studies focus on isolated timepoints before and after conditioning, leaving the dynamic neural transformations that occur during memory formation and extinction poorly understood.

To address this, we recorded gustatory cortex (GC) activity using Neuropixels across three days of CTA: baseline, acquisition, extinction test, and final test. We tracked how GC responses to the conditioned stimulus (sucrose) evolve over time, analyzing single-neuron coding, ensemble dynamics, and shifts in functional connectivity. Preliminary findings suggest that changes in activity unfold in discrete stages and align with the behavioral transition from aversion acquisition to extinction.

We also examined how responses to non-conditioned tastes change across days, testing whether GC activity reflects the generalization and eventual specificity of aversion. Using facial video analysis alongside neural recordings, we relate behavioral taste reactivity to evolving neural population states.

These data offer a high-resolution, time-resolved view of how sensory cortical representations and connectivity reorganize across learning and extinction, providing new insights into the temporal dynamics of memory formation in cortex.

**Funding:** This work was supported by the Israel Science Foundation (Grant No. 2895/20, Anan Moran).

**abstract citation id: bjaf055.212**

### Evoking olfactory imagery through glass and multisensory artistic stimuli

*Lena Trost*[1]

[1]*Bauhaus-University Weimar. Germany*

Olfactory imagery, the ability to perceive smells without physical stimuli, is subjective and challenging. Given the individual and biographical nature of olfactory memory, this project explored whether multisensory artistic stimuli and odorless glass as a projection surface facilitate imagined olfactory experiences. To investigate this, a series of *Glass Tastings* was developed combining guided narration, non-figurative drawings (texture, color, shape), touch, sound, and crafted glass objects to focus attention and activate sensorimotor processes. Conducted with 291 participants in in-person and digital formats (including browser-based solo experiences and Zoom workshops), 151 data sets were qualitatively analyzed using interviews and written reflections. Despite methodological differences, consistencies emerged: participants experienced olfactory imagery in highly individual ways, with tendencies for certain stimuli—e.g., touching wood often evoked episodic odor recall. Texture drawings stimulated trigeminal and temperature-related sensations, while pitch-based sounds modulated perceived temperature of imagined smells, suggesting sound-temperature-odor correspondences. Furthermore, hunger states influenced imagery content, with hungry participants reporting more food-related odor imagery. Notably, participants with olfactory dysfunction ($n=7$) also reported olfactory imagery, identifying glass as key to their experience. Findings highlight the potential of a multisensory artistic approach using glass to evoke olfactory imagery, aligning with grounded cognition theory that embodied, crossmodal experience activates olfactory imagination. Glass, as an odorless medium, effectively focused attention and enabled vivid imagery.

**Funding:** This project is supported by Alexander-Tutsek Foundation, Women's Promotion Fund of Bauhaus Research School, and Graduate Center Koblenz University of Applied Sciences.

**abstract citation id: bjaf055.213**

### Modulation of the attentional system by trigeminal olfactory stimuli

*Cédric Manesse[1], Claire-Sophie Vigneron[1], Jonathan Warr[1]*

*[1]Takasago Europe Perfumery Laboratory. France*

Attention processing involves the thalamus. If the olfactory pathway bypasses it and projects directly to cortical areas, this is not the case when odor activates the trigeminal pathway. Here, we studied how attention is modulated when odors include a trigeminal component, through both olfactory-driven and cross-modal attention tasks.

In the first phase, we explored the impact on sustained attention to odors, focusing on olfactory habituation. 35 participants rated intensity and liking of repeated odor presentations (20 times). Results showed that trigeminal odor (containing “fresh” menthol derivative compound) showed reduced habituation, sustaining intensity ratings, unlike the control odor which declined over time ($t(46) = -2.16$, $p = 0.0359$). These results suggest that trigeminal input helps maintain attentional resources toward olfactory stimuli, reducing the natural decline in salience over repeated exposure.

In the second phase, 40 participants (20 per group) were asked to perform an auditory oddball task while wearing a 14- electrode EEG headset, twice. In the first time, no odor was used (baseline), then during the second time, they were exposed to one of two versions of a fragrance: one “simple” odor and one enriched with a trigeminal component. Participants exposed to the simple odor reported the second task felt more “mentally demanding”, “difficult” and “harder to follow”. In trigeminal odor group, a significant increase in auditory P3 amplitude was observed during the second repetition (t-test, $p = 0.021$, at F3), suggesting that trigeminal odor exposure helped maintain attentional engagement even for non-olfactory stimuli.

These findings support the idea that trigeminal activation during olfactory stimulation engages thalamic circuits and modulates attentional resource allocation. Altogether, these results offer perspectives for further investigations into the processing of trigeminal chemosignals and attention circuitry.

**abstract citation id: bjaf055.214**

### Can olfactory awareness increase after exposure to odors?

*Anna Oleszkiewicz[1,2], Daniel Marek[1]*

*[1]Institute of Psychology, University of Wroclaw, Wroclaw 50-527, Poland*
*[2]Department of Otorhinolaryngology, Faculty of Medicine Carl Gustav Carus, Smell & Taste Clinic, Technische Universität Dresden, Dresden, Germany*

People differ in terms of olfactory abilities and awareness of ambient odors. Olfactory training (OT), mainly used for smell rehabilitation, has shown positive effects on olfactory and cognitive abilities. This study tested whether standardized OT enhances odor awareness, the significance of olfaction, odor identification and verbal fluency in children aged 6-9 years. A total of 101 children (52 girls) participated in a 12-week OT. The experimental group (57 children) smelled four odors (rose, eucalyptus, lemon, cloves) twice daily, while the placebo group (44 children) received odorless OT. Results showed a slight increase in odor awareness and significance of olfaction, especially in the experimental group and among children with lower baseline odor awareness. OT did not affect odor identification or verbal fluency. In conclusion, OT may improve metacognitive aspects of olfaction in children with less odor experience. Further research is needed to clarify its effects on olfactory development.

**Funding:** This study was supported by a National Science Centre (Poland) research grant #2020/39/B/HS6/01533 awarded to AO.

**abstract citation id: bjaf055.215**

### Prevalence of chemosensory dysfunction among US adults with and without a history of cancer in NHANES 2013-2014

*Vicente Ramirez[1], Lakmani Tharaka Galaniha[2], Victoria Esparza[1], Kara Stromberg[3], Alissa Nolden[2], Valentina Parma[1]*

*[1]Monell Chemical Senses Center, Philadelphia, PA. United States*
*[2]University of Massachusetts, Amherst, MA. United States*
*[3]Fox Chase Cancer Center, Philadephia, PA. United States*

Chemosensory dysfunction is a recognized quality-of-life burden and safety risk for adults, particularly cancer survivors. We conducted a cross-sectional analysis of National Health and Nutrition Examination Survey (NHANES) data from 2013–2014 to determine the prevalence of chemosensory dysfunction. Adults ≥40 years (n=3,090) completed questionnaires assessing cancer history, self-reported problems with smell and taste in the past 12 months, changes in perception since age 25, and phantom odors. Olfactory and gustatory function were psychophysically evaluated using the 8-item Pocket Smell Test (dysfunction= ≤6 correct identifications) and bitter identification with a 1 mM quinine solution. Weighted prevalence estimates were calculated using survey design-adjusted methods. Survey-weighted quasibinomial generalized linear models were used to assess the effects of demographic variables. Among 3,090 participants, 418 reported a history of cancer. It is estimated that 27.0% of those with cancer (CI:21.4–32.6%) reported smell problems in the past year, compared to 20.7% (CI:17.5–23.0%) in those without cancer ($p<0.014$). For taste, it is estimated that 11.8% (CI:7.0–16.7%) of those with cancer reported dysfunction, compared to 9.5% (CI:7.6–11.5%) without cancer ($p=0.56$). Psychophysical testing estimated olfactory dysfunction in 19.7% (CI:14.2–25.2%) of those with cancer, significantly higher than the 10.7% (CI:8.5–12.9%) prevalence in those without cancer (p<0.0001). Quinine-based testing found taste dysfunction in 13.6% (CI:6.9–20.3%) of those with cancer and 17.9% (CI:15.9–20.0%) without cancer (p=0.2). Demographics were not significantly associated with smell or taste dysfunction in those with cancer. Adults with a history of cancer have a higher prevalence of both subjective and psychophysically measured chemosensory dysfunction, highlighting the need to include chemosensation assessment in survivorship care.

**Funding:** This work was supported by T32DC000014.

### References

1 Centers for Disease Control and Prevention (CDC). (2015). National Health and Nutrition Examination Survey: 2013–2014 Data Documentation, Codebook, and Frequencies. U.S. Department of Health and Human Services, Centers for Disease Control and Prevention. https://wwwn.cdc.gov/nchs/nhanes/continuousnhanes/default.aspx?BeginYear=2013

2 Nolden AA, Hwang L-D, Blotong A, Reed DR. Chemosensory Changes from Cancer Treatment and Their Effects on Patients' Food Behavior: A Scoping Review. Nutrients.2019;11(10):2285. https://doi.org/10.3390/nu11102285

**abstract citation id: bjaf055.216**

### Multivariate pattern analysis in the human brain reveals distributed representations of odors beyond traditional olfactory regions

*Alice Stephan[1], Jens V. Schwarzbach[1]*

*[1]University of Regensburg. Germany*

Neuroimaging studies on the human sense of smell have primarily highlighted activity in the primary and secondary olfactory cortices. However, little is known about how odors are processed in more downstream or widespread brain regions. We argue that this limited understanding is partly due to the predominant use of traditional mass-univariate fMRI analyses, which examine each voxel independently. Here, we aimed to identify distributed activation patterns across the whole brain involved in odor discrimination.

In our human fMRI study, we presented ten different odorants to 30 participants using an MRI-compatible olfactometer while recording blood-oxygen-level dependent (BOLD) signals. We then estimated whole-brain activation patterns for each participant. To identify where in the brain odor identity could be distinguished, we conducted a multivariate whole-brain searchlight analysis. In this approach, a small spherical cluster (the "searchlight") is systematically moved through the entire brain volume, and within each local neighborhood, we applied 10-fold cross-validation combined with linear discriminant analysis to test for odor classification.

The resulting group-level accuracy maps revealed odor discrimination significantly above chance level not only in expected regions such as the piriform cortex, anterior olfactory nuclei, and amygdala, but also in further downstream areas including the parietal cortex and precuneus.

Our findings demonstrate that multivariate pattern-based analyses provide a powerful means to investigate, across the whole brain, whether specific regions can differentiate between individual odors or odor categories. Such approaches hold great promise for uncovering how chemical differences between odorants are ultimately transformed into distinct neural representations and perceptual experiences.

**Funding:** Deutsche Forschungsgemeinschaft (DFG) GRK2174, University of Regensburg.

**abstract citation id: bjaf055.217**

### Do anxiety chemosignals influence our smell sensitivity for common odors? Research utilizing dental phobia participants

*Annkatrin Wunder[1], Nele Bürkel[1], Imke Guder[1], Jose Zorzin[1], Christiane Mühle[1], Helene Loos[1], Jessica Freiherr[1]*

*[1]FAU Erlangen-Nürnberg. Germany*

Chemocommunication plays a crucial role in human nonverbal communication. While the behavioral effects of anxiety- related chemosignals on recipients are well-documented, it remains unclear whether such stimuli can modulate sensitivity for common odors. This study aimed to investigate whether exposure to anxiety chemosignals influences olfactory sensitivity.

We assessed the odor sensitivity of 36 participants to phenylethyl alcohol (PEA) and n-butanol following exposure to anxiety and neutral chemosignals (generated by dental phobia participants), and a blank condition. Additionally, a fourth condition involved viewing a horror movie to compare the effects of anxiety induced by visual stimuli with those of chemosignals.

Results revealed a significant increase in sensitivity to PEA following exposure to anxiety chemosignals compared to the blank condition, whereas no significant change was observed for n-butanol.

To further analyze the chemical composition of the applied chemosensory stimuli, we employed untargeted one-dimensional gas chromatography-mass spectrometry (GC-MS) and gas chromatography-olfactometry (GC-O). While GC-MS data did not identify compounds with significantly higher normalized peak areas in anxiety sweat samples compared to neutral sweat samples, GC-O analysis suggested that dodecanoic acid and 3-hydroxy-3-methylhexanoic acid were key contributors to the odor profile of anxiety sweat. In contrast, tetradecanoic acid and patchouli alcohol were prominent in neutral sweat samples. These findings provide evidence that anxiety chemosignals enhance sensitivity to common odors, offering new insights into the role of emotional states in olfactory perception. Moreover, this study contributes to a deeper understanding of the chemical composition of sweat involved in human chemocommunication.

**Funding:** The study was funded through appointment funds from Prof. Dr. Jessica Freiherr.

**abstract citation id: bjaf055.218**

### Bitter peptides of pea protein hydrolysates induce gastric signals of satiation and reduce healthy subjects' total energy intake

*Katrin Gradl[1], Sonja Sterneder[1], Phil Richter[1], Kristin Kahlenberg[1], Beate Brandl[2], Thomas Skurk[2], Veronika Somoza[3]*

*[1]Leibniz Institute for Food Systems Biology at the Technical University of Munich. Germany*
*[2]Technical University of Munich. Germany*
*[3]University of Vienna. Austria*

With the shift towards plant-based diets, the bitterness of plant-based proteins, isolates, and hydrolysates has become an important consideration, as these sources are particularly prone to developing bitter flavors. Meanwhile, the satiating effects of proteins and bitter-tasting food constituents have been demonstrated. Building on our previous findings that non-bitter proteins can be cleaved into bitter peptides during gastric digestion[1,2], we hypothesized that bitter peptides from plant protein hydrolysates might elicit gastric satiation signals.

In vitro, gastric digests of a more bitter-tasting (MBT) and a less bitter-tasting (LBT) pea protein hydrolysate (PPH) both stimulated proton secretion (PS) and serotonin release in parietal HGT-1 cells, implicating bitter taste receptors (TAS2Rs). A sensory-guided LC-MS approach identified six bitter peptides from MBT-PPH or LBT-PPH. TAS2R4 and TAS2R43 involvement was confirmed via CRISPR-Cas9 knockout. All peptides equally stimulated PS, while the three LBT-PPH-derived peptides more potently triggered serotonin release over a concentration range of 0.005 to 50 µg/ml.[3]

Given the lower yield of bitter peptides in LBT-PPH, we tested the satiating effects of both hydrolysates in a randomized, single-blinded, controlled proof-of-concept human study. Nineteen moderately overweight male subjects consumed 300 ml boluses of a 75 g glucose solution (control) or solutions containing 15 g LBT-PPH or MBT-PPH. MBT-PPH administration delayed gastric emptying by 65% ($p < 0.0001$) and reduced energy intake from a standardized breakfast by 126 ± 329 kcal ($p < 0.05$) compared to control. In contrast, LBT-PPH decreased plasma ghrelin levels, indicating a satiating potential, although total energy intake remained unchanged.

These findings highlight the promise of PPHs as functional ingredients in weight management, with bitter peptides — whether formed during digestion or already present — playing a key role in promoting satiation.

### References

1 Richter, P., Sebald, K., Fischer, K., Behrens, M., Schnieke, A., & Somoza, V. (2022). Bitter peptides YFYPEL, VAPFPEVF, and YQEPVLGPVRGPFPIIV, released during gastric digestion of casein, stimulate mechanisms of gastric acid secretion via bitter taste receptors TAS2R16 and TAS2R38. Journal of Agricultural and Food Chemistry (Vol. 70, Issue 37, pp. 11591–11602). American Chemical Society (ACS). https://doi.org/10.1021/acs.jafc.2c05228

2 Richter, P., Sebald, K., Fischer, K., Schnieke, A., Jlilati, M., Mittermeier-Klessinger, V., & Somoza, V. (2024). Gastric digestion of the sweet-tasting plant protein thaumatin releases bitter peptides that reduce H. pylori induced pro-inflammatory IL-17A release via the TAS2R16 bitter taste receptor. Food Chemistry (Vol. 448, p. 139157). Elsevier BV. https://doi.org/10.1016/j.foodchem.2024.139157

3 Gradl, K., Richter, P., Somoza, V. (2025) Bitter peptides formed during in-vitro gastric digestion induce mechanisms of gastric acid secretion and release satiating serotonin via bitter taste receptors TAS2R4 and TAS2R43 in human parietal cells in culture. Food Chemistry (Vol 482, p. 144174). Elsevier BV. doi: 10.1016/j.foodchem.2025.144174.

**abstract citation id: bjaf055.219**

### The effect of olfactory and auditory contexts on associative memory in an fMRI study

*Javier Albayay[1], Elena Nicolau[1], Nils Kohn[1]*

*[1]Donders Institute, Radboud University, Nijmegen, The Netherlands*

Odors are known to evoke vivid and emotionally salient memories, but evidence for their facilitatory effect on associative memory is mixed and task-dependent. This study aimed to examine how olfactory and auditory contexts influence associative memory, and whether odors enhance performance beyond general sensory stimulation. In Experiment 1, thirty normosmic participants completed a functional MRI (fMRI) associative memory task with encoding and retrieval phases. During encoding, participants viewed neutral images paired with distinct map locations. Each image-location pair was presented three times and accompanied by either an odor (via olfactometer) or a sound (via headphones). Retrieval included three tasks: (i) location recall, (ii) modality recognition with confidence ratings, and (iii) image recall. Both encoding and the first retrieval task were performed inside the scanner. No significant behavioral differences were found between sensory conditions ($p > 0.05$). However, fMRI showed modality-specific activation: olfactory context engaged the piriform cortex and amygdala, while auditory context activated the primary auditory cortex and insula. In Experiment 2, we tested whether odors modulate memory performance compared to a no-stimulation control.

Although analyses are ongoing, preliminary behavioral results suggest enhanced spatial performance in an associative memory task when encoding occurred with odor, reflected in shorter distances to target locations compared to control in high confidence trials. These findings support the idea that odors and sounds engage distinct sensory-memory pathways, and tentatively suggest that odors may improve spatial performance in an associative memory task when contrasted with an unstimulated context, aligning with and extending prior mixed evidence on olfactory memory enhancement.

**abstract citation id: bjaf055.220**

### Beyond the Human Nose: AI in Odor Recognition and Mixture Analysis

*Satnam Singh[1], Doris Gasser[1], Jessica Freiherr[1,2], Andreas T. Grasskamp[1]*

*[1]Sensory Analytics and Technology, Fraunhofer Institute for Process Engineering and Packaging IVV, Giggenhauser Str. 35, 85354 Freising, Germany*
*[2]Department of Psychiatry and Psychotherapy, Friedrich-Alexander-Universität Erlangen-Nürnberg. Germany*

Odor recognition and mixture analysis are complex, non-linear problems where one compound can alter the perceived odor of a mixture. Odor mixtures involve dynamic interactions, with some compounds dominating or only detectable at specific concentrations, creating new sensations when combined. This complexity makes developing a machine learning (ML) model to predict an odor mixture's profile challenging. However, doing so would be valuable, as most everyday smells are mixtures. A significant hurdle is the lack of a standardized dataset, compounded by human subjectivity that adds variability and complicates clear sample labeling. Additionally, the concentration of compounds and the medium used to present odors significantly influence perception and assessment, hindering the generalization of predictions across diIerent datasets.

In this work, we trained a convolutional neural network and a linear classification algorithm, OWSum, to predict the origins and aromas of 16 whisky samples from American and Scotch sources. By combining fast analytical tools with sensory data from 11 experienced panelists, our methods surpassed inter-panelist agreement with promising accuracy, demonstrating previously impossible data-driven sensory assessment in mixtures (up to F1: 0.71, MCC: 0.68, ROCAUC: 0.78). These methods focused only on molecular structures, not considering odor activity values, thresholds, and molecular concentrations. Thus, we would also like to introduce our work "NextML" that aims to bridge this gap by acquiring a comprehensive and a wide spectrum of molecular mixture data, including perceived intensity, thresholds, activity values and pleasantness. This data can be used to train new ML algorithms that extend the current understanding of odor recognition and mixture analysis.

**Funding:** Funding: Bavarian State Ministry for Economic AIairs, Regional Development and Energy (Campus of the Senses), Fraunhofer Internal Program Schnelle Mittelstandsorientierte Eigenforschung (SME)
